# Directed C–H Functionalization of C3-Aldehyde, Ketone, and Acid/Ester-Substituted Free (NH) Indoles with Iodoarenes *via* a Palladium Catalyst System

**DOI:** 10.1021/acs.joc.2c00716

**Published:** 2022-05-24

**Authors:** Yunus Taskesenligil, Murat Aslan, Tuba Cogurcu, Nurullah Saracoglu

**Affiliations:** Department of Chemistry, Faculty of Sciences, Atatürk University, Erzurum 25240, Turkey

## Abstract

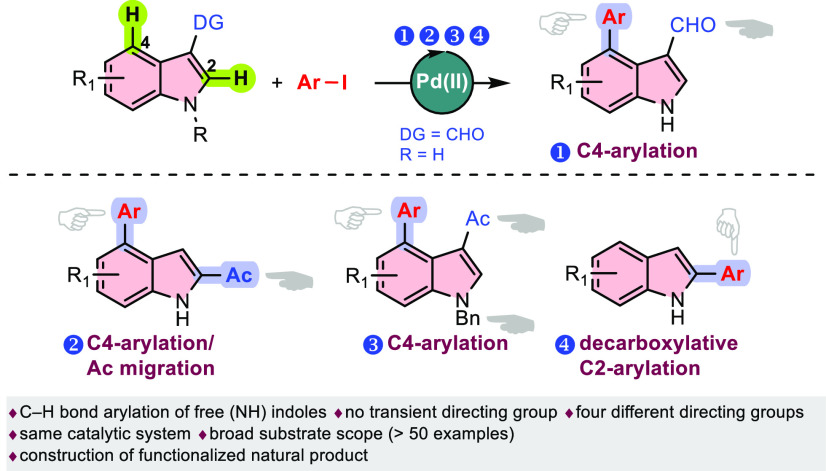

Pd(II)-catalyzed C–H arylations of free (NH) indoles including different carbonyl directing groups on C3-position with aryl iodides are demonstrated. Importantly, the reactions are carried out using the same catalyst system without any additional transient directing group (TDG). In this study, the formyl group as a directing group gave the C4-arylated indoles versus C2-arylation. Using this catalyst system, C–H functionalization of 3-acetylindoles provided domino C4-arylation/3,2-carbonyl migration products. This transformation involves the unusual migration of the acetyl group to the C2-position following C4-arylation in one pot. Meanwhile, migration of the acetyl group could be simply controlled and *N*-protected 3-acetylindoles afforded C4-arylation products without migration of the acetyl group. Functionalization of indole-3-carboxylic acid (or methyl ester) with aryl iodides using the present Pd(II)-catalyst system resulted in decarboxylation followed by the formation of C2-arylated indoles. Based on the control experiments and the literature, plausible mechanisms are proposed. The synthetic utilities of these acetylindole derivatives have also been demonstrated. Remarkably, C4-arylated acetylindoles have allowed the construction of functionalized pityiacitrin (a natural product).

## Introduction

Transition-metal (TM)-catalyzed functionalization reactions through directing group-assisted C–H activation have emerged as a powerful tool for C–C and C–X bond-forming reactions, providing an atom- and step-economical strategy for organic synthesis.^1^ The C–H activation, which represents a paradigmatic change in the field of synthesis of complex heterocyclic and carbocyclic molecules, allows not only specifically functionalizes of the inert C–H bonds but also provides the formation of various compounds by coupling the introduced functional groups.^2^

**Figure 1:**
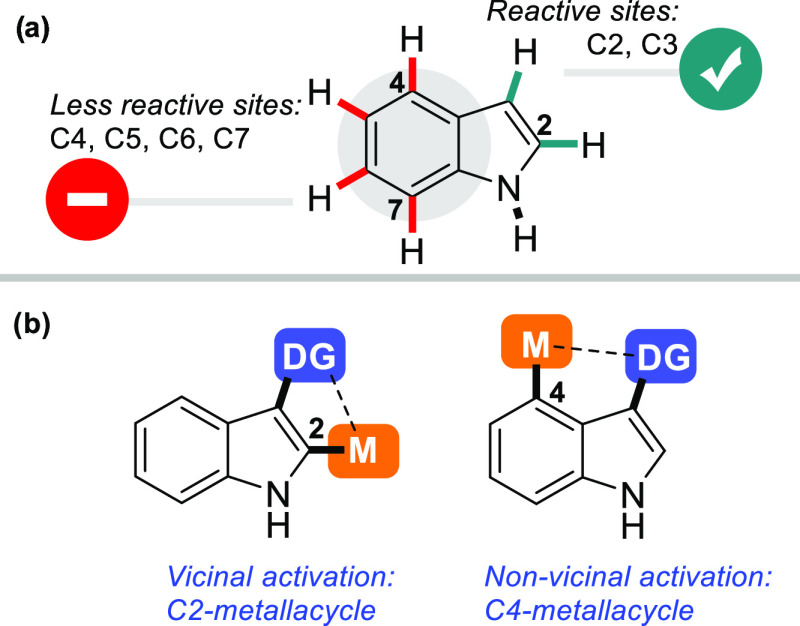
C–H functionalization of indoles and directed C–H bond activation mode examples.

Indole core is found in numerous natural products, pharmaceuticals, materials chemistry, and other bio-relevant compounds and has been recognized as a privileged structure scaffold.^3^ Because of this, enormous efforts have been devoted to the construction of functionalized indoles. Due to the high nucleophilic activity of the pyrrole ring of the indole, most C–H activation reactions primarily occur at the C2 or C3 position of pyrrole moiety (Figure 1a).^4^ As opposed to these positions, C–H functionalization of the low active C4–C7 positions in the benzenoid ring is less scrutinized and remains a long-standing challenge (Figure 1a). In recent years, transition-metal-catalyzed directed C–H activation has been studied as a powerful synthetic tool to access the functionalized indoles at C4–C7 positions.^5^ Especially, Shi,^6^ You,^7^ Yu,^8^ Ackermann,^9^ and others^10^ pioneered the developments in benzenoid functionalization. C–H activation through C3 at the pyrrole ring of indole has two directing modes. The first is vicinal activation, in which the directing group (DG) and the target C–H bond are at vicinal positions of the indole (Figure 1b).^11^ With this activation mode for a suitable DG at the C3-position, a metallacycle is formed after C–H activation, which leads to C2-functionalization *via* the resulting metal–C bond. The second directing mode for DG on the indole C3-position involves nonvicinal activation to form a metallacycle for C4-functionalization (Figure 1b).^7,11a,11b^ Similarly, by installing a directing group at the indole nitrogen, both C2- and C7-activation have also been enabled.^5j,11c^ However, both C5 and C6 positions lie even more remote from a directing group, and therefore, used strategies have mimicked those used for remote meta functionalization. These strategies include template-controlled palladium-catalyzed C–H alkenylation,^8^ C–H alkylation *via* σ-activation,^12^ and copper-catalyzed arylation using diaryliodonium salts as the arylating agent.^13^

**Scheme 1:**
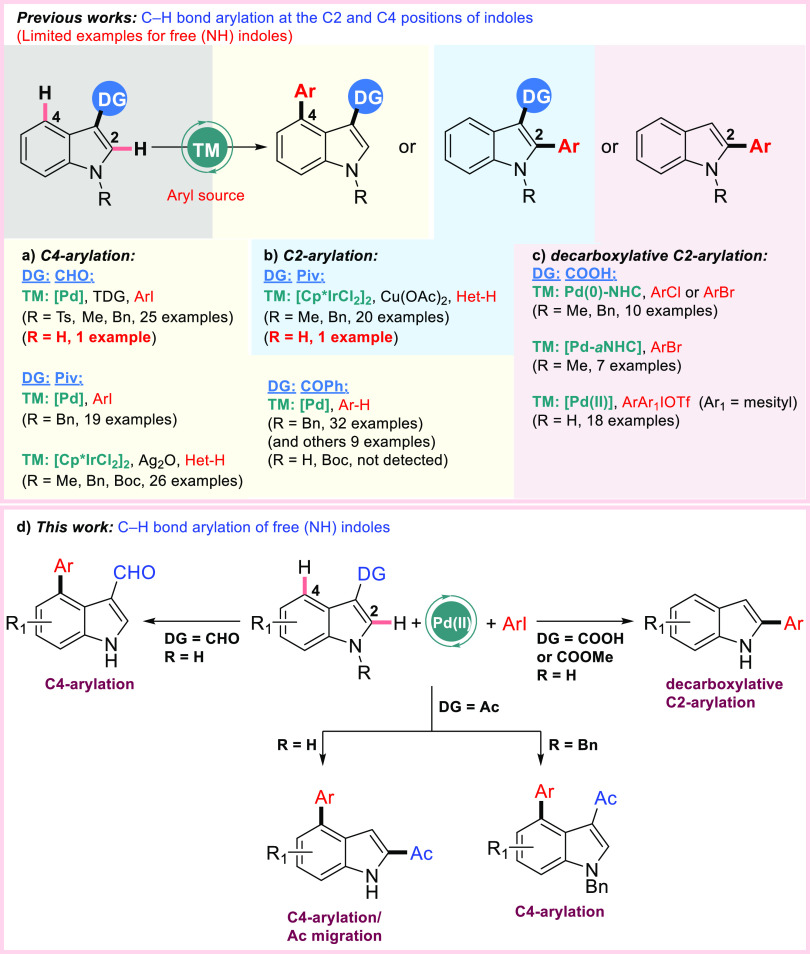
Transition-Metal-Catalyzed Arylation of Indoles *via* C–H Functionalization

Due to the remarkable importance of C4-functionalized indoles in natural products and medicinal chemistry, researchers have concentrated on the direct diversification of relatively less explored C-4 position of indoles *via* C–H activation strategy. Therefore, alkenylation,^10a,14^ acylation,^10c^ amidation,^7b,15^ allylation,^16^ alkylation,^17^ borylation,^5f^ cyclization,^18^ fluoroalkylation,^6e,19^ and halogenation^20^ of indoles at C4-positions have been demonstrated using different transition-metal catalysts (such as Rh, Ir, Pd, Co, and Ru) in the presence of appropriate directing groups. Despite the importance of biaryl compounds, a limited number of arylations at the C4-position through C–H activation have been performed so far. In 2017, Shi’s group installed an elegant Pd(0)-catalyzed strategy for the C4-arylation of *N*-benzylindoles using a pivaloyl directing group (Scheme 1a).^13a^ Almost simultaneously, Yu and Zhang’s group showed a single example (in a yield of 82%) of Pd(II)-C4-arylation of *N*-tosyl-3-fomylindole with methyl 4-iodobenzoate, in which the formyl group was installed in the C3 position of the indole (Scheme 1a).^21^ In this work, 2-amino-2-methylpropanoic acid was used as a transient directing group. Later, Maiti and Volla’s group reported a similar transformation for the C4-arylation of unprotected 3-formylindole (single example) and *N*-protected (methyl, tosyl, and benzyl) 3-formylindoles with aryl iodides using glycine as an inexpensive transient directing group (Scheme 1a).^10d^ Yang and You’s group disclosed an iridium-catalyzed C2/C4-regioselective C–H heteroarylation of indoles with the help of a pivaloyl group at the C3-position (Scheme 1a/b).^7a^ The oxidants Cu(OAc)_2_**·**H_2_O and Ag_2_O have been demonstrated to play a vital role in the C2/C4-regioselectivity. Recently, Punniyamurthy’s group reported the palladium-catalyzed weak chelation-assisted regioselective C4-arylation of indoles utilizing arenes as the aryl source *via* a twofold C–H activation/C–C bond formation (Scheme 1a).^22^ When using carboxylic acid as the directing group, *N*-heterocyclic carbene (NHC) and abnormal NHC (*a*NHC)-based Pd-catalyzed arylation reactions of *N*-alkylindole-2-carboxylic acids with aryl bromides and aryl chlorides as the coupling partners resulted in decarboxylative C2-arylation (Scheme 1c).^23^ Synthesis of 2-arylindoles was also reported *via* a Pd-catalyzed decarboxylative strategy in water without base, oxidant, and ligand using diaryliodonium salts as the aryl partners (Scheme 1c).^24^ Despite the above background, the examples of the directed C–H arylation of unprotected indoles remain extremely limited and include transient directing group strategies. At this point, it is important to note that An, Li, and Yang’s group recently reported C4-arylation or domino C4-arylation/3,2-carbonyl migration (*via* migration of acetyl substituent from the C3- to C2-indole position) of 3-acetyl indoles *via* the different pathways by tuning either the Pd(I)–Pd(II) pathway or Pd(II) catalysis.^25^ Our simultaneous results complete this research, and also expand its scope, and include new findings. Herein, we now wish to report the arylations for unprotected indoles including formyl, acetyl, carboxylic acid, and methyl ester groups as a directing group at the C3-position with aryl iodides and without a transient directing group using the single Pd(II) catalyst system. With different directing groups, different pathways were observed (Scheme 1d).

## Results and Discussion

In continuation of our research interest in C–H arylation reactions,^26^ we started our investigation, focusing on the C–H arylation reactions of unprotected indole **1a** involving the formyl group at the C3-position as a directing group without a transient directing group. Initially, 1*H*-indole-3-carbaldehyde (**1a**) and iodobenzene (**2a**) were selected as model substrates to screen the reaction conditions (Table 1). When 10 mol % Pd(OAc)_2_ was used as the catalyst and AgOAc (2 equiv) was used as the oxidant at 100 °C, among the employed solvents such as 1,1,1,3,3,3- hexafluoroisopropanol (HFIP), HOAc, 1,2-dichloroethane (DCE), *N*,*N*-dimethylformamide (DMF), toluene (Tol), trifluoroacetic acid (TFA), and 2,2,2-trifluoroethanol (TFE) (Table 1, entries 1–8), TFA gave the desired product **3aa** in 23% yield (Table 1, entry 8). The yields were improved to 47 and 58% when HOAc and TFA were used as the additive, respectively (Table 1, entries 9 and 10). Entries 9 and 10 showed that the use of TFA as a co-solvent was also effective. To test the effect of the co-solvents such as DCE, *N*,*N-*dimethylacetamide (DMA), DMF, Tol, or TFE, further optimization was conducted (Table 1, entries 11–15). Under these conditions, the formation of **3aa** was observed as trace amounts. To our delight, decreasing the reaction time to 3.5 h, we obtained the desired product **3aa** with an 87% isolated yield (Table 1, entries 16 and 17). When the reaction was carried out at 120 °C for 3.5 h, the yield decreased (Table 1, entry 18). The reaction at 65 °C for 15 h did not give the expected increase in yield (Table 1, entry 19). Other oxidants such as Ag_2_CO_3_, Ag_2_O, Cu(OAc)_2_·H_2_O, and AgTFA were also screened, but none of them achieved the same effect as silver acetate (Table 1, entries 20–23). Further experiments revealed that other palladium catalysts such as PdCl_2_ and Pd(PPh_3_)_2_Cl_2_, and Pd(TFA)_2_ in HFIP (with and without TFA) were inferior to Pd(OAc)_2_ (entry 24–27). Also, when the Pd(TFA)_2_/AgOAc catalyst system in HFIP/TFA was used, the C4-arylation proceeded cleanly, and **3aa** was isolated in 87% yield (entry 28). Based on the screening conditions described above, the use of Pd(OAc)_2_ as a catalyst due to inexpensive AgOAc as an oxidant and TFA as an additive in HFIP at 100 °C was determined to be the optimal reaction conditions (entry 17).

**Table 1:**
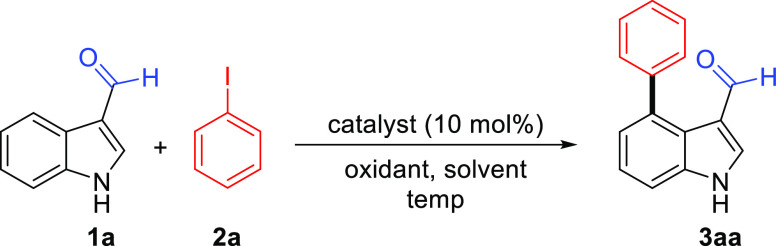
Optimization of Reaction Conditions^a^

entry	catalyst	oxidant	temp (°C)	solvent	additive	time (h)	yield (**3aa**, %)^b^
1–7	Pd(OAc)_2_	AgOAc	100	solvent^c^		10–12	trace
8	Pd(OAc)_2_	AgOAc	100	TFA		10	23
9	Pd(OAc)_2_	AgOAc	100	HFIP	HOAc	10	47
10	Pd(OAc)_2_	AgOAc	100	HFIP	TFA	10	57
11–15	Pd(OAc)_2_	AgOAc	100	HFIP	other solvent^d^	5	trace
16	Pd(OAc)_2_	AgOAc	100	HFIP	TFA	5	70
**17**	**Pd(OAc)**_**2**_	**AgOAc**	**100**	**HFIP**	**TFA**	**3.5**	**87**
18	Pd(OAc)_2_	AgOAc	120	HFIP	TFA	3.5	72
19	Pd(OAc)_2_	AgOAc	65	HFIP	TFA	15	81
20	Pd(OAc)_2_	Ag_2_CO_3_	100	HFIP	TFA	3.5	45
21	Pd(OAc)_2_	Ag_2_O	100	HFIP	TFA	3.5	70
22	Pd(OAc)_2_	Cu(OAc)_2_·H_2_O	100	HFIP	TFA	3.5	trace
23	Pd(OAc)_2_	AgTFA	100	HFIP		5	trace
24	PdCl_2_	AgOAc	100	HFIP	TFA	3.5	71
25	Pd(PPh_3_)_2_Cl_2_	AgOAc	100	HFIP	TFA	3.5	67
26	Pd(TFA)_2_	AgOAc	100	HFIP		3.5	trace
27	Pd(TFA)_2_	AgTFA	100	HFIP		36	74
**28**	**Pd(TFA)**_**2**_	**AgOAc**	**100**	**HFIP**	**TFA**	**3.5**	**87**

a Reaction conditions: **1a** (0.40 mmol), Pd(OAc)_2_ (10 mol %), **2a** (0.80 mmol), oxidant (0.80 mmol), solvent (1 mL), additive (1 mL).

b Isolated yield.

c Solvent: HFIP, HOAc, DCE, DMA, DMF, Tol, or TFE.

d Other solvent: DCE, DMA, DMF, Tol, or TFE.

**Scheme 2:**
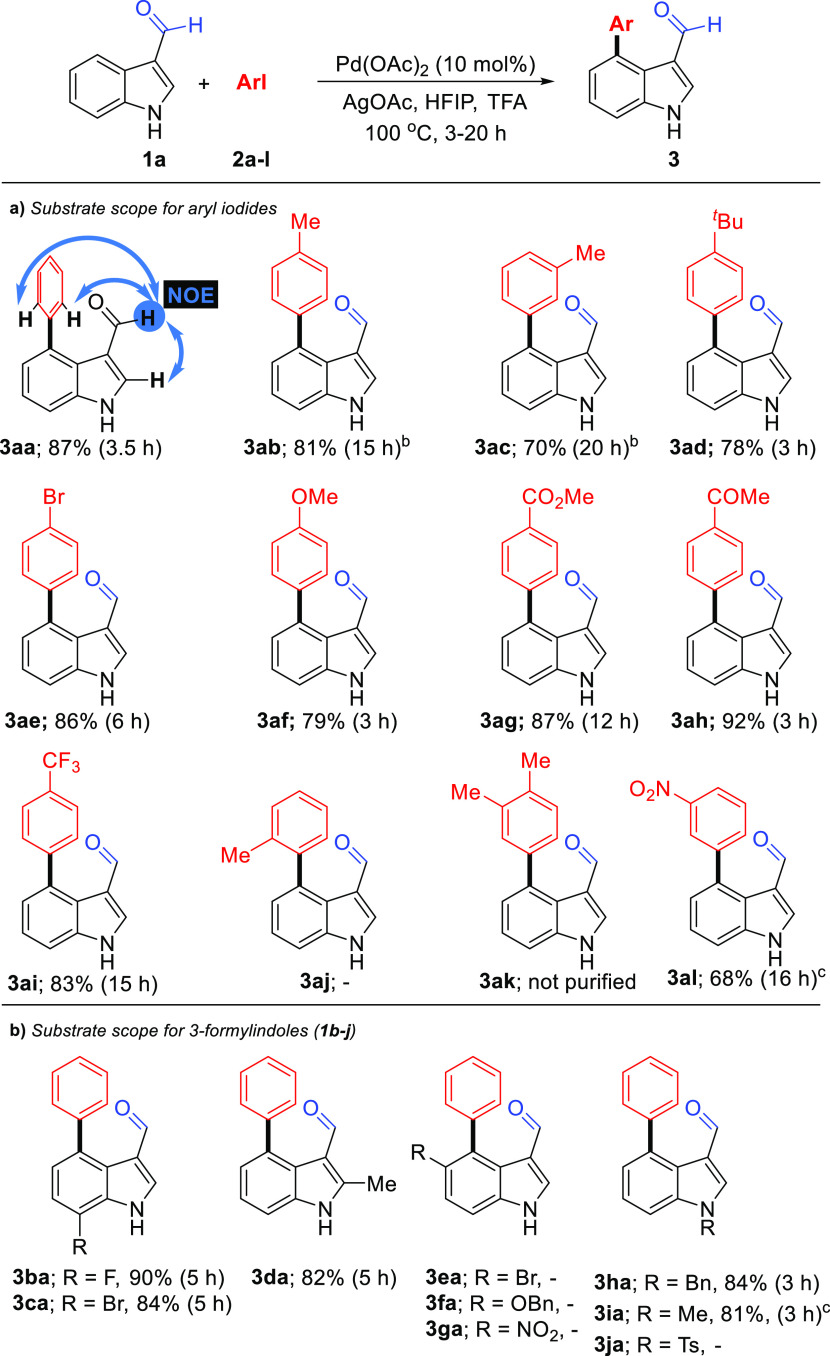
Substrate Scope of Iodoarenes and 3-Formylindoles Reaction conditions: **1a** (0.40 mmol), Pd(OAc)_2_ (10 mol %), **2** (0.80 mmol), AgOAc (0.80 mmol), HFIP (1 mL), TFA (1 mL). At 65 °C. At 120 °C.

With the optimized condition in hand, the substrate scope of this C–H arylation reaction was investigated, and the results are depicted in Scheme 2. Initially, both various electron-rich (Me, *t*-Bu, OMe) and electron-poor (Br, CO_2_Me, COMe, and CF_3_) aryl iodides **2a-l** were subjected to C4 arylation with 1*H*-indole-3-carbaldehyde (**1a**) under the standard conditions. Generally, reactions of aryl iodides possessing diverse substituents at different positions of the phenyl ring proceeded smoothly, and the desired C4 arylated products **3aa-ai** and **3al** were obtained in good to excellent yields (Scheme 2a). *ortho*-Methyl group at the iodoarene is not tolerated, suggesting that a sterically crowded intermediate is being formed during the coupling (Scheme 2a). Using 1-iodo-1,3-dimethylbenzene (**2k**) gave a reaction mixture that could not be purified. Then, the influence of the substitution pattern at the indole ring was investigated. The coupling of iodobenzene (**2a**) with a variety of substituted 1*H*-indole-3-carbaldehydes **1b-j** was tested (Scheme 2b). No arylation products **3ea-ga** were obtained from 5-substituted indoles, indicating that the reaction is very sensitive to steric hindrance at this position. Conversely, substituted indoles at C2, C7, or *N*1 positions readily couple with iodobenzene to give the corresponding C4 arylated products **3ba-da**, **3ha**, and **3ia** in excellent yields. Particularly noteworthy are the 7-halogen substrates in which the presence of the fluorine and bromine substituents at C7 does not hamper the arylation in the C4 position. The structures of C–H arylation products were confirmed by ^1^H and ^13^C NMR spectroscopy and high-resolution mass spectrometry (HRMS). The arylation at C4-position in **1a** was also assigned by a nuclear Overhauser effect (NOE) study. NOE correlations between the C2 hydrogen atom and *ortho*-hydrogen atoms of the C4 phenyl substituent of the aldehyde hydrogen atom within **3a** (see blue arrows) support the relative arylation depicted (Scheme 2a).

**Scheme 3:**
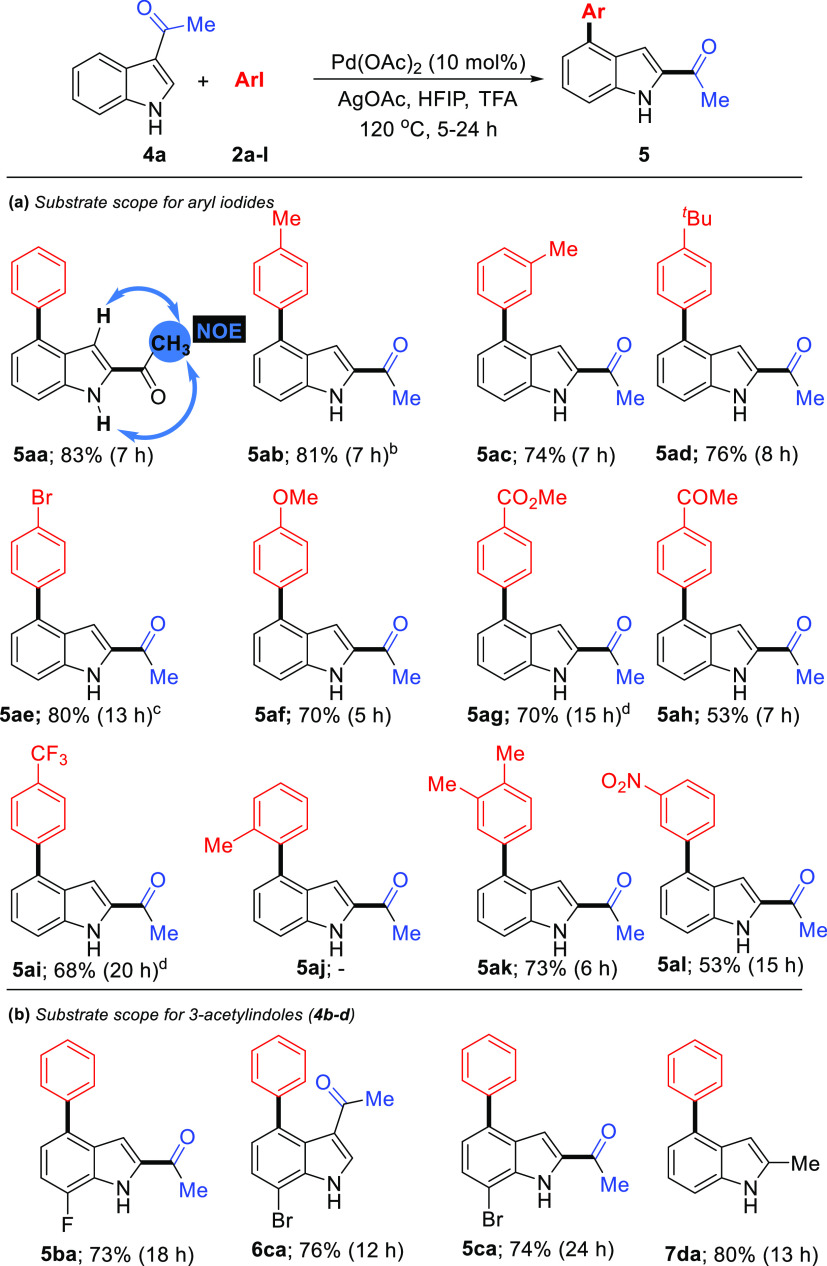
Substrate Scope of Iodoarenes and 3-Acetylindoles Reaction conditions: **1a** (0.40 mmol), Pd(OAc)_2_ (10 mol %), **2** (0.80 mmol), AgOAc (0.80 mmol), HFIP (1 mL), TFA (1 mL). At 130 °C. At 100 °C. At 110 °C.

Notably, 3-acetylindole (**4a**) was subjected to reaction with **2a** under the determined optimized reaction conditions for indole-3-aldehydes, and the desired product **5aa** was obtained in 83% yields (Scheme 3a). Recently, An, Li, and Yang’s group researched the same reaction and reported this domino C4-arylation/3,2-carbonyl migration and structures of corresponding products.^25^ The structure of products was assigned by NMR spectra and HRMS. At the ^1^H NMR spectrum, the chemical shift of the C2-H (low-field) and C3-H (high-field) protons for indoles is a characteristic indication. The C3-H resonance (δ 7.34 ppm) of **5aa** appeared at a lower field than the C2-H resonance (δ 7.87 ppm) of **4a**. Comparison of the NMR spectra of **4a** and **5aa**, the disappearance of characteristic C2-H resonance, and the appearance of a new C3-H resonance at high-field indicated the formation of **5aa**, which reveals that the ketone group is migrated under these conditions. Additionally, for domino C4-arylation/1,2-carbonyl migration product **5aa**, structure assignment was confirmed according to NOE signals (see blue arrows) between acetyl methyl protons and C3–H/N–H protons (Scheme 3a). The evaluation of the substrate scope for this transformation is depicted in Scheme 3a. A wide variety of aryl iodides **2a-l** (except **2j**) were also well tolerated by the palladium catalyst to deliver domino C4-arylation/1,2-carbonyl migration products **5ab-al** (except **5aj**). For example, aryl iodides **2b-d** with the electron-donating groups at the para and meta positions, such as methyl and *tert*-butyl, promoted this transformation smoothly. The corresponding products (**5ab-ad**) were obtained in excellent yields (74–81%). *para*-Methoxy-substituted iodobenzene gives the product **5af** in a high yield (70%). In contrast, aryl iodides **2e** and **2g-i** substituted with electron-withdrawing groups, such as −Br, −COOMe, −COMe, and −CF_3_, also reacted with **4a** smoothly, giving the corresponding products **5ae** and **5ag-ai** in 53–80% yields. When *ortho*-methyl-substituted iodobenzene **5j** was employed under standard conditions, no corresponding product **5aj** was determined. This entry indicated that the steric hindrance of ortho-substitution had a significant effect on the progress of the reaction. In addition, product **5al** was obtained in a low yield (53%) when the −NO_2_ substitution as a strong electron-withdrawing substituent exists on the meta position. When 3,4-dimethyliodobenzene (**2k**) was used as a substrate, the corresponding product **5ak** was obtained in good yield (73%). To further widen the scope of this domino C4-arylation/3,2-carbonyl migration strategy, 3-acetylindoles **4b-d** with iodobenzene (**2a**) were subjected to optimized reaction conditions (Scheme 3b). Also, 3-acetyl-7-fluoro-1*H*-indole (**4b**) worked well to yield **5ba** in good yield (73%). Interestingly, 3-acetyl-7-bromo-1*H*-indole (**4c**) afforded C4-arylated product **6ca** (76% yield) in which the acetyl group did not migrate at 120 °C for 12 h, while domino C4-arylation/3,2-carbonyl migration product **5ca** was obtained when the reaction was performed at 120 °C for 24 h. In the case of C2-protected indole, 3-acetyl-2-methyl-1*H*-indole (**4e**) provided C4-arylated product **7da** with directing group removal.

**Scheme 4:**
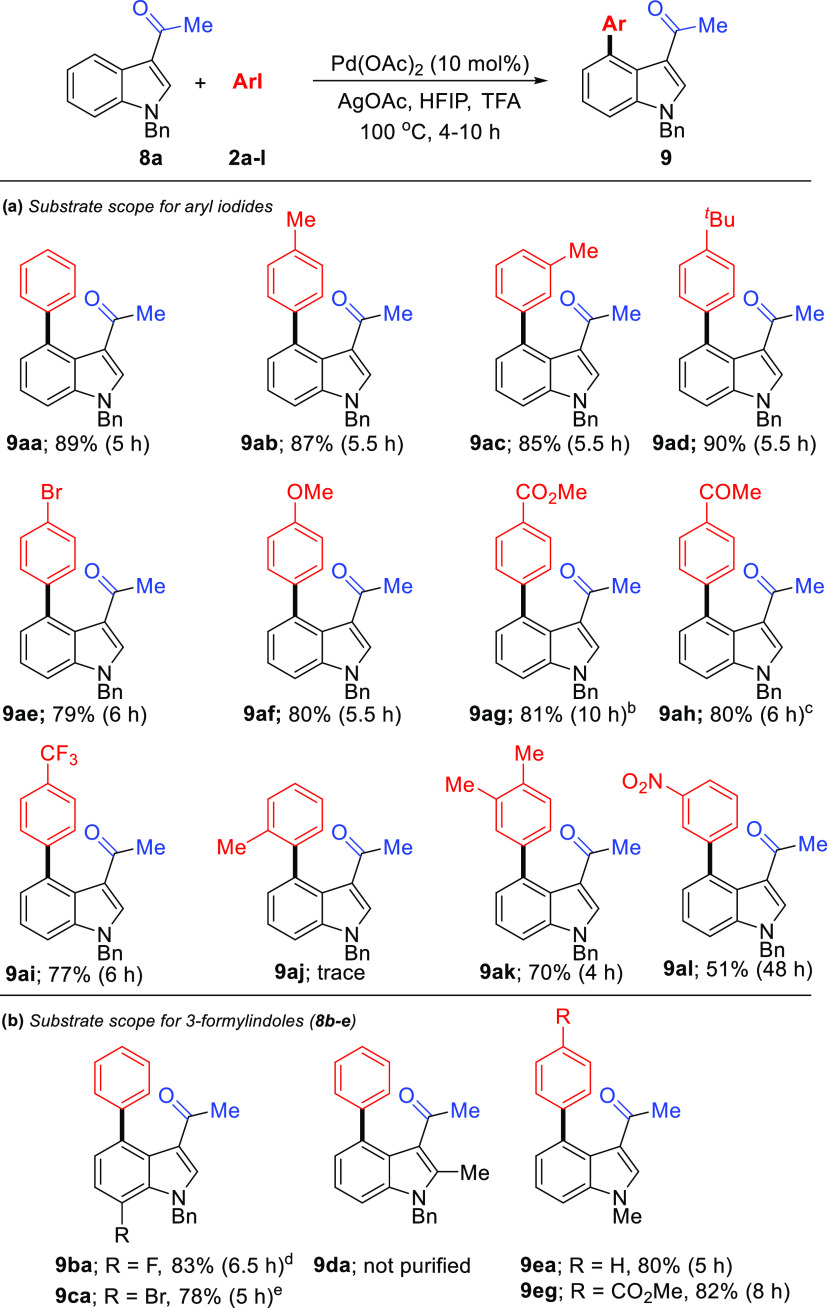
Substrate Scope of Iodoarenes and 3-Acetyl-*N*-Benzylindoles Reaction conditions: **1a** (0.20 mmol), Pd(OAc)_2_ (10 mol %), **2** (0.40 mmol), AgOAc (0.40 mmol), HFIP (1 mL), TFA (1 mL). At 110 °C. At 120 °C. At 90 °C. At 75 °C.

Shi’s group reported C4/C5-arylation (single examples) in a lower yield of an *N*–Bn-protected indole bearing directing groups at the C3 position, such as formyl, acetyl, and isobutyryl substituents.^13^ To confirm the importance of the NH-unprotected indoles for this unusual DG migration, we investigated the reaction between 3-acetyl-*N*-benzylindole **8a** with a variety of aryl iodides **2a-l** containing a wide variety of electron-donating or electron-withdrawing substituents under the same optimized reaction conditions (Scheme 4a). No significant electronic effect on the reaction progress was observed. Aryl iodides with substituents on meta, para-positions delivered corresponding C4-arylation products **9aa-al** in good to excellent yields. But 2-iodotoluene (**9j**) led to a trace amount of product **9aj**. Substrates **8b** and **8c** containing fluoro and bromo participated in C4-arylation smoothly in excellent yields (Scheme 4b). Compared to 3-acetyl-*N*-benzylindole (**8a**), 3-acetyl-*N*-methylindole (**8e**) gave C4 arylation products **9ea** and **9eg** in almost similar yields. However, the presence of the methyl group on the C2-position of indole led to an unpurified reaction mixture (Scheme 4b, for **9d**).

**Scheme 5:**
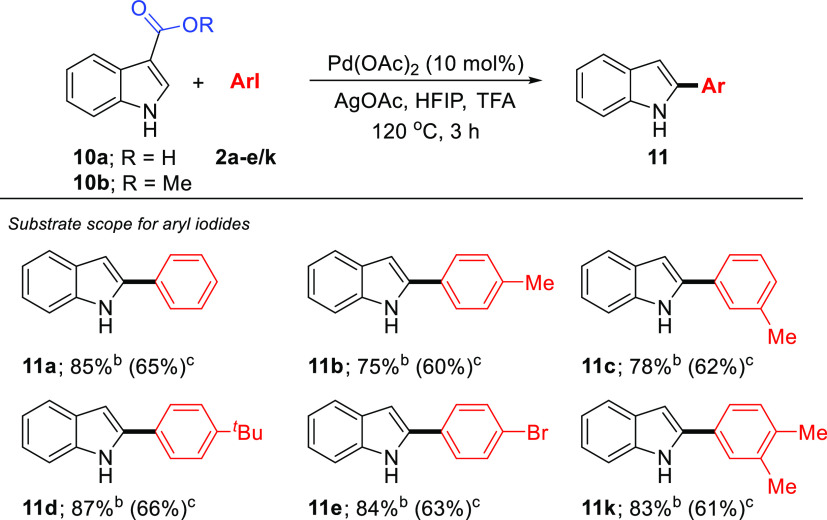
Substrate Scope of Iodoarenes and 1*H*-Indole-3-Carboxylic Acid or Methyl 1*H*-Indole-3-Carboxylate Reaction conditions: **10a** (or **10b**) (0.40 mmol), Pd(OAc)_2_ (10 mol %), **2** (0.80 mmol), AgOAc (0.80 mmol), HFIP (1 mL), TFA (1 mL). For R = H. For R = Me.

With the catalyst system in hand, we next examined the scope of acid/ester-directed C–H arylation with iodoarenes using *N*-unprotected 1*H*-indole-3-carboxylic acid (**10a**) and methyl 1*H*-indole-3-carboxylate (**10b**) (Scheme 5). First, the indole-3-carboxylic acid (**10a**) without substituents on position *N*1 with aryl iodides (**2a-e** and **2k**) was tested. All gave the corresponding decarboxylative C2-arylation products **11a-e** and **11k** in high yields (75–87%) with complete site selectivity.^27−30^ However, the methyl 1*H*-indole-3-carboxylate (**11b**) was effective and gave the same products **11a-e** and **11k** successfully, but in moderate yields (60–66%). Since this catalytic process yields decarboxylative C2-arylation products, we have limited the scope of aryl iodides.

**Scheme 6:**
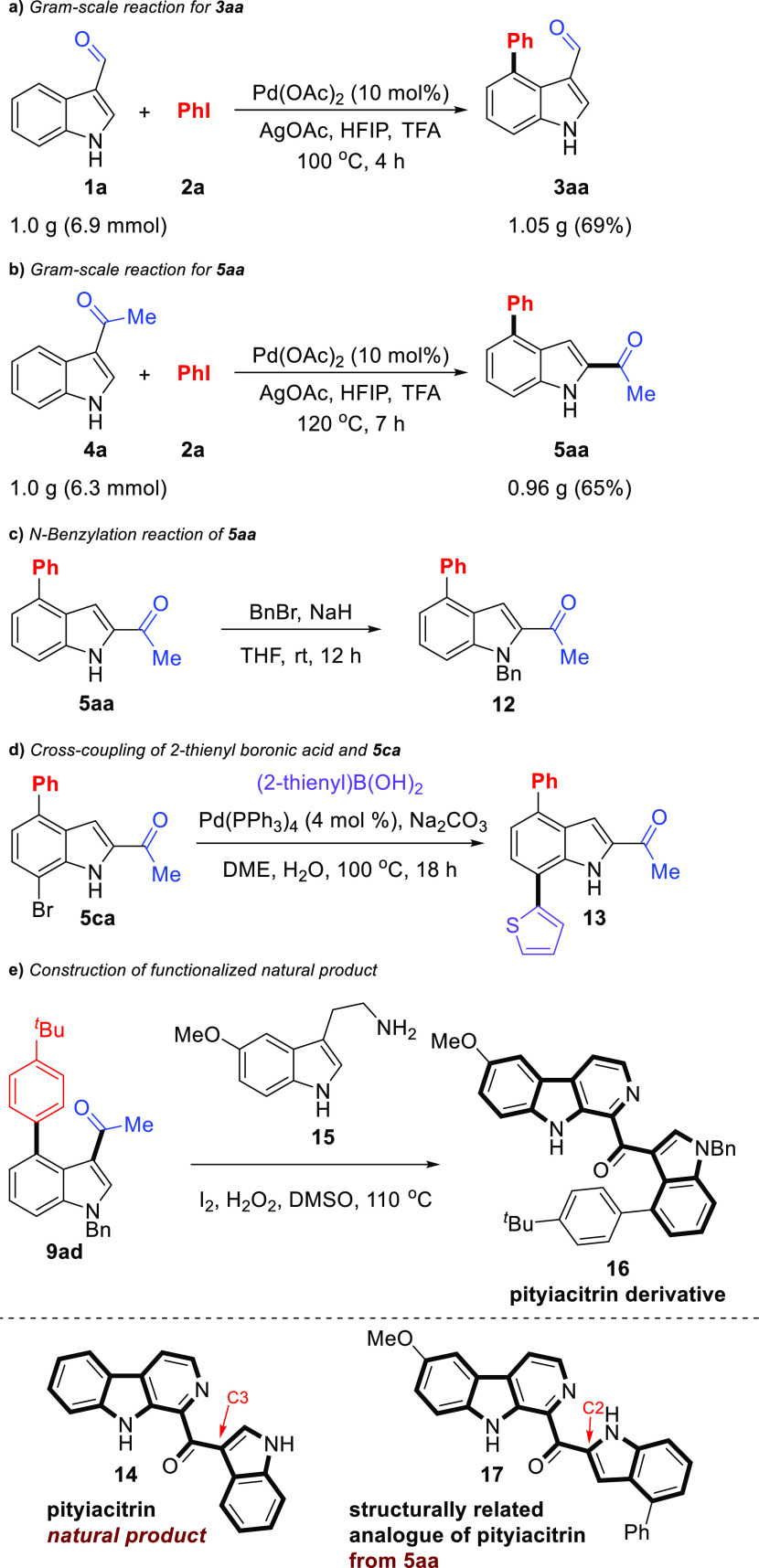
Gram-Scale Reaction and Synthetic Applications

To explore the practical utility of this C(sp^2^)–H arylation reaction, a gram-scale reaction of 3-formylindole (**1a**) was carried out under the standard conditions (Scheme 6a). The desired arylation product **3aa** could be obtained in 69% yield. Also, to confirm the synthetic utility of this domino C4-arylation/1,2-carbonyl migration process, a gram-scale reaction of 3-acetylindole (**4a**, 6.3 mmol) and iodobenzene (**2a**, 25.1 mmol) was conducted under the standard conditions. A total of 0.98 g of compound **5aa** was obtained in a satisfying yield (65%), which was comparable with the small-scale reaction (Scheme 6b). Furthermore, the potential applications of both domino C4-arylation/1,2-carbonyl migration products and C4-arylation products as useful synthetic blocks are illustrated (Scheme 6c–e). To perform structure confirmation as well as synthetic diversification, the benzylation reaction of **5aa** gave the *N*1-benzylated indole derivative **12** in good yields (Scheme 6c). Comparison of the NMR spectra of **12** and **9aa** is important to confirm that the acetyl group does not migrate during the arylation of 3-acetyl-*N*-benzylindole **8a** under the standard conditions. Besides C–H arylation on the C4-position of the indole ring, the bromine group on the 7-position of **5ca** has a potential derivatization site. The classical palladium-catalyzed Suzuki–Miyaura coupling of **5ca** with thiophene-2-boronic acid provided **13** in excellent yield (89%) (Scheme 6d).^31^ Pityiacitrin (**14**) is a 1-indolyl-β-carboline alkaloid isolated from various sources and has also been tested for various biological activities.^32^ To demonstrate utility in the synthesis of the functionalized natural product of C4-arylated acetylindoles, the synthesis of the substituted pityiacitrin starting from **9ad** was carried out successfully (Scheme 6e). For this, **9ad** and 5-methoxytryptamine (**15**) were reacted with 1.0 equiv of I_2_ and 1.5 equiv of H_2_O_2_ in dimethyl sulfoxide (DMSO) at 100 °C for 24 h to form substituted pityiacitrin **16**. The protocol involves Kornblum oxidation to form indolylglyoxal, Pictet–Spengler condensation with tryptamine to yield dihydro-β-carboline, and finally, aromatization of dihydro-β-carboline to provide the desired product **16**. This one-pot process successfully led to the synthesis of structurally related analogue **17** of pityiacitrin from **5aa** (Scheme 6e).

**Scheme 7:**
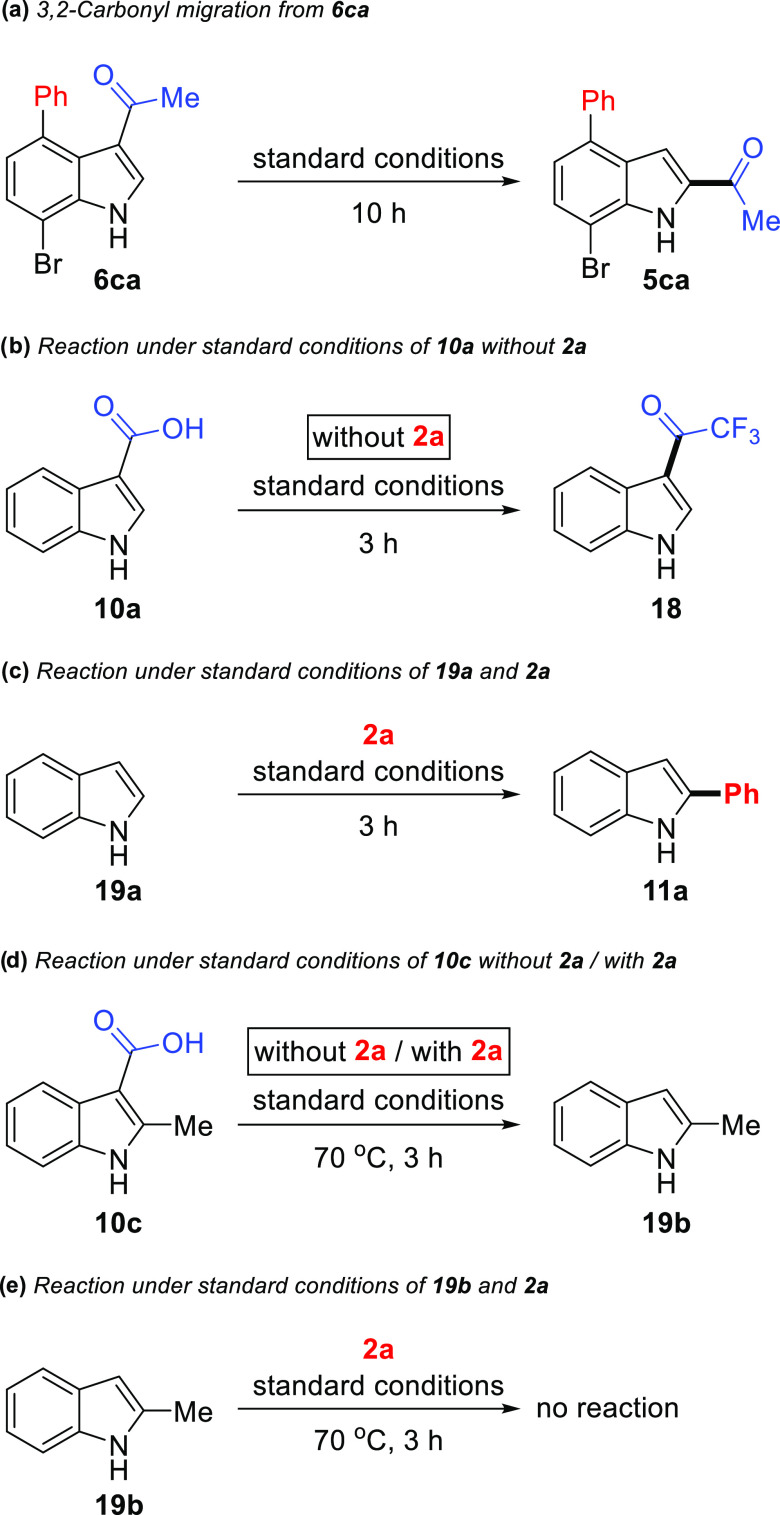
Control Experiments

To further understand both structure characterization and the progress of these reactions, several control experiments were performed. During arylation of **4c**, product **6ca** was also isolated in 76% yield (Scheme 3b). Under the standard conditions, **6ca** itself provided 3,2-carbonyl migration product **5ca** in 74% yield (Scheme 7a). In fact, the C–H arylation reaction of *N*-unprotected 3-acetylindoles **4a-d** can follow two paths: respectively, migration/arylation or arylation/migration. This result strongly supports both the migration of the acetyl group after the arylation reaction and the reported results of An, Li, and Yang’s group.^25^ To understand the mechanism of the decarboxylative arylation reaction, several control experiments were performed (Scheme 7b–e). First, indole-3-carboxylic acid (**10a**) (or methylindole-3-carboxylate (**10b**)) was subjected to standard reaction conditions without using substrate **2a**, wherein we isolated 3-acylation product **18** in 75% (or 70%) yield as a sole product (Scheme 7b).^33^ We first assume that indole-3-carboxylic acid yields indole (**19a**) *via* palladium-catalyzed decarboxylation. Then, product **18** is formed from the acylation of indole (**19a**) with TFA. In addition, when indole (**19a**) and **2a** were reacted under the standard reaction conditions, 2-phenylindole (**11a**) was obtained in 70% yield (Scheme 7c). These results indicate that indole (**19a**) might be the key intermediate for the decarboxylative arylation reaction. Furthermore, the reaction of 2-methylindole-3-carboxylic acid (**10c**)^34^ without **2a**/with **2a** under standard reaction conditions gave 2-methylindole (**19b**) *via* decarboxylation (Scheme 7d). But the treatment of **19b** with **2a** did not provide any product (Scheme 7e). These experiments showed that C–H arylation could not proceed *via* C2 protection under our standard conditions.

**Scheme 8:**
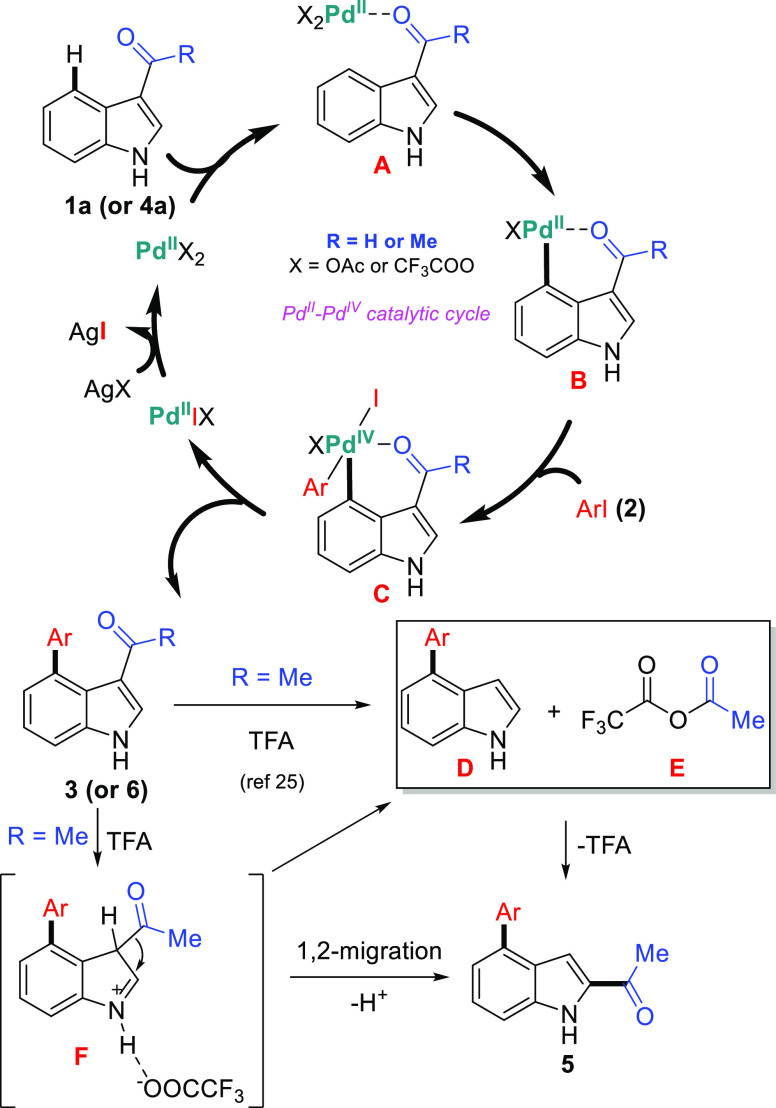
Proposed Pd^II^-Pd^IV^ Catalytic Cycle for C4-Arylation and Migration Mechanism

To probe the role of TFA in C4-arylation, optimization experiments were carried out (Table 1, especially entries 17 and 28). The role of TFA can be categorized into two aspects: (i) Pd(OAc)_2_ could be readily converted to Pd(TFA)_2_ to catalyze reactions (Table 1, entries 8, 10, 16–21, 24, and 25); (ii) it may affect the reaction rate or by increasing the solubility of the reaction mixture (Table 1, entries 26–28). Based on previous reports,^25,35^ our findings, and control experiments, possible catalytic mechanisms are illustrated in Scheme 8. Initially, the active catalytic species **A** is formed from Pd(II) catalyst (Pd(TFA)_2_, a catalyst formed *in situ* from Pd(OAc)_2_ and TFA) and 3-formylindole (**1a**) (or 3-acetylindole) (or **4a**) in the presence of AgOAc, followed by the C–H bond activation to form the cyclometalated Pd(II) intermediate **B**. The intermediate **B** further underwent oxidative addition with aryl iodide **2** to produce diaryl Pd(IV) species **C**. Reductive elimination of **C** produced the desired C4-arylated product **3** (or **6**) and regeneration of the active Pd(II) species by AgOAc to regenerate the catalytic cycle. To explain carbonyl migration, An, Li, and Yang’s group conducted many independent experiments and mechanism studies and proposed a plausible reaction pathway.^25^ Accordingly, after the formation of Pd(II)-catalyzed C4-arylation product **6**, the 3,2-carbonyl migration (or Friedel–Crafts acyl rearrangement) process takes place. The first step would be a reaction between **6** and TFA, yielding the product **D** and mixed (or unsymmetrical) acid anhydride **E***via* protonation and reverse Friedel–Crafts process. Next, TFA (or Pd(TFA)_2_)-promoted intermolecular Friedel–Crafts reaction takes place between **D** and **E** to yield selectively migration product **5** through acylation rearomatization. This unusual Friedel–Crafts acyl rearrangement is not reversible. We speculate that C3-acetylated product **6** was the kinetically controlled product, whereas C2-acetylated product **5** was the thermodynamically controlled product. NH-free 3-acetylindoles underwent unusual migration of the acetyl group to the C2-position following C4-arylation in one pot, whereas *N*-alkylated 3-acetylindoles showed C4-arylations without migration. These results indicate that an alkyl group at the nitrogen atom of the indoles plays a crucial role to prevent the migration of the acetyl group. We believe the existence of iminium intermediate **F***via* a hydrogen-bond interaction between the CF_3_COO^–^ and the NH-free indole, which could promote both reverse Friedel–Crafts process and 1,2-acetyl migration process.

**Scheme 9:**
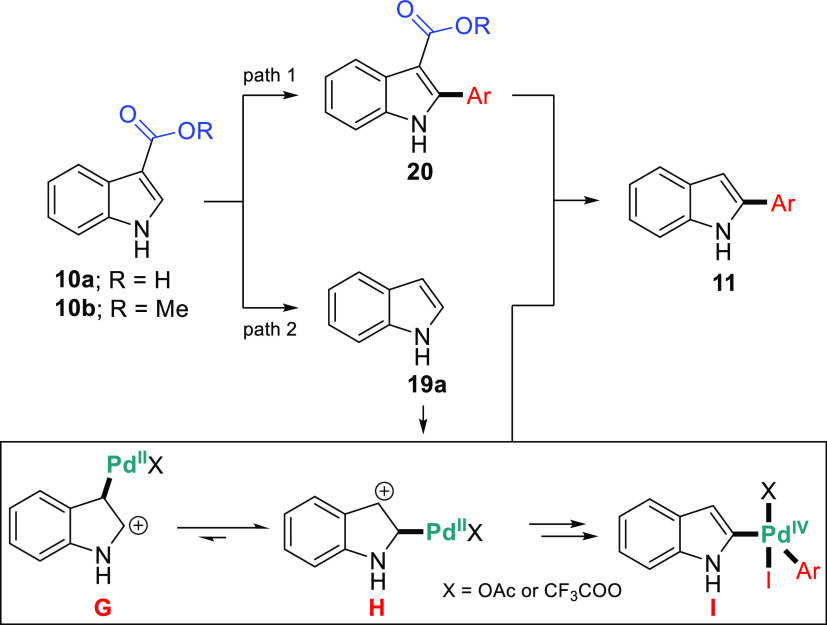
Proposed Pd^II^-Pd^IV^ Catalytic Cycle for C2-Arylation

Two different pathways for the formation of decarboxylative C2-arylated indoles **11**, through either the C–H arylation/decarboxylation process (path 1) or the decarboxylation/C–H arylation process (path 2), could be evaluated (Scheme 9). Based on the above control results and literature reports,^26,36^ a plausible reaction pathway is path 2. In this context, the mechanism involves initial palladation at C3 followed by palladium migration to C2 *via* the Pd(II)/Pd(IV) pathway. Accordingly, this reaction is progressing to yield aryl-palladium intermediate indole cations **G** and **H**, followed by oxidative addition of palladium(II) to the ArI. This aryl-palladium intermediate **I** undergoes sequential reductive elimination to afford the C2-arylated product **11** and PdIOAc or PdI_2_ (from 2 turnovers). Finally, the catalytically active Pd(OAc)_2_ (or Pd(TFA)_2_) for the cycle is regenerated from inactive PdIOAc or PdI_2_*via* silver salt and TFA.

## Conclusions

In summary, we have reported Pd(II)-catalyzed protocol for accessing arylated indole scaffolds utilizing iodoarenes as the aryl source *via* the C–H bond activation of *N*-unprotected indoles with the aid of readily accessible carbonyl directing groups (aldehyde, acetyl, carboxylic acid, and methyl ester) at the C3 position. The protocol is operationally simple and utilizes Pd(OAc)_2_ as a catalyst, AgOAc as an oxidant, and TFA as an additive in HFIP at 65–120 °C. The substrate scope is broad and displays excellent selectivity (C4- arylation for 3-formylindoles and *N*-protected 3-acetylindoles, C4-arylation including 3,2-carbonyl migration for 3-acetylindoles, C2-arylation *via* decarboxylation/arylation steps for both indole-3-carboxylic acid and methylindole-3-carboxylate). Based on the control experiments and the literature, plausible mechanisms are proposed *via* Pd(II)/Pd(IV) catalytic cycles. To show the synthetic utility of both our catalytic system and the arylation products, gram-scale reaction and synthetic applications were performed. In this context, C4-arylated acetylindoles allowed us to construct the functionalized and structural-related analogues of pityiacitrin.

## Experimental Section

### General Information

Unless otherwise mentioned, all reagents and solvents from commercial sources were used without further purification. NMR spectra were recorded in CDCl_3_, DMSO-*d*_6_, or acetone-*d*_6_ solvents at 400 MHz (^1^H) and 100 MHz (^13^C), respectively. Chemical shifts (δ) are reported in parts per million (ppm), using the residual solvent peak in CDCl_3_ (δ = 7.26 ppm for ^1^H NMR and δ = 77.0 ppm for ^13^C NMR), DMSO-*d*_6_ (δ = 2.50 ppm for ^1^H NMR and δ = 39.4 ppm for ^13^C NMR), and acetone-*d*_6_ (δ = 2.05 ppm for ^1^H NMR and δ = 29.8 ppm for ^13^C NMR) as an internal standard, and coupling constants (*J*) are indicated in hertz (Hz). Signal multiplicities are abbreviated as s = singlet, d = doublet, t = triplet, q = quartet, dd = doublet of doublets, m = multiplet, and br = broad. High-resolution mass spectrometry (HRMS) of all compounds was performed using a quadrupole time-of-flight (QTOF) spectrometry device. Column chromatography was performed using silica gel (70–230 mesh).

### General Procedures

#### General Procedure A: Preparation of 1*H*-Indole-3-carbaldehydes (**1b-g**)^37^

1*H*-Indole-3-carbaldehydes **1b-g** were prepared according to the reported literature method.^37^ Pyrophosphoryl chloride (0.9 mL, 9.4 mmol, 1.1 equiv) was added dropwise to a stirred mixture of *N,N*-dimethylformamide (2.8 mL, 36.7 mmol, 4.3 equiv) at 10–20 °C. To this mixture, a solution of indole substrate (8.5 mmol) in *N,N*-dimethylformamide (1.67 mL, 5 M for indole substrate) was added slowly, keeping the temperature at 20–30 °C. The mixture was then stirred in a preheated oil bath at 35 °C for 45 min. After completion of the reaction, the mixture was cooled to room temperature and carefully quenched with crushed ice (3.5 g). The mixture was stirred vigorously, and further crushed ice (3.5 g) was added, followed by a solution of NaOH (3.77 g, 94.3 mmol, 11 equiv) in water (10 mL). Then, the mixture was heated under reflux for 15 min, and the precipitate was filtered, washed with water (3 × 20 mL), and dried in vacuo to afford 1*H*-indole-3-carbaldehydes **1b-g**.

#### General Procedure B: Preparation of 3-Acetyl-1*H*-indoles (**4a-d**)^38^

3-Acetyl-1*H*-indoles **4a-d** were prepared according to the reported literature method.^38^ SnCl_4_ (0.75 mL, 6 mmol, 1.2 equiv) was added dropwise into a stirred solution of 1*H*-indole derivative (5 mmol) in dichloromethane (10 mL, 2 M for 1*H*-indole derivative) at 0 °C in an inert atmosphere of N_2_. The resultant mixture was warmed to room temperature and stirred for 30 min, then acetic anhydride (510 mg, 0.5 mL, 5 mmol, 1 equiv) was added, followed by nitromethane (7.5 mL), and stirred for 2 h at room temperature. After the completion of the reaction, the reaction was quenched by the addition of ice and water (20 mL), then extracted with dichloromethane (3 × 30 mL), and dried over anhydrous Na_2_SO_4_. The solvent was evaporated, and the crude product was then purified by silica gel chromatography to give 3-acetyl-1*H*-indoles.

#### General Procedure C: Preparation of *N*-Alkyl Indoles (**1h-i** and **8a-d**)^10a^

To a suspension of NaH (1.1 mmol, 1.1 equiv, 60% dispersion in mineral oil) in THF at 0 °C, a solution of 1*H*-3-acetylindole (or 1H-indole-3-carbaldehyde) (1 mmol) in THF (5 mL, 0.2 M for 1*H*-3-acetylindole or 1*H*-indole-3-carbaldehyde) was added dropwise. Benzyl bromide (methyl iodide for methylation) (1.1 mmol, 1.1 equiv) was then added dropwise to this solution and stirred for 12 h at room temperature. After completion, the reaction was quenched with water and extracted by EtOAc (2 × 30 mL). The combined organic phase was dried over Na_2_SO_4_, then concentrated under reduced pressure, and the residue was purified on silica gel column chromatography to provide the corresponding *N*-alkyl 3-acetyl indoles (or *N*-alkyl 3-carbaldehydes) indoles.

#### General Procedure D: C–H Arylation

The indole material **1** (or **4**, or **8**, or **10**) (0.4 mmol), Pd(OAc)_2_ (9 mg, 40 μmol, 10 mol %), and AgOAc (133 mg, 0.8 mmol, 2 equiv) were weighed in air and placed in a sealed tube (15 mL) with a magnetic stir bar. To the reaction mixture, aryl iodide **2** (0.8 mmol, 2 equiv) and HFIP/TFA (2 mL, 1:1, v/v, 0.2 M for the indole material **1** (or **4**, or **8**, or **10**)) were added. The reaction mixture was then stirred in a preheated oil bath at 65–130 °C for 3–24 h. Upon completion, the reaction mixture was cooled to room temperature, the solvents were removed under reduced pressure, and the resulting mixture was purified by a silica gel column chromatography column to obtain the corresponding C–H arylation product using hexane/EtOAc as the eluent.

#### Spectral Data of Starting Materials

##### 7-Fluoro-1*H*-indole-3-carbaldehyde (**1b**)^39^

Off-white solid, mp: 140–141 °C; ^1^H NMR (400 MHz, DMSO-*d*_6_): δ 12.70 (bs, NH, 1H), 9.97 (s, CHO, 1H), 8.37 (s, CH, 1H), 7.91 (d, *J* = 7.8 Hz, CH, 1H), 7.24–7.16 (m, CH, 1H), 7.13–7.07 (m, CH, 1H). ^13^C{^1^H} NMR (100 MHz, DMSO-*d*_6_): δ 185.2, 149.0 (d, *J* = 245.2 Hz), 139.0 (s), 127.7 (d, *J* = 4.5 Hz), 124.7 (d, *J* = 13.2 Hz), 122.8 (d, *J* = 6.0 Hz), 118.7, 116.9 (d, *J* = 3.6 Hz), 108.5 (d, *J* = 15.9 Hz).

##### 7-Bromo-1*H*-indole-3-carbaldehyde (**1c**)^40^

Yellow solid, mp: 166–167 °C; ^1^H NMR (400 MHz, Acetone-*d*_6_): δ 11.32 (bs, NH, 1H), 10.06 (s, CHO, 1H), 8.29 (s, CH, 1H), 8.23 (d, *J* = 8.2 Hz, CH, 1H), 7.51–7.49 (m, CH, 1H), 7.20 (t, *J* = 7.8 Hz, CH, 1H). ^13^C{^1^H} NMR (100 MHz, Acetone-*d*_6_): δ 185.7, 138.5, 136.7, 127.2, 127.0, 124.5, 121.7, 120.9, 105.5.

##### 2-Methyl-1*H*-indole-3-carbaldehyde (**1d**)^41^

White solid, mp: 200–201 °C; ^1^H NMR (400 MHz, Acetone-*d*_6_): δ 10.94 (bs, NH, 1H), 10.18 (s, CHO, 1H), 8.29–8.09 (m, CH, 1H), 7.52–7.34 (m, CH, 1H), 7.27–7.09 (m, CH, 2H), 2.75 (s, CH_3_, 3H). ^13^C{^1^H} NMR (100 MHz, Acetone-*d*_6_): δ 184.6, 148.5, 136.5, 127.1, 123.6, 122.8, 121.3, 115.3, 112.0, 11.8

##### 5-Bromo-1*H*-indole-3-carbaldehyde (**1e**)^40^

White solid, mp: 204–205 °C; ^1^H NMR (400 MHz, Acetone-*d*_6_): δ 11.31 (bs, NH, 1H), 10.02 (s, CHO, 1H), 8.39 (d, *J* = 1.9 Hz, CH, 1H), 8.26 (s, CH, 1H), 7.53 (d, *J* = 8.6 Hz, CH, 1H), 7.40 (dd, *J* = 8.6, 2.0 Hz, CH, 1H). ^13^C{^1^H} NMR (100 MHz, Acetone-*d*_6_): δ 185.4, 139.0, 137.0, 127.3, 127.2, 124.6, 119.4, 116.1, 115.0.

##### 5-(Benzyloxy)-1*H*-indole-3-carbaldehyde (**1f**)^42^

Yellow solid, mp: 108–109 °C; ^1^H NMR (400 MHz, DMSO-*d*_6_): δ 12.10 (bs, NH, 1H), 9.89 (s, CHO, 1H), 8.22 (s, CH, 1H), 7.70 (d, *J* = 2.5 Hz, CH, 1H), 7.52–7.46 (m, CH, 2H), 7.46–7.37 (m, CH, 3H), 7.35–7.29 (m, CH, 1H), 6.97 (dd, *J* = 8.7, 2.5 Hz, CH, 1H), 5.12 (s, CH_2_, 2H). ^13^C{^1^H} NMR (100 MHz, DMSO-*d*_6_): δ 184.8, 154.6, 138.5, 137.4, 132.0, 128.3, 127.6, 127.6, 124.8, 118.0, 113.8, 113.2, 104.0, 69.6.

##### 5-Nitro-1*H*-indole-3-carbaldehyde (**1g**)^40^

Yellow solid, mp: 292–293 °C; ^1^H NMR (400 MHz, DMSO-*d*_6_): δ 10.02 (s, CHO, 1H), 8.93 (s, CH, 1H), 8.56 (s, CH, 1H), 8.14 (d, *J* = 8.2 Hz, CH, 1H), 7.71 (d, *J* = 8.2 Hz, CH, 1H). ^13^C{^1^H} NMR (100 MHz, DMSO-*d*_6_): δ 185.4, 142.7, 141.6, 140.3, 123.5, 119.0, 118.6, 117.0, 113.2.

##### 1-Benzyl-1*H*-indole-3-carbaldehyde (**1h**)^40^

White solid, mp: 107–108 °C; ^1^H NMR (400 MHz, CDCl_3_): δ 9.99 (s, CHO, 1H), 8.46–8.23 (m, CH, 1H), 7.70 (s, CH, 1H), 7.40–7.28 (m, CH, 6H), 7.21–7.16 (m, CH, 2H), 5.35 (s, CH_2_, 2H). ^13^C{^1^H} NMR (100 MHz, CDCl_3_): δ 184.6, 138.6, 137.5, 135.3, 129.1, 128.4, 127.2, 125.5, 124.2, 123.1, 122.2, 118.5, 110.4, 50.9.

##### 1-Methyl-1*H*-indole-3-carbaldehyde (**1i**)^40^

Pale-brown solid, mp: 68–69 °C; ^1^H NMR (400 MHz, CDCl_3_): δ 9.94 (s, CHO, 1H), 8.34–8.30 (m, CH, 1H), 7.60 (s, CH, 1H), 7.41–7.28 (m, CH, 3H), 3.80 (s, CH_3_, 3H). ^13^C{^1^H} NMR (100 MHz, CDCl_3_): δ 184.5, 139.5, 137.9, 125.2, 124.0, 122.9, 121.9, 117.9, 110.0, 33.6.

##### 1-Tosyl-1*H*-indole-3-carbaldehyde (**1j**)^43^

1-Tosyl-1*H*-indole-3-carbaldehyde (**1j**) were prepared according to the reported literature method.^41^ Purple solid, mp: 139–140 °C; ^1^H NMR (400 MHz, CDCl_3_): δ 10.09 (s, CHO, 1H), 8.25 (d, *J* = 7.3 Hz, =CH, 1H), 8.23 (s, =CH, 1H), 7.95 (d, *J* = 7.9 Hz, =CH, 1H), 7.87–7.82 (m, AA′ part of AA′BB′ system, =CH, 2H), 7.44–7.32 (m, =CH, 2H), 7.31–7.26 (m, BB′ part of AA′BB′ system, =CH, 2H), 2.36 (s, CH_3_, 3H). ^13^C{^1^H} NMR (100 MHz, CDCl_3_): δ 185.4, 146.2 (2C), 136.2, 135.2, 134.4, 130.3, 127.2, 126.3, 125.1, 122.6, 122.4, 113.3, 21.6.

##### 1-(1*H*-indol-3-yl)ethan-1-one (**4a**)^10a^

White solid, mp: 189–190 °C; ^1^H NMR (400 MHz, CDCl_3_): δ 8.93 (bs, NH 1H), 8.43–8.37 (m, CH, 1H), 7.87 (d, *J* = 3.0 Hz, CH, 1H), 7.48–7.39 (m, CH, 1H), 7.35–7.23 (m, CH, 2H), 2.56 (s, CH_3_, 3H). ^13^C{^1^H} NMR (100 MHz, CDCl_3_): δ 194.2, 136.6, 132.3, 125.4, 123.6, 122.6, 122.2, 118.2, 111.7, 27.6.

##### 1-(7-Fluoro-1*H*-indol-3-yl)ethan-1-one (**4b**)

White solid, mp: 195–196 °C: ^1^H NMR (400 MHz, Acetone-*d*_6_): δ 11.41 (bs, NH, 1H), 8.28 (d, *J* = 3.0 Hz, CH, 1H), 8.12 (d, *J* = 8.0 Hz, CH, 1H), 7.20–7.14 (m, CH, 1H), 7.01 (dd, *J* = 11.5, 8.0 Hz, CH, 1H), 2.50 (s, CH_3_, 3H). ^13^C{^1^H} NMR (100 MHz, Acetone-*d*_6_): δ 193.2 (d, *J* = 0.9 Hz), 150.3 (d, *J* = 243.8 Hz), 134.5 (d, *J* = 17.1 Hz), 130.4 (d, *J* = 4.4 Hz), 125.8 (d, *J* = 13.4 Hz), 123.2 (d, *J* = 6.0 Hz), 119.3 (d, *J* = 1.6 Hz), 118.9 (d, *J* = 3.7 Hz), 108.6 (d, *J* = 15.8 Hz), 27.5. HRMS (ESI-TOF) *m*/*z*: [M + H]^+^ calcd for C_10_H_9_FNO: 178.0663; found: 178.0659.

##### 1-(7-Bromo-1*H*-indol-3-yl)ethan-1-one (**4c**)^44^

White solid, mp: 191–192 °C: ^1^H NMR (400 MHz, DMSO-*d*_6_): δ 12.16 (bs, NH, 1H), 8.35 (s, CH, 1H), 8.19 (d, *J* = 7.7 Hz, CH, 1H), 7.44 (d, *J* = 7.7 Hz, CH, 1H), 7.12 (t, *J* = 7.7 Hz, CH, 1H), 2.48 (s, CH_3_, 3H). ^13^C{^1^H} NMR (100 MHz, DMSO-*d*_6_): δ 193.0, 135.1, 135.0, 126.9, 125.4, 123.1, 120.7, 117.6, 104.6, 27.4.

##### 1-(2-Methyl-1*H*-indol-3-yl)ethan-1-one (**4d**)^45^

Off-white solid, mp: 201–202 °C: ^1^H NMR (400 MHz, CDCl_3_): δ 8.70 (bs, NH, 1H), 8.03 (d, *J* = 7.8 Hz, CH, 1H), 7.37–7.33 (m, CH, 1H), 7.29–7.19 (m, CH, 2H), 2.76 (s, CH_3_, 3H), 2.67 (s, CH_3_, 3H). ^13^C{^1^H} NMR (100 MHz, CDCl_3_): δ 194.8, 143.7, 134.5, 127.0, 122.4, 122.1, 120.9, 114.7, 110.8, 31.3, 15.5.

##### 1-(1-Benzyl-1*H*-indol-3-yl)ethan-1-one (**8a**)^10a^

White solid; mp 114–115 °C: ^1^H NMR (400 MHz, CDCl_3_): δ 8.36–8.28 (m, CH, 1H), 7.64 (s, CH, 1H), 7.29–7.13 (m, CH, 6H), 7.08–7.04 (m, CH, 2H), 5.23 (s, CH_2_, 2H), 2.41 (s, CH_3_, 3H). ^13^C{^1^H} NMR (100 MHz, CDCl_3_): δ 193.0, 137.1, 135.8, 135.0, 129.1, 128.2, 127.0, 126.5, 123.5, 122.7 (2C), 117.5, 110.2, 50.7, 27.7.

##### 1-(1-Benzyl-7-fluoro-1*H*-indol-3-yl)ethan-1-one (**8b**)

White solid; mp 132–133 °C: ^1^H NMR (400 MHz, CDCl_3_): δ 8.19 (d, *J* = 8.0 Hz, CH, 1H), 7.69 (s, CH, 1H), 7.41–7.29 (m, CH, 3H), 7.22–7.14 (m, CH, 3H), 6.95 (dd, *J* = 12.6, 7.9 Hz, CH, 1H), 5.50 (s, CH_2_, 2H), 2.50 (s, CH_3_, 3H). ^13^C{^1^H} NMR (100 MHz, CDCl_3_): δ 192.9, 149.8 (d, *J* = 245.5 Hz), 136.6, 136.1, 130.1 (d, *J* = 4.1 Hz), 129.0, 128.2, 127.0, 124.9 (d, *J* = 9.1 Hz), 123.1 (d, *J* = 6.7 Hz), 118.5 (d, *J* = 3.9 Hz), 118.0, 109.4 (d, *J* = 17.7 Hz), 53.0 (d, *J* = 6.1 Hz), 27.7. HRMS (ESI-TOF) *m*/*z*: [M + H]^+^ calcd for C_17_H_15_FNO: 268.1132; found: 268.1130.

##### 1-(1-Benzyl-7-bromo-1*H*-indol-3-yl)ethan-1-one (**8c**)

White solid; mp 154-153 °C: ^1^H NMR (400 MHz, CDCl_3_): δ 8.47 (dd, *J* = 8.0, 0.9 Hz, CH, 1H), 7.70 (s, CH, 1H), 7.43 (dd, *J* = 7.6, 0.8 Hz, CH, 1H), 7.36–7.29 (m, CH, 3H), 7.13 (t, *J* = 7.9 Hz, CH, 1H), 7.06–7.01 (m, CH, 2H), 5.85 (s, CH_2,_ 2H), 2.49 (s, CH_3_, 3H). ^13^C{^1^H} NMR (100 MHz, CDCl_3_): δ 192.8, 137.8, 137.4, 133.6, 129.5, 129.1, 129.0, 127.9, 126.3, 123.9, 122.2, 117.1, 104.0, 52.2, 27.7. HRMS (ESI-TOF) *m*/*z*: [M + H]^+^ calcd for C_17_H_15_BrNO: 328.0332; found: 328.0329.

##### 1-(1-Benzyl-2-methyl-1*H*-indol-3-yl)ethan-1-one (**8d**)^10a^

White solid; mp 96–97 °C: ^1^H NMR (400 MHz, CDCl_3_): δ 8.12–8.08 (m, CH, 1H), 7.39–7.31 (m, CH, 5H), 7.29 (dd, *J* = 7.0, 1.4 Hz, CH, 1H), 7.06 (d, *J* = 6.5 Hz, CH, 2H), 5.43 (s, CH_2_, 2H), 2.80 (s, CH_3_, 3H), 2.79 (s, CH_3_, 3H). ^13^C{^1^H} NMR (100 MHz, CDCl_3_): δ 194.8, 144.9, 136.5, 136.1, 129.0, 127.8, 126.5, 125.9, 122.3, 122.1, 120.8, 114.7, 110.0, 46.4, 31.8, 12.7.

##### 1-(1-Benzyl-2-methyl-1*H*-indol-3-yl)ethan-1-one (**8e**)^10a^

White solid; mp 101–102 °C. ^1^H NMR (400 MHz, CDCl_3_): δ 8.43–8.31 (m, CH, 1H), 7.67 (s, CH, 1H), 7.40–7.24 (m, CH, 3H), 3.82 (s, CH_3_, 3H), 2.51 (s, CH_3_, 3H). ^13^C{^1^H} NMR (100 MHz, CDCl_3_): δ 192.9, 137.5, 135.8, 126.2, 123.3, 122.5 (2C), 116.9, 109.6, 33.5, 27.6.

##### 2-Methyl-1*H*-indole-3-carboxylic Acid (**10c**)^34^

(Pink solid; mp: 174–175 °C); ^1^H NMR (400 MHz, DMSO-*d*_6_): 11.86 (bs, NH, OH, 2H), 7.94–7.89 (m, CH, 1H), 7.37–7.31 (m, CH, 1H), 7.11–7.05 (m, CH, 2H), 2.64 (s, CH_3_, 3H). ^13^C{^1^H} NMR (100 MHz, DMSO-*d*_6_): δ 166.7, 144.3, 134.7, 127.2, 121.3, 120.6, 120.4, 111.0, 103.2, 13.7.

#### Spectral Data for C–H Arylation Products

##### 4-Phenyl-1*H*-indole-3-carbaldehyde (**3aa**)

Compound **3aa** was synthesized by following general procedure D using 1*H*-indole-3-carbaldehyde (**1a**, 58 mg, 0.4 mmol) and iodobenzene (**2a**, 90 μL, 0.8 mmol) at 100 °C for 3.5 h and purified by silica gel column chromatography (80:20 hexane/ethyl acetate): **3aa** (76 mg, 87%, a brown solid, mp: 151–152 °C); ^1^H NMR (400 MHz, CDCl_3_): δ 10.05 (bs, NH, 1H), 9.52 (s, CHO, 1H), 8.01 (d, *J* = 2.6 Hz, CH, 1H), 7.57–7.51 (m, CH, 2H), 7.50–7.39 (m, CH, 4H), 7.33 (t, *J* = 7.5 Hz, CH, 1H), 7.20 (d, *J* = 7.5 Hz, CH, 1H). ^13^C{^1^H} NMR (100 MHz, CDCl_3_): δ 187.2, 141.9, 137.1, 135.6, 131.8, 129.0, 128.6, 127.7, 124.2, 123.9, 123.4, 119.2, 111.5. HRMS (ESI-TOF) m/z: [M + H]^+^ calcd for C_15_H_12_NO: 222.0913; found: 222.0914.

##### 4-(*p*-Tolyl)-1*H*-indole-3-carbaldehyde (**3ab**)

Compound **3ab** was synthesized by following general procedure D using 1*H*-indole-3-carbaldehyde (**1a**, 58 mg, 0.4 mmol) and 1-iodo-4-methylbenzene (**2b**, 175 mg, 0.8 mmol) at 65 °C for 15 h and purified by silica gel column chromatography (80:20 hexane/ethyl acetate): **3ab** (76 mg, 81%, a brown solid, mp: 207–208 °C); ^1^H NMR (400 MHz, CDCl_3_): δ 9.52 (bs, NH, 1H), 9.02 (s, CHO, 1H), 8.02 (d, *J* = 3.1 Hz, CH, 1H), 7.46–7.39 (m, CH, 3H), 7.33 (t, *J* = 7.3 Hz, CH, 1H), 7.29–7.25 (m, BB′ part of AA′BB′ system, CH, 2H), 7.18 (dd, *J* = 7.1, 0.7 Hz, CH, 1H), 2.42 (s,CH_3,_ 3H). ^13^C{^1^H} NMR (100 MHz, CDCl_3_): δ 187.3, 138.9, 137.5, 137.0, 135.7, 131.2, 129.3, 128.8, 124.3, 123.9, 123.4, 119.3, 111.1, 21.3. HRMS (ESI-TOF) m/z: [M + H]^+^ calcd for C_16_H_14_NO: 236.1070; found: 236.1071.

##### 4-(*m*-Tolyl)-1*H*-indole-3-carbaldehyde (**3ac**)

Compound **3ac** was synthesized by following general procedure D using 1*H*-indole-3-carbaldehyde (**1a**, 58 mg, 0.4 mmol) and 1-iodo-3-methylbenzene (**2c**, 103 μL, 0.8 mmol) at 65 °C for 20 h and purified by silica gel column chromatography (80:20 hexane/ethyl acetate): **3ac** (65 mg, 70%, a dark brown solid, mp: 87–88 °C); ^1^H NMR (400 MHz, CDCl_3_): δ 9.78 (bs, NH, 1H), 9.51 (s, CHO, 1H), 8.01 (d, *J* = 3.0 Hz, CH, 1H), 7.42 (d, *J* = 8.0 Hz, CH, 1H), 7.38–7.28 (m, CH, 4H), 7.22 (d, *J* = 7.0 Hz, CH, 1H), 7.18 (d, *J* = 6.9 Hz, CH, 1H), 2.40 (s, CH_3_, 3H). ^13^C{^1^H} NMR (100 MHz, CDCl_3_): δ 187.4, 138.3, 137.0, 135.7, 131.3, 129.7, 128.5, 128.4, 126.0, 123.8, 123.4, 119.2, 111.3, 21.5. HRMS (ESI-TOF) m/z: [M + H]^+^ calcd for C_16_H_14_NO: 236.1070; found: 236.1070.

##### 4-(4-(*tert*-Butyl)phenyl)-1*H*-indole-3-carbaldehyde (**3ad**)

Compound **3ad** was synthesized by following general procedure D using 1*H*-indole-3-carbaldehyde (**1a**, 58 mg, 0.4 mmol) and 1-(*tert*-butyl)-4-iodobenzene (**2d**, 141 μL, 0.8 mmol) at 100 °C for 3 h and purified by silica gel column chromatography (80:20 hexane/ethyl acetate): **3ad** (86 mg, 78%, a dark brown solid, mp: 153–154 °C); ^1^H NMR (400 MHz, CDCl_3_): δ 9.79 (bs, NH, 1H), 9.56 (s, CHO, 1H), 8.00 (d, *J* = 3.0 Hz, CH, 1H), 7.52–7.44 (m, CH, 4H), 7.41 (d, *J* = 8.0 Hz, CH, 1H), 7.30 (t, *J* = 7.2 Hz, CH, 1H), 7.18 (d, *J* = 7.2 Hz, CH, 1H), 1.37 (s, CH_3_, 9H). ^13^C{^1^H} NMR (100 MHz, CDCl_3_): δ 187.5, 150.7, 144.6, 138.8, 137.0, 135.6, 131.2, 128.6, 125.5, 124.3, 124.0, 123.4, 119.3, 117.7, 111.1, 34.6, 31.4. HRMS (ESI-TOF) *m*/*z*: [M + H]^+^ calcd for C_19_H_20_NO: 278.1539; found: 278.1541.

##### 4-(4-Bromophenyl)-1*H*-indole-3-carbaldehyde (**3ae**)

Compound **3ae** was synthesized by following general procedure D using 1*H*-indole-3-carbaldehyde (**1a**, 58 mg, 0.4 mmol) and 1-bromo-4-iodobenzene (**2e**, 226 mg, 0.8 mmol) at 100 °C for 6 h and purified by silica gel column chromatography (80:20 hexane/ethyl acetate): **3ae** (102 mg, 86%, a dark brown solid, mp: >300 °C); ^1^H NMR (400 MHz, CDCl_3_): δ 9.55 (bs, NH, 1H), 9.04 (s, CHO, 1H), 8.03 (d, *J* = 2.7 Hz, CH, 1H), 7.64–7.56 (m, AA′ part of AA′BB′ system, CH, 2H), 7.51–7.44 (m, CH, 1H), 7.42–7.37 (m, BB′ part of AA′BB′ system, CH, 2H), 7.34 (t, *J* = 7.5 Hz, CH, 1H), 7.16 (d, *J* = 7.5 Hz, CH, 1H). ^13^C{^1^H} NMR (100 MHz, CDCl_3_): δ 186.2, 140.8, 137.0, 134.4, 131.7, 131.6, 130.7, 123.9, 123.7, 123.6, 121.9, 119.4, 111.5. HRMS (ESI-TOF) m/z: [M + H]^+^ calcd for C_15_H_11_BrNO: 300.0019; found: 300.0018.

##### 4-(4-Methoxyphenyl)-1*H*-indole-3-carbaldehyde (**3af**)

Compound **3af** was synthesized by following general procedure D using 1*H*-indole-3-carbaldehyde (**1a**, 58 mg, 0.4 mmol) and 1-iodo-4-methoxybenzene (**2f**, 187 mg, 0.8 mmol) at 100 °C for 3 h and purified by silica gel column chromatography (80:20 hexane/ethyl acetate): **3af** (88 mg, 79%, a dark brown solid, mp: 111–112 °C); ^1^H NMR (400 MHz, CDCl_3_): δ 9.55 (bs, NH, 1H), 9.21 (s, CHO, 1H), 8.02 (d, *J* = 3.2 Hz, CH, 1H), 7.48–7.40 (m, CH, 3H), 7.32 (t, *J* = 7.3 Hz, CH, 1H), 7.19–7.14 (m, CH, 1H), 7.03–6.97 (m, BB′ part of AA′BB′ system, CH, 2H), 3.87 (s, CH_3_, 3H). ^13^C{^1^H} NMR (100 MHz, CDCl_3_): δ 187.2, 159.2, 137.0, 135.4, 134.2, 131.2, 130.1, 124.4, 123.9, 123.4, 119.4, 114.0, 111.0, 55.3. HRMS (ESI-TOF) m/z: [M + H]^+^ calcd for C_16_H_14_NO_2_: 252.1019; found: 252.1019.

##### Methyl 4-(3-Formyl-1*H*-indol-4-yl)benzoate (**3ag**)

Compound **3ag** was synthesized by following general procedure D using 1*H*-indole-3-carbaldehyde (**1a**, 58 mg, 0.4 mmol) and methyl 4-iodobenzoate (**2g**, 210 mg, 0.8 mmol) at 100 °C for 12 h and purified by silica gel column chromatography (80:20 hexane/ethyl acetate): **3ag** (97 mg, 87%, a brown solid, mp: 190–191 °C); ^1^H NMR (400 MHz, Acetone-*d*_6_): δ 11.46 (bs, NH, 1H), 9.56 (s, CHO, 1H), 8.20 (d, *J* = 3.3 Hz, CH, 1H), 8.15–8.06 (m, AA′ part of AA′BB′ system, CH, 2H), 7.70–7.58 (m, CH, 3H), 7.36 (t, *J* = 7.4 Hz, CH, 1H), 7.17 (dd, *J* = 7.5, 0.8 Hz, CH, 1H), 3.92 (s,CH_3_, 3H). ^13^C{^1^H} NMR (100 MHz, Acetone-*d*_6_): δ 184.6, 167.2, 148.2, 138.8, 135.2, 134.7, 130.2 (2C), 130.0, 124.2, 124.1, 124.0, 119.9, 113.1, 52.4. HRMS (ESI-TOF) m/z: [M + H]^+^ calcd for C_17_H_14_NO_3_: 280.0968; found: 280.0968.

##### 4-(4-Acetylphenyl)-1*H*-indole-3-carbaldehyde (**3ah**)

Compound **3ah** was synthesized by following general procedure D using 1*H*-indole-3-carbaldehyde (**1a**, 58 mg, 0.4 mmol) and 1-(4-iodophenyl)ethan-1-one (**2h**, 196 mg, 0.8 mmol) at 100 °C for 3 h and purified by silica gel column chromatography (80:20 hexane/ethyl acetate): **3ah** (96 mg, 92%, a dirty white solid, mp: 220–221 °C); ^1^H NMR (400 MHz, Acetone-*d*_6_): δ 11.45 (bs, NH, 1H), 9.56 (s, CHO, 1H), 8.19 (d, *J* = 3.2 Hz, CH, 1H), 8.14–8.03 (m, AA′ part of AA′BB′ system, CH, 2H), 7.70–7.60 (m, CH, 3H), 7.36 (t, *J* = 7.4 Hz, CH, 1H), 7.19–7.14 (m, CH, 1H), 2.65 (s, CH_3_, 3H). ^13^C{^1^H} NMR (100 MHz, Acetone-*d*_6_): δ 197.7, 184.7, 148.2, 138.8, 137.1, 135.2, 134.5, 130.2, 129.1, 124.2, 124.1, 124.0, 119.8, 113.1, 26.8. HRMS (ESI-TOF) m/z: [M + H]^+^ calcd for C_17_H_14_NO_2_: 264.1019; found: 264.1024.

##### 4-(4-(Trifluoromethyl)phenyl)-1*H*-indole-3-carbaldehyde (**3ai**)

Compound **3ai** was synthesized by following general procedure D using 1*H*-indole-3-carbaldehyde (**1a**, 58 mg, 0.4 mmol) and 1-iodo-4-(trifluoromethyl)benzene (**2i**, 117 μL, 0.8 mmol) at 100 °C for 15 h and purified by silica gel column chromatography (70:30 hexane/ethyl acetate): **3ai** (95 mg, 83%, a dark brown solid, mp: 190–191 °C); ^1^H NMR (400 MHz, CDCl_3_): δ 9.53 (bs, NH, 1H), 9.03 (s, CHO, 1H), 8.04 (d, *J* = 3.1 Hz, CH, 1H), 7.79–7.70 (m, AA′ part of AA′BB′ system, CH, 2H), 7.66–7.62 (m, BB′ part of AA′BB′ system, CH, 2H), 7.50 (d, *J* = 7.6 Hz, CH, 1H), 7.37 (t, *J* = 7.6 Hz, CH, 1H), 7.19 (d, *J* = 7.6 Hz, CH, 1H). ^13^C{^1^H} NMR (100 MHz, CDCl_3_): δ 185.9, 145.6, 137.1, 134.2, 132.4, 129.8 (q, *J* = 32.6 Hz), 129.4, 126.9 (q, *J* = 272.2 Hz), 125.4 (q, *J* = 3.4 Hz), 124.1, 123.6, 123.5, 119.2, 111.9. HRMS (ESI-TOF) m/z: [M + H]^+^ calcd for C_16_H_11_F_3_NO: 290.0787; found: 290.0792.

##### 4-(3-Nitrophenyl)-1*H*-indole-3-carbaldehyde (**3al**)

Compound **3al** was synthesized by following general procedure D using 1*H*-indole-3-carbaldehyde (**1a**, 58 mg, 0.4 mmol) and 1-iodo-3-nitrobenzene (**2l**, 199 mg, 0.8 mmol) at 120 °C for 16 h and purified by silica gel column chromatography (80:20 hexane/ethyl acetate): **3ab** (72 mg, 68%, a yellow solid, mp: 187–188 °C); ^1^H NMR (400 MHz, Acetone-*d*_6_): δ 11.54 (bs, NH, 1H), 9.65 (s, CHO, 1H), 8.30 (d, *J* = 3.3 Hz, CH, 1H), 8.29–8.24 (m, CH, 2H), 7.94–7.81 (m, CH, 1H), 7.72 (t, *J* = 7.9 Hz, CH, 1H), 7.69–7.66 (m, CH, 1H), 7.40 (t, *J* = 7.4 Hz, CH, 1H), 7.27–7.21 (m, CH, 1H). ^13^C{^1^H} NMR (100 MHz, Acetone-*d*_6_): δ 183.8, 148.7, 145.0, 139.3, 138.1, 136.2, 134.2, 129.8, 124.9, 124.8, 124.3, 123.2, 122.6, 120.1, 113.4. HRMS (ESI-TOF) m/z: [M + H]^+^ calcd for C_15_H_11_N_2_O_3_: 267.0764; found: 267.0765.

##### 7-Fluoro-4-phenyl-1*H*-indole-3-carbaldehyde (**3ba**)

Compound **3ba** was synthesized by following general procedure D using 7-fluoro-1*H*-indole-3-carbaldehyde (**1b**, 65 mg, 0.4 mmol) and iodobenzene (**2a**, 90 μL, 0.8 mmol) at 100 °C for 5 h and purified by silica gel column chromatography (70:30 hexane/ethyl acetate): **3ba** (85 mg, 90%, a purple solid, mp: 114–115 °C); ^1^H NMR (400 MHz, CDCl_3_): δ 9.48 (bs, NH, 1H), 9.21 (s, CHO, 1H), 8.03 (d, *J* = 3.0 Hz, C, 1H), 7.51–7.40 (m, CH, 5H), 7.12–7.02 (m, CH, 2H). ^13^C{^1^H} NMR (100 MHz, CDCl_3_): δ 187.1, 149.2 (d, *J* = 246.3 Hz), 141.1, 132.2, 131.5 (d, *J* = 3.8 Hz), 129.1, 128.6, 127.8, 127.3 (d, *J* = 4.3 Hz), 125.2 (d, *J* = 13.8 Hz), 124.0 (d, *J* = 6.2 Hz), 119.7 (d, *J* = 1.1 Hz), 108.1 (d, *J* = 16.0 Hz). HRMS (ESI-TOF) m/z: [M + H]^+^ calcd for C_15_H_11_FNO: 240.0819; found: 240.0820.

##### 7-Bromo-4-phenyl-1*H*-indole-3-carbaldehyde (**3ca**)

Compound **3ca** was synthesized by following general procedure D using 7-bromo-1*H*-indole-3-carbaldehyde (**1c**, 45 mg, 0.2 mmol), AgOAc (67 mg, 0.4 mmol), Pd(OAc)_2_ (5 mg, 20 μmol, 10 mol %), and iodobenzene (**2a**, 90 μL, 0.8 mmol) at 100 °C for 5 h and purified by silica gel column chromatography (80:20 hexane/ethyl acetate): **3ca** (50 mg, 84%, a dark brown solid, mp: >300 °C); ^1^H NMR (400 MHz, CDCl_3_): δ 9.46 (bs, NH, 1H), 9.15 (s, CHO, 1H), 8.07 (d, *J* = 2.9 Hz, CH, 1H), 7.58–7.37 (m, CH, 6H), 7.07 (d, *J* = 7.8 Hz, B part of AB system, CH, 1H). ^13^C{^1^H} NMR (100 MHz, CDCl_3_): δ 186.7, 140.8, 135.3, 135.2, 130.9, 128.9, 128.7, 128.0, 125.7, 125.2, 124.8, 120.5, 104.6. HRMS (ESI-TOF) m/z: [M + H]^+^ calcd for C_15_H_11_BrNO: 300.0019; found: 300.0018.

##### 2-Methyl-4-phenyl-1*H*-indole-3-carbaldehyde (**3da**)

Compound **3da** was synthesized by following general procedure D using 2-methyl-1*H*-indole-3-carbaldehyde (**1d**, 58 mg, 0.4 mmol) and iodobenzene (**2a**, 90 μL, 0.8 mmol) at 100 °C for 3.5 h and purified by silica gel column chromatography (80:20 hexane/ethyl acetate): **3da** (77 mg, 82%, a pale-brown solid, mp: 190–191 °C); ^1^H NMR (400 MHz, CDCl_3_): δ 9.54 (bs, NH, 1H), 8.66 (s, CHO, 1H), 7.54–7.49 (m, CH, 2H), 7.48–7.34 (m, CH, 3H), 7.31–7.27 (m, CH, 2H), 7.13 (dd, *J* = 7.4, 0.7 Hz, CH, 1H), 2.78 (s, CH_3_, 3H). ^13^C{^1^H} NMR (100 MHz, CDCl_3_): δ 187.9, 145.4, 142.2, 135.1, 134.8, 128.9, 128.6, 127.6, 125.7, 123.8, 122.6, 114.4, 110.4, 29.7. HRMS (ESI-TOF) m/z: [M + H]^+^ calcd for C_16_H_14_NO: 236.1070; found: 236.1070.

##### 1-Benzyl-4-phenyl-1*H*-indole-3-carbaldehyde (**3ha**)

Compound **3ha** was synthesized by following general procedure D using 1-benzyl-1*H*-indole-3-carbaldehyde (**1h**, 47 mg, 0.2 mmol), AgOAc (67 mg, 0.4 mmol), Pd(OAc)_2_ (5 mg, 20 μmol, 10 mol %), and iodobenzene (**2a**, 45 μL, 0.4 mmol) at 100 °C for 3 h and purified by silica gel column chromatography (80:20 hexane/ethyl acetate): **3ha** (52 mg, 84%, a pale-brown solid, mp: 171–172 °C); ^1^H NMR (400 MHz, CDCl_3_): δ 9.45 (s, CHO, 1H), 7.96 (s, CH, 1H), 7.53 (d, *J* = 7.0 Hz, CH, 2H), 7.50–7.40 (m, CH, 3H), 7.39–7.28 (m, CH, 5H), 7.25–7.16 (m, CH, 3H), 5.38 (s, CH_2_ 2H). ^13^C{^1^H} NMR (100 MHz, CDCl_3_): δ 186.3, 141.9, 137.5, 136.0, 135.3, 134.2, 129.2, 129.1, 128.6, 128.4, 127.8, 127.4, 125.3, 123.9, 123.2, 118.4, 110.0, 51.2. HRMS (ESI-TOF) m/z: [M + H]^+^ calcd for C_22_H_18_NO: 312.1383; found: 312.1382.

##### 1-Methyl-4-phenyl-1*H*-indole-3-carbaldehyde (**3ia**)

Compound **3ia** was synthesized by following general procedure D using 1-methyl-1*H*-indole-3-carbaldehyde (**1i**, 64 mg, 0.4 mmol) and iodobenzene (**2a**, 90 μL, 0.8 mmol) at 65 °C for 3 h and purified by silica gel column chromatography (80:20 hexane/ethyl acetate): **3ia** (76 mg, 81%, a yellow oil); ^1^H NMR (400 MHz, CDCl_3_): δ 9.35 (s, CHO, 1H), 7.82 (s, CH, 1H), 7.42 (d, *J* = 7.4 Hz, CH, 2H), 7.37 (t, *J* = 7.4 Hz, CH, 2H), 7.34–7.27 (m, CH, 3H), 7.11 (dd, *J* = 6.0, 2.0 Hz, CH, 1H), 3.80 (s, CH_3_, 3H). ^13^C{^1^H} NMR (100 MHz, CDCl_3_): δ 186.2, 141.9, 137.9, 135.8, 134.8, 129.0, 128.6, 127.7, 125.0, 123.8, 123.0, 118.0, 109.4, 33.9. HRMS (ESI-TOF) m/z: [M + H]^+^ calcd for C_16_H_14_NO: 236.1070; found: 236.1070.

##### 1-(4-Phenyl-1*H*-indol-2-yl)ethan-1-one (**5aa**)

Compound **5aa** was synthesized by following general procedure D using 1-(1*H*-indol-3-yl)ethan-1-one (**4a**, 64 mg, 0.4 mmol) and iodobenzene (**2a**, 90 μL, 0.8 mmol) at 120 °C for 7 h and purified by silica gel column chromatography (90:10 hexane/ethyl acetate): **5aa** (78 mg, 83%, a yellow solid, mp: 188–189 °C); ^1^H NMR (400 MHz, CDCl_3_): δ 9.14 (bs, NH, 1H), 7.69–7.65 (m, CH, 2H), 7.53 (t, *J* = 7.6 Hz, CH, 2H), 7.46–7.39 (m, CH, 3H), 7.34 (d, *J* = 1.8 Hz, CH, 1H), 7.22 (dd, *J* = 5.9, 2.2 Hz, CH, 1H), 2.58 (s, CH_3_, 3H). ^13^C{^1^H} NMR (100 MHz, CDCl_3_): δ 190.8, 140.4, 138.0, 137.0, 135.6, 128.8, 128.7, 127.5, 126.6, 126.2, 120.6, 111.4, 109.7, 25.9. HRMS (ESI-TOF) m/z: [M + H]^+^ calcd for C_16_H_14_NO: 236.1070; found: 236.1070.

##### 1-(4-(*p*-Tolyl)-1*H*-indol-2-yl)ethan-1-one (**5ab**)

Compound **5ab** was synthesized by following general procedure D using 1-(1*H*-indol-3-yl)ethan-1-one (**4a**, 64 mg, 0.4 mmol) and 1-iodo-4-methylbenzene (**2b**, 175, 0.8 mmol) at 130 °C for 7 h and purified by silica gel column chromatography (90:10 hexane/ethyl acetate): **5ab** (81 mg, 81%, a dirty white solid, mp: 185–186 °C); ^1^H NMR (400 MHz, CDCl_3_): δ 9.46 (bs, NH, 1H), 7.62–7.56 (m, AA′ part of AA′BB′ system, CH, 2H), 7.45–7.39 (m, BB′ part of AA′BB′ system, 2H), 7.38–7.32 (m, CH, 3H), 7.20 (t, *J* = 4.1 Hz, CH, 1H), 2.59 (s, CH_3_, 3H), 2.47 (s, CH_3_, 3H). ^13^C{^1^H} NMR (100 MHz, CDCl_3_): δ 190.6, 138.0, 137.5, 137.3, 137.0, 135.5, 129.5, 128.6, 126.7, 126.2, 120.5, 111.1, 109.7, 25.9, 21.3. HRMS (ESI-TOF) m/z: [M + H]^+^ calcd for C_17_H_16_NO: 250.1226; found: 250.1226.

##### 1-(4-(*m*-Tolyl)-1*H*-indol-2-yl)ethan-1-one (**5ac**)

Compound **5ac** was synthesized by following general procedure D using 1-(1*H*-indol-3-yl)ethan-1-one (**4a**, 64 mg, 0.4 mmol) and 1-iodo-3-methylbenzene (**2c**, 103 μL, 0.8 mmol) at 120 °C for 7 h and purified by silica gel column chromatography (90:10 hexane/ethyl acetate): **5ac** (74 mg, 74%, a pale-yellow solid, mp: 110–111 °C); ^1^H NMR (400 MHz, CDCl_3_): δ 9.59 (bs, NH, 1H), 7.51–7.47 (m, CH, 2H), 7.45–7.38 (m, CH, 3H), 7.35 (d, *J* = 1.9 Hz, CH, 1H), 7.25 (d, *J* = 7.4 Hz, CH, 1H), 7.20 (dd, *J* = 6.3, 1.9 Hz, CH, 1H), 2.60 (s, CH_3_, 3H), 2.47 (s, CH_3_, 3H). ^13^C{^1^H} NMR (100 MHz, CDCl_3_): δ 190.7, 140.4, 138.4, 138.0, 137.2, 135.6, 129.5, 128.6, 128.3, 126.6, 126.2, 125.9, 120.6, 111.3, 109.7, 26.0, 21.6. HRMS (ESI-TOF) m/z: [M + H]^+^ calcd for C_17_H_16_NO: 250.1226; found: 250.1226.

##### 1-(4-(4-(*tert*-Butyl)phenyl)-1*H*-indol-2-yl)ethan-1-one (**5ad**)

Compound **5ad** was synthesized by following general procedure D using 1-(1*H*-indol-3-yl)ethan-1-one (**4a**, 64 mg, 0.4 mmol) and 1-(*tert*-butyl)-4-iodobenzene (**2d**, 142 μL, 0.8 mmol) at 120 °C for 8 h and purified by silica gel column chromatography (90:10 hexane/ethyl acetate): **5ad** (89 mg, 76%, a brown solid, mp: 209-210 °C); ^1^H NMR (400 MHz, CDCl_3_): δ 9.38 (bs, NH, 1H), 7.67–7.60 (m, AA′ part of AA′BB′ system, CH, 2H), 7.59–7.54 (m, BB′ part of AA′BB′ system, 2H), 7.45–7.39 (m, CH, 3H), 7.22 (dd, *J* = 4.6, 3.5 Hz, CH, 1H), 2.60 (s, CH_3_, 3H), 1.43 (s, CH_3_, 9H). ^13^C{^1^H} NMR (100 MHz, CDCl_3_): δ 190.6, 150.5, 137.9, 137.4, 136.9, 135.5, 128.4, 126.7, 126.2, 125.7, 120.5, 111.0, 109.8, 34.7, 31.4, 25.9. HRMS (ESI-TOF) m/z: [M + H]^+^ calcd for C_20_H_22_NO: 292.1696; found: 292.1696.

##### 1-(4-(4-Bromophenyl)-1*H*-indol-2-yl)ethan-1-one (**5ae**)

Compound **5ae** was synthesized by following general procedure D using 1-(1*H*-indol-3-yl)ethan-1-one (**4a**, 64 mg, 0.4 mmol) and 1-bromo-4-iodobenzene (**2e**, 227 mg, 0.8 mmol) at 100 °C for 13 h and purified by silica gel column chromatography (90:10 hexane/ethyl acetate): **5ae** (101 mg, 80%, a white solid, mp: 198–199 °C); ^1^H NMR (400 MHz, CDCl_3_): δ 9.14 (bs, NH, 1H), 7.68–7.61 (m, AA′ part of AA′BB′ system, CH, 2H), 7.56–7.51 (m, BB′ part of AA′BB′ system, CH, 2H), 7.45–7.40 (m, CH, 2H), 7.27 (d, *J* = 1.9 Hz, CH, 1H), 7.18 (dd, *J* = 4.7, 3.4 Hz, CH, 1H), 2.58 (s, CH_3_, 3H). ^13^C{^1^H} NMR (100 MHz, CDCl_3_): δ 190.5, 139.3, 137.8, 135.8, 135.7, 131.9, 130.3, 126.6, 125.9, 121.7, 120.6, 111.7, 109.0, 25.9. HRMS (ESI-TOF) m/z: [M + H]^+^ calcd for C_16_H_13_BrNO: 314.0175; found: 314.0174.

##### 1-(4-(4-Methoxyphenyl)-1*H*-indol-2-yl)ethan-1-one (**5af**)

Compound **5af** was synthesized by following general procedure D using 1-(1*H*-indol-3-yl)ethan-1-one (**4a**, 64 mg, 0.4 mmol) and 1-iodo-4-methoxybenzene (**2f**, 188 mg, 0.8 mmol) at 120 °C for 5 h and purified by silica gel column chromatography (90:10 hexane/ethyl acetate): **5af** (74 mg, 70%, a pale-yellow solid, mp: 196–197 °C); ^1^H NMR (400 MHz, CDCl_3_): δ 9.34 (bs, NH, 1H), 7.66–7.56 (m, AA′ part of AA′BB′ system, CH, 2H), 7.46–7.37 (m, CH, 2H), 7.34 (d, *J* = 1.5 Hz, CH, 1H), 7.17 (dd, *J* = 5.8, 2.2 Hz, CH, 1H), 7.14–7.03 (m, CH, BB′ part of AA′BB′ system, 2H), 3.90 (s, CH_3_, 3H), 2.59 (s, CH_3_, 3H). ^13^C{^1^H} NMR (100 MHz, CDCl_3_): δ 190.6, 159.2, 138.0, 136.7, 135.5, 132.9, 129.8, 126.7, 126.2, 120.3, 114.2, 110.8, 109.7, 55.4, 25.9. HRMS (ESI-TOF) m/z: [M + H]^+^ calcd for C_17_H_16_NO_2_: 266.1176; found: 266.1175.

##### Methyl 4-(2-Acetyl-1*H*-indol-4-yl)benzoate (**5ag**)

Compound **5ag** was synthesized by following general procedure D using 1-(1*H*-indol-3-yl)ethan-1-one (**4a**, 64 mg, 0.4 mmol) and methyl 4-iodobenzoate (**2g**, 210 mg, 0.8 mmol) at 110 °C for 15 h and purified by silica gel column chromatography (90:10 hexane/ethyl acetate): **5ag** (82 mg, 70%, a white solid, mp: 190–191 °C); ^1^H NMR (400 MHz, CDCl_3_): δ 9.53 (bs, NH, 1H), 8.42–7.96 (m, AA′ part of AA′BB′ system, CH, 2H), 7.95–7.67 (m, BB′ part of AA′BB′ system, CH, 2H), 7.52–7.40 (m, CH, 2H), 7.32 (s, CH, 1H), 7.24 (d, *J* = 6.8 Hz, CH, 1H), 3.98 (s, CH_3_, 3H), 2.60 (s, CH_3_, 3H). ^13^C{^1^H} NMR (100 MHz, CDCl_3_): δ 190.6, 167.0, 145.0, 137.9, 135.8, 135.8, 130.0, 129.1, 128.7, 126.6, 126.0, 120.9, 112.1, 109.0, 52.2, 25.9. HRMS (ESI-TOF) m/z: [M + H]^+^ calcd for C_18_H_16_NO_3_: 294.1125; found: 294.1124.

##### 1-(4-(2-Acetyl-1*H*-indol-4-yl)phenyl)ethan-1-one (**5ah**)

Compound **5ah** was synthesized by following general procedure D using 1-(1*H*-indol-3-yl)ethan-1-one (**4a**, 64 mg, 0.4 mmol) and 1-(4-iodophenyl)ethan-1-one (**2h**, 198 mg, 0.8 mmol) at 120 °C for 7 h and purified by silica gel column chromatography (90:10 hexane/ethyl acetate): **5ah** (59 mg, 53%, a white solid, mp: 223–224 °C); ^1^H NMR (400 MHz, CDCl_3_): δ 9.53 (bs, NH, 1H), 8.42–7.96 (m, AA′ part of AA′BB′ system, CH, 2H), 7.95–7.67 (m, BB′ part of AA′BB′ system, CH, 2H), 7.52–7.40 (m, CH, 2H), 7.32 (s, CH, 1H), 7.24 (d, *J* = 6.8 Hz, CH, 1H), 3.98 (s, CH_3_, 3H), 2.60 (s, CH_3_, 3H). ^13^C{^1^H} NMR (100 MHz, CDCl_3_): δ 190.6, 167.0, 145.0, 137.9, 135.8, 135.8, 130.0, 129.1, 128.7, 126.6, 126.0, 120.9, 112.1, 109.0, 52.2, 25.9. HRMS (ESI-TOF) m/z: [M + H]^+^ calcd for C_18_H_16_NO_2_: 278.1176; found: 278.1182.

##### 1-(4-(4-(Trifluoromethyl)phenyl)-1*H*-indol-2-yl)ethan-1-one (**5ai**)

Compound **5aa** was synthesized by following general procedure D using 1-(1*H*-indol-3-yl)ethan-1-one (**4a**, 64 mg, 0.4 mmol) and 1-iodo-4-(trifluoromethyl)benzene (**2i**, 118 μL, 0.8 mmol) at 110 °C for 20 h and purified by silica gel column chromatography (90:10 hexane/ethyl acetate): **5ai** (83 mg, 68%, a white solid, mp: 216-217 °C); ^1^H NMR (400 MHz, CDCl_3_): δ 9.56 (bs, NH, 1H), 7.82–7.75 (m, CH, 4H), 7.50 (d, *J* = 7.5 Hz, CH, 1H), 7.45 (t, *J* = 7.5 Hz, CH, 1H), 7.30 (d, *J* = 1.1 Hz, CH, 1H), 7.23 (d, *J* = 7.5 Hz, CH, 1H), 2.61 (s, CH_3_, 3H). ^13^C{^1^H} NMR (100 MHz, CDCl_3_): δ 190.6, 144.0, 137.9, 135.9, 135.4, 129.5 (q, *J* = 32.4 Hz), 129.0, 126.6, 126.0, 125.7 (q, *J* = 3.8 Hz), 121.6 (q, *J* = 272.4 Hz), 120.9, 112.2, 108.8, 25.9. HRMS (ESI-TOF) m/z: [M + H]^+^ calcd for C_17_H_13_F_3_NO: 304.0944; found: 304.0944.

##### 1-(4-(3,4-Dimethylphenyl)-1*H*-indol-2-yl)ethan-1-one (**5ak**)

Compound **5aa** was synthesized by following general procedure D using 1-(1*H*-indol-3-yl)ethan-1-one (**4a**, 64 mg, 0.4 mmol) and 4-iodo-1,2-dimethylbenzene (**2k**, 114 μL, 0.8 mmol) at 120 °C for 6 h and purified by silica gel column chromatography (90:10 hexane/ethyl acetate): **5ak** (77 mg, 73%, a brown solid, mp: 180–181 °C); ^1^H NMR (400 MHz, CDCl_3_): δ 9.41 (bs, NH, 1H), 7.47–7.40 (m, CH, 4H), 7.37 (d, *J* = 2.0 Hz, CH, 1H), 7.31 (d, *J* = 7.6 Hz, CH, 1H), 7.23–7.18 (m, CH, 1H), 2.60 (s, CH_3_, 3H), 2.40 (s, CH_3_, 3H), 2.38 (s, CH_3_, 3H). ^13^C{^1^H} NMR (100 MHz, CDCl_3_): δ 190.6, 138.0, 137.9, 137.2, 137.0, 136.0, 135.5, 129.98, 129.96, 126.6, 126.2, 126.2, 120.4, 110.9, 109.7, 25.9, 20.0, 19.6. HRMS (ESI-TOF) m/z: [M + H]^+^ calcd for C_18_H_18_NO: 264.1383; found: 264.1382.

##### 1-(4-(3-Nitrophenyl)-1*H*-indol-2-yl)ethan-1-one (**5al**)

Compound **5al** was synthesized by following general procedure D using 1-(1*H*-indol-3-yl)ethan-1-one (**4a**, 64 mg, 0.4 mmol) and 1-iodo-3-nitrobenzene (**2l**, 200 mg, 0.8 mmol) at 100 °C for 15 h and purified by silica gel column chromatography (90:10 hexane/ethyl acetate): **5al** (59 mg, 53%, a yellow solid, mp: 210–211 °C); ^1^H NMR (400 MHz, DMSO-*d*_6_): δ 12.02 (bs, NH, 1H), 8.42 (s, CH, 1H), 8.28 (d, *J* = 7.8 Hz, CH, 1H), 8.16 (d, *J* = 7.8 Hz, CH, 1H), 7.83 (t, *J* = 7.8 Hz, CH, 1H), 7.54 (d, *J* = 7.7 Hz, CH, 1H), 7.48 (s, CH, 1H), 7.42 (t, *J* = 7.7 Hz, CH, 1H), 7.28 (d, *J* = 7.7 Hz, CH, 1H), 2.56 (s, CH_3_, 3H). ^13^C{^1^H} NMR (100 MHz, DMSO-*d*_6_): δ 190.3, 148.3, 141.4, 138.2, 136.5, 134.8, 133.1, 130.4, 125.7, 124.8, 122.7, 122.1, 120.3, 113.1, 107.7, 26.2. HRMS (ESI-TOF) m/z: [M + H]^+^ calcd for C_16_H_13_N_2_O_3_281.0921; found: 281.0920.

##### 1-(7-Fluoro-4-phenyl-1*H*-indol-2-yl)ethan-1-one (**5ba**)

Compound **5ba** was synthesized by following general procedure D using 1-(7-fluoro-1*H*-indol-3-yl)ethan-1-one (**4b**, 71 mg, 0.4 mmol) and iodobenzene (**2a**, 90 μL, 0.8 mmol) at 120 °C for 18 h and purified by silica gel column chromatography (90:10 hexane/ethyl acetate): **5ba** (74 mg, 73%, an orange solid, mp: 164–165 °C); ^1^H NMR (400 MHz, CDCl_3_): δ 9.31 (bs, NH, 1H), 7.62 (d, *J* = 7.2 Hz, CH, 2H), 7.52 (t, *J* = 7.6 Hz, CH, 2H), 7.46–7.40 (m, CH, 1H), 7.34–7.31 (m, CH, 1H), 7.14–7.10 (m, CH, 2H), 2.59 (s, CH_3_, 3H). ^13^C{^1^H} NMR (100 MHz, CDCl_3_): δ 190.2, 149.2 (d, *J* = 247.0 Hz), 139.7, 136.2, 133.0 (d, *J* = 4.0 Hz), 129.2 (d, *J* = 5.2 Hz), 128.8, 128.7, 127.5, 126.4 (d, *J* = 14.8 Hz), 120.6 (d, *J* = 5.6 Hz), 110.8 (d, *J* = 16.0 Hz), 109.7, 26.0. HRMS (ESI-TOF) m/z: [M + H]^+^ calcd for C_16_H_13_FNO: 254.0976; found: 254.0976.

##### 1-(7-Bromo-4-phenyl-1*H*-indol-2-yl)ethan-1-one (**5ca**)

Compound **5ca** was synthesized by following general procedure D using 1-(7-bromo-1*H*-indol-3-yl)ethan-1-one (**4c**, 71 mg, 0.3 mmol), AgOAc (100 mg, 0.6 mmol), Pd(OAc)_2_ (7 mg, 30 μmol, 10 mol %), and iodobenzene (**2a**, 60 μL, 0.6 mmol) at 120 °C for 24 h and purified by silica gel column chromatography (85:15 hexane/ethyl acetate): **5ca** (69 mg, 74%, a pale-brown solid, mp: 125–126 °C); ^1^H NMR (400 MHz, CDCl_3_): δ 9.21 (bs, NH, 1H), 7.64 (d, *J* = 8.0 Hz, CH, 2H), 7.59–7.50 (m, CH, 3H), 7.48–7.42 (m, CH, 1H), 7.38 (d, *J* = 1.7 Hz, CH, 1H), 7.14–7.08 (m, CH, 1H), 2.59 (s, CH_3_, 3H). ^13^C{^1^H} NMR (100 MHz, CDCl_3_): δ 190.2, 139.5, 136.4, 136.4, 135.9, 128.9, 128.7, 128.7, 127.8, 127.0, 121.7, 110.1, 104.5, 25.9. HRMS (ESI-TOF) m/z: [M + H]^+^ calcd for C_16_H_13_BrNO: 314.0175; found: 314.0175.

##### 1-(7-Bromo-4-phenyl-1*H*-indol-3-yl)ethan-1-one (**6ca**)

Compound **6ca** was synthesized by following general procedure D using 1-(7-bromo-1*H*-indol-3-yl)ethan-1-one (**4c**, 71 mg, 0.3 mmol), AgOAc (100 mg, 0.6 mmol), Pd(OAc)_2_ (7 mg, 30 μmol, 10 mol %), and iodobenzene (**2a**, 60 μL, 0.6 mmol) at 120 °C for 20 h and purified by silica gel column chromatography (90:10 hexane/ethyl acetate): **6ca** (71 mg, 76%, a brown solid, mp: 162–163 °C); ^1^H NMR (400 MHz, CDCl_3_): δ 9.01 (bs, NH, 1H), 7.79 (d, *J* = 2.9 Hz, CH, 1H), 7.49 (d, *J* = 7.9 Hz, A part of AB system, CH, 1H), 7.45–7.33 (m, CH, 5H), 7.09 (d, *J* = 7.9 Hz, B part of AB system, CH, 1H), 2.07 (s, CH_3,_ 3H). ^13^C{^1^H} NMR (100 MHz, CDCl_3_): δ 194.2, 142.0, 136.0, 135.8, 131.2, 128.6, 128.1, 127.2, 125.9, 125.1, 123.6, 121.9, 104.1, 29.1. HRMS (ESI-TOF) m/z: [M + H]^+^ calcd for C_16_H_13_BrNO: 314.0175; found: 314.0175.

##### 2-Methyl-4-phenyl-1*H*-indole (**7da**)

Compound **7da** was synthesized by following general procedure D using 1-(2-methyl-1*H*-indol-3-yl)ethan-1-one (**4d**, 69 mg, 0.4 mmol) and iodobenzene (**2a**, 90 μL, 0.8 mmol) at 120 °C for 13 h and purified by silica gel column chromatography (90:10 hexane/ethyl acetate): **7da** (66 mg, 80%, a brown solid, mp: 96–97 °C); ^1^H NMR (400 MHz, CDCl_3_): δ 7.78 (bs, NH, 1H), 7.64–7.57 (m, CH, 2H), 7.38 (t, *J* = 7.6 Hz, CH, 2H), 7.30–7.23 (m, CH, 1H), 7.18–7.03 (m, CH, 3H), 6.32 (s, CH, 1H), 2.32 (s, CH_3_, 3H). ^13^C{^1^H} NMR (100 MHz, CDCl_3_): δ 141.5, 136.5, 135.5, 133.4, 128.8, 128.4, 127.3, 126.8, 121.3, 119.6, 109.5, 100.0, 13.8. HRMS (ESI-TOF) m/z: [M + H]^+^ calcd for C_15_H_14_N: 208.1121; found: 208.1120.

##### 1-(1-Benzyl-4-phenyl-1*H*-indol-3-yl)ethan-1-one (**9aa**)

Compound **9aa** was synthesized by following general procedure D using 1-(1-benzyl-1*H*-indol-3-yl)ethan-1-one (**8a**, 50 mg, 0.2 mmol), AgOAc (67 mg, 0.4 mmol), Pd(OAc)_2_ (5 mg, 20 μmol, 10 mol %), and iodobenzene (**2a**, 45 μL, 0.4 mmol) at 100 °C for 5 h and purified by silica gel column chromatography (90:10 hexane/ethyl acetate): **9aa** (58 mg, 89%, a white solid, mp: 132–133 °C); ^1^H NMR (400 MHz, CDCl_3_): δ 7.71 (s, CH, 1H), 7.47–7.42 (m, CH, 4H), 7.42–7.29 (m, CH, 6H), 7.24–7.18 (m, CH, 3H), 5.37 (s, CH_2_, 2H), 2.00 (s, CH_3_, 3H). ^13^C{^1^H} NMR (100 MHz, CDCl_3_): δ 194.0, 143.0, 138.0, 136.8, 135.8, 134.6, 129.1, 128.7, 128.2, 128.0, 127.1, 127.0, 124.1, 123.6, 123.3, 119.9, 109.3, 50.8, 29.1. HRMS (ESI-TOF) m/z: [M + H]^+^ calcd for C_23_H_20_NO: 326.1539; found: 326.1539.

##### 1-(1-Benzyl-4-phenyl-1*H*-indol-3-yl)ethan-1-one (**9ab**)

Compound **9ab** was synthesized by following general procedure D using 1-(1-benzyl-1*H*-indol-3-yl)ethan-1-one (**8a**, 50 mg, 0.2 mmol), AgOAc (67 mg, 0.4 mmol), Pd(OAc)_2_ (5 mg, 20 μmol, 10 mol %), and 1-iodo-4-methylbenzene (**2b**, 87 mg, 0.4 mmol) at 100 °C for 5.5 h and purified by silica gel column chromatography (85:15 hexane/ethyl acetate): **9ab** (59 mg, 87%, a white solid, mp: 125–126 °C); ^1^H NMR (400 MHz, CDCl_3_): δ 7.68 (s, CH, 1H), 7.36–7.31 (m, CH, 5H), 7.30–7.27 (m, CH, 2H), 7.24–7.16 (m, CH, 5H), 5.34 (s, CH_2_, 2H), 2.40 (s, CH_3_, 3H), 1.98 (s, CH_3_, 3H). ^13^C{^1^H} NMR (100 MHz, CDCl_3_): δ 194.3, 140.1, 138.0, 136.8, 136.6, 135.8, 134.5, 129.1, 128.8, 128.6, 128.2, 127.1, 124.1, 123.6, 123.3, 120.0, 109.1, 50.8, 29.3, 21.3. HRMS (ESI-TOF) m/z: [M + H]^+^ calcd for C_24_H_22_NO: 340.1696; found: 340.1695.

##### 1-(1-Benzyl-4-(*m*-tolyl)-1*H*-indol-3-yl)ethan-1-one (**9ac**)

Compound **9ac** was synthesized by following general procedure D using 1-(1-benzyl-1*H*-indol-3-yl)ethan-1-one (**8a**, 50 mg, 0.2 mmol), AgOAc (67 mg, 0.4 mmol), Pd(OAc)_2_ (5 mg, 20 μmol, 10 mol %), and 1-iodo-3-methylbenzene (**2c**, 51 μL, 0.4 mmol) at 100 °C for 5.5 h and purified by silica gel column chromatography (90:10 hexane/ethyl acetate): **9ac** (57 mg, 85%, a pale-brown solid, mp: 115–116 °C); ^1^H NMR (400 MHz, CDCl_3_): δ 7.60 (s, 1H), 7.29–7.19 (m, 7H), 7.17–7.08 (m, 5H), 5.27 (s, 2H), 2.31 (s, 3H), 1.88 (s, 3H). ^13^C{^1^H} NMR (100 MHz, CDCl_3_): δ 194.4, 142.8, 137.9, 137.7, 136.8, 135.8, 134.3, 129.3, 129.0, 128.2, 128.0, 127.8, 127.1, 125.8, 123.9, 123.6, 123.3, 120.0, 109.2, 50.8, 29.2, 21.5. HRMS (ESI-TOF) m/z: [M + H]^+^ calcd for C_24_H_22_NO: 340.1696; found: 340.1696.

##### 1-(1-Benzyl-4-(4-(*tert*-butyl)phenyl)-1*H*-indol-3-yl)ethan-1-one (**9ad**)

Compound **9ad** was synthesized by following general procedure D using 1-(1-benzyl-1*H*-indol-3-yl)ethan-1-one (**8a**, 50 mg, 0.2 mmol), AgOAc (67 mg, 0.4 mmol), Pd(OAc)_2_ (5 mg, 20 μmol, 10 mol %), and 1-(*tert*-butyl)-4-iodobenzene (**2d**, 71 μL, 0.4 mmol) at 100 °C for 5.5 h and purified by silica gel column chromatography (90:10 hexane/ethyl acetate): **9ad** (68 mg, 90%, a white solid, mp: 166–167 °C); ^1^H NMR (400 MHz, CDCl_3_): δ 7.66 (s, CH, 1H), 7.47–7.40 (m, CH, 4H), 7.39–7.29 (m, CH, 5H), 7.24–7.19 (m, CH, 3H), 5.35 (s, CH_2_, 2H), 1.79 (s, CH_3_, 3H), 1.38 (s, CH_3_, 9H). ^13^C{^1^H} NMR (100 MHz, CDCl_3_): δ 195.9, 150.2, 139.9, 137.9, 136.4, 135.8, 133.8, 129.0, 128.4, 128.2, 127.2, 125.3, 123.8, 123.7, 123.2, 120.5, 109.2, 50.8, 34.6, 31.4, 29.6. HRMS (ESI-TOF) m/z: [M + H]^+^ calcd for C_27_H_28_NO: 382.2165; found: 382.2165.

##### 1-(1-Benzyl-4-(4-bromophenyl)-1*H*-indol-3-yl)ethan-1-one (**9ae**)

Compound **9ae** was synthesized by following general procedure D using 1-(1-benzyl-1*H*-indol-3-yl)ethan-1-one (**8a**, 50 mg, 0.2 mmol), AgOAc (67 mg, 0.4 mmol), Pd(OAc)_2_ (5 mg, 20 μmol, 10 mol %), and 1-bromo-4-iodobenzene (**2e**, 113 mg, 0.4 mmol) at 100 °C for 6 h and purified by silica gel column chromatography (90:10 hexane/ethyl acetate): **9ae** (64 mg, 79%, a pale-brown solid, mp: 145–146 °C); ^1^H NMR (400 MHz, CDCl_3_): δ 7.75 (s, CH, 1H), 7.55–7.50 (m, AA′ part of AA′BB′ system, CH, 2H), 7.39–7.24 (m, CH, 7H), 7.20–7.13 (m, CH, 3H), 5.39 (s, CH_2_, 2H), 2.17 (s, CH_3_, 3H). ^13^C{^1^H} NMR (100 MHz, CDCl_3_): δ 192.7, 142.0, 138.1, 135.7, 135.7, 135.5, 130.9, 130.3, 129.1, 128.3, 127.0, 124.5, 123.5, 123.2, 120.9, 119.2, 109.6, 50.8, 28.8. HRMS (ESI-TOF) m/z: [M + H]^+^ calcd for C_23_H_19_BrNO: 404.0645; found: 404.0644.

##### 1-(1-Benzyl-4-(4-methoxyphenyl)-1*H*-indol-3-yl)ethan-1-one (**9af**)

Compound **9af** was synthesized by following general procedure D using 1-(1-benzyl-1*H*-indol-3-yl)ethan-1-one (**8a**, 50 mg, 0.2 mmol), AgOAc (67 mg, 0.4 mmol), Pd(OAc)_2_ (5 mg, 20 μmol, 10 mol %), and 1-iodo-4-methoxybenzene (**2f**, 113 mg, 0.4 mmol) at 100 °C for 5.5 h and purified by silica gel column chromatography (90:10 hexane/ethyl acetate): **9af** (57 mg, 80%, a brown solid, mp: 132–133 °C); ^1^H NMR (400 MHz, CDCl_3_): δ 7.70 (s, CH, 1H), 7.40–7.37 (m, AA′ part of AA′BB′ system, CH, 2H), 7.36–7.28 (m, CH, 5H), 7.22–7.16 (m, CH, 3H), 7.05–6.87 (m, BB′ part of AA′BB′ system, CH, 2H), 5.36 (s, CH_2_, 2H), 3.86 (s, CH_3_, 3H), 2.01 (s, CH_3_, 3H). ^13^C{^1^H} NMR (100 MHz, CDCl_3_): δ 194.6, 158.8, 138.0, 136.4, 135.8, 135.5, 134.4, 129.7, 129.1, 128.2, 127.1, 124.0, 123.7, 123.3, 120.1, 113.6, 109.0, 55.2, 50.8, 29.4. HRMS (ESI-TOF) m/z: [M + H]^+^ calcd for C_24_H_22_NO_2_: 356.1645; found: 356.1645.

##### Methyl 4-(3-Acetyl-1-benzyl-1*H*-indol-4-yl)benzoate (**9ag**)

Compound **9ag** was synthesized by following general procedure D using 1-(1-benzyl-1*H*-indol-3-yl)ethan-1-one (**8a**, 50 mg, 0.2 mmol), AgOAc (67 mg, 0.4 mmol), Pd(OAc)_2_ (5 mg, 20 μmol, 10 mol %), and methyl 4-iodobenzoate (**2g**, 105 mg, 0.4 mmol) at 110 °C for 10 h and purified by silica gel column chromatography (90:10 hexane/ethyl acetate): **9ag** (62 mg, 81%, a brown solid, mp: 165–166 °C); ^1^H NMR (400 MHz, CDCl_3_): δ 8.18–8.04 (m, AA′ part of AA′BB′ system, CH, 2H), 7.78 (s, CH, 1H), 7.55–7.41 (m, BB′ part of AA′BB′ system, CH, 2H), 7.42–7.27 (m, CH, 5H), 7.25–7.15 (m, CH, 3H), 5.39 (s, CH_2_, 2H), 3.93 (s, CH_3_, 3H), 2.18 (s, CH_3_, 3H). ^13^C{^1^H} NMR (100 MHz, CDCl_3_): δ 192.4, 167.2, 147.8, 138.1, 135.9, 135.7, 135.5, 129.1, 129.1, 128.7, 128.4, 128.3, 127.0, 124.5, 123.4, 123.2, 119.2, 109.9, 52.0, 50.9, 28.6. HRMS (ESI-TOF) m/z: [M + H]^+^ calcd for C_25_H_22_NO_3_: 384.1594; found: 384.1595.

##### 1-(4-(3-Acetyl-1-benzyl-1*H*-indol-4-yl)phenyl)ethan-1-one (**9ah**)

Compound **9ah** was synthesized by following general procedure D using 1-(1-benzyl-1*H*-indol-3-yl)ethan-1-one (**8a**, 50 mg, 0.2 mmol), AgOAc (98 mg, 0.4 mmol), Pd(OAc)_2_ (5 mg, 20 μmol, 10 mol %), and 1-(4-iodophenyl)ethan-1-one (**2h**, 87 mg, 0.4 mmol) at 120 °C for 6 h and purified by silica gel column chromatography (90:10 hexane/ethyl acetate): **9ah** (58 mg, 80%, a brown solid, mp: 92–93 °C); ^1^H NMR (400 MHz, CDCl_3_): δ 8.05–7.97 (m, AA′ part of AA′BB′ system, CH, 2H), 7.85 (s, CH, 1H), 7.53–7.41 (m, BB′ part of AA′BB′ system, CH, 2H), 7.41–7.30 (m, CH, 5H), 7.24–7.14 (m, CH, 3H), 5.41 (s, CH_2_, 2H), 2.65 (s, CH_3_, 3H), 2.25 (s, CH_3_, 3H). ^13^C{^1^H} NMR (100 MHz, CDCl_3_): δ 198.1, 192.5, 148.1, 138.2, 136.0, 135.8, 135.6, 135.3, 129.1(2C), 128.8, 128.3, 127.9, 127.0, 124.7, 123.5, 123.1, 118.9, 110.1, 50.9, 26.6. HRMS (ESI-TOF) m/z: [M + H]^+^ calcd for C_25_H_22_NO_2_: 368.1645; found: 368.1666.

##### 1-(1-Benzyl-4-(4-(trifluoromethyl)phenyl)-1*H*-indol-3-yl)ethan-1-one (**9ai**)

Compound **9ai** was synthesized by following general procedure D using 1-(1-benzyl-1*H*-indol-3-yl)ethan-1-one (**8a**, 50 mg, 0.2 mmol), AgOAc (67 mg, 0.4 mmol), Pd(OAc)_2_ (5 mg, 20 μmol, 10 mol %), and 1-iodo-4-(trifluoromethyl)benzene (**2i**, 59 μL, 0.4 mmol) at 100 °C for 5.5 h and purified by silica gel column chromatography (90:10 hexane/ethyl acetate): **9ai** (60 mg, 77%, a white solid, mp: 169–170 °C); ^1^H NMR (400 MHz, CDCl_3_): δ 7.80 (s, CH, 1H), 7.70–7.63 (m, AA′ part of AA′BB′ system, CH, 2H), 7.55–7.46 (m, BB′ part of AA′BB′ system, CH, 2H), 7.42–7.30 (m, CH, 5H), 7.25–7.15 (m, CH, 3H), 5.41 (s, CH_2_, 2H), 2.21 (s, CH_3_, 3H). ^13^C{^1^H} NMR (100 MHz, CDCl_3_): δ 192.1, 146.7, 138.1, 135.7(2C), 135.6, 129.1, 128.9, 128.7 (q, *J* = 32.4 Hz), 128.3, 127.0, 124.7, 124.6 (q, *J* = 3.4 Hz), 124.3 (q, *J* = 272.4 Hz), 123.5, 123.2, 119.0, 110.0, 50.9, 28.5. HRMS (ESI-TOF) m/z: [M + H]^+^ calcd for C_24_H_19_F_3_NO: 394.1413; found: 394.1413.

##### 1-(1-Benzyl-4-(3,4-dimethylphenyl)-1*H*-indol-3-yl)ethan-1-one (**9ak**)

Compound **9ak** was synthesized by following general procedure D using 1-(1-benzyl-1*H*-indol-3-yl)ethan-1-one (**8a**, 50 mg, 0.2 mmol), AgOAc (67 mg, 0.4 mmol), Pd(OAc)_2_ (5 mg, 20 μmol, 10 mol %), and 4-iodo-1,2-dimethylbenzene (**2k**, 57 μL, 0.4 mmol) at 100 °C for 4 h and purified by silica gel column chromatography (90:10 hexane/ethyl acetate): **9ak** (49 mg, 70%, a yellow gum); ^1^H NMR (400 MHz, CDCl_3_): δ 7.65 (s, CH, 1H), 7.34–7.27 (m, CH, 3H), 7.26–7.24 (m, CH, 1H), 7.23–7.19 (m, CH, 2H), 7.18–7.11 (m, CH, 5H), 5.33 (s, CH_2_, 2H), 2.28 (s, CH_3_, 3H), 2.26 (s, CH_3_, 3H), 1.92 (s, CH_3_, 3H). ^13^C{^1^H} NMR (100 MHz, CDCl_3_): δ 194.7, 140.4, 137.9, 136.7, 136.3, 135.9, 135.3, 134.2, 129.8, 129.5, 129.1, 128.2, 127.1, 126.1, 123.9, 123.6, 123.3, 120.1, 109.0, 50.8, 29.4, 19.9, 19.7. HRMS (ESI-TOF) m/z: [M + H]^+^ calcd for C_25_H_24_NO: 354.1852; found: 354.1852.

##### 1-(1-Benzyl-4-(3-nitrophenyl)-1*H*-indol-3-yl)ethan-1-one (**9al**)

Compound **9al** was synthesized by following general procedure D using 1-(1-benzyl-1*H*-indol-3-yl)ethan-1-one (**8a**, 50 mg, 0.2 mmol), AgOAc (67 mg, 0.4 mmol), Pd(OAc)_2_ (5 mg, 20 μmol, 10 mol %), and 1-iodo-3-nitrobenzene (**2l**, 100 mg, 0.4 mmol) at 100 °C for 48h and purified by silica gel column chromatography (80:20 hexane/ethyl acetate): **9al** (37 mg, 51%, a yellow solid, mp: 135–136 °C); ^1^H NMR (400 MHz, CDCl_3_): δ 8.25–8.19 (m, CH, 2H), 7.85 (s, CH, 1H), 7.73–7.70 (m, CH, 1H), 7.56 (t, *J* = 7.9 Hz, CH, 1H), 7.41–7.33 (m, CH, 5H), 7.24–7.18 (m, CH, 3H), 5.44 (s, CH_2_, 2H), 2.34 (s, CH_3_, 3H). ^13^C{^1^H} NMR (100 MHz, CDCl_3_): δ 191.5, 147.5, 144.7, 138.3, 136.6, 135.6, 134.8, 134.7, 129.3, 128.5, 128.3, 127.0, 125.0, 124.0, 123.8, 123.3, 121.7, 118.6, 110.4, 51.1, 28.3. HRMS (ESI-TOF) m/z: [M + H]^+^ calcd for C_23_H_19_N_2_O_3_: 371.1390; found: 371.1389.

##### 1-(1-Benzyl-7-fluoro-4-phenyl-1*H*-indol-3-yl)ethan-1-one (**9ba**)

Compound **9ba** was synthesized by following general procedure D using 1-(1-benzyl-7-fluoro-1*H*-indol-3-yl)ethan-1-one (**8b**, 54 mg, 0.2 mmol), AgOAc (67 mg, 0.4 mmol), Pd(OAc)_2_ (5 mg, 20 μmol, 10 mol %), and iodobenzene (**2a**, 45 μL, 0.4 mmol) at 90 °C for 6 h and purified by silica gel column chromatography (90:10 hexane/ethyl acetate): **9ba** (57 mg, 83%, a dirty white solid, mp: 112–113 °C); ^1^H NMR (400 MHz, CDCl_3_): δ 7.64 (s, CH, 1H), 7.44–7.31 (m, CH, 8H), 7.22 (d, *J* = 6.6 Hz, CH, 2H), 7.10–6.98 (m, CH, 2H), 5.54 (s, CH_2_, 2H), 1.97 (s, CH_3_, 3H). ^13^C{^1^H} NMR (100 MHz, CDCl_3_): δ 194.1, 149.5 (d, *J* = 245.2 Hz), 142.2, 136.6, 135.5, 132.6 (d, *J* = 3.8 Hz), 129.0, 128.6, 128.20, 128.17, 127.1, 127.0, 126.9 (d, *J* = 5.0 Hz), 125.5 (d, *J* = 9.9 Hz), 124.2 (d, *J* = 6.7 Hz), 120.6, 109.2 (d, *J* = 18.1 Hz), 53.0 (d, *J* = 6.9 Hz), 29.2. HRMS (ESI-TOF) m/z: [M + H]^+^ calcd for C_23_H_19_FNO: 344.1445; found: 344.1447.

##### 1-(1-Benzyl-7-bromo-4-phenyl-1*H*-indol-3-yl)ethan-1-one (**9ca**)

Compound **9ca** was synthesized by following general procedure D using 1-(1-benzyl-7-bromo-1*H*-indol-3-yl)ethan-1-one (**8c**, 66 mg, 0.2 mmol), AgOAc (67 mg, 0.4 mmol), Pd(OAc)_2_ (5 mg, 20 μmol, 10 mol %), and iodobenzene (**2a**, 45 μL, 0.4 mmol) at 75 °C for 5 h and purified by silica gel column chromatography (90:10 hexane/ethyl acetate): **9ca** (63 mg, 78%, a pale-brown solid, mp: 130–131 °C); ^1^H NMR (400 MHz, CDCl_3_): δ 7.62 (s, CH, 1H), 7.50 (d, *J* = 7.9 Hz, A part of AB system, CH, 1H), 7.45–7.28 (m, CH, 8H), 7.08 (d, *J* = 6.9 Hz, CH, 2H), 7.04 (d, *J* = 7.9 Hz, B part of AB system, CH, 1H), 5.90 (s, CH_2_, 2H), 1.95 (s, CH_3_, 3H). ^13^C{^1^H} NMR (100 MHz, CDCl_3_): δ 194.2, 142.0, 137.5, 136.9, 136.1, 134.1, 128.9 (2C), 128.4, 128.3, 127.9, 127.3, 126.5, 126.4, 124.9, 120.1, 103.2, 52.3, 29.3. HRMS (ESI-TOF) m/z: [M + H]^+^ calcd for C_23_H_19_BrNO: 404.0645; found: 404.0644.

##### 1-(1-Methyl-4-phenyl-1*H*-indol-3-yl)ethan-1-one (**9ea**)

Compound **9ea** was synthesized by following general procedure D using 1-(1-methyl-1*H*-indol-3-yl)ethan-1-one (**8e**, 70 mg, 0.4 mmol) and iodobenzene (**2a**, 90 μL, 0.8 mmol) at 100 °C for 5 h and purified by silica gel column chromatography (90:10 hexane/ethyl acetate): **9ea** (79 mg, 80%, a yellow gum); ^1^H NMR (400 MHz, CDCl_3_): δ 7.67 (s, CH, 1H), 7.45–7.32 (m, CH, 7H), 7.23 (d, *J* = 6.9 Hz, CH, 1H), 3.86 (s, CH_3_, 3H), 2.02 (s, CH_3_, 3H). ^13^C{^1^H} NMR (100 MHz, CDCl_3_): δ 193.6, 143.1, 138.4, 136.7, 135.6, 128.7, 128.0, 126.9, 124.1, 123.3, 123.2, 119.2, 108.7, 33.6, 29.0. HRMS (ESI-TOF) m/z: [M + H]^+^ calcd for C_17_H_16_NO: 250.1226; found: 250.1226.

##### Methyl 4-(3-Acetyl-1-methyl-1*H*-indol-4-yl)benzoate (**9eg**)

Compound **9eg** was synthesized by following general procedure D using 1-(1-methyl-1*H*-indol-3-yl)ethan-1-one (**8e**, 70 mg, 0.2 mmol) and methyl 4-iodobenzoate (**2g**, 210 mg, 0.8 mmol) at 100 °C for 8 h and purified by silica gel column chromatography (90:10 hexane/ethyl acetate): **9eg** (100 mg, 82%, off-white solid, mp: 149–150 °C); ^1^H NMR (400 MHz, CDCl_3_): δ 8.13–8.03 (m, AA′ part of AA′BB′ system, CH, 2H), 7.73 (s, CH, 1H), 7.49–7.42 (m, BB′ part of AA′BB′ system, CH, 2H), 7.40–7.32 (m, CH, 2H), 7.25–7.19 (m, CH, 1H), 3.92 (s, CH_3_, 3H), 3.89 (s, CH_3_, 3H), 2.18 (s, CH_3_, 3H). ^13^C{^1^H} NMR (100 MHz, CDCl_3_): δ 192.0, 167.2, 147.9, 138.5, 136.3, 135.8, 129.1, 128.7, 128.3, 124.4, 123.3, 122.9, 118.5, 109.3, 52.0, 33.7, 28.5. HRMS (ESI-TOF) m/z: [M + H]^+^ calcd for C_19_H_18_NO_3_: 308.1281; found: 308.1281.

##### 2-Phenyl-1*H*-indole (**11a**)^27^

Compound **11a** was synthesized by following general procedure D using 1-1*H*-indole-3-carboxylic acid (**10a**, 65 mg, 0.4 mmol) and iodobenzene (**2a**, 90 μL, 0.8 mmol) at 120 °C for 3 h and purified by silica gel column chromatography (95:5 hexane/ethyl acetate): **11a** (66 mg, 85%, off-white solid, mp: 188–189 °C); ^1^H NMR (400 MHz, CDCl_3_): δ 8.34 (bs, NH, 1H), 7.67 (t, *J* = 6.6 Hz, CH, 3H), 7.49–7.39 (m, CH, 3H), 7.34 (t, *J* = 7.3 Hz, CH, 1H), 7.22 (t, *J* = 7.4 Hz, CH, 1H), 7.15 (t, *J* = 7.5 Hz, CH, 1H), 6.85 (d, *J* = 1.3 Hz, CH, 1H). ^13^C{^1^H} NMR (100 MHz, CDCl_3_): δ 137.9, 136.8, 132.4, 129.3, 129.0, 127.7, 125.2, 122.4, 120.7, 120.3, 110.9, 100.0.

##### 2-(p-Tolyl)-1*H*-indole (**11b**)^27^

Compound **11b** was synthesized by following general procedure D using 1-1*H*-indole-3-carboxylic acid (**10a**, 65 mg, 0.4 mmol) and 1-iodo-4-methylbenzene (**2b**, 175 mg, 0.8 mmol) at 120 °C for 3 h and purified by silica gel column chromatography (95:5 hexane/ethyl acetate): **11b** (62 mg, 75%, a white solid, mp: 214–215 °C); ^1^H NMR (400 MHz, CDCl_3_): δ 8.30 (bs, NH, 1H), 7.63 (d, *J* = 7.7 Hz, CH, 1H), 7.60–7.54 (m, CH, 2H), 7.39 (d, *J* = 8.0 Hz, CH, 1H), 7.28–7.24 (m, CH, 2H), 7.22–7.17 (m, CH, 1H), 7.15–7.10 (m, CH, 1H), 6.80 (d, *J* = 1.5 Hz, CH, 1H), 2.40 (s, CH_3_, 3H). ^13^C{^1^H} NMR (100 MHz, CDCl_3_): δ 138.1, 137.7, 136.7, 129.7, 129.6, 129.4, 125.1, 122.1, 120.5, 120.2, 110.8, 99.4, 21.2.

##### 2-(*m*-Tolyl)-1*H*-indole (**11c**)^28^

Compound **11c** was synthesized by following general procedure D using 1-1*H*-indole-3-carboxylic acid (**10a**, 64 mg, 0.4 mmol) and 1-iodo-3-methylbenzene (**2c**, 102 μL, 0.8 mmol) at 120 °C for 3 h and purified by silica gel column chromatography (95:5 hexane/ethyl acetate): **11c** (65 mg, 78%, a white solid, mp: 128–129 °C); ^1^H NMR (400 MHz, CDCl_3_): δ 8.32 (bs, NH, 1H), 7.65 (d, *J* = 7.7 Hz, CH, 1H), 7.54–7.46 (m, CH, 2H), 7.41 (d, *J* = 8.0 Hz, CH, 1H), 7.35 (t, *J* = 7.6 Hz, CH, 1H), 7.23–7.18 (m, CH, 1H), 7.18–7.12 (m, CH, 2H), 6.84 (d, *J* = 1.3 Hz, CH, 1H), 2.44 (s, CH_3_, 3H). ^13^C{^1^H} NMR (100 MHz, CDCl_3_): δ 138.7, 138.1, 136.8, 132.3, 129.3, 129.0, 128.6, 125.9, 122.3, 122.3, 120.6, 120.2, 110.9, 99.9, 21.6.

##### 2-(4-(*tert*-Butyl)phenyl)-1*H*-indole (**11d**)^27^

Compound **11d** was synthesized by following general procedure D using 1-1*H*-indole-3-carboxylic acid (**10a**, 64 mg, 0.4 mmol) and 1-(*tert*-butyl)-4-iyodobenzen (**2d**, 90 μL, 0.8 mmol) at 120 °C for 3 h and purified by silica gel column chromatography (95:5 hexane/ethyl acetate): **11d** (87 mg, 87%, a white solid, mp: 251–252 °C); ^1^H NMR (400 MHz, CDCl_3_): δ 8.32 (bs, NH, 1H), 7.66–7.59 (m, CH, 3H), 7.48 (d, *J* = 8.4 Hz, CH, 2H), 7.40 (d, *J* = 7.9 Hz, CH, 1H), 7.22–7.16 (m, CH, 1H), 7.16–7.09 (m, CH, 1H), 6.81 (d, *J* = 1.5 Hz, CH, 1H), 1.37 (s, CH_3,_ 9H). ^13^C{^1^H} NMR (100 MHz, CDCl_3_): δ 150.9, 138.0, 136.7, 129.6, 129.4, 126.0, 124.9, 122.1, 120.5, 120.2, 110.8, 99.5, 34.7, 31.3.

##### 2-(4-Bromophenyl)-1*H*-indole (**11e**)^29^

Compound **11e** was synthesized by following general procedure D using 1-1*H*-indole-3-carboxylic acid (**10a**, 64 mg, 0.4 mmol) and 1-bromo-4-iodobenzene (**2e**, 227 mg, 0.8 mmol) at 120 °C for 3 h and purified by silica gel column chromatography (95:5 hexane/ethyl acetate): **11e** (90 mg, 84%, a white solid, mp: 209–210 °C); ^1^H NMR (400 MHz, CDCl_3_): δ 8.29 (bs, CH, 1H), 7.63 (d, *J* = 7.9 Hz, CH, 1H), 7.59–7.55 (m, CH, 2H), 7.54–7.50 (m, CH, 2H), 7.40 (d, *J* = 8.1 Hz, CH, 1H), 7.24–7.19 (m, CH, 1H), 7.16–7.11 (m, CH, 1H), 6.83–6.82 (m, CH, 1H). ^13^C{^1^H} NMR (100 MHz, CDCl_3_): δ 136.9, 136.7, 132.2, 131.3, 129.2, 126.6, 122.8, 121.5, 120.8, 120.5, 111.0, 100.6.

##### 2-(3,4-Dimethylphenyl)-1*H*-indole (**11k**)^30^

Compound **11k** was synthesized by following general procedure D using 1-1*H*-indole-3-carboxylic acid (**10a**, 64 mg, 0.4 mmol) and 4-iodo-1,2-dimethylbenzene (**2k**, 114 μL, 0.8 mmol) at 120 °C for 3 h and purified by silica gel column chromatography (95:5 hexane/ethyl acetate): **11k** (74 mg, 83%, a white solid, mp: 142–143 °C); ^1^H NMR (400 MHz, CDCl_3_): δ 8.29 (bs, NH, 1H), 7.66 (d, *J* = 7.4 Hz, CH, 1H), 7.47 (s, CH, 1H), 7.44–7.38 (m, CH, 2H), 7.25–7.12 (m, CH, 3H), 6.81 (s, CH, 1H), 2.36 (s, CH_3_, 3H), 2.33 (s, CH_3_, 3H). ^13^C{^1^H} NMR (100 MHz, CDCl_3_): δ 138.2, 137.3, 136.7, 136.4, 130.3, 130.0, 129.4, 126.5, 122.6, 122.1, 120.5, 120.2, 110.8, 99.3, 20.0, 19.6.

### Gram-Scale Reaction and Synthetic Applications

#### (a) Gram-Scale Reaction of **3aa**

Compound **3aa** (1.05 g, 69%) was synthesized by following general procedure D using 1*H*-indole-3-carbaldehyde (**1a**) (1.0 g, 6.9 mmol), Pd(OAc)_2_ (154 mg, 0.69 mmol, 10 mol %), AgOAc (2.3 g, 13.8 mmol, 2 equiv), and iodobenzene (**2a**) (1.54 mL, 13.8 mmol, 2 equiv) at 100 °C for 4 h and purified by silica gel column chromatography (80:20 hexane/ethyl acetate).

#### (b) Gram-Scale Reaction of **5aa**

Compound **5aa** (960 mg, 65%) was synthesized by following general procedure D using 1-(1*H*-indol-3-yl)ethan-1-one (**4a**) (1.0 g, 6.3 mmol), Pd(OAc)_2_ (141 mg, 0.63 mmol, 10 mol %), AgOAc (2.1 g, 0.8 mmol, 2 equiv), and iodobenzene (**2a**) (1.4 mL, 12.6 mmol, 2 equiv) at 120 °C for 10 h and purified by silica gel column chromatography (90:10 hexane/ethyl acetate).

#### (c) *N*-Benzylation Reaction of **5aa**

##### 1-(1-Benzyl-4-phenyl-1*H*-indol-2-yl)ethan-1-one (**12**)

Compound **12** (80 mg, 82%) was prepared starting from **5aa** (71 mg, 0.3 mmol) according to the General Procedure C. Yellow oil. ^1^H NMR (400 MHz, CDCl_3_): δ 7.61 (dd, *J* = 8.1, 1.1 Hz, CH, 2H), 7.45 (t, *J* = 7.6 Hz, CH, 2H), 7.42 (s, CH, 1H), 7.39–7.33 (m, CH, 1H), 7.33–7.27 (m, CH, 2H), 7.20–7.11 (m, CH, 4H), 7.04–6.97 (m, CH, 2H), 5.81 (s, CH_2_, 2H), 2.49 (s, CH_3_, 3H). ^13^C{^1^H} NMR (100 MHz, CDCl_3_): δ 191.3, 140.5, 140.3, 138.3, 136.9, 134.7, 128.9, 128.8, 128.6, 127.6, 127.2, 126.6, 126.5, 124.6, 120.8, 112.5, 110.0, 48.4, 28.2. HRMS (ESI-TOF) *m*/*z*: [M + H]^+^ calcd for C_23_H_20_NO: 326.1539; found: 326.1539.

#### (d) Cross-Coupling of 2-Thienyl Boronic Acid and **5ca**(31)

##### 1-(4-Phenyl-7-(thiophen-2-yl)-1*H*-indol-2-yl)ethan-1-one (**13**)

To a solution of 1-(7-bromo-4-phenyl-1*H*-indol-2-yl)ethan-1-one (**5ca**, 63 mg, 0.2 mmol) and thiophen-2-ylboronic acid (31 mg, 0.24 mmol, 1.2 equiv) in DME/water (15 mL, 2:1 v/v, 0.013 M for **5ca**) was added Na_2_CO_3_ (42 mg, 0.4 mmol, 2 equiv). After degassing, Pd(PPh_3_)_4_ (12 mg, 10 μmol) was added and the mixture was boiled in a preheated oil bath at 100 °C for 18 h. Then, the mixture was cooled to room temperature and the solution was extracted with CH_2_Cl_2_ (3 × 30 mL). The organic phase was combined, washed with water (3 × 20 mL), dried with Na_2_SO_4_, and concentrated under reduced pressure. The crude material was purified by column chromatography on silica gel (EtOAc/hexane, 1:9) to afford **13** as a red solid (41 mg, 85% yield, mp: 163-164 °C); ^1^H NMR (400 MHz, CDCl_3_): δ 9.45 (bs, NH, 1H), 7.73–7.69 (m, CH, 2H), 7.59–7.53 (m, CH, 3H), 7.49–7.39 (m, CH, 4H), 7.27 (d, *J* = 7.6 Hz, CH, 1H), 7.23 (dd, *J* = 5.0, 3.6 Hz, CH, 1H), 2.59 (s, CH_3_, 3H). ^13^C{^1^H} NMR (100 MHz, CDCl_3_): δ 190.2, 140.0, 139.9, 136.6, 136.0, 135.2, 128.82, 128.75, 128.2, 127.7, 126.8, 126.1, 125.5, 125.0, 121.1, 118.6, 109.8, 25.9. HRMS (ESI-TOF) *m*/*z*: [M + H]^+^ calcd for C_20_H_16_NOS: 318.0947; found: 318.0947.

#### (e) Construction of Functionalized Natural Product

##### (1-Benzyl-4-(4-(*tert*-butyl)phenyl)-1*H*-indol-3-yl)(6-methoxy-9*H*-pyrido[3,4-b]indol-1-yl)methanone (**16**)

Compounds **16** and **17** were prepared according to the method reported in the literature.^32b^ 3-Acetylindole derivative **9ad** (57 mg, 0.15 mmol), 5-methoxytryptamine (**15**, 28 mg, 0.15 mmol, 1 equiv), I_2_ (30 mg, 0.12 mmol, 0.8 equiv), hydrogen peroxide (30% aqueous solution, 1.5 equiv), and DMSO (2 mL, 2 M for **9ad**) were placed in a sealed tube (15 mL) with a magnetic stir bar. The resulting mixture was stirred in a preheated oil bath at 110 °C for 5 h, monitoring the reaction by TLC. Once the reaction was complete, the mixture was cooled to rt, diluted with water (50 mL), and then the reaction mixture was extracted with EtOAc (3 × 50 mL). The combined organic layers were washed with 10% Na_2_S_2_O_3_ solution, and then with brine, dried over anhydrous Na_2_SO_4_, and evaporated. The residue was purified by column chromatography on silica gel (80:20 hexane/ethyl acetate) to give **16** (63 mg, 75%, yellow solid, mp: 269-270 °C); ^1^H NMR (400 MHz, CDCl_3_): δ 10.04 (bs, NH, 1H), 8.40–8.30 (m, CH, 2H), 7.90 (d, *J* = 5.0 Hz, CH, 1H), 7.50 (d, *J* = 2.4 Hz, CH, 1H), 7.36–7.27 (m, CH, 7H), 7.26–7.14 (m, CH, 5H), 7.10 (d, *J* = 8.3 Hz, CH, 2H), 5.42 (s, CH_2_, 2H), 3.89 (s, CH_3_, 3H), 1.07 (s, CH_3_, 9H). ^13^C{^1^H} NMR (100 MHz, CDCl_3_): δ 189.9, 154.3, 148.9, 139.8, 138.1, 137.6, 137.3, 137.2, 136.7, 136.7, 136.1, 135.8, 130.8, 129.0, 128.1, 127.9, 127.2, 125.3, 124.4, 123.9, 123.3, 121.2, 118.7, 117.5, 116.4, 112.6, 109.1, 103.7, 56.1, 51.0, 34.2, 31.2. HRMS (ESI-TOF) m/z: [M + H]^+^ calcd for C_38_H_34_N_3_O_2_: 564.2646; found: 564.2645.

##### (6-Methoxy-9*H*-pyrido[3,4-b]indol-1-yl)(4-phenyl-1*H*-indol-2-yl)methanone (**17**)

Compound **17** was was synthesized starting from **5aa** (48 mg, 0.25 mmol) (prepared according to the literature procedure^32b^). The residue was purified by column chromatography on silica gel (80:20 hexane/ethyl acetate) to give **17** (86 mg, 83%, yellow solid, mp: >300 °C); ^1^H NMR (400 MHz, CDCl_3_): δ 12.18 (bs, NH, 1H), 10.54 (bs, NH, 1H), 8.58 (d, *J* = 4.8 Hz, CH, 1H), 8.13 (d, *J* = 4.8 Hz, CH, 1H), 7.96 (s, CH, 1H), 7.77 (d, *J* = 7.2 Hz, CH, 2H), 7.62–7.41 (m, CH, 7H), 7.29–7.20 (m, CH, 2H), 3.93 (s, CH_3_, 3H). ^13^C{^1^H} NMR (100 MHz, CDCl_3_): δ 182.3, 154.7, 140.6, 138.4, 137.5, 137.4, 137.1, 136.8, 136.6, 136.0, 131.8, 128.9, 128.6, 127.4, 126.4, 125.6, 121.0, 120.3, 119.3, 118.6, 112.9, 111.7, 111.3, 103.8, 56.1. HRMS (ESI-TOF) m/z: [M + H]^+^ calcd for C_27_H_20_N_3_O_2_: 418.1550; found: 418.1552.

### Control Experiments

#### a 3,2-Carbonyl Migration from **6ca**

##### 1-(7-Bromo-4-phenyl-1*H*-indol-2-yl)ethan-1-one (**5ca**)

Compound **6ca** (63 mg, 0.2 mmol), Pd(OAc)_2_ (5 mg, 20 μmol, 10 mol %), and AgOAc (67 mg, 0.4 mmol, 2 equiv) were weighed in air and placed in a sealed tube (15 mL) with a magnetic stir bar. To the reaction mixture, HFIP/TFA (2 mL, 1:1, v/v) was added. The reaction mixture was then heated to 120 °C for 10 h under vigorous stirring. Upon completion, the reaction mixture was cooled to room temperature, the solvents were removed under reduced pressure, and the resulting mixture was purified by a silica gel column chromatography column to give **5ca** (46 mg, 74%) using hexane/EtOAc (85:15 hexane/ethyl acetate).

#### b Reaction under Standard Conditions without **2a**

##### 2,2,2-Trifluoro-1-(1*H*-indol-3-yl)ethan-1-one (**18**)^33^

Reaction of **10a** (or **10b**) under standard condition without **2a** gave the compound **18**. 2,2,2-trifluoro-1-(1*H*-indol-3-yl)ethan-1-one (**18**) (63 mg, 75% (or 59 mg, 70%), white solid, mp 153–154 °C); ^1^H NMR (400 MHz, DMSO-*d*_6_): δ 12.72 (bs, NH, 1H), 8.51–8.47 (m, CH, 1H), 8.24–8.16 (m, CH, 1H), 7.62–7.56 (m, CH, 1H), 7.38–7.27 (m, CH, 2H). ^13^C{^1^H} NMR (100 MHz, DMSO-*d*_6_): δ 174.1 (q, *J* = 33.8 Hz), 137.6 (q, *J* = 4.8 Hz), 136.6, 125.7, 124.3, 123.4, 121.1, 116.9 (q, *J* = 291.7 Hz), 113.0, 108.8.

#### c Reaction under Standard Conditions of **19a** and **2a**

Compound **11a** (27 mg, 70%) was synthesized by following general procedure D using 1*H*-indole (**19a**, 24 mg, 0.2 mmol), AgOAc (67 mg, 0.4 mmol), Pd(OAc)_2_ (5 mg, 20 μmol, 10 mol %), and iodobenzene (**2a**, 45 μL, 0.4 mmol) at 120 °C for 3 h and purified by silica gel column chromatography (95:15 hexane/ethyl acetate).

#### d Reaction under Standard Conditions of **10c** without **2a**/with **2a**

##### 2-Methyl-1*H*-indole (**19b**)^46^

(Red solid; mp: 59–60 °C); ^1^H NMR (400 MHz, CDCl_3_): δ 7.75 (bs, NH, 1H), 7.56 (d, *J* = 7.6 Hz, CH, 1H), 7.28 (d, *J* = 7.9 Hz, CH, 1H), 7.18–7.09 (m, CH, 2H), 6.25 (s, CH, 1H), 2.44 (s, CH, 3H). ^13^C{^1^H} NMR (100 MHz, CDCl_3_): δ 136.1, 135.2, 129.1, 121.0, 119.7 (2C), 110.3, 100.4, 13.8.

#### e Reaction under Standard Conditions of **19b** and **2a**

In the reaction of **19b** and **2a** under standard conditions, no products were observed.

## Supporting Information Available

NMR and HRMS spectra of compounds (PDF)
