# Supplementary material for: Directed C–H Functionalization of C3-Aldehyde, Ketone, and Acid/Ester-Substituted Free (NH) Indoles with Iodoarenes via a Palladium Catalyst System
Source: J Org Chem. 2022 May 24;88(3):1299–318. doi: 10.1021/acs.joc.2c00716 (PMC9903333; doi:10.1021/acs.joc.2c00716)

Supporting Information

Directed C–H Functionalization of C3-Aldehyde, Ketone, and Acid/Ester-Substituted Free (NH) Indoles with Iodoarenes *via* a Palladium Catalyst System

Yunus Taskesenligil, Murat Aslan, Tuba Cogurcu, Nurullah Saracoglu*

Department of Chemistry, Faculty of Sciences, Atatürk University, Erzurum 25240, Turkey

*E-mail: nsarac@atauni.edu.tr

Table of contents

1. Optimization of Reaction Conditions[a] ……..…………………………………………. SI-2

2. Comparisons of ^{1}H NMR spectra ……….………………………………………….... SI-3

3. NOE Experiments ……………………………………………………….….. SI-4-SI-5

4. ^{1}H NMR (400 MHz) and $^{13}C\{^1H\}$ NMR (100 MHz) spectra of compounds SI-6-SI-81

5. HRMS spectra of compounds SI-82-SI-94

1. Optimization of Reaction Conditions[a]

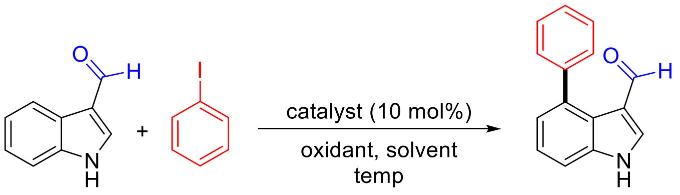

entry	catalyst	oxidant	temp (ºC)	solvent	additive	time (h)	yield (**3aa**, %)[b]
1	$Pd(OAc)_2$	AgOAc	100	HFIP	-	10	trace
2	$Pd(OAc)_2$	AgOAc	100	HOAc	-	10	trace
3	$Pd(OAc)_2$	AgOAc	100	DCE	-	12	trace
4	$Pd(OAc)_2$	AgOAc	100	DMA	-	12	trace
5	$Pd(OAc)_2$	AgOAc	100	DMF	-	12	trace
6	$Pd(OAc)_2$	AgOAc	100	Tol	-	12	trace
7	$Pd(OAc)_2$	AgOAc	100	TFA	-	10	23
8	$Pd(OAc)_2$	AgOAc	100	TFE	-	12	trace
9	$Pd(OAc)_2$	AgOAc	100	HFIP	DCE	5	trace
10	$Pd(OAc)_2$	AgOAc	100	HFIP	DMA	5	trace
11	$Pd(OAc)_2$	AgOAc	100	HFIP	DMF	5	trace
12	$Pd(OAc)_2$	AgOAc	100	HFIP	Tol	5	trace
13	$Pd(OAc)_2$	AgOAc	100	HFIP	TFE	5	trace
14	$Pd(OAc)_2$	AgOAc	100	HFIP	HOAc	10	47
15	$Pd(OAc)_2$	AgOAc	100	HFIP	TFA	10	57
16	$Pd(OAc)_2$	AgOAc	100	HFIP	TFA	5	70
**17**	**$Pd(OAc)_2$**	**AgOAc**	**100**	**HFIP**	**TFA**	**3.5**	**87**
18	$Pd(OAc)_2$	AgOAc	120	HFIP	TFA	3.5	72
19	$Pd(OAc)_2$	AgOAc	65	HFIP	TFA	15	81
20	$Pd(OAc)_2$	Ag_2CO_3	100	HFIP	TFA	3.5	45
21	$Pd(OAc)_2$	Ag_2O	100	HFIP	TFA	3.5	70
22	$Pd(OAc)_2$	$Cu(OAc)_2{\cdot}H_2O$	100	HFIP	TFA	3.5	trace
23	$Pd(OAc)_2$	AgTFA	100	HFIP	-	5	trace
24	$PdCl_2$	AgOAc	100	HFIP	TFA	3.5	71
25	$Pd(PPh_3)_2Cl_2$	AgOAc	100	HFIP	TFA	3.5	67
26	$Pd(TFA)_2$	AgOAc	100	HFIP	-	3.5	trace
27	$Pd(TFA)_2$	AgTFA	100	HFIP	-	36	74
**28**	**$Pd(TFA)_2$**	**AgOAc**	**100**	**HFIP**	**TFA**	**3.5**	**87**

[a]Reaction conditions: **1a** (0.40 mmol), $Pd(OAc)_2$ (10 mol%), **2a** (0.80 mmol), oxidant (0.80 mmol), solvent (1 mL), additive (1 mL). [b]Isolated yield.

2. Comparisons of ¹H NMR spectra

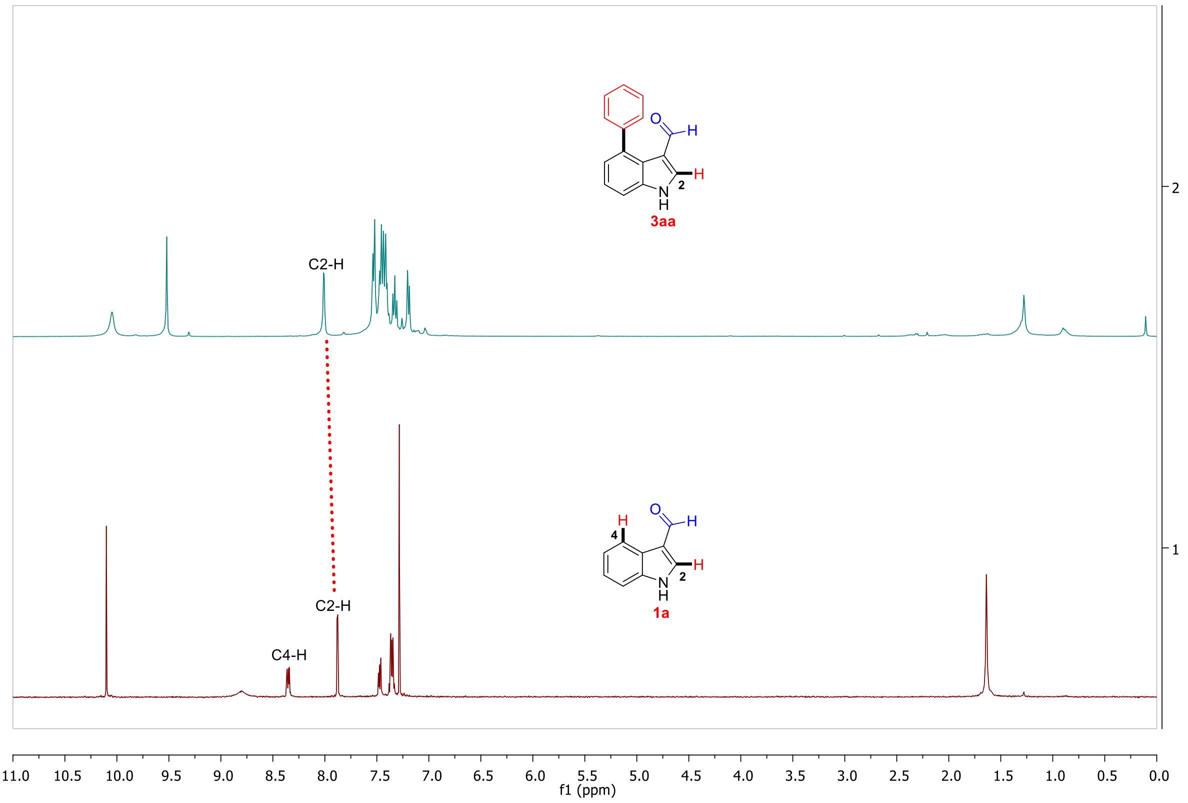

Figure 1S. Comparisons of ¹H NMR (400 MHz, $CDCl_3$) spectra of 1*H*-indole-3-carbaldehyde (**1a**) and 4-phenyl-1*H*-indole-3-carbaldehyde (**3aa**)

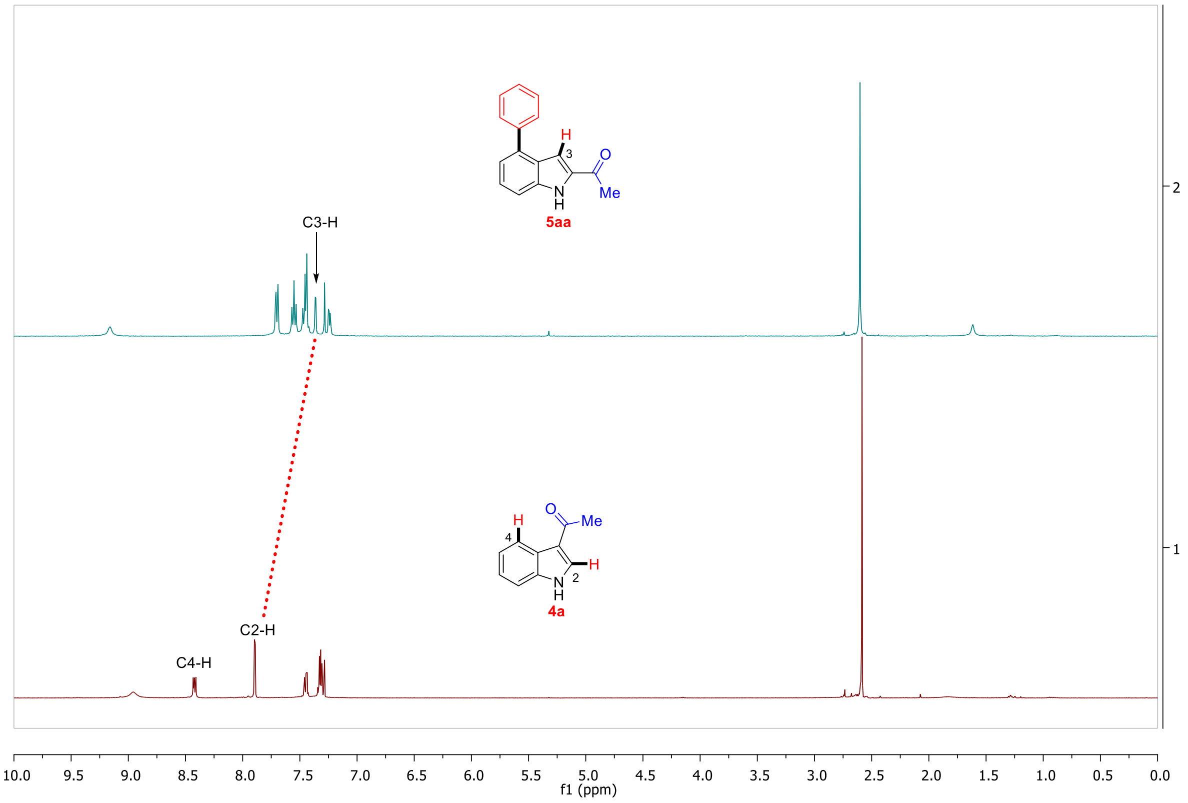

Figure 2S. Comparisons of ¹H NMR spectra of 1-(1*H*-indol-3-yl)ethan-1-one (**4a**) and 1-(4-phenyl-1*H*-indol-2-yl)ethan-1-one (**5aa**) (400 MHz ¹H NMR spectra, $CDCl_3$)

3. NOE Experiments

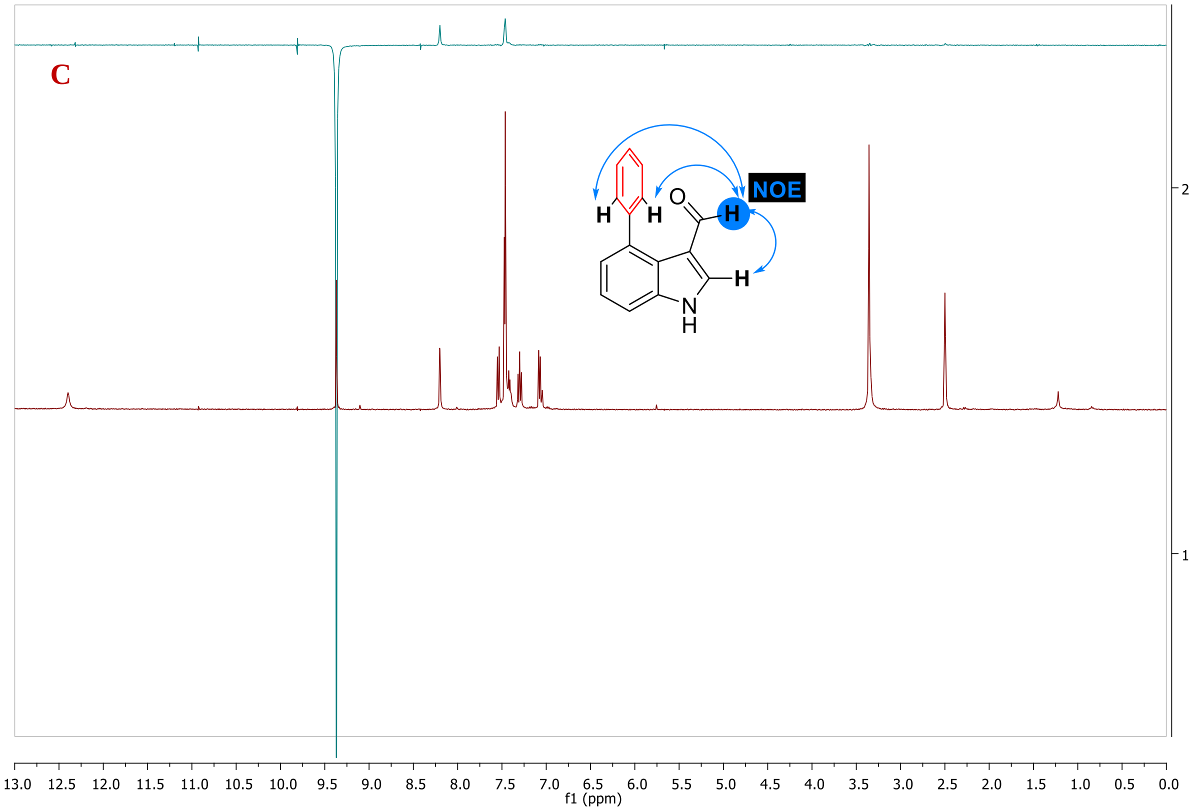

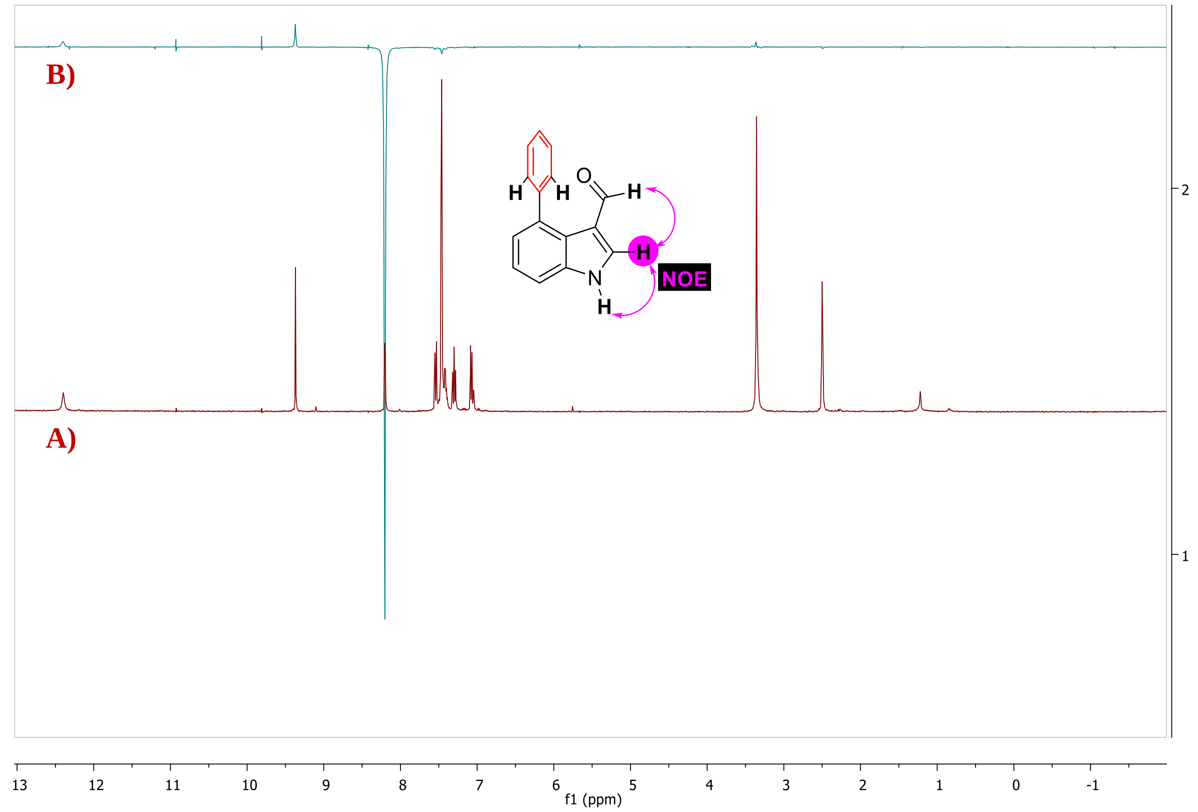

Figure 3S. ^{1}H NMR spectrum (A) and ^{1}H NOE NMR spectra (B, C) spectra of 4-phenyl-1*H*-indole-3-carbaldehyde (**3aa**) (400 MHz, DMSO-d_6).

In Figure 3S, there were NOE correlations between C2-H, NH, *ortho*-protons of the phenyl ring, and aldehyde, which are assigned to be **3aa**.

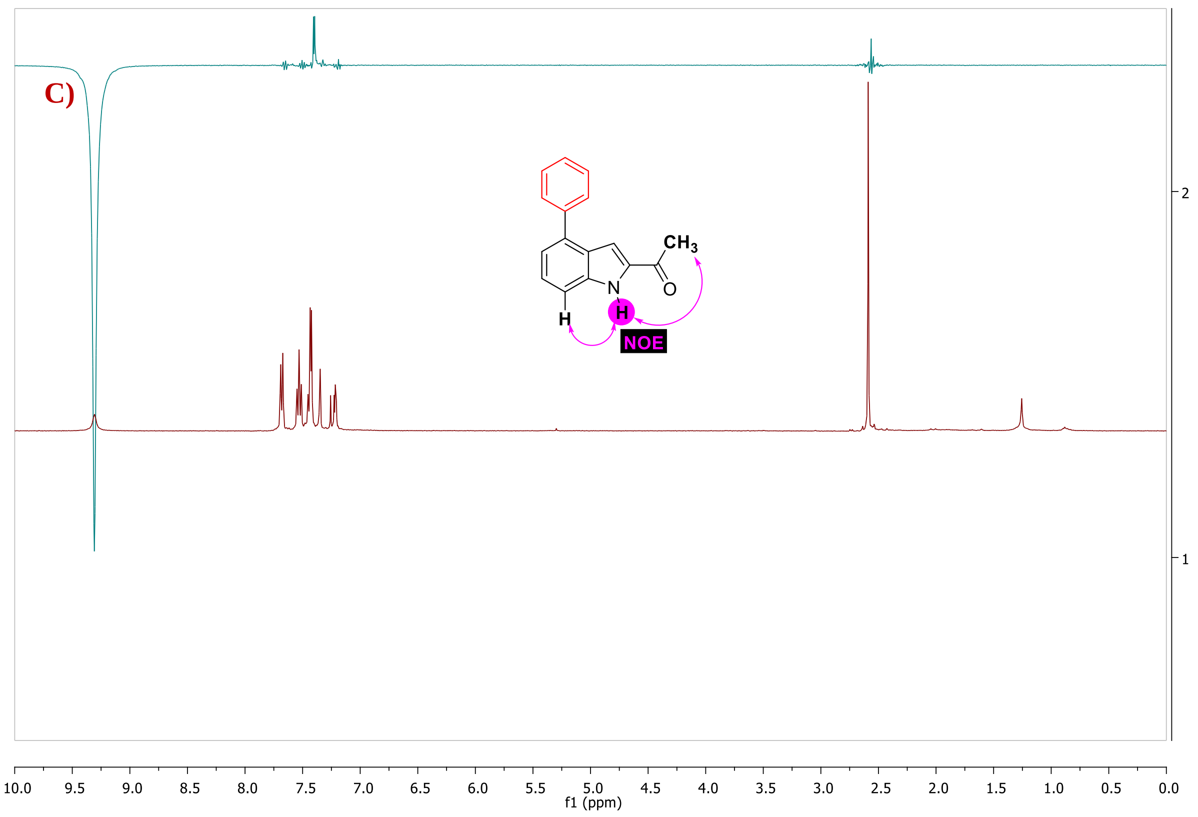

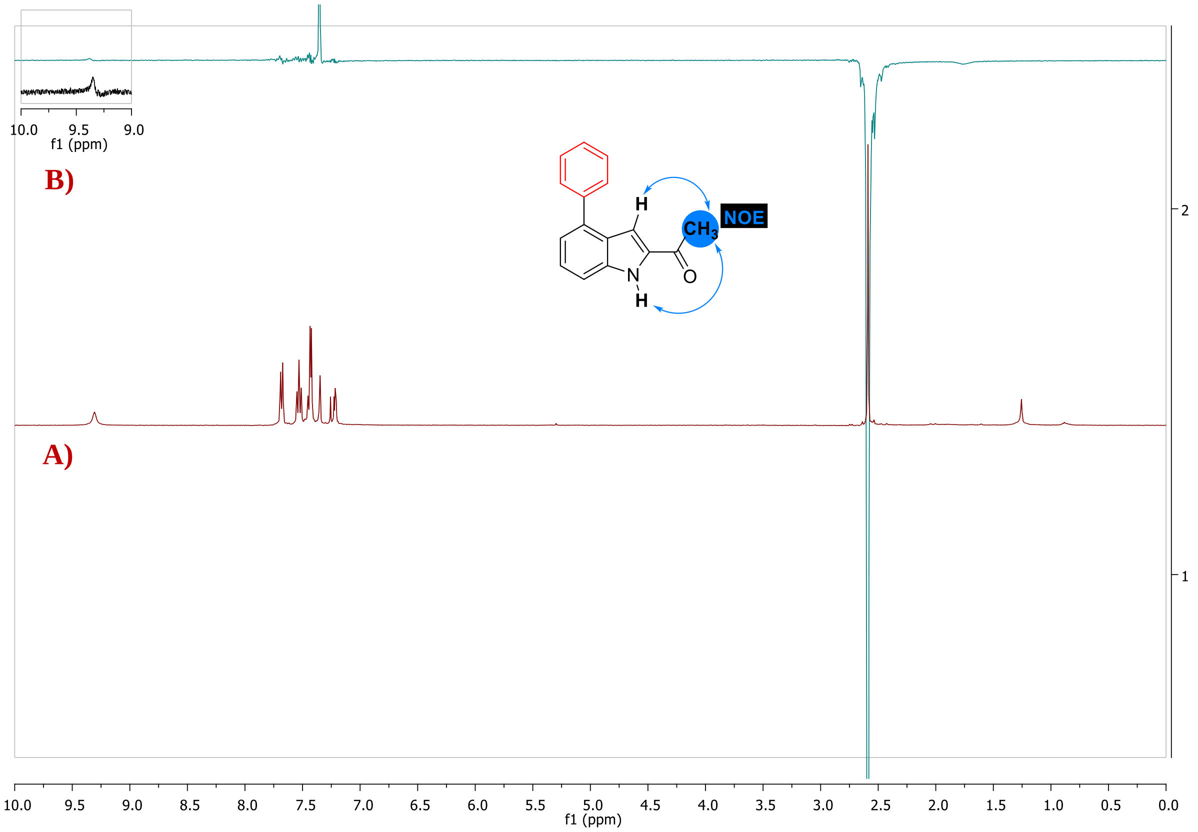

Figure 4S. ^{1}H NMR spectrum (A) and ^{1}H NOE NMR spectra (B, C) spectra of 1-(4-phenyl-1*H*-indol-2-yl)ethan-1-one (**5aa**) (400 MHz, $CDCl_3$).

In Figure 4S, there were NOE correlations between C7-H, C3-H, NH, and CH_3, which is assigned to be **5aa**.

4. ^{1}H NMR (400 MHz) and ^{13}C{^{1}H} NMR (100 MHz) spectra of compounds

7-Fluoro-1*H*-indole-3-carbaldehyde (1b)

^{1}H NMR spectrum of 1b (400 MHz, DMSO-d_6)

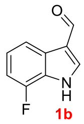

1b

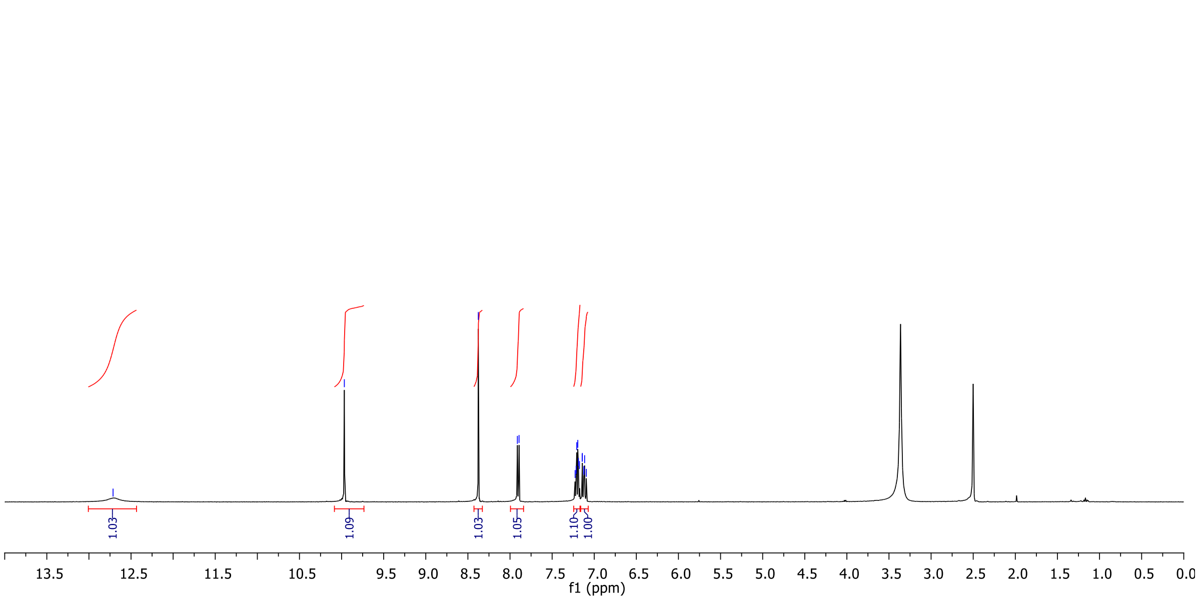

^{13}C{^{1}H} NMR spectrum of 1b (100 MHz, DMSO-d_6)

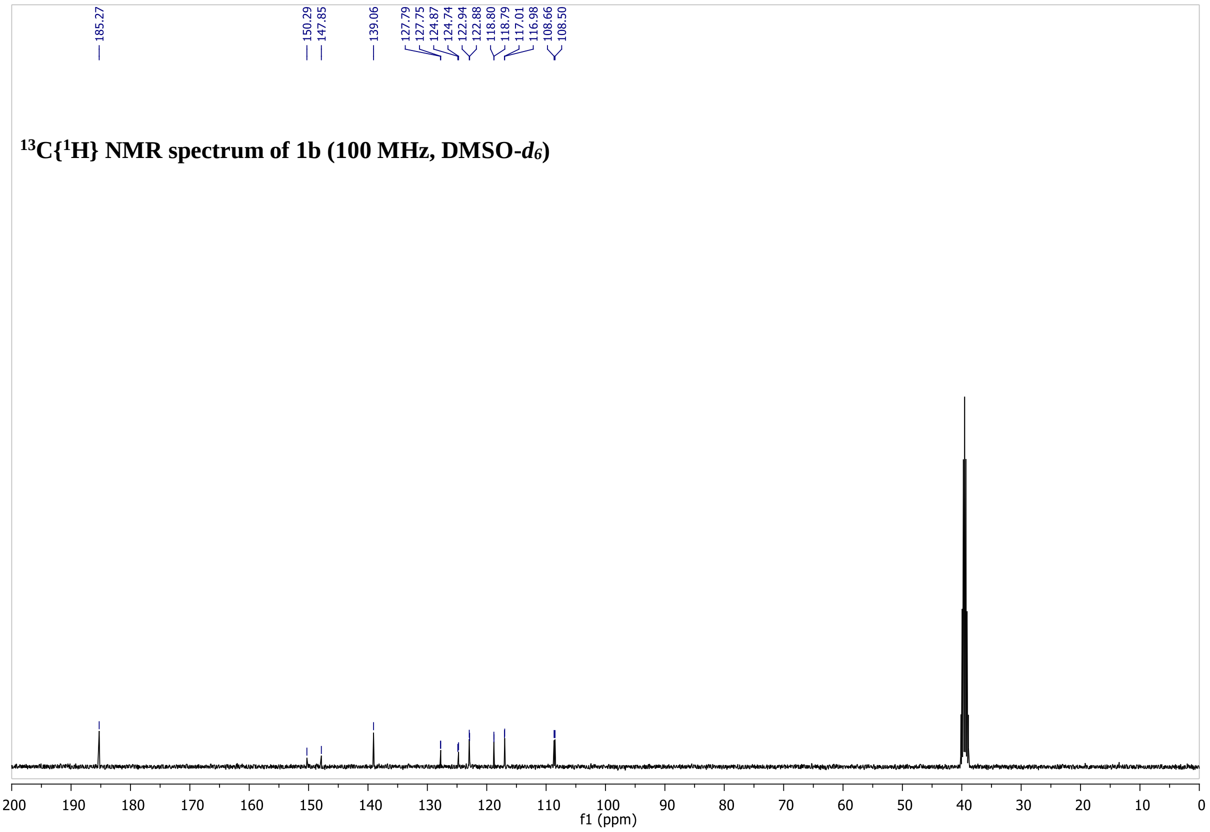

7-Bromo-1*H*-indole-3-carbaldehyde (1c)

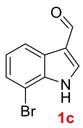

1c

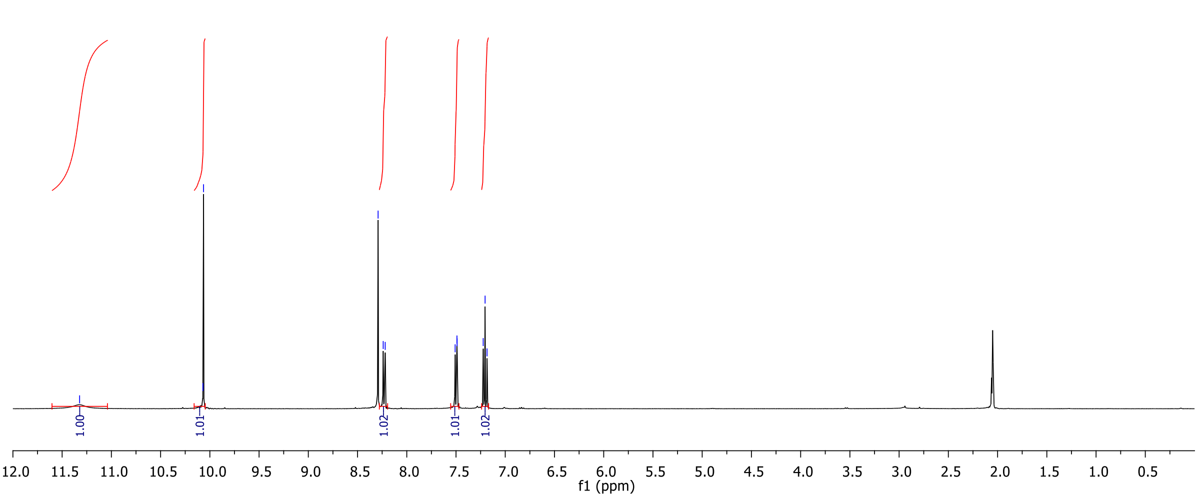

^{1}H NMR spectrum of 1c (400 MHz, Acetone-d_6)

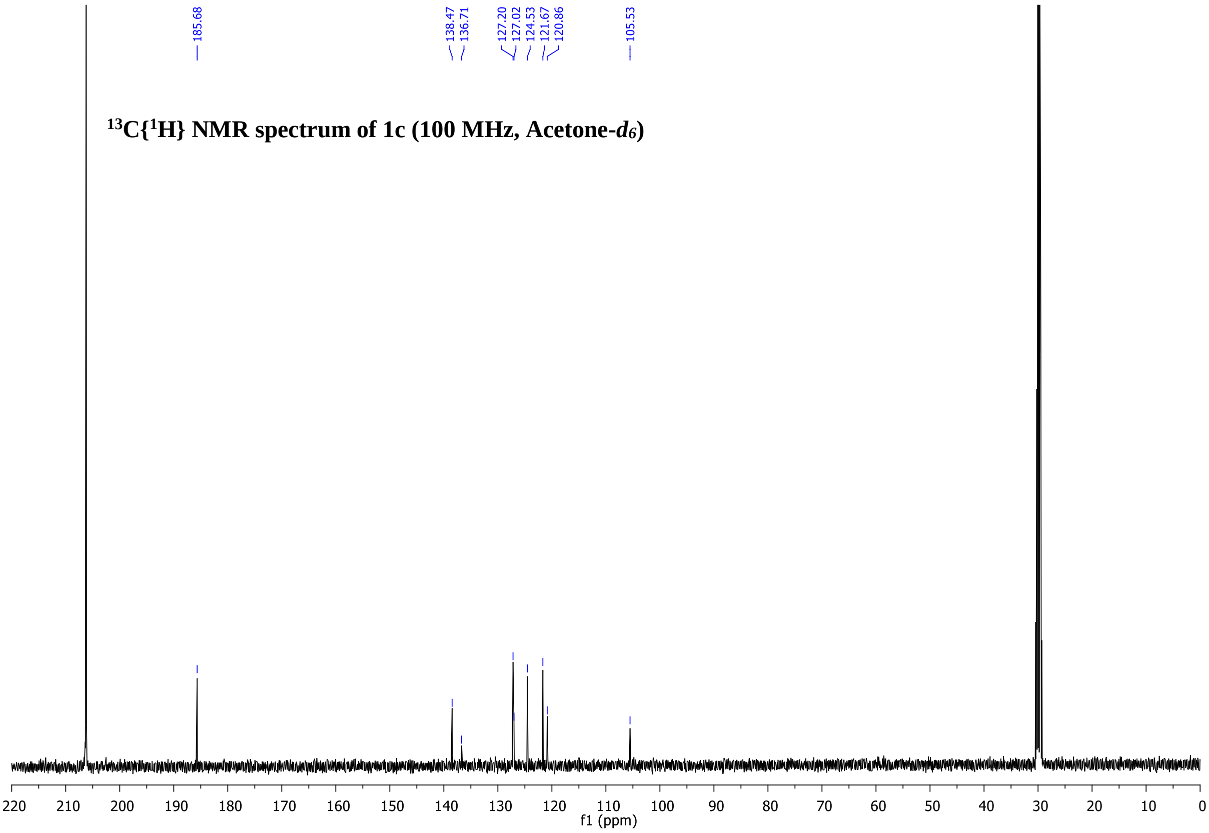

^{13}C{^{1}H} NMR spectrum of 1c (100 MHz, Acetone-d_6)

2-Methyl-1*H*-indole-3-carbaldehyde (1d)

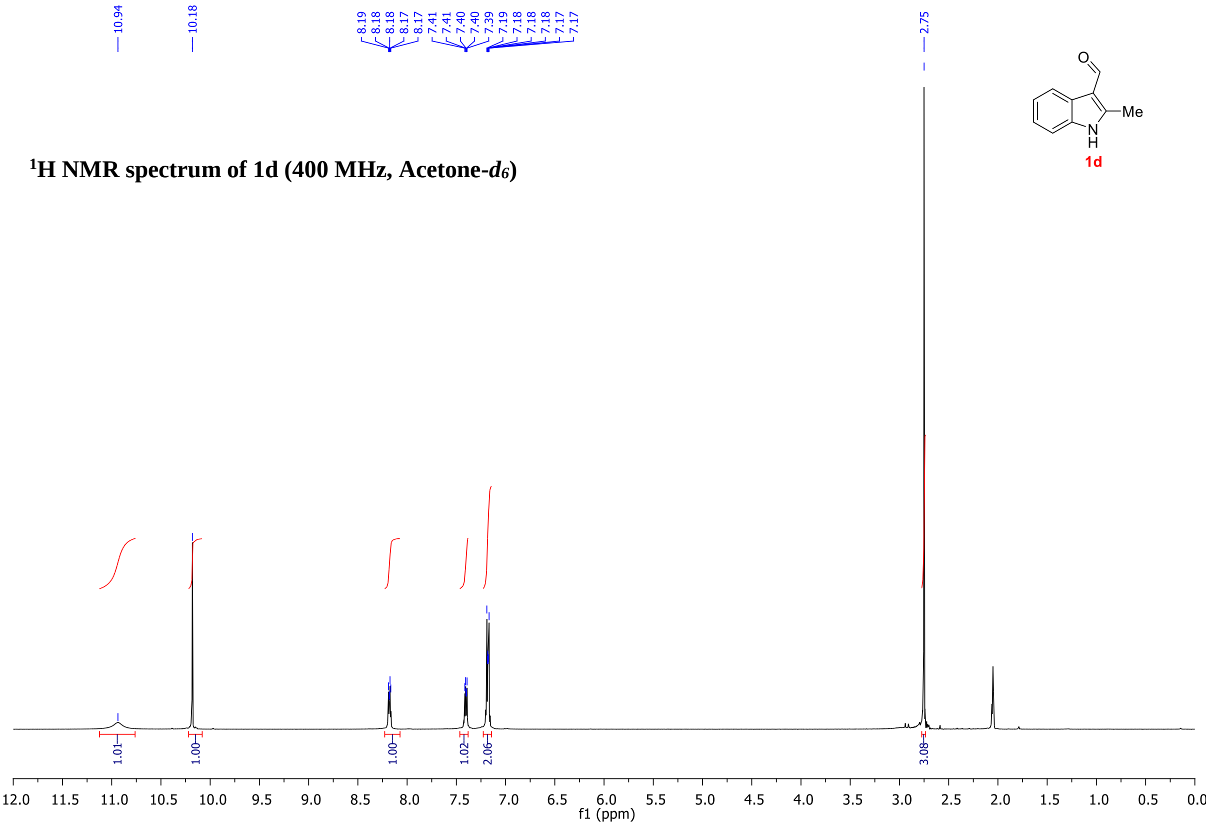

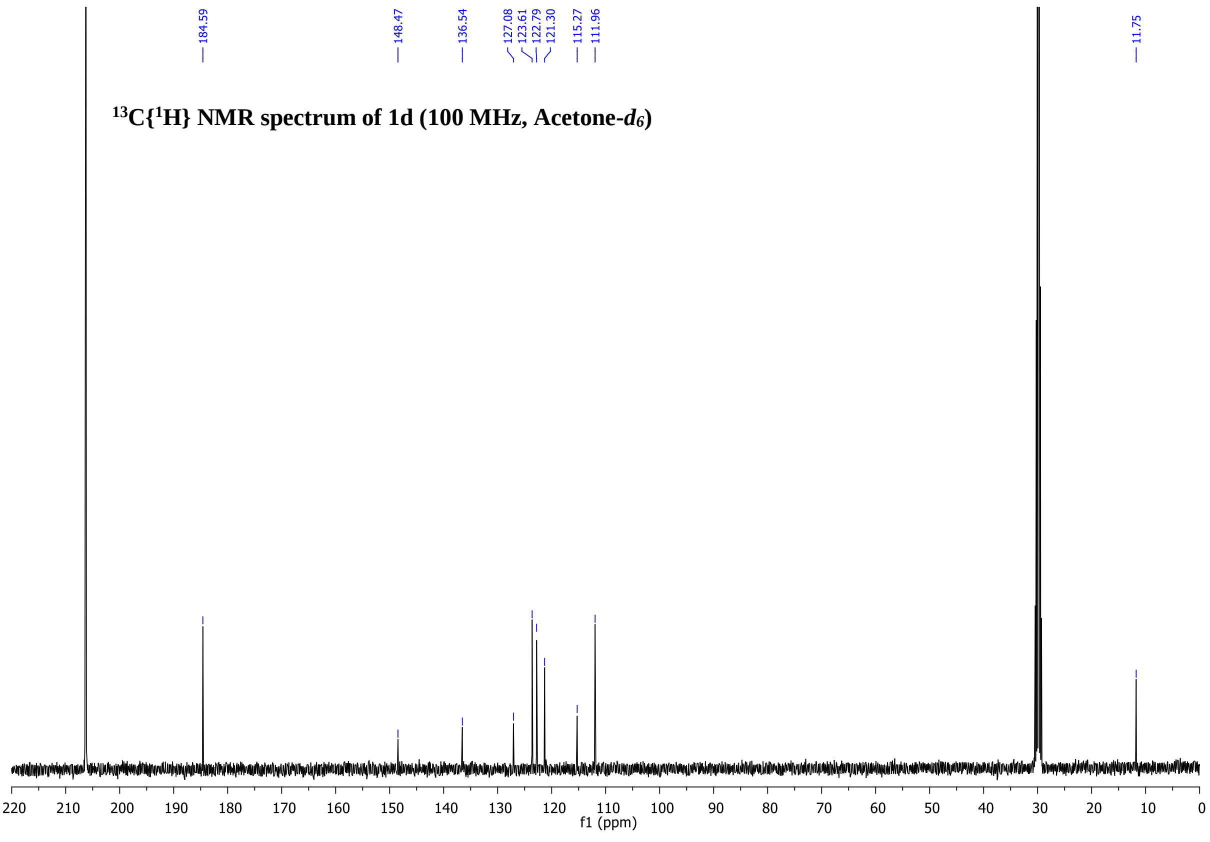

5-Bromo-1*H*-indole-3-carbaldehyde (1e)

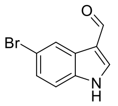

1e

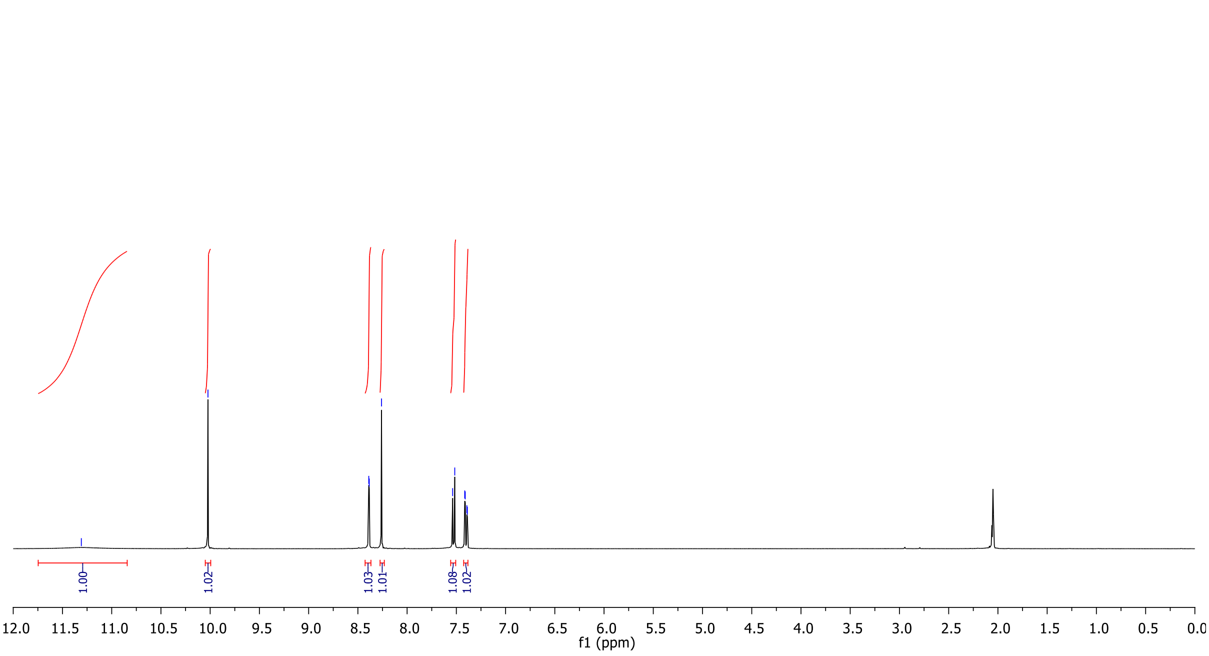

^{1}H NMR spectrum of 1e (400 MHz, Acetone-d_6)

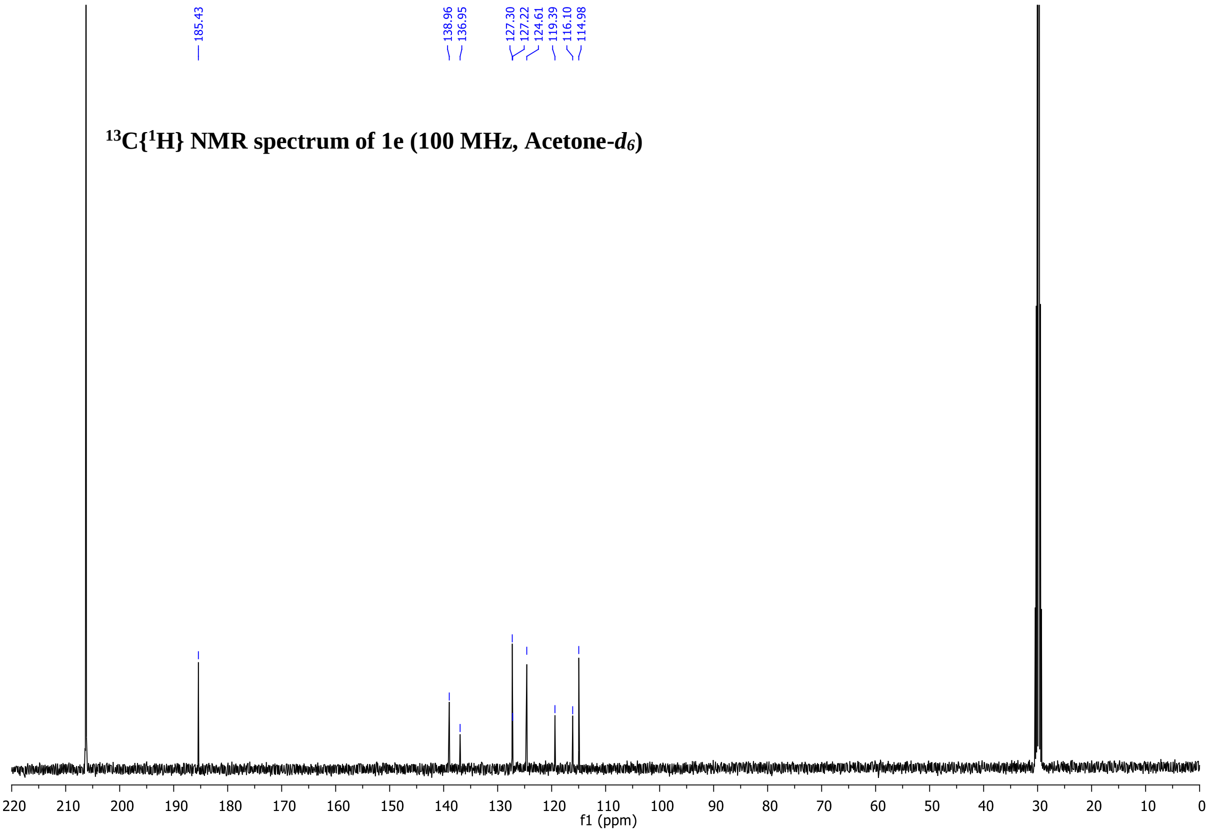

$^{13}C\{^{1}H\}$ NMR spectrum of 1e (100 MHz, Acetone-d_6)

5-(Benzyloxy)-1*H*-indole-3-carbaldehyde (1f)

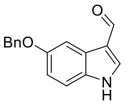

1f

^{1}H NMR spectrum of 1f (400 MHz, DMSO-*d*6)

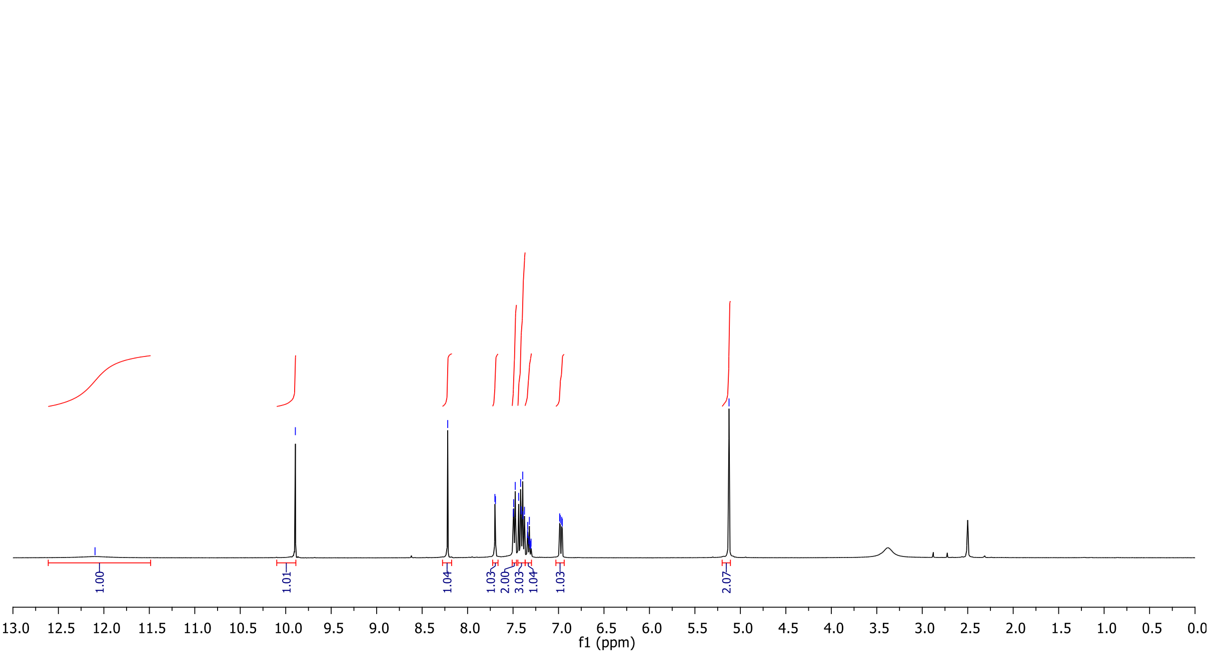

^{13}C{^{1}H} NMR spectrum of 1f (100 MHz, DMSO-*d*6)

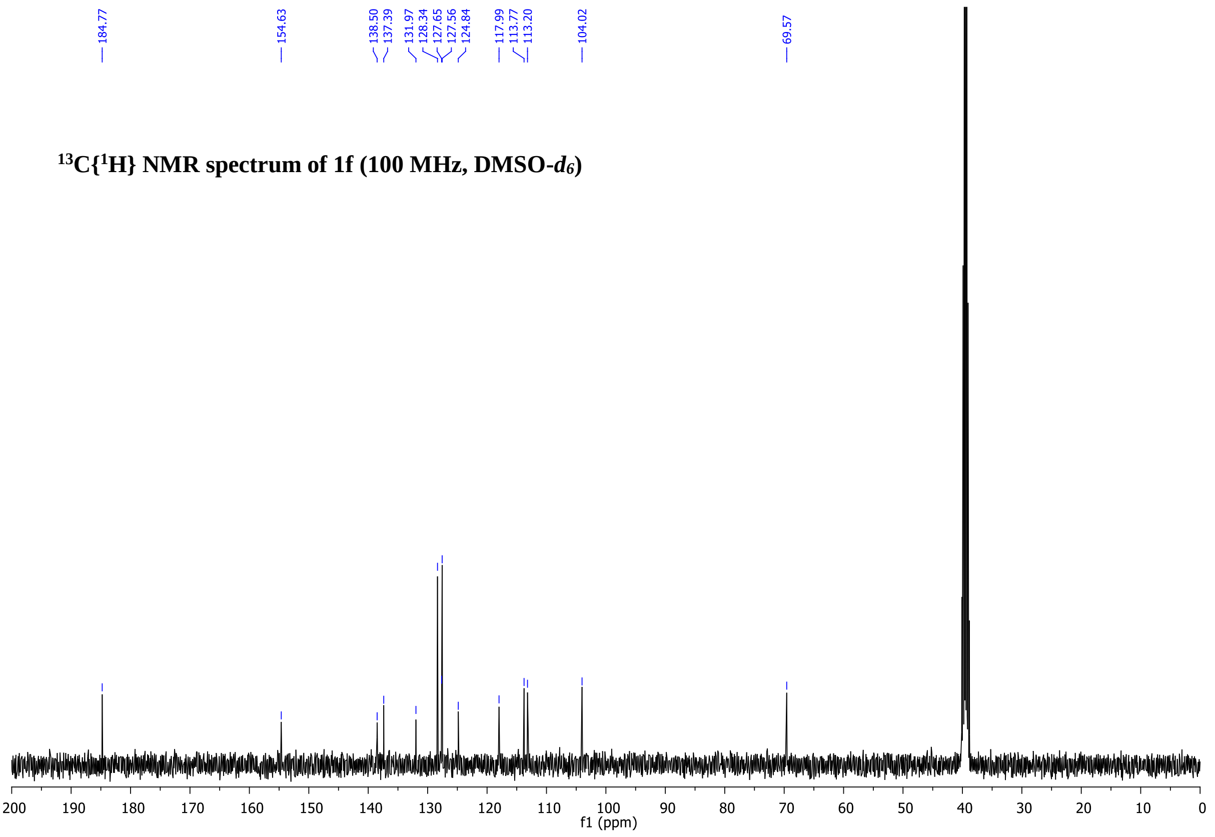

5-Nitro-1*H*-indole-3-carbaldehyde (1g)

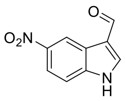

1g

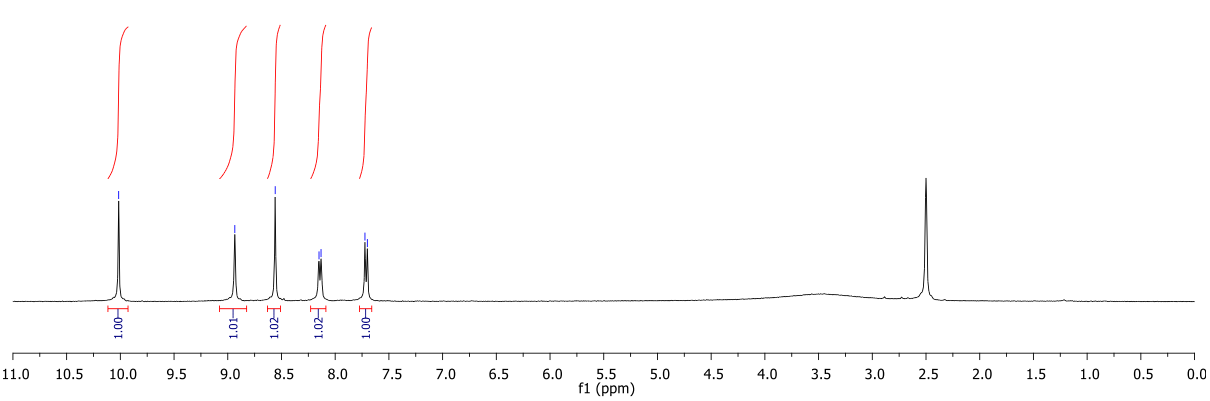

^{1}H NMR spectrum of 1g (400 MHz, DMSO-*d*$_6$)

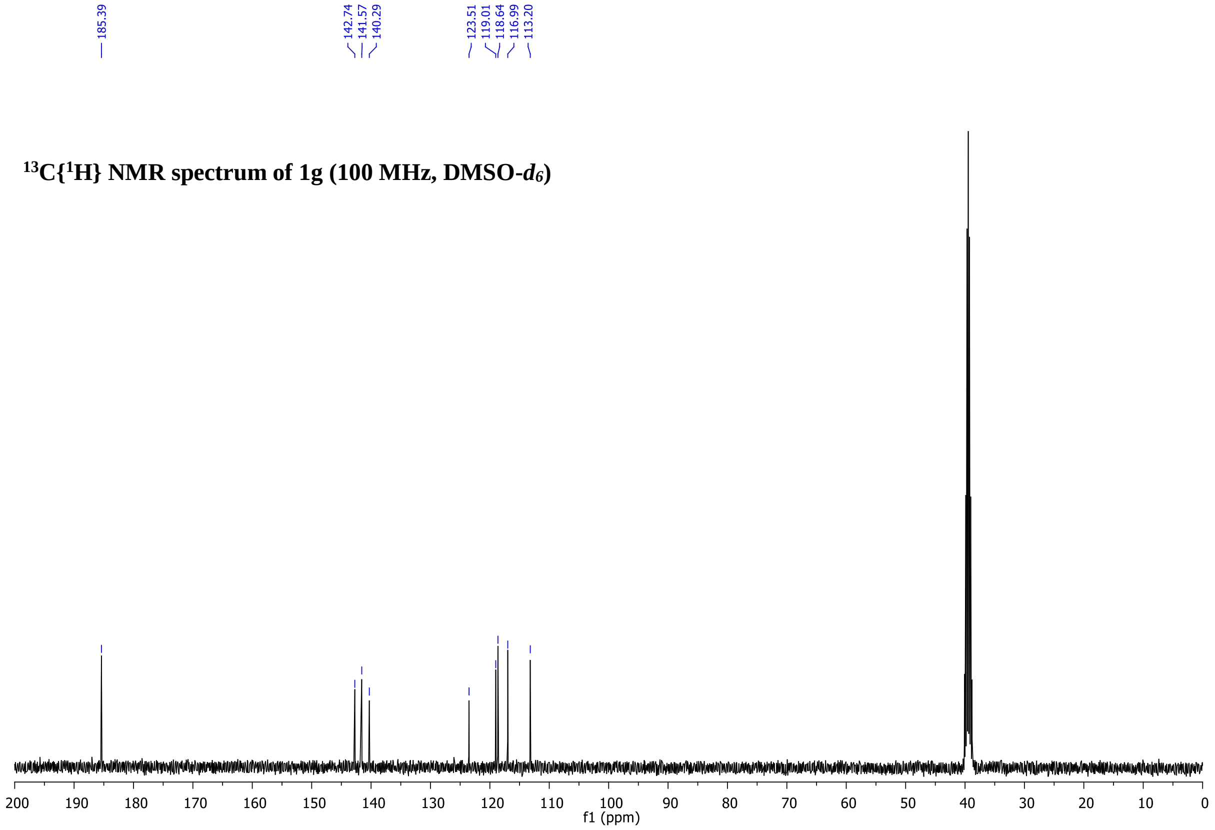

^{13}C{^{1}H} NMR spectrum of 1g (100 MHz, DMSO-*d*$_6$)

1-Benzyl-1*H*-indole-3-carbaldehyde (1h)

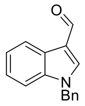

1h

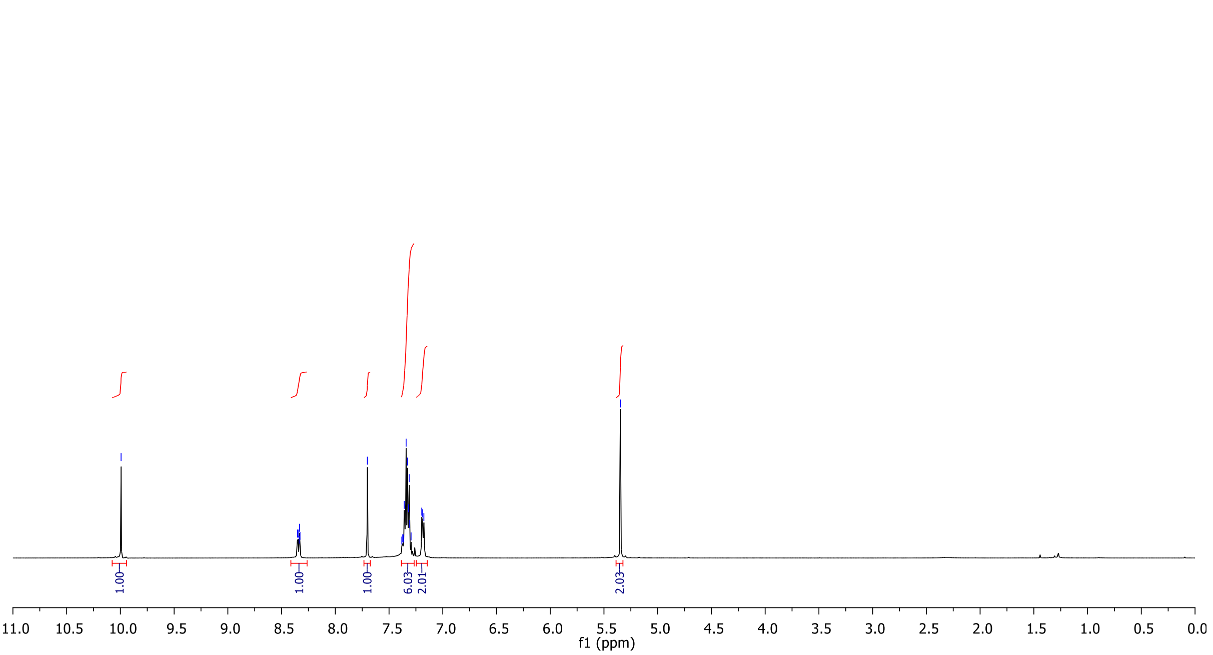

^{1}H NMR spectrum of 1h (400 MHz, $CDCl_3$)

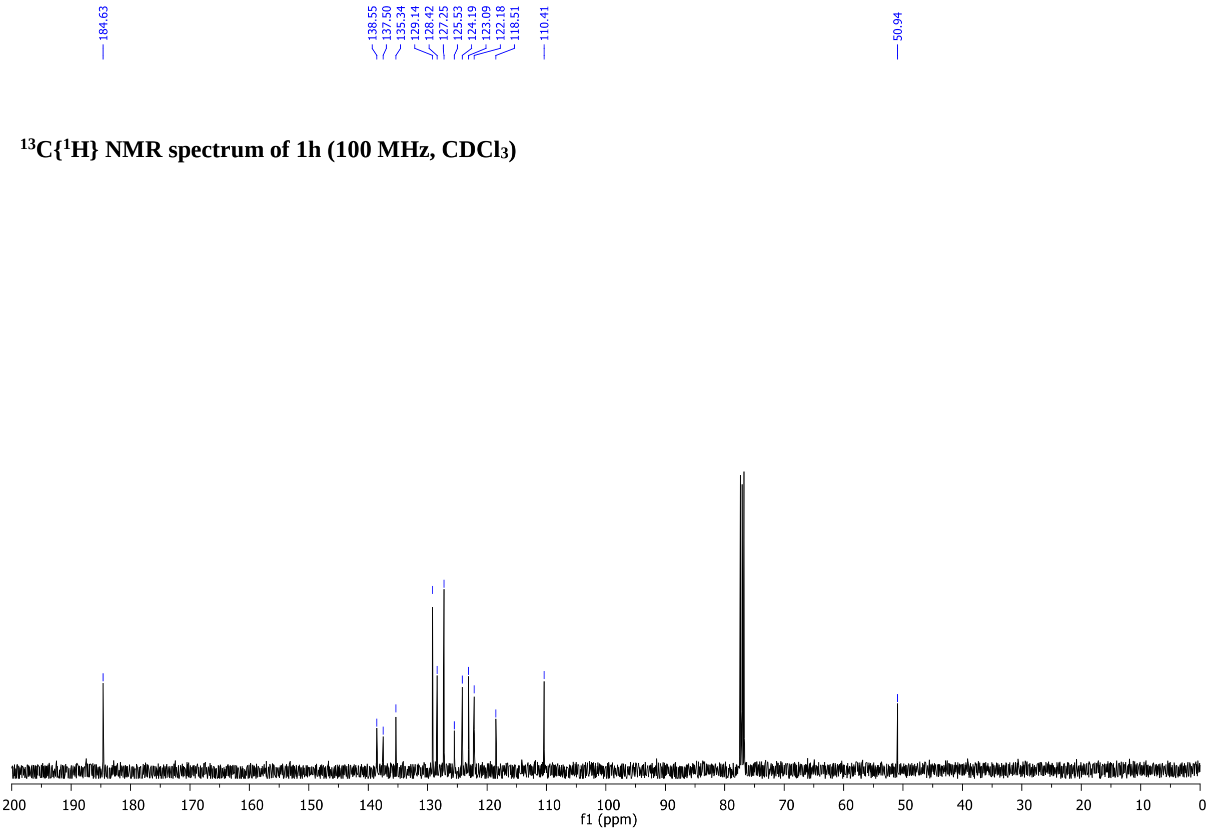

^{13}C{^{1}H} NMR spectrum of 1h (100 MHz, $CDCl_3$)

1-Methyl-1*H*-indole-3-carbaldehyde (1i)

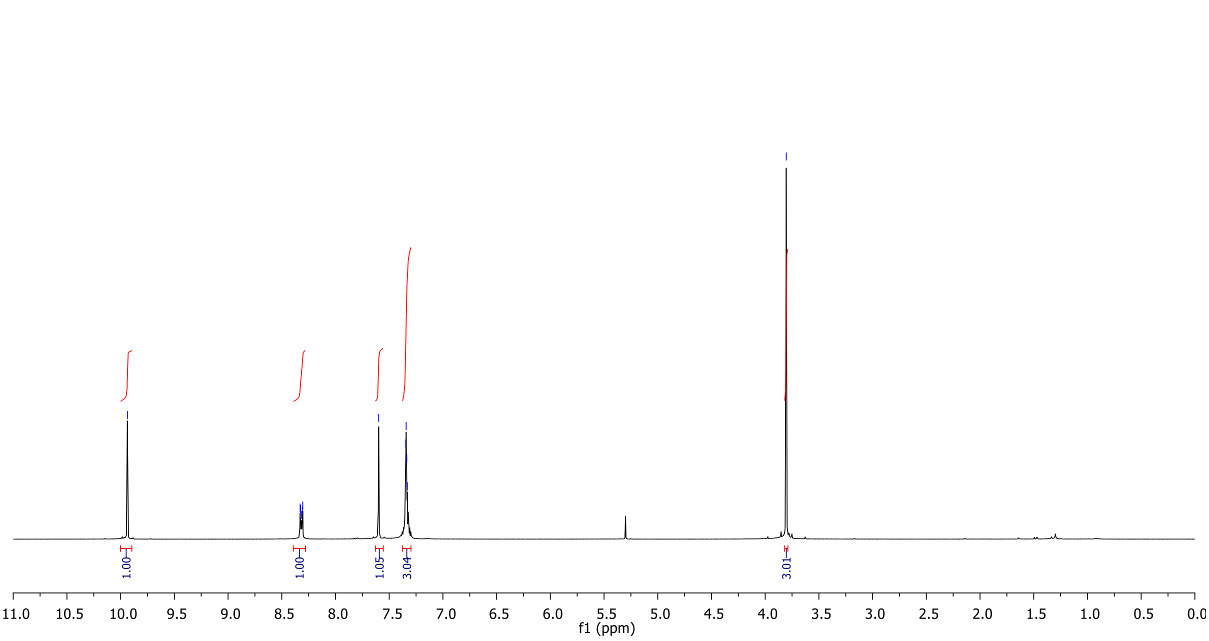

^{1}H NMR spectrum of 1i (400 MHz, $CDCl_3$)

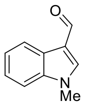

1i

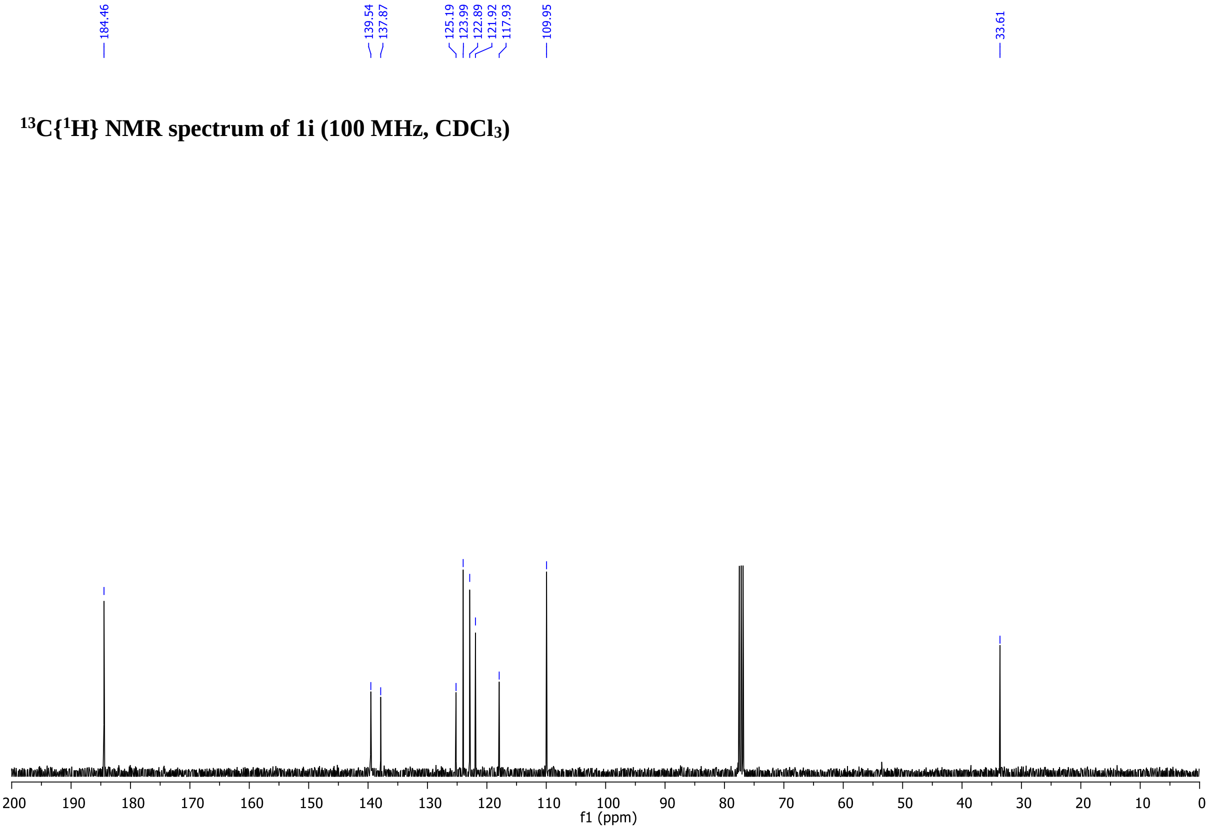

$^{13}C\{^{1}H\}$ NMR spectrum of 1i (100 MHz, $CDCl_3$)

1-Tosyl-1*H*-indole-3-carbaldehyde (1j)

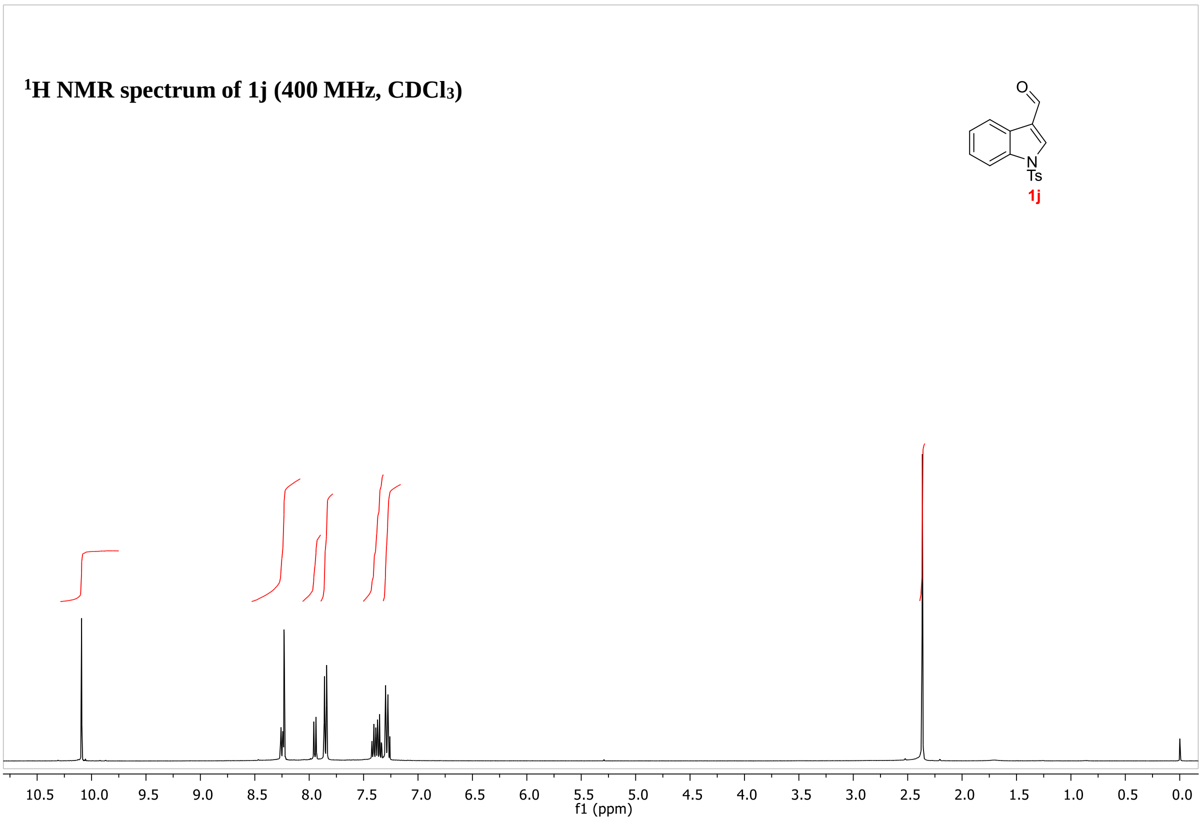

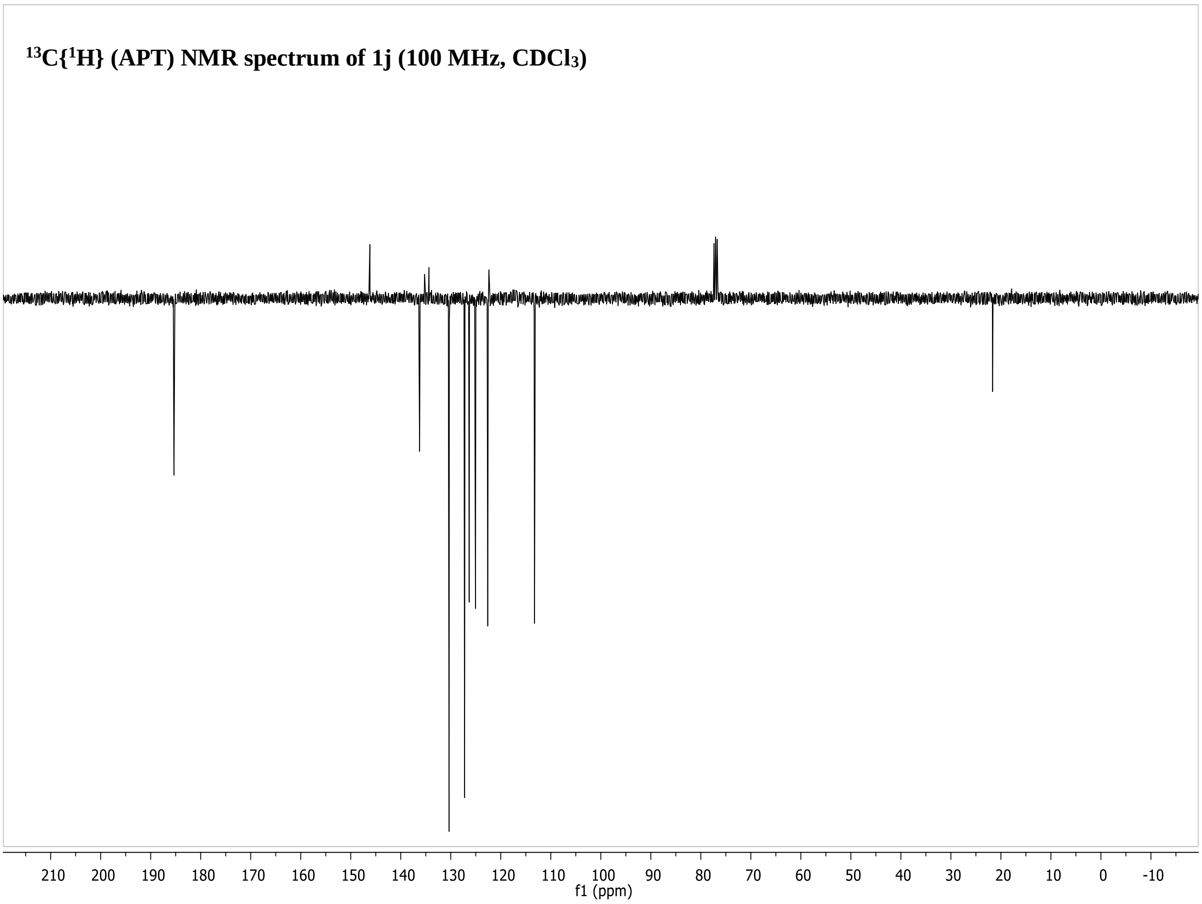

4-Phenyl-1*H*-indole-3-carbaldehyde (3aa)

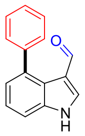

3aa

^{1}H NMR spectrum of 3aa (400 MHz, $CDCl_3$)

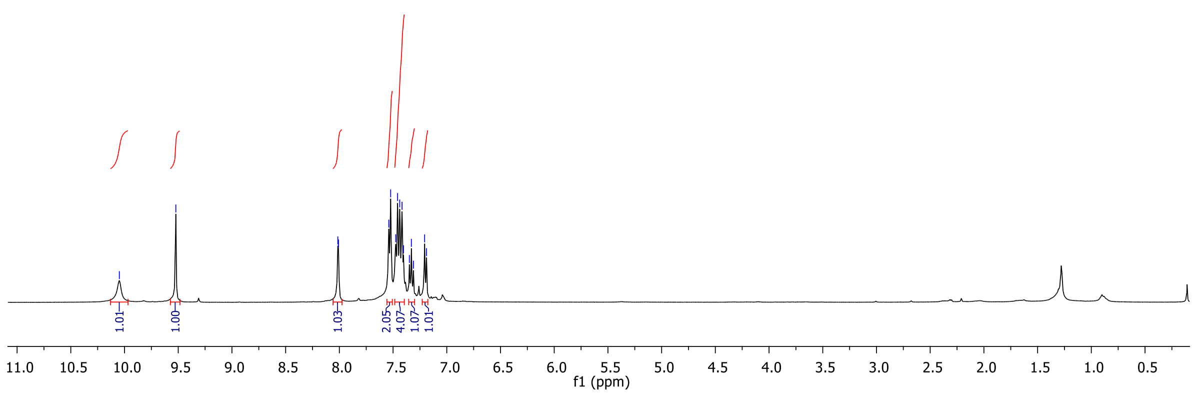

^{13}C{^{1}H} NMR spectrum of 3aa (100 MHz, $CDCl_3$)

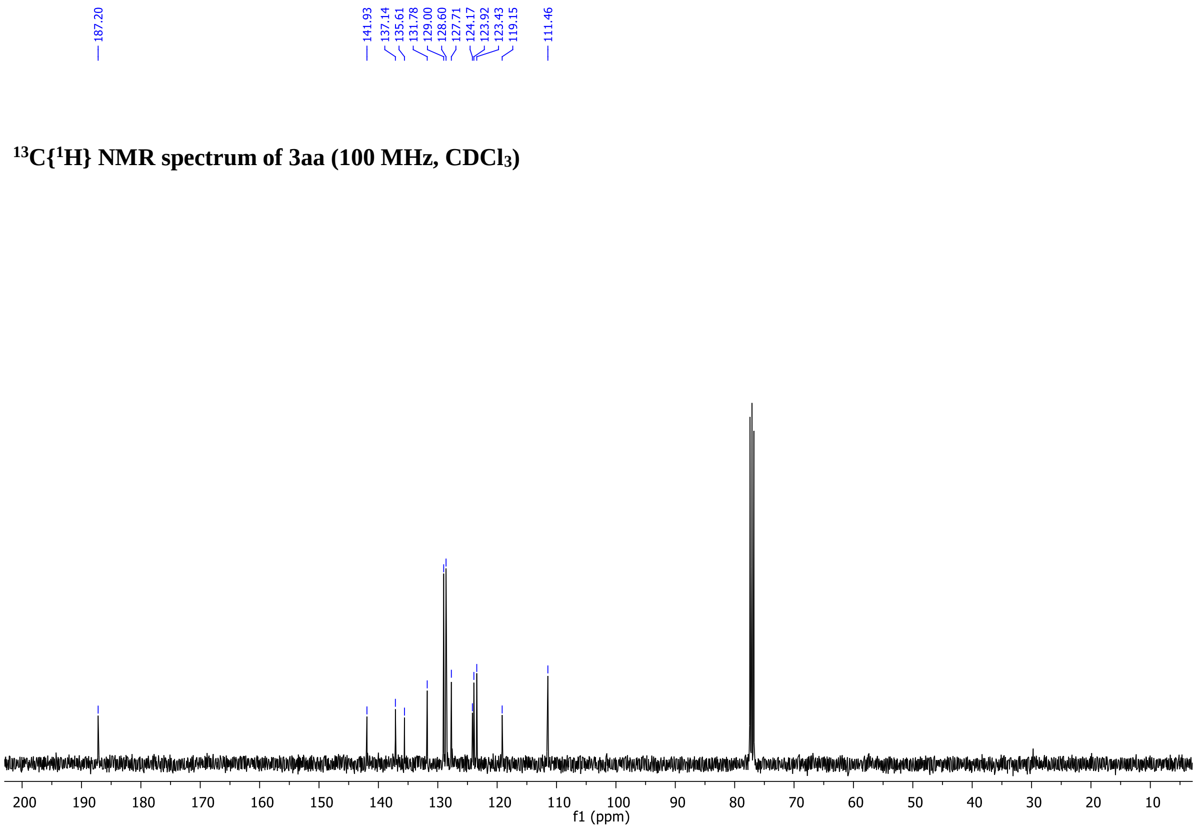

4-(*p*-Tolyl)-1*H*-indole-3-carbaldehyde (3ab)

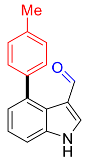

3ab

^{1}H NMR spectrum of 3ab (400 MHz, $CDCl_3$)

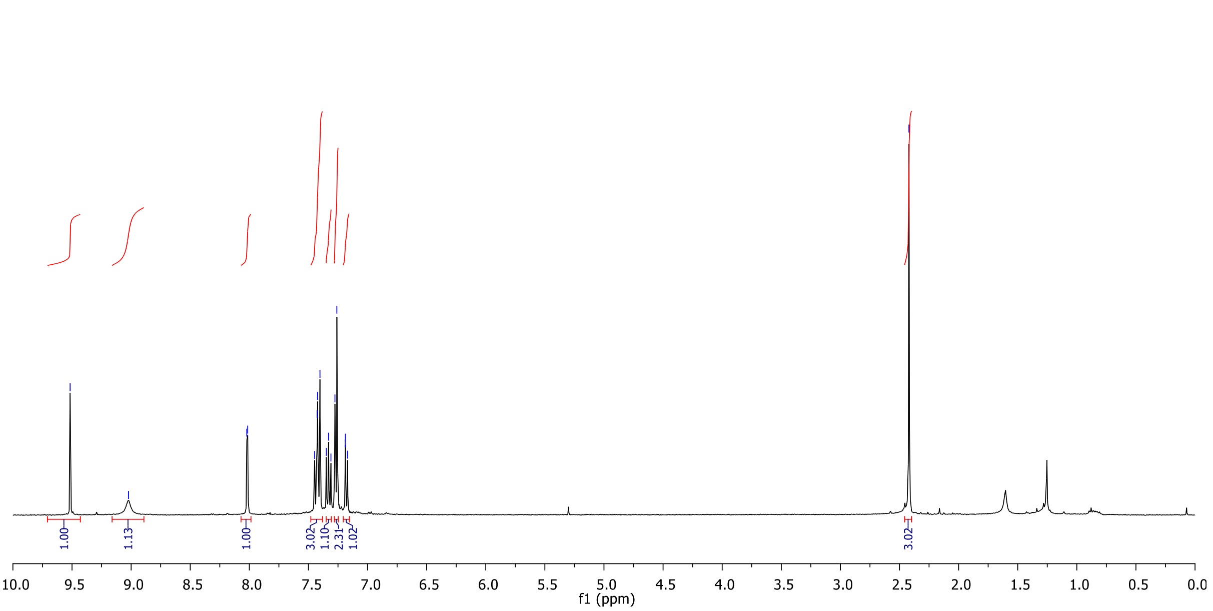

^{13}C{^{1}H} NMR spectrum of 3ab (100 MHz, $CDCl_3$)

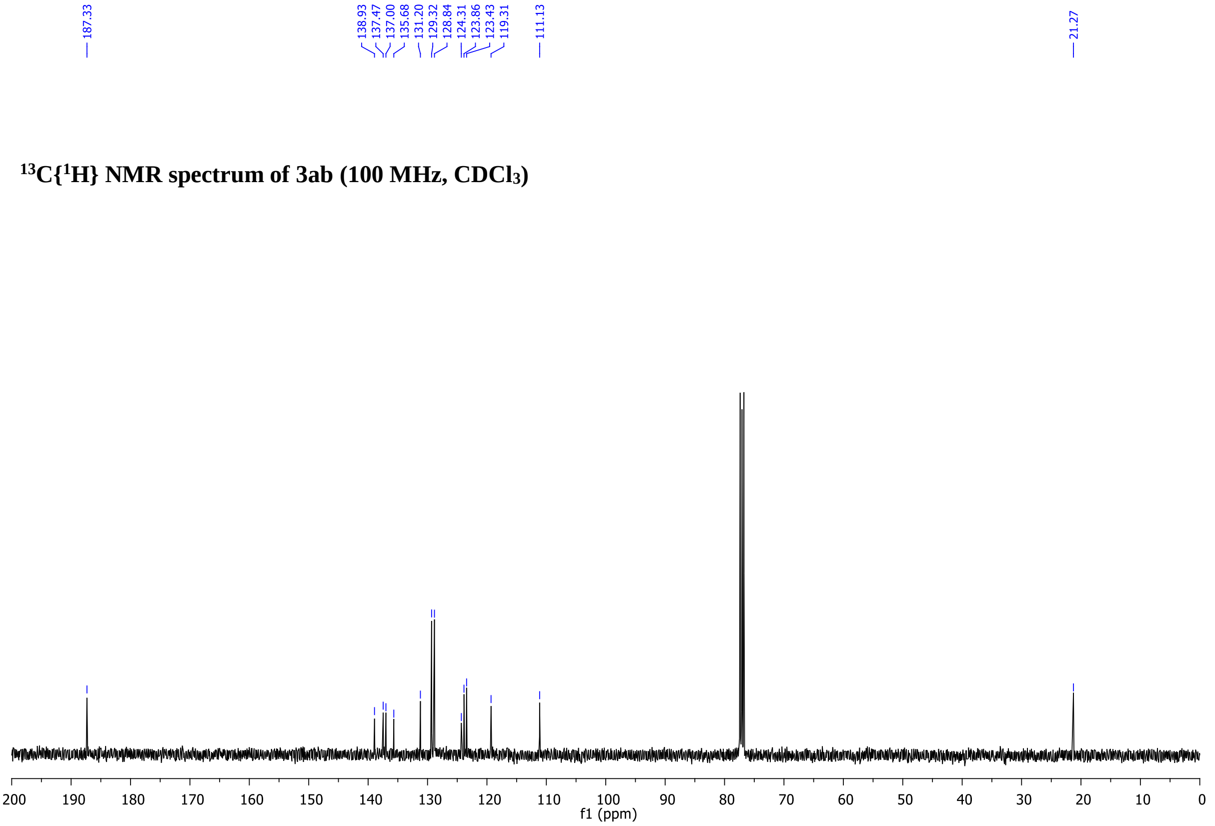

4-(*m*-Tolyl)-1*H*-indole-3-carbaldehyde (3ac)

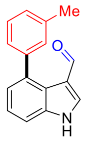

^{1}H NMR spectrum of 3ac (400 MHz, $CDCl_3$)

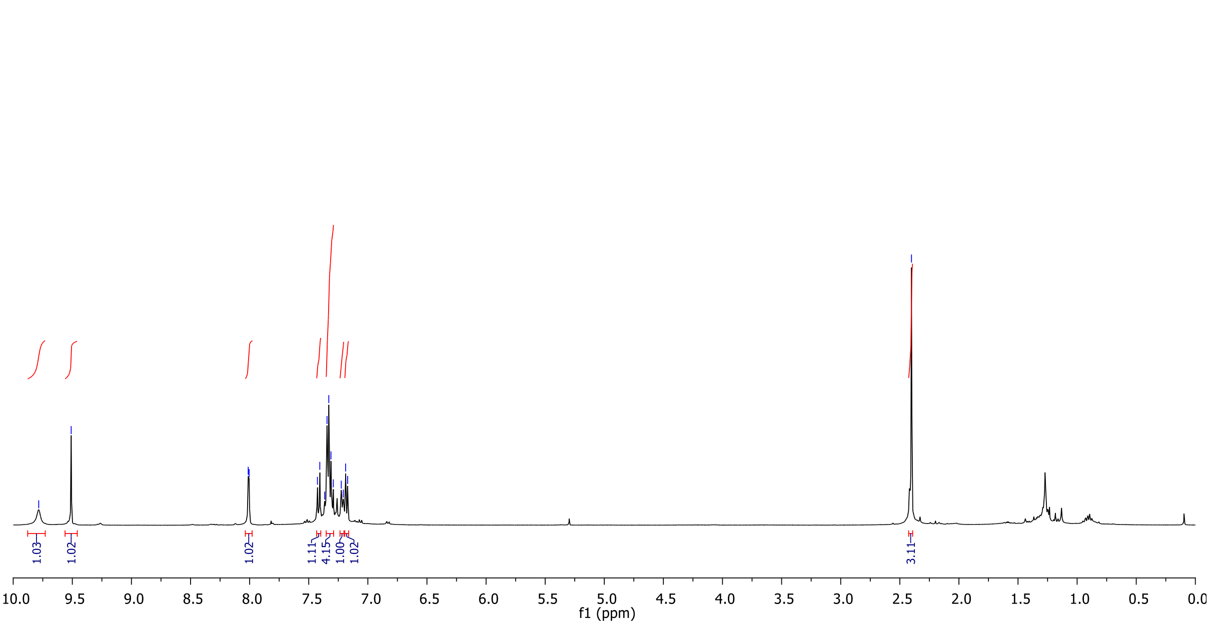

$^{13}C\{^1H\}$ NMR spectrum of 3ac (100 MHz, $CDCl_3$)

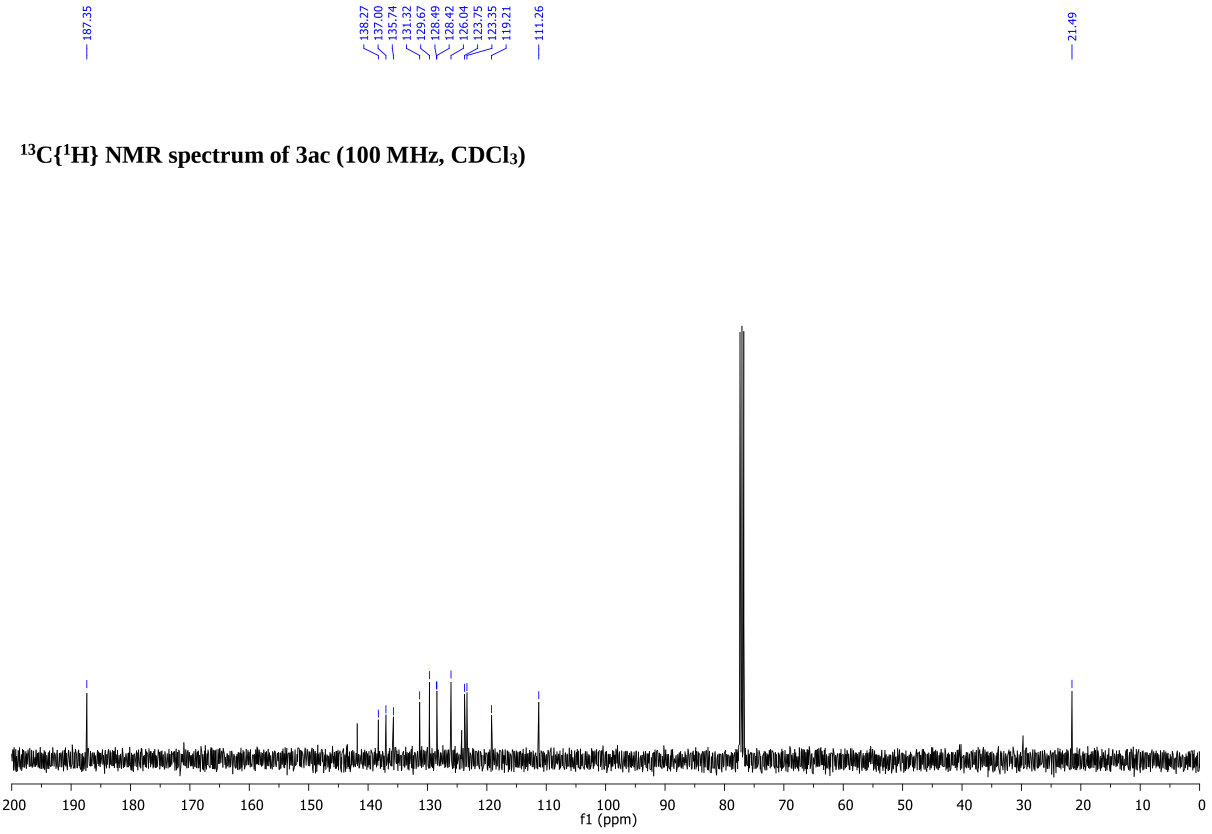

4-(4-(*tert*-Butyl)phenyl)-1*H*-indole-3-carbaldehyde (3ad)

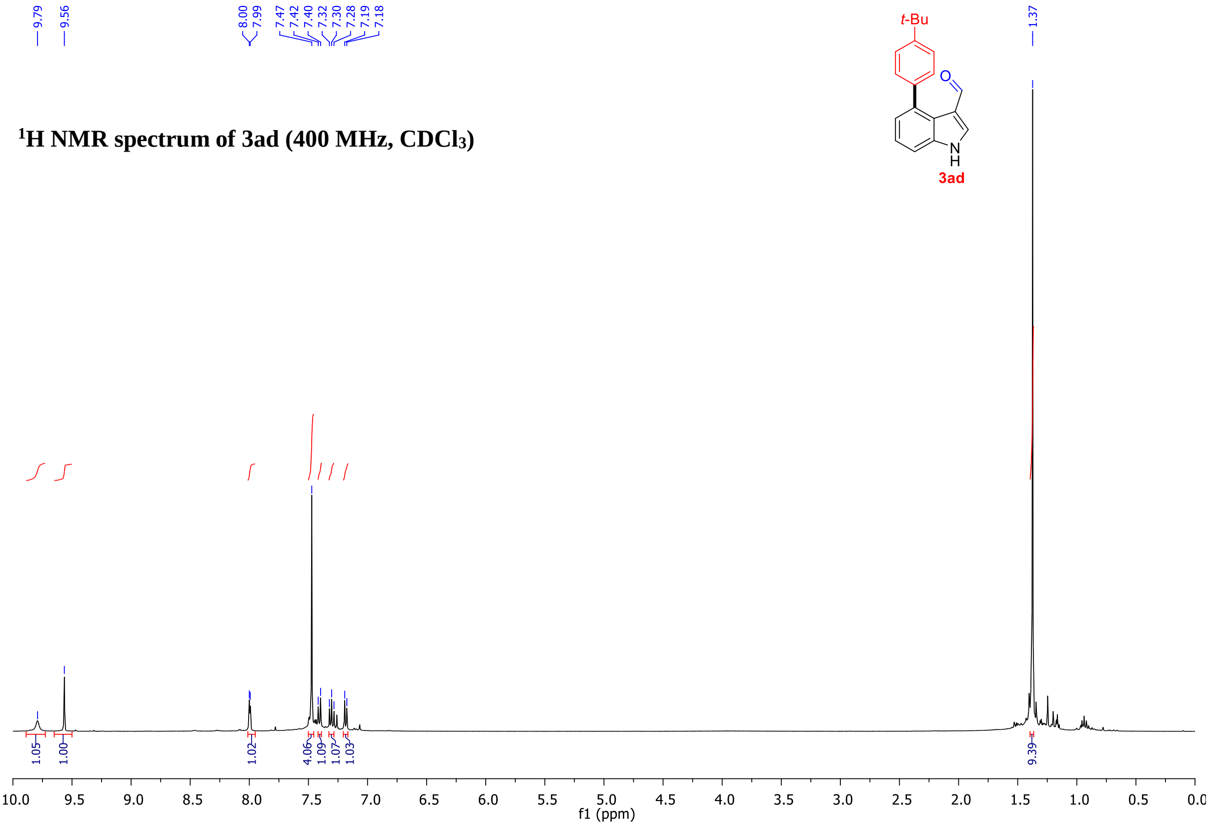

^{1}H NMR spectrum of 3ad (400 MHz, $CDCl_3$)

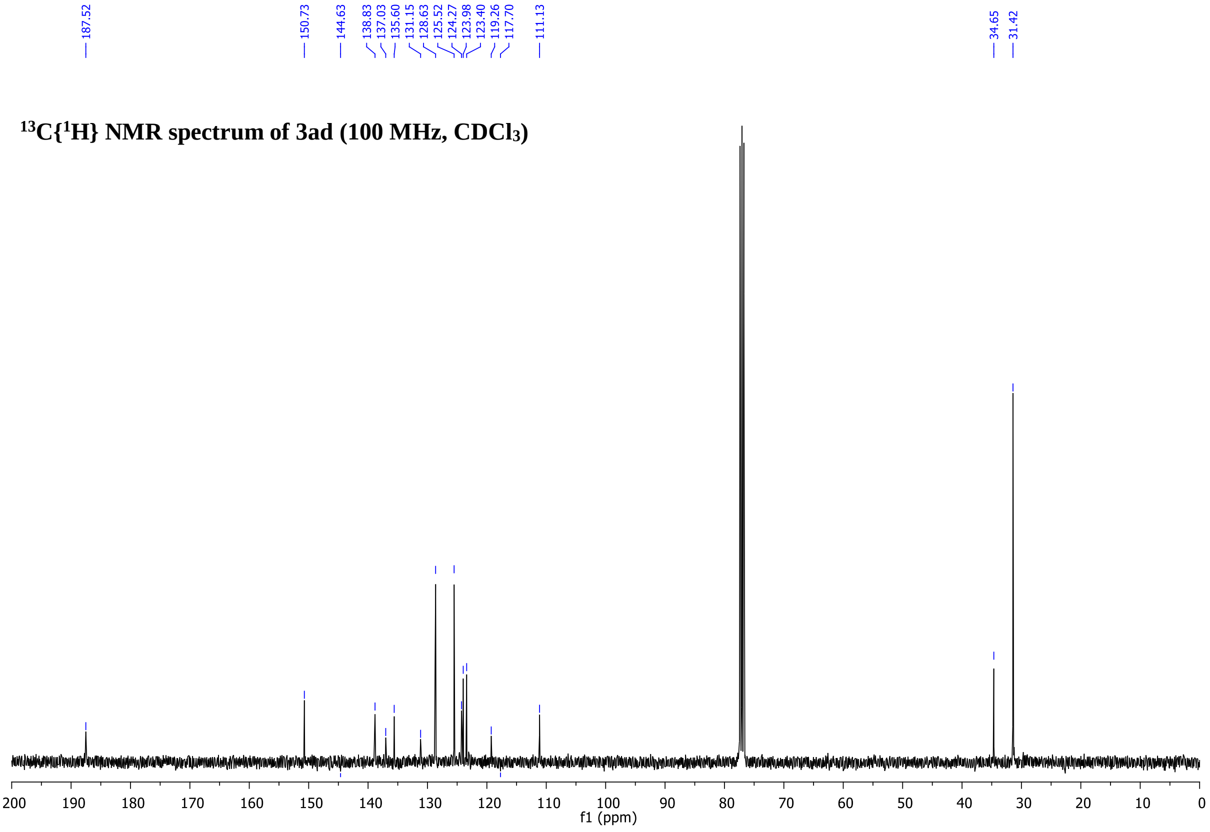

^{13}C{^{1}H} NMR spectrum of 3ad (100 MHz, $CDCl_3$)

4-(4-Bromophenyl)-1*H*-indole-3-carbaldehyde (3ae)

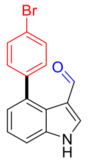

^{1}H NMR spectrum of 3ae (400 MHz, $CDCl_3$)

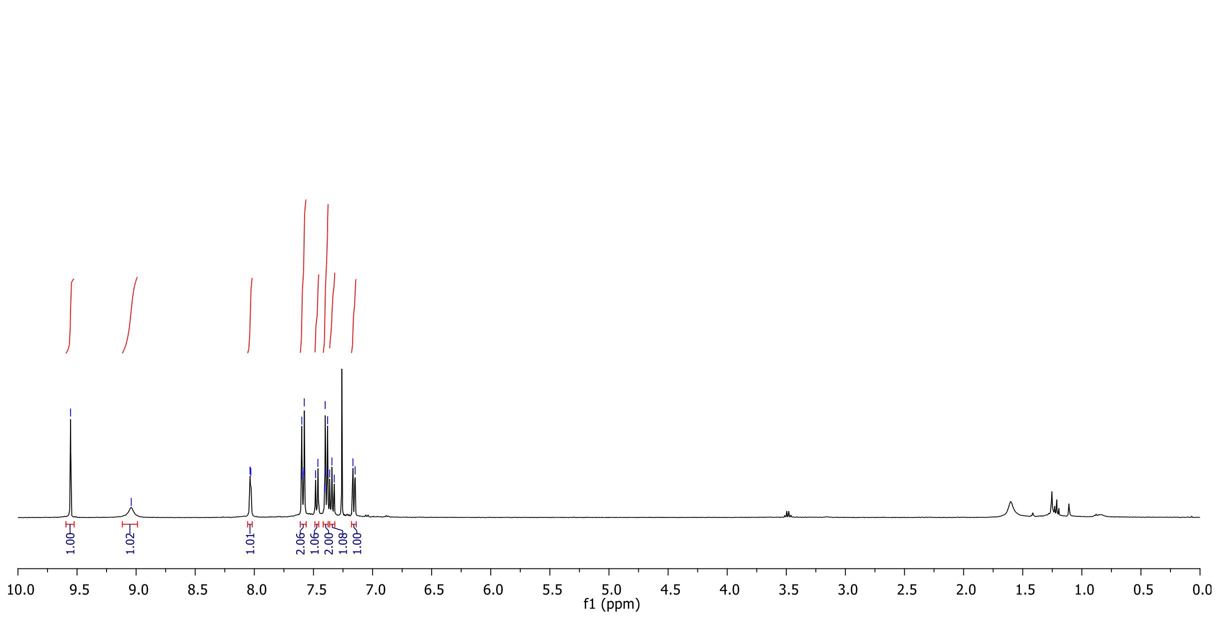

^{13}C{^{1}H} NMR spectrum of 3ae (100 MHz, $CDCl_3$)

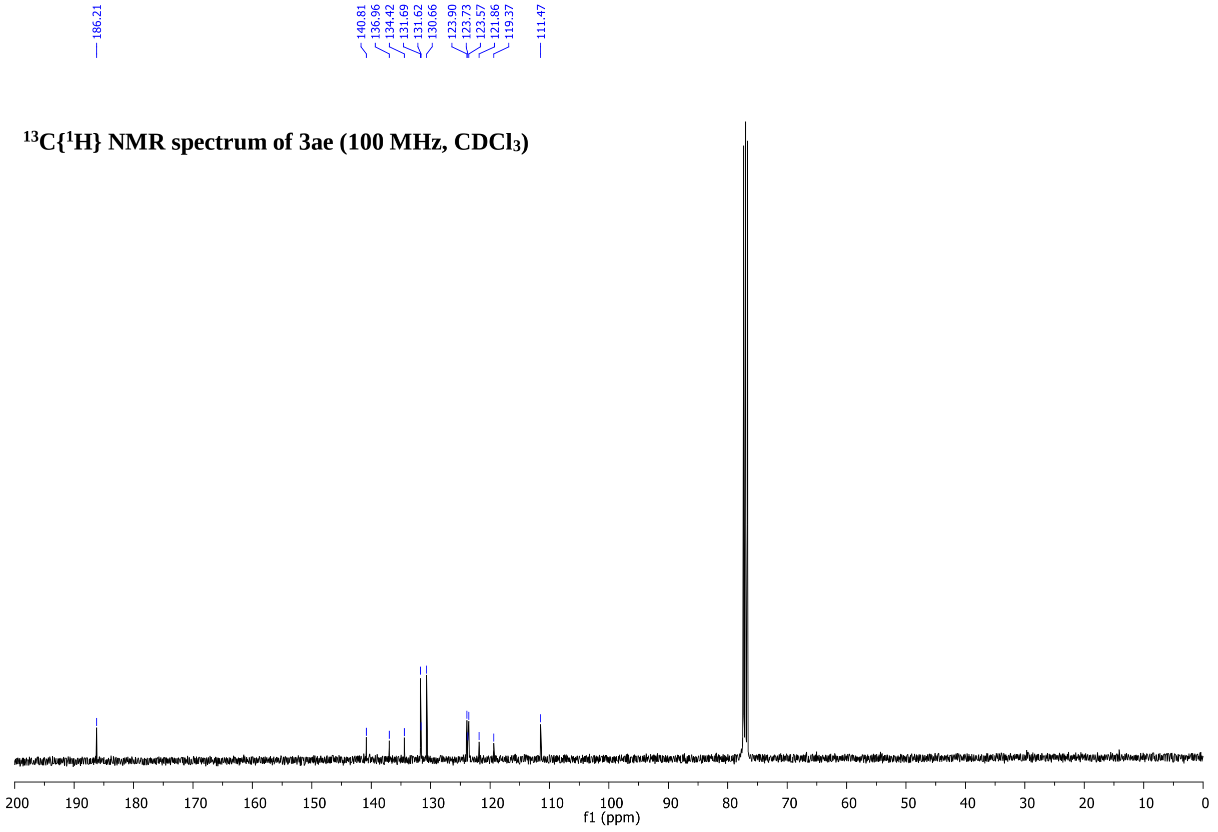

4-(4-Methoxyphenyl)-1*H*-indole-3-carbaldehyde (3af)

^{1}H NMR spectrum of 3af (400 MHz, $CDCl_3$)

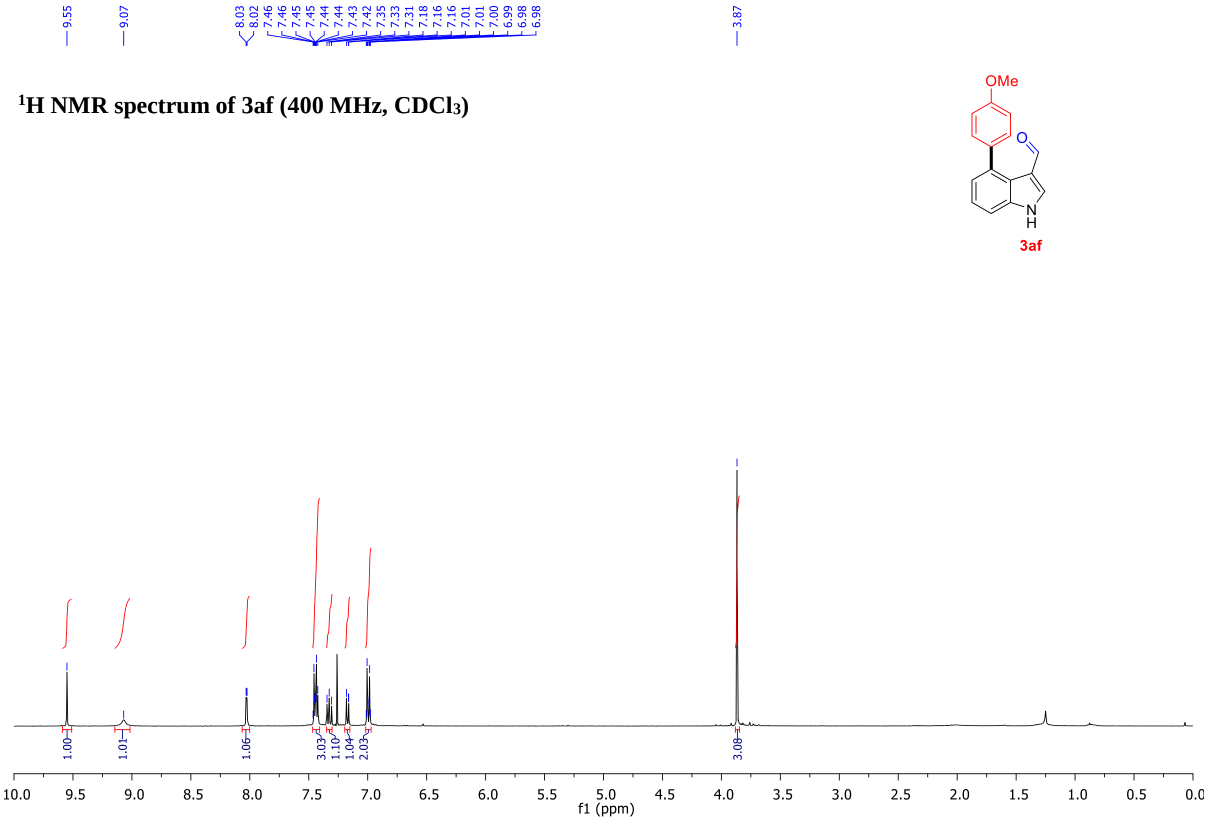

^{13}C{^{1}H} NMR spectrum of 3af (100 MHz, $CDCl_3$)

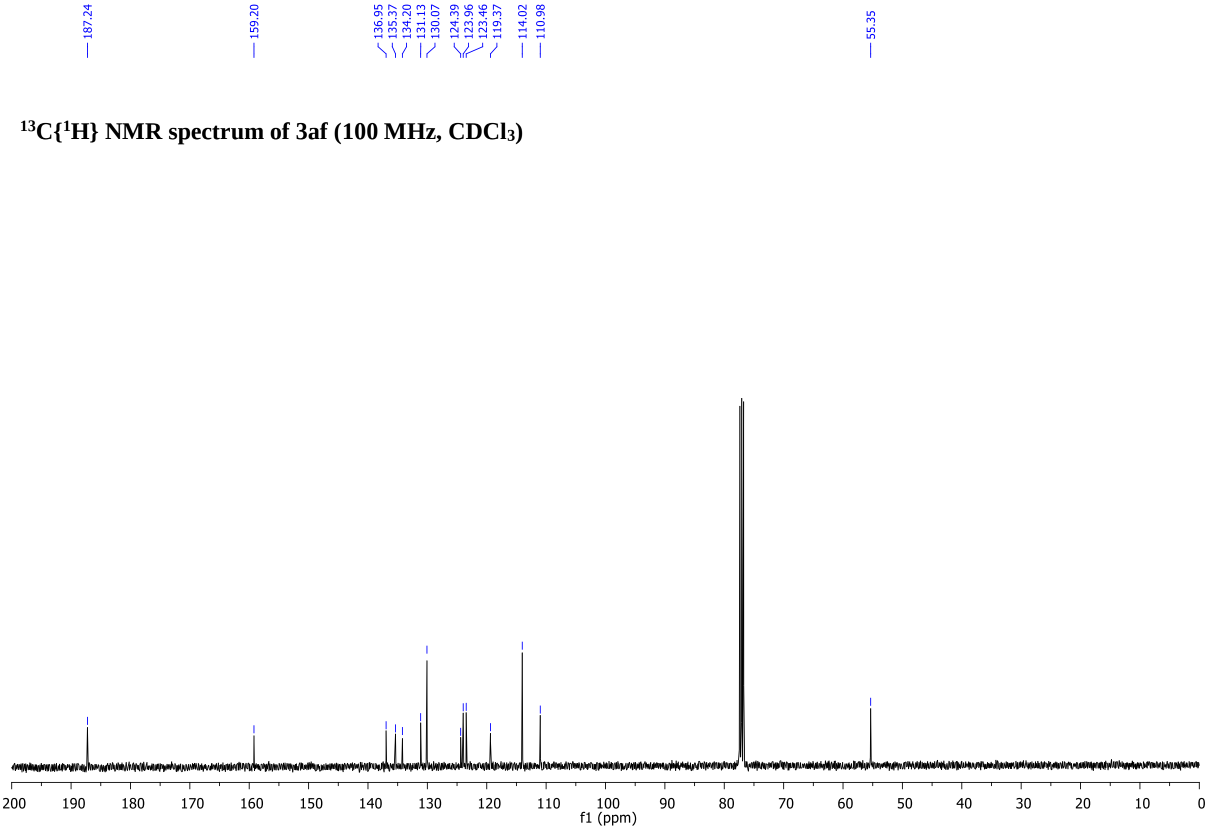

Methyl 4-(3-formyl-1*H*-indol-4-yl)benzoate (3ag)

^{1}H NMR spectrum of 3ag (400 MHz, Acetone-*d*$_6$)

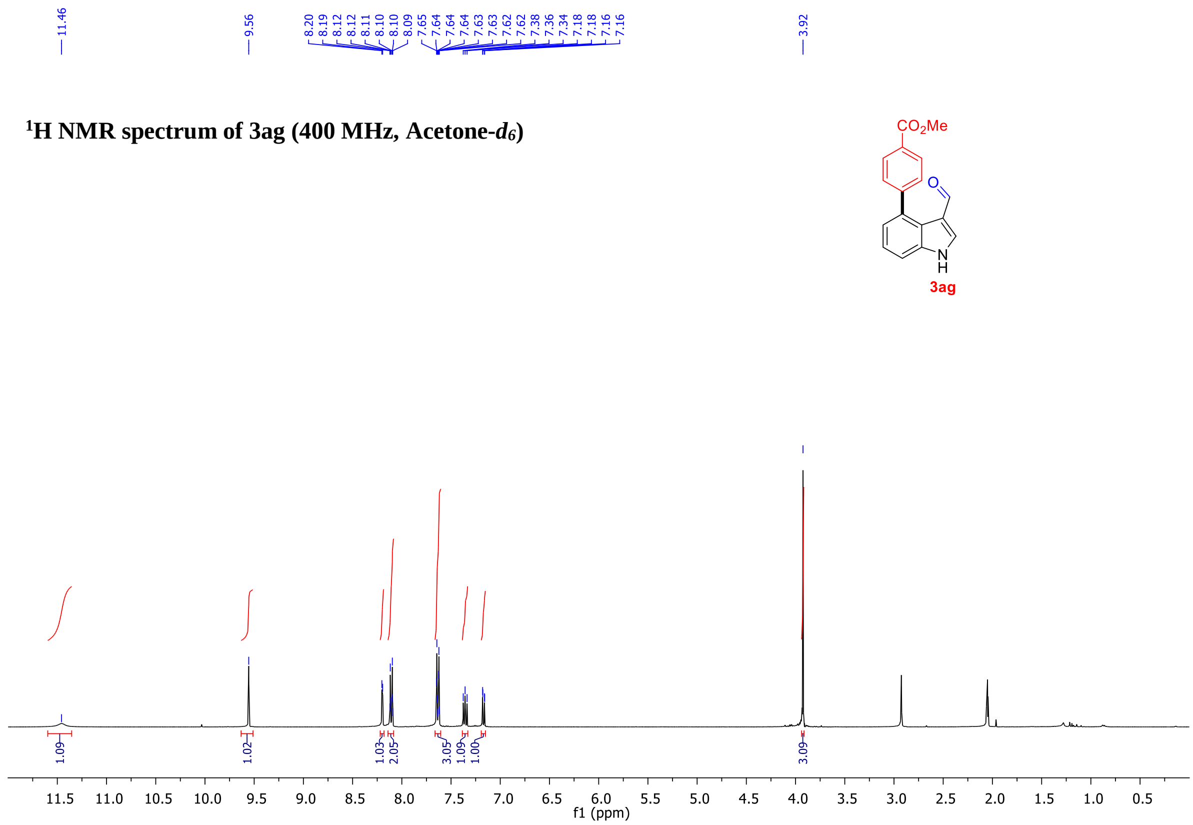

3ag

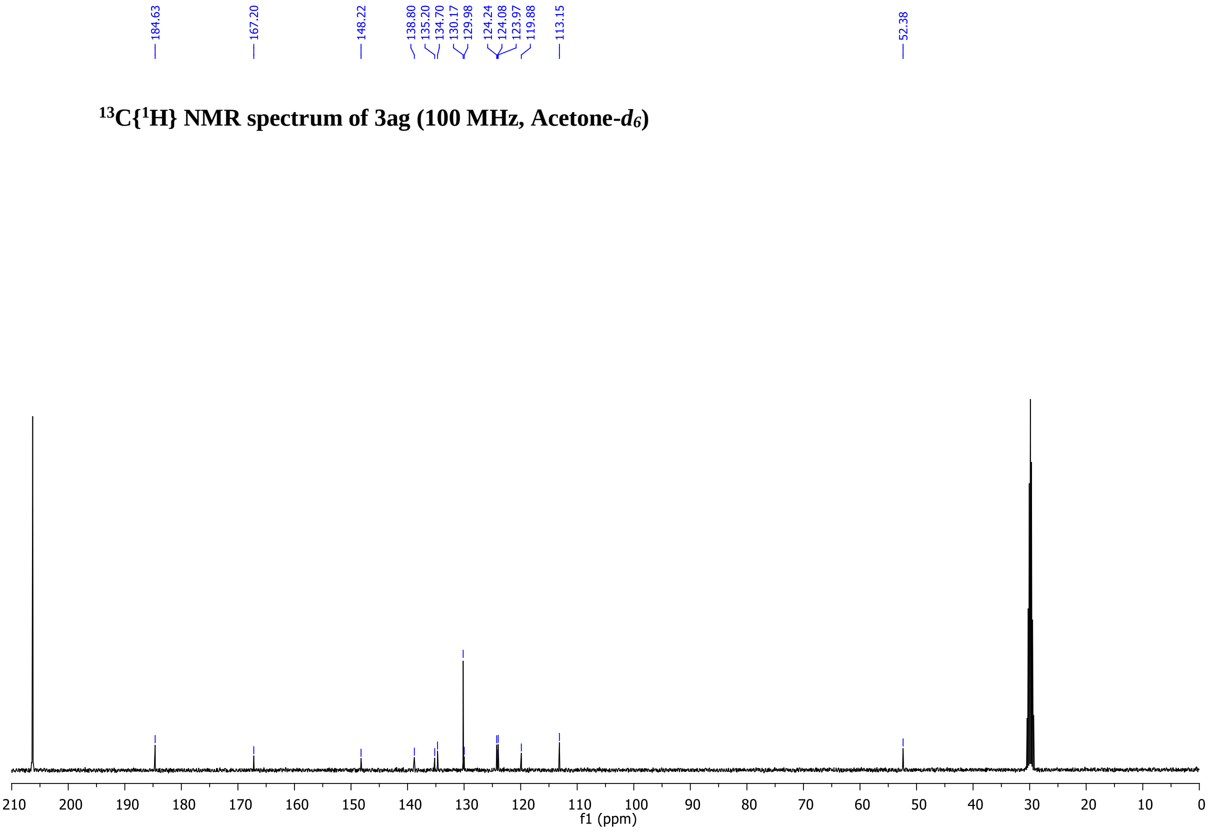

^{13}C{^{1}H} NMR spectrum of 3ag (100 MHz, Acetone-*d*$_6$)

4-(4-Acetylphenyl)-1*H*-indole-3-carbaldehyde (3ah)

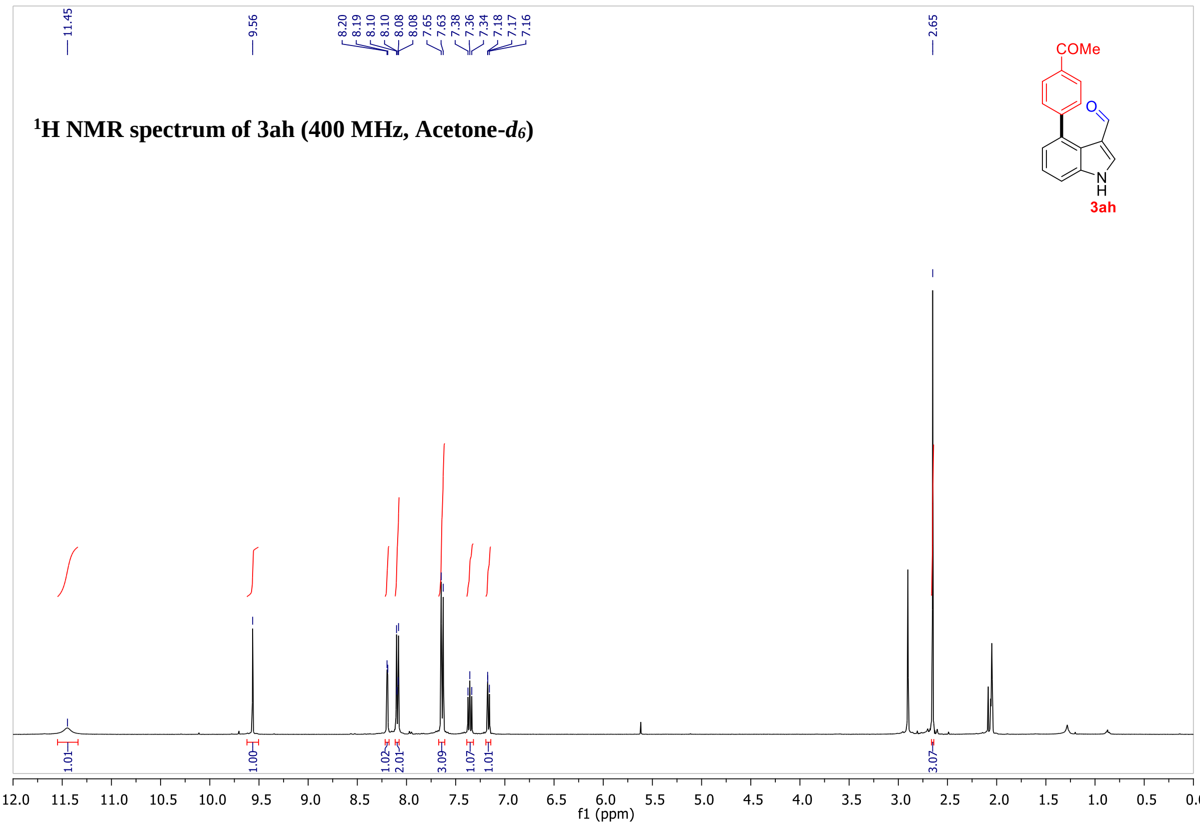

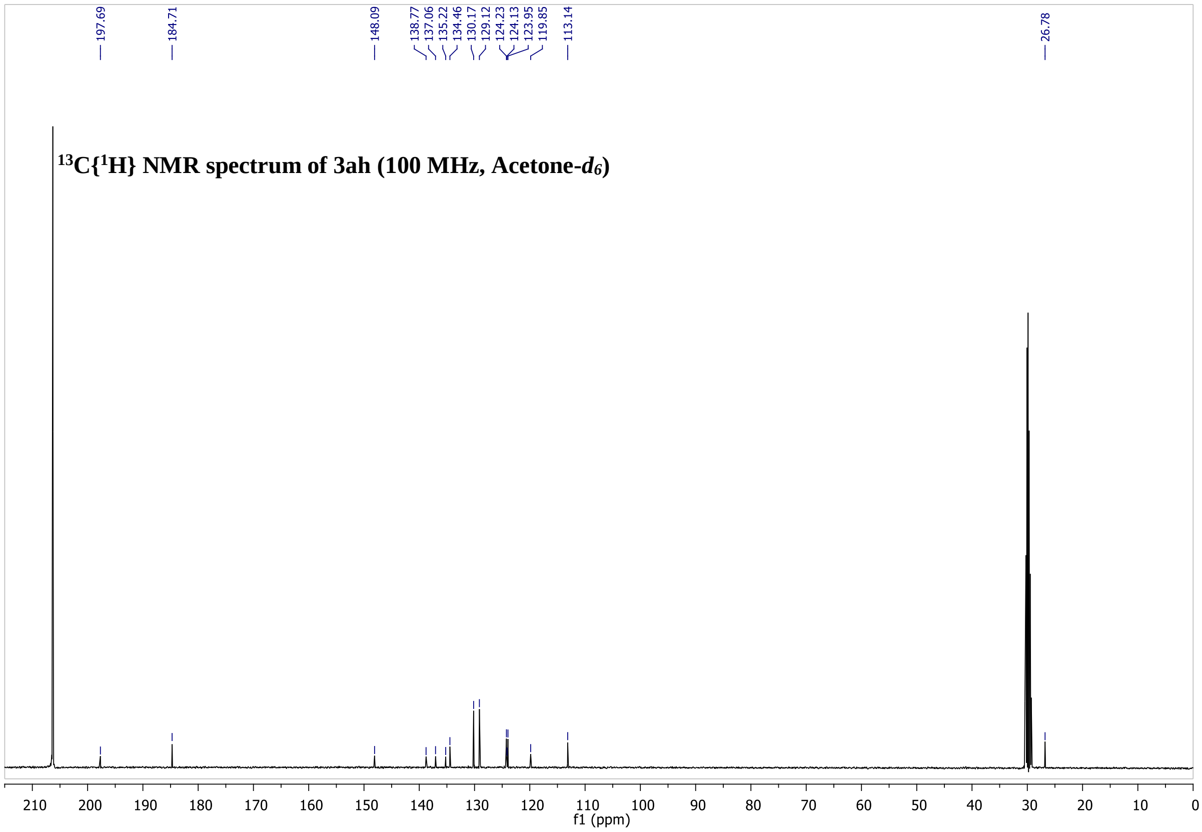

4-(4-(Trifluoromethyl)phenyl)-1*H*-indole-3-carbaldehyde (3ai)

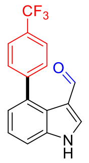

3ai

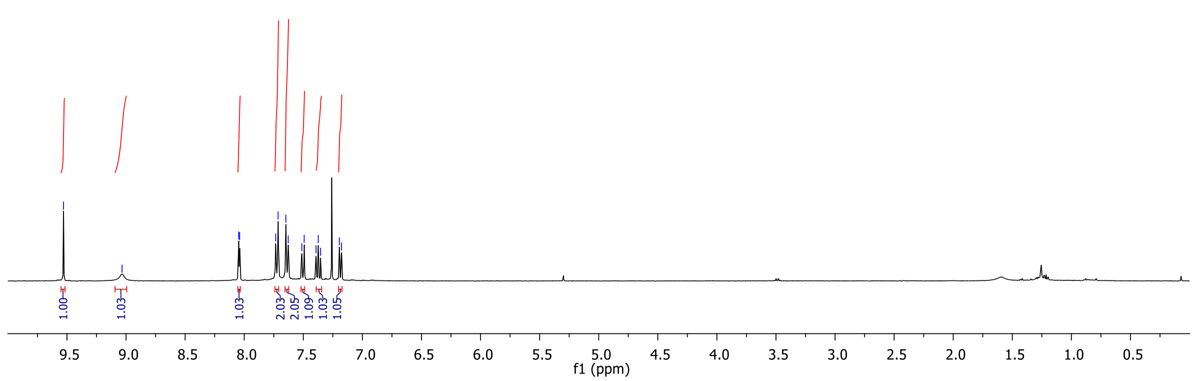

^{1}H NMR spectrum of 3ai (400 MHz, $CDCl_3$)

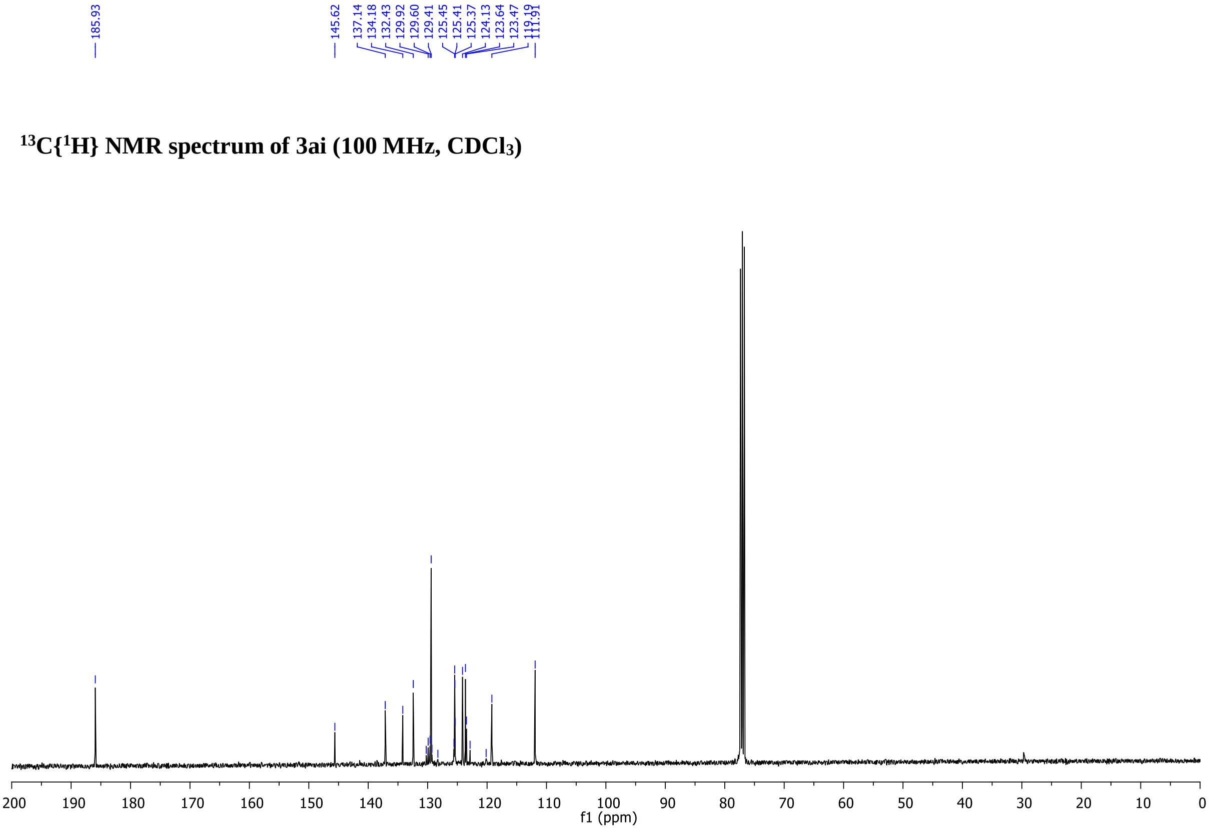

^{13}C{^{1}H} NMR spectrum of 3ai (100 MHz, $CDCl_3$)

4-(3-Nitrophenyl)-1*H*-indole-3-carbaldehyde (3al)

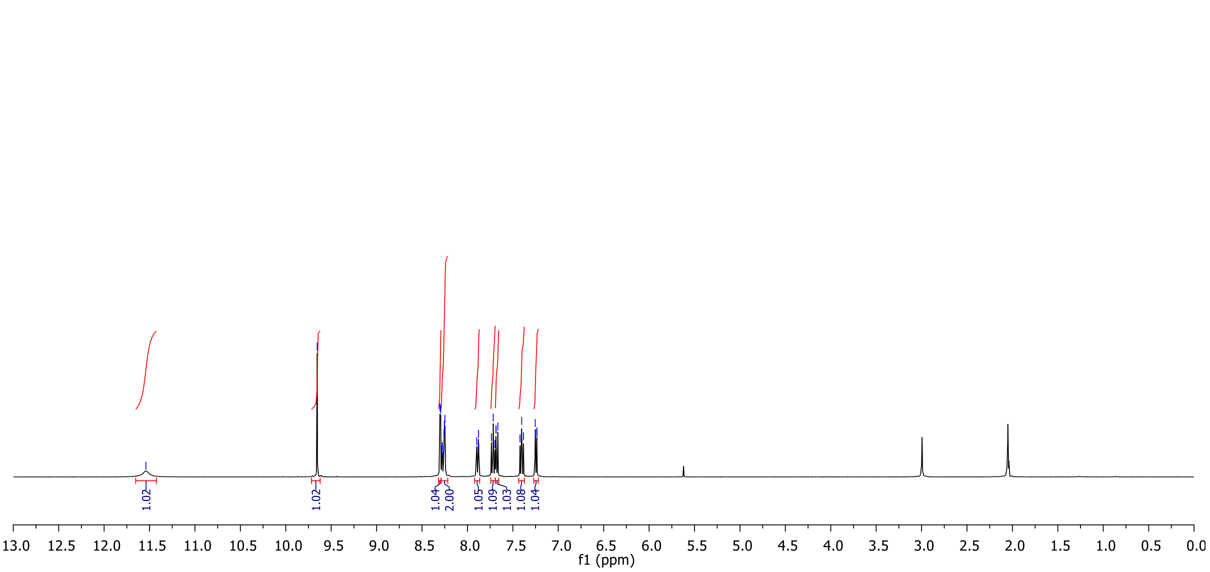

^{1}H NMR spectrum of 3al (400 MHz, Acetone-d_6)

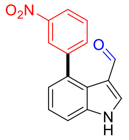

3al

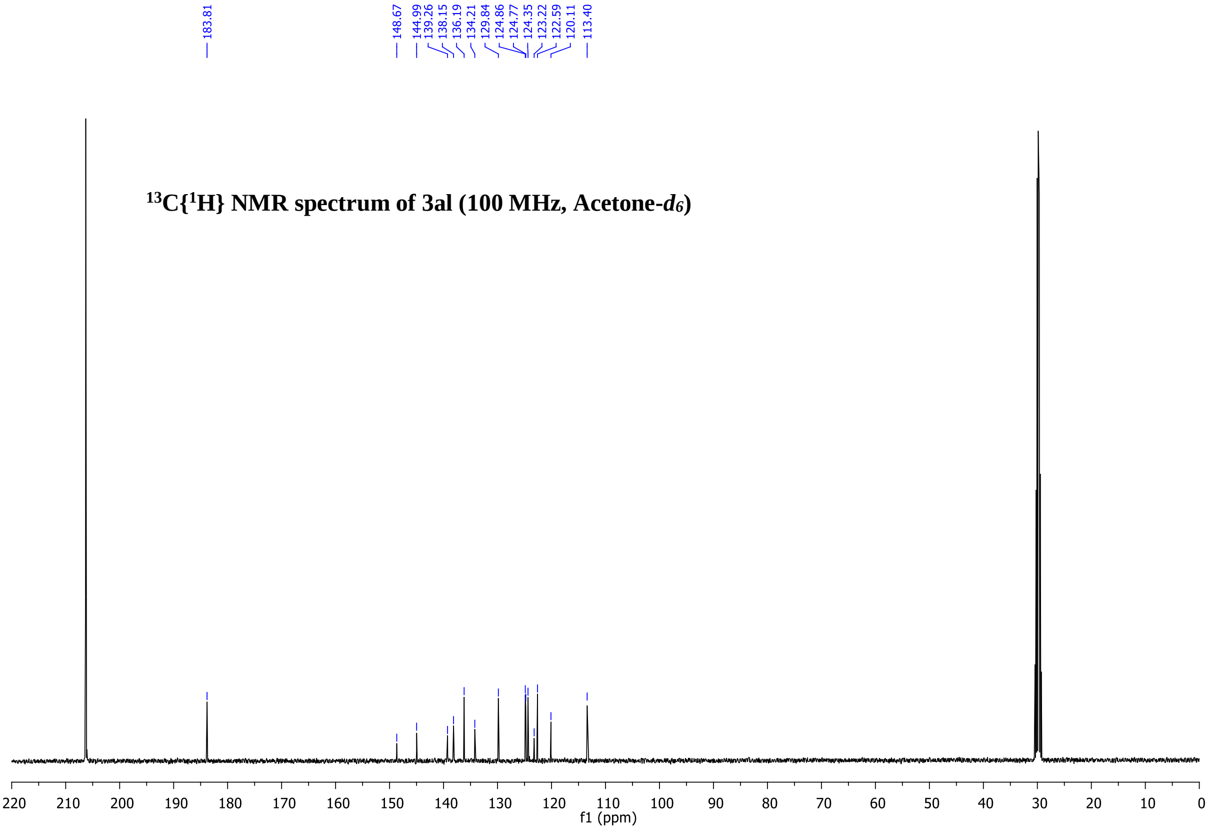

^{13}C{^{1}H} NMR spectrum of 3al (100 MHz, Acetone-d_6)

7-Fluoro-4-phenyl-1*H*-indole-3-carbaldehyde (3ba)

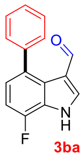

3ba

^{1}H NMR spectrum of 3ba (400 MHz, $CDCl_3$)

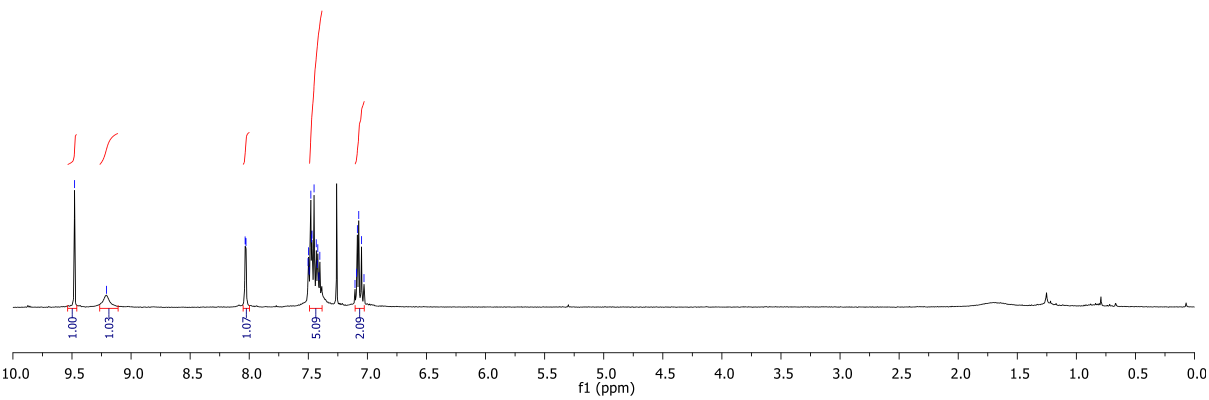

^{13}C{^{1}H} NMR spectrum of 3ba (100 MHz, $CDCl_3$)

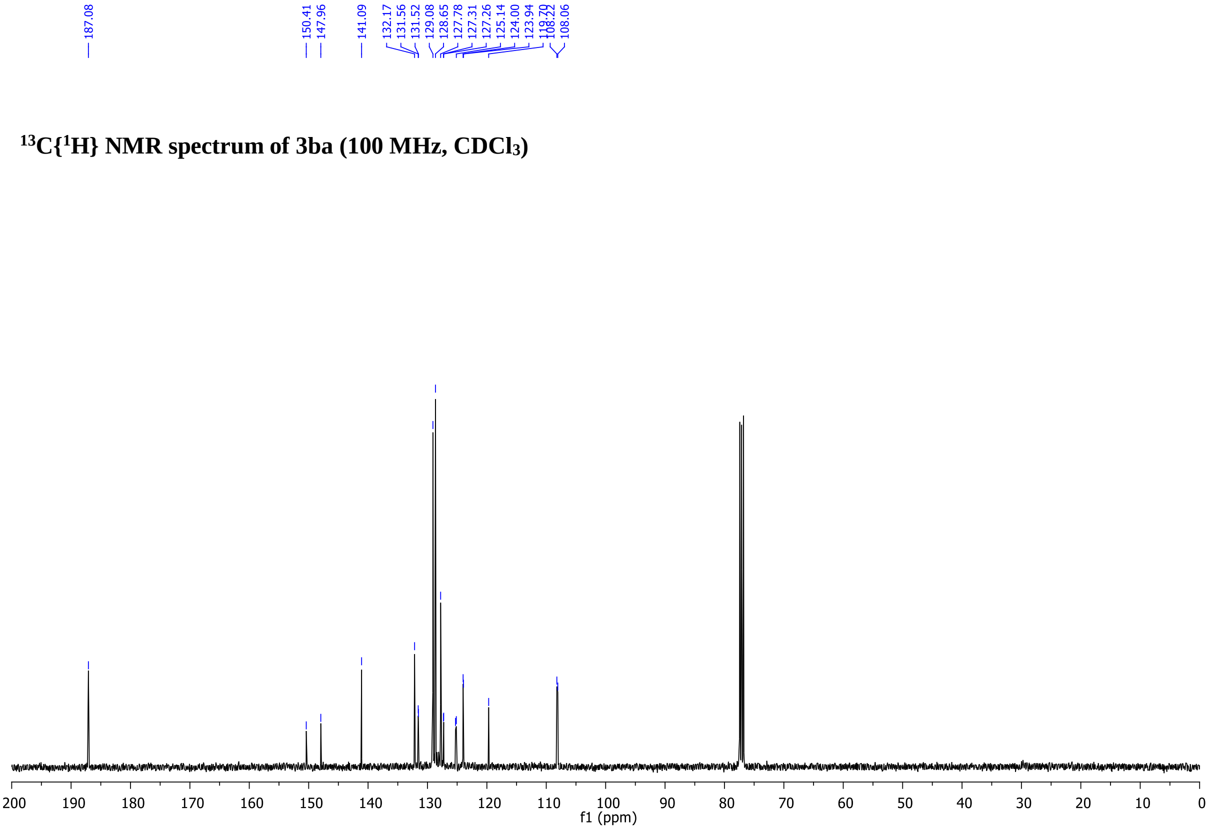

7-Bromo-4-phenyl-1*H*-indole-3-carbaldehyde (3ca)

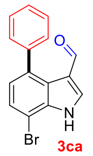

^{1}H NMR spectrum of 3ca (400 MHz, $CDCl_3$)

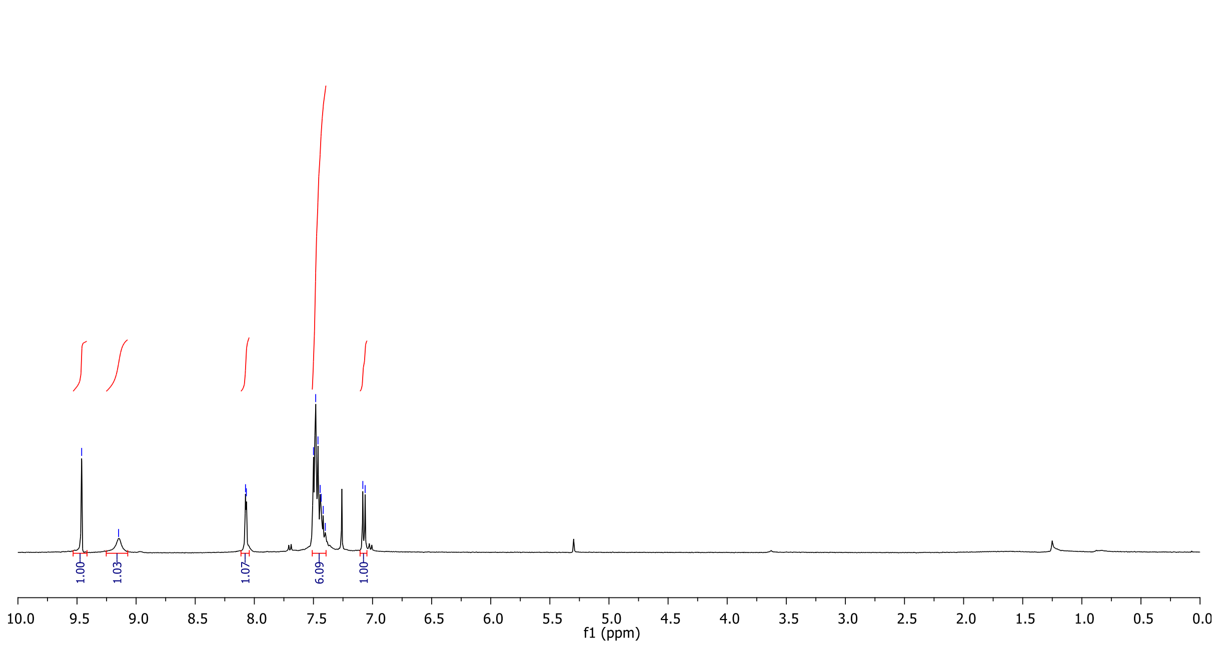

^{13}C{^{1}H} NMR spectrum of 3ca (100 MHz, $CDCl_3$)

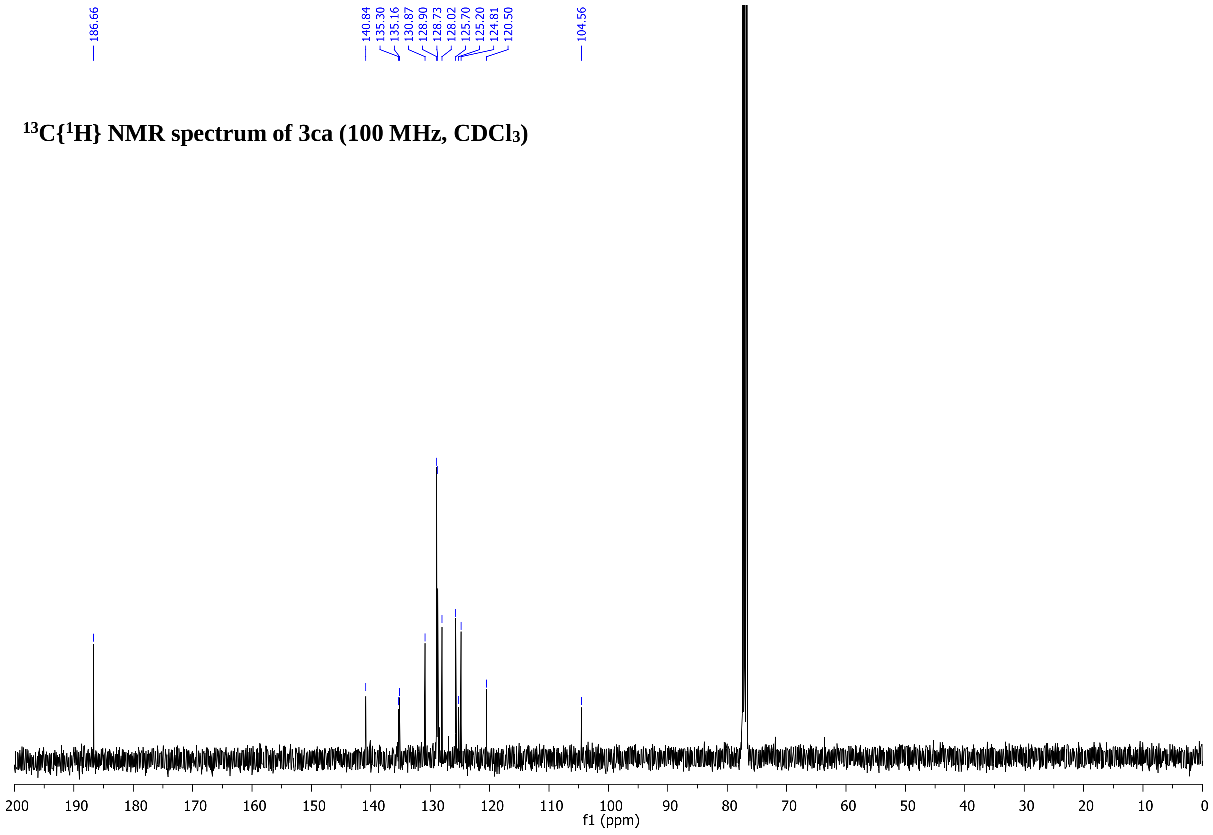

2-Methyl-4-phenyl-1*H*-indole-3-carbaldehyde (3da)

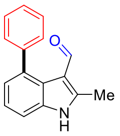

3da

^{1}H NMR spectrum of 3da (400 MHz, $CDCl_3$)

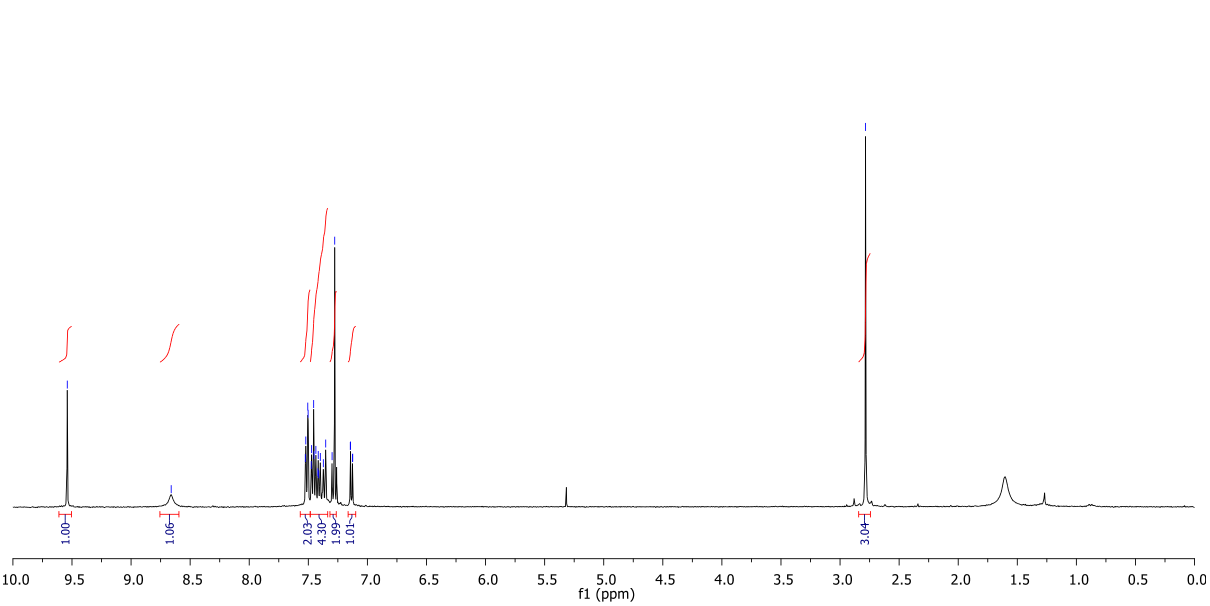

^{13}C{^{1}H} NMR spectrum of 3da (100 MHz, $CDCl_3$)

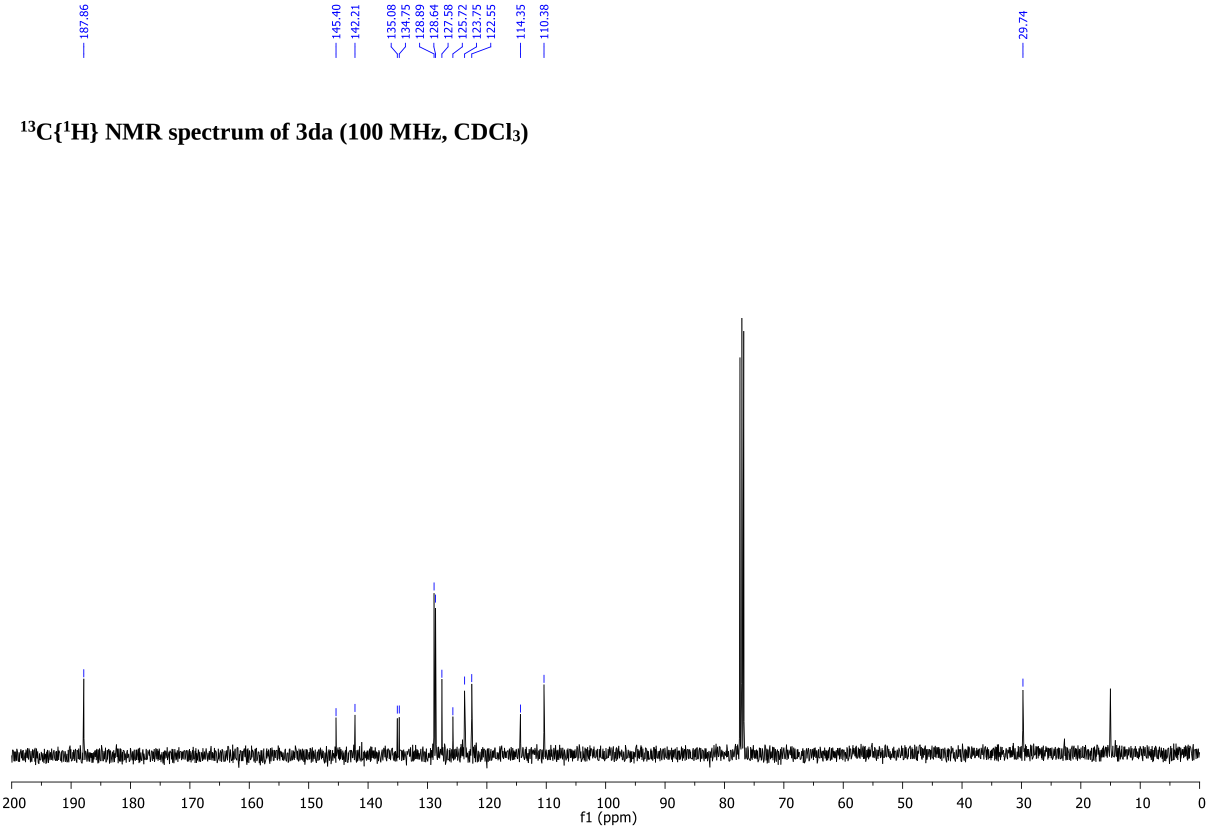

1-Benzyl-4-phenyl-1*H*-indole-3-carbaldehyde (3ha)

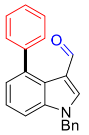

3ha

^{1}H NMR spectrum of 3ha (400 MHz, $CDCl_3$)

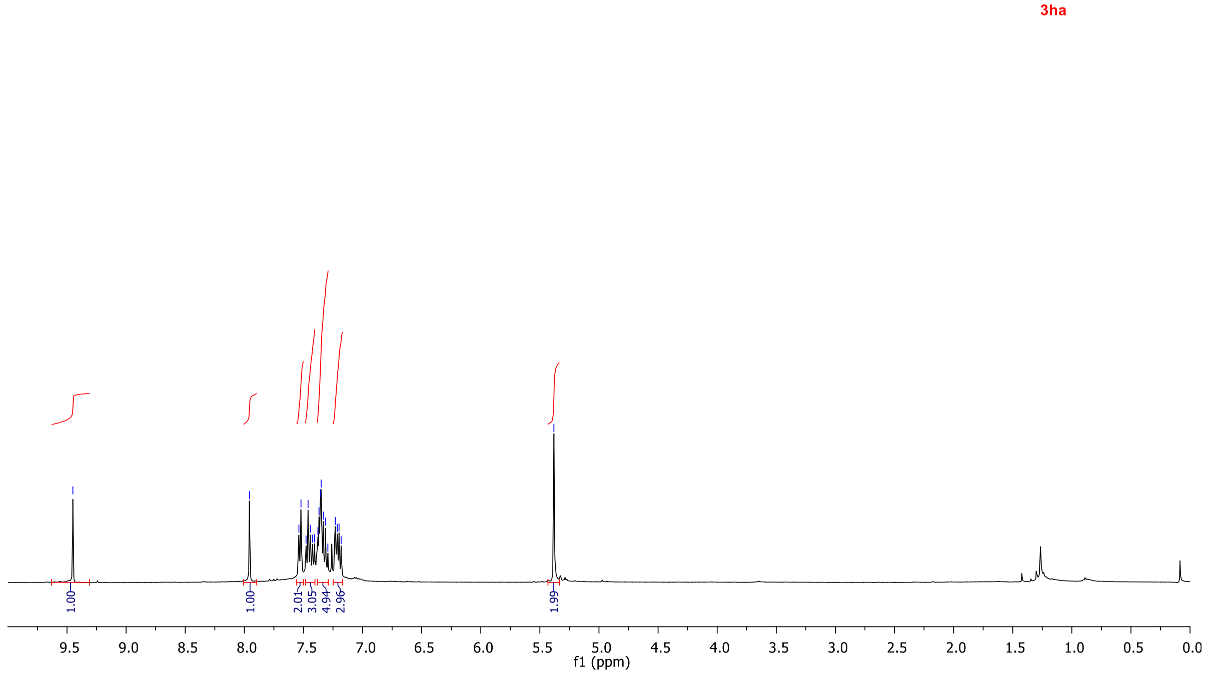

^{13}C{^{1}H} NMR spectrum of 3ha (100 MHz, $CDCl_3$)

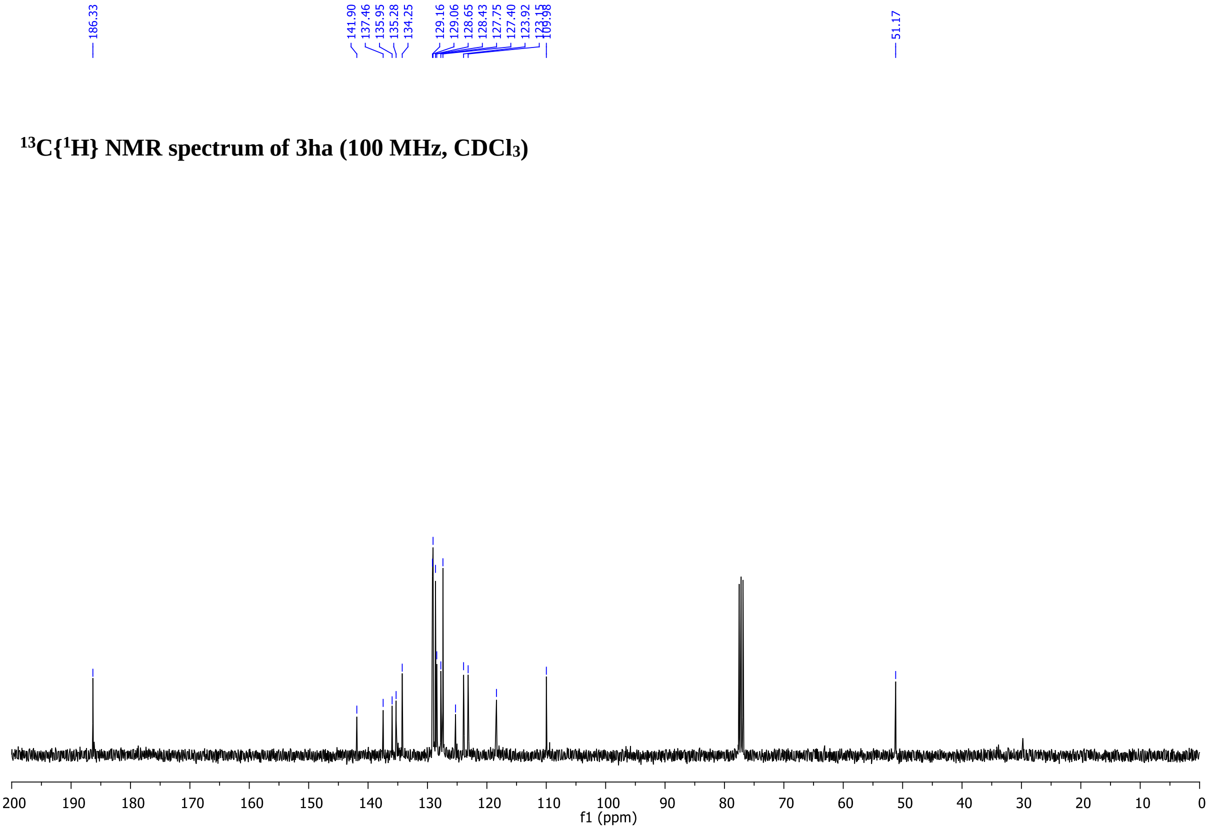

1-Methyl-4-phenyl-1*H*-indole-3-carbaldehyde (3ia)

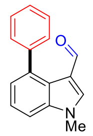

3ia

^{1}H NMR spectrum of 3ia (400 MHz, $CDCl_3$)

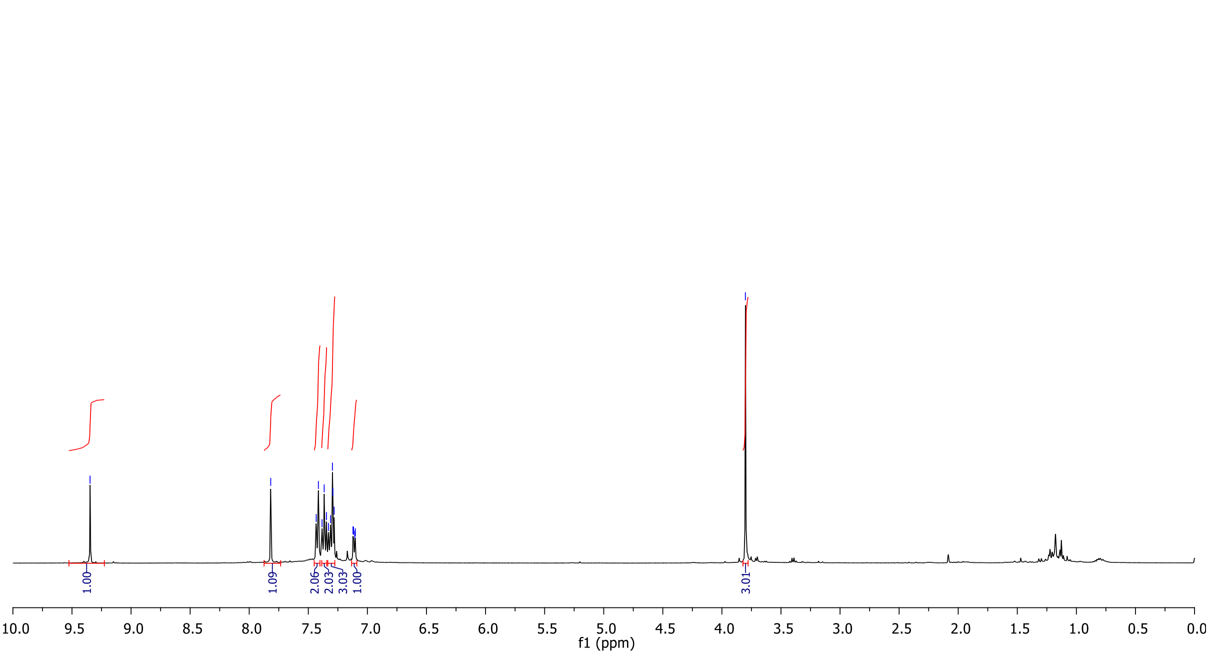

^{13}C{^{1}H} NMR spectrum of 3ia (100 MHz, $CDCl_3$)

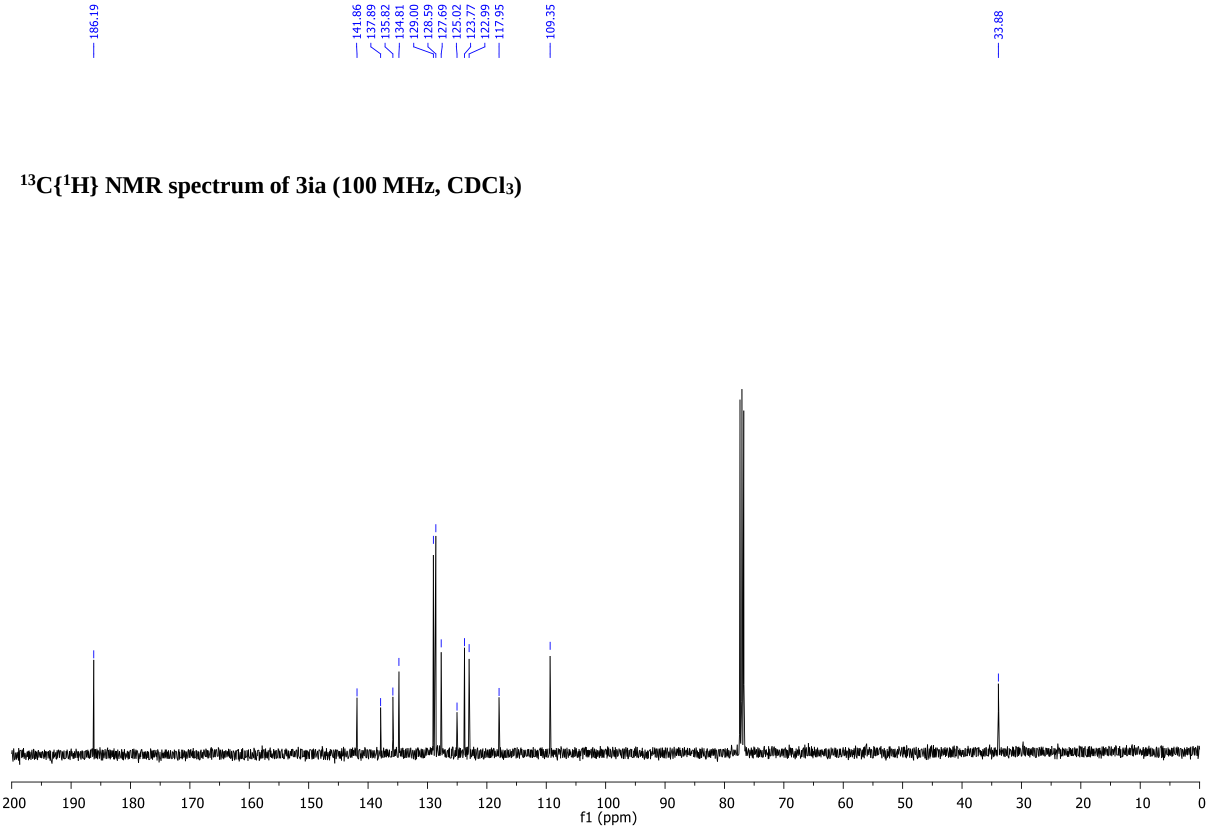

1-(1*H*-Indol-3-yl)ethan-1-one (4a)

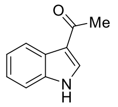

^{1}H NMR spectrum of 4a (400 MHz, $CDCl_3$)

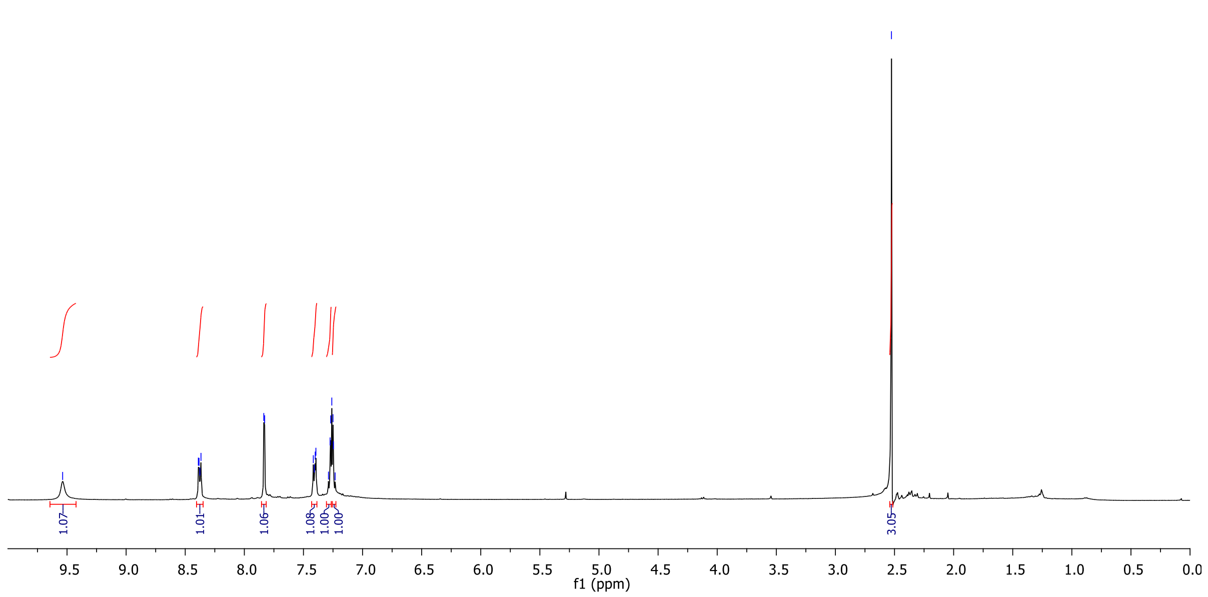

^{13}C{1H} NMR spectrum of 4a (100 MHz, $CDCl_3$)

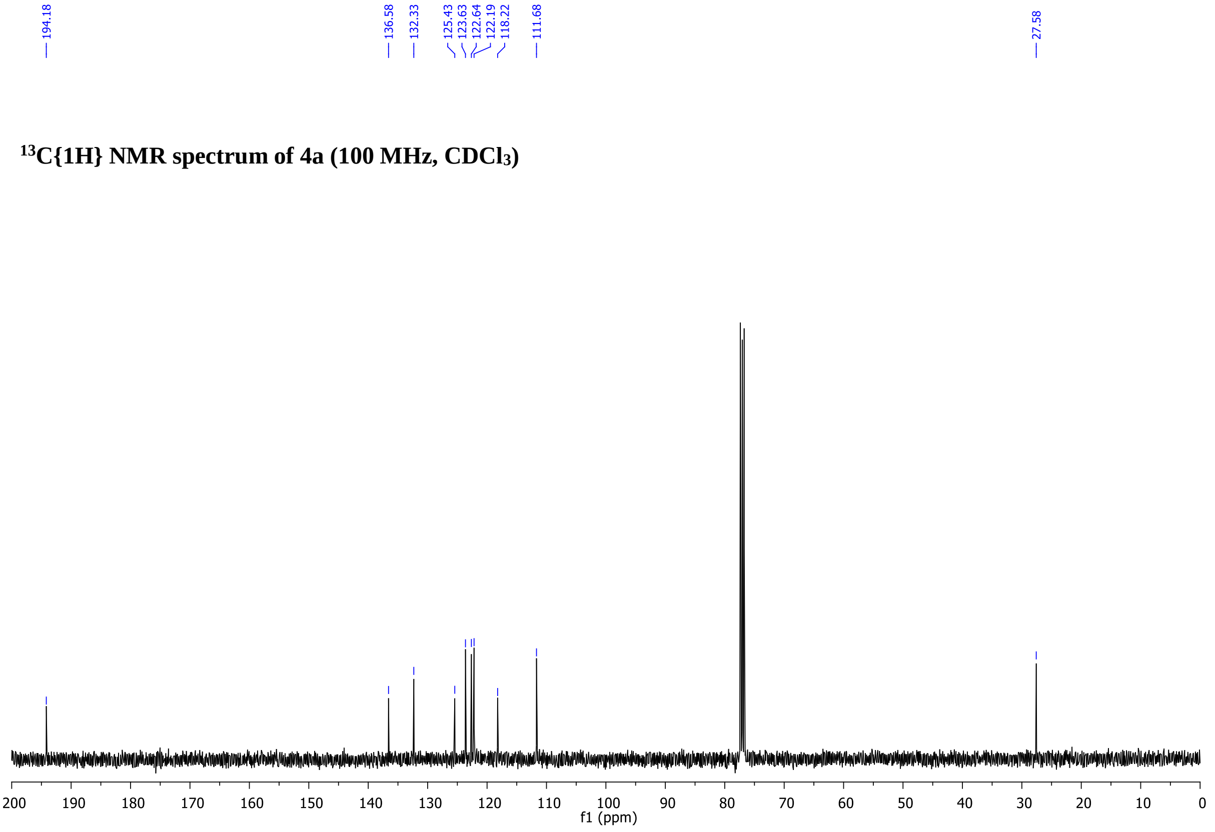

1-(7-Fluoro-1*H*-indol-3-yl)ethan-1-one (4b)

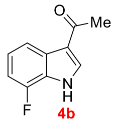

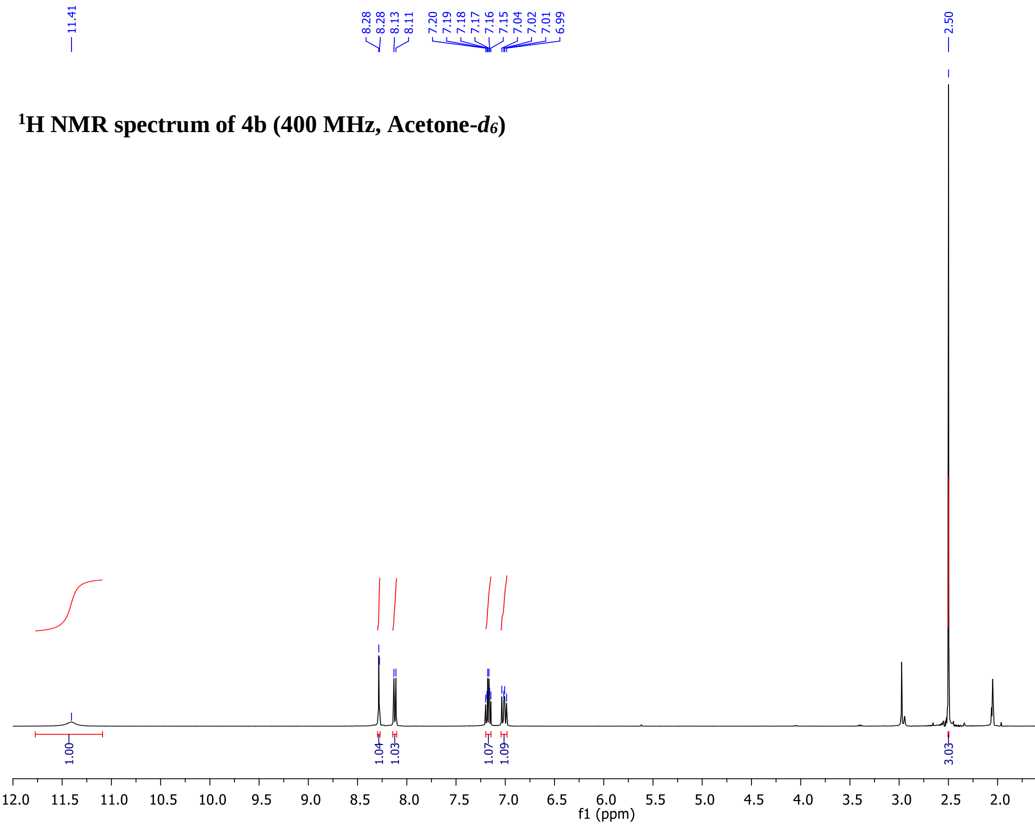

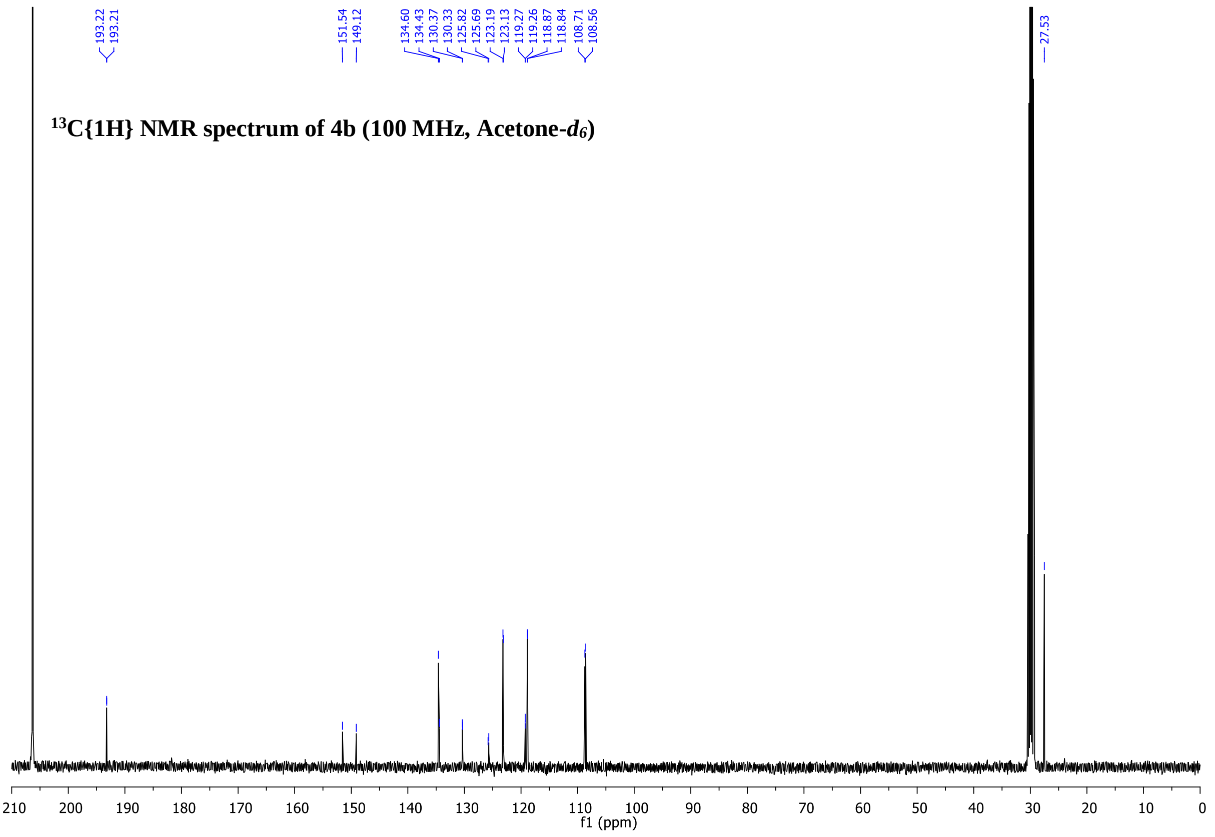

1-(7-Bromo-1*H*-indol-3-yl)ethan-1-one (4c)

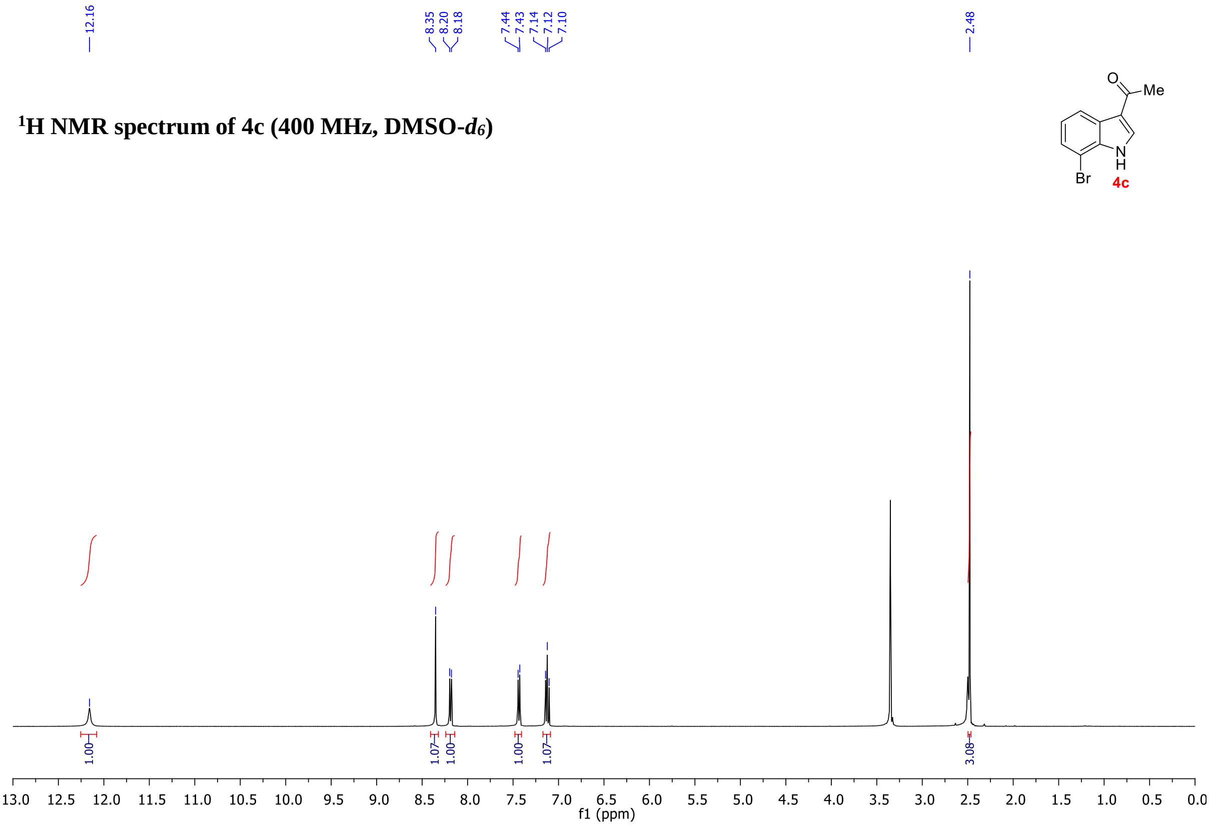

^{1}H NMR spectrum of 4c (400 MHz, DMSO-d_6)

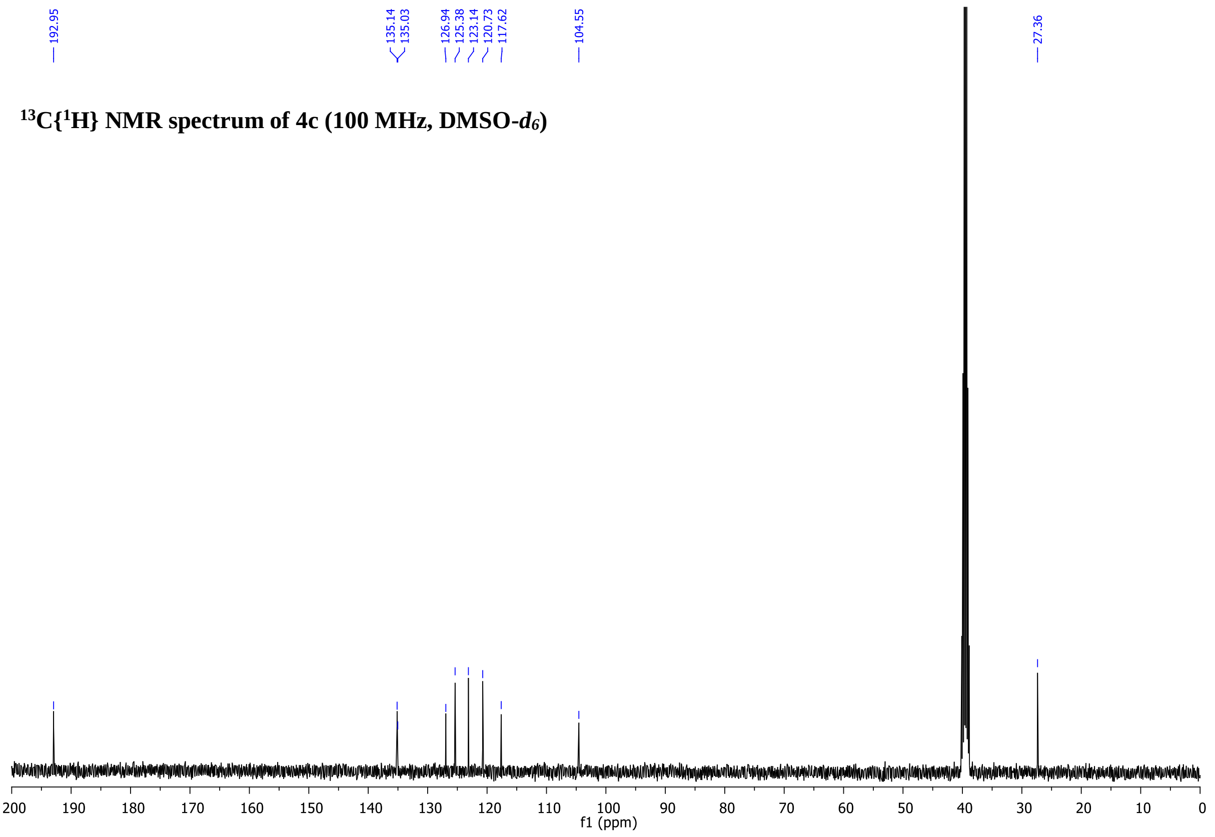

^{13}C{^{1}H} NMR spectrum of 4c (100 MHz, DMSO-d_6)

1-(2-Methyl-1*H*-indol-3-yl)ethan-1-one (4d)

^{1}H NMR spectrum of 4d (400 MHz, $CDCl_3$)

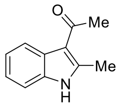

4d

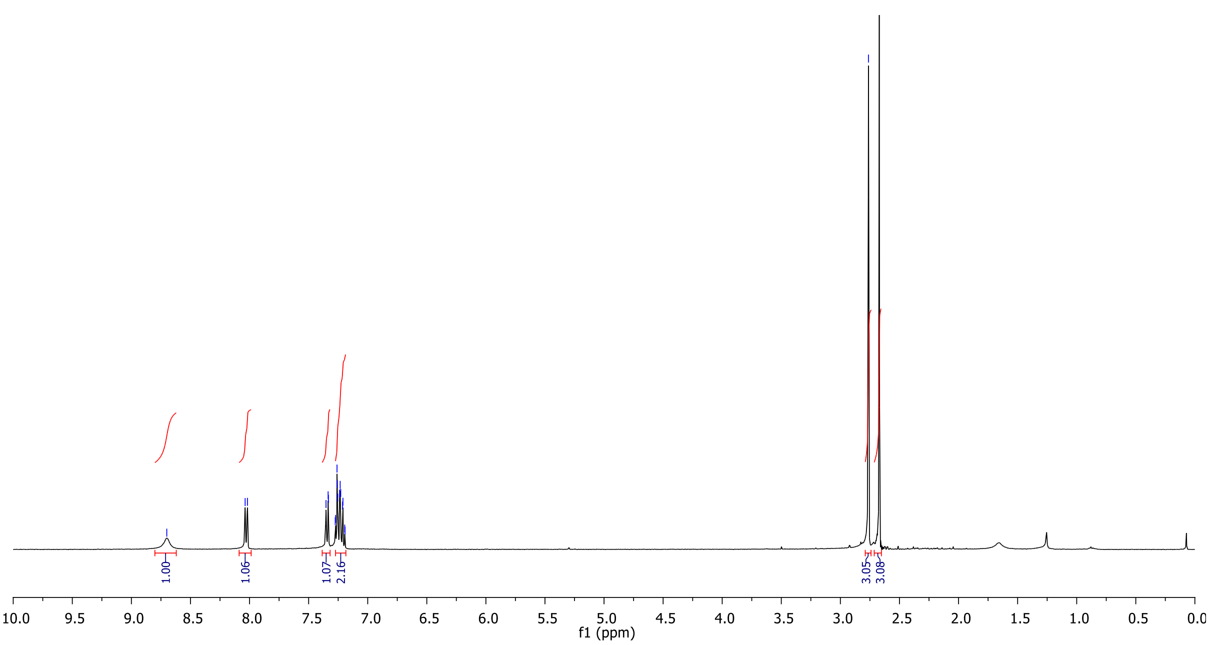

^{13}C{^{1}H} NMR spectrum of 4d (100 MHz, $CDCl_3$)

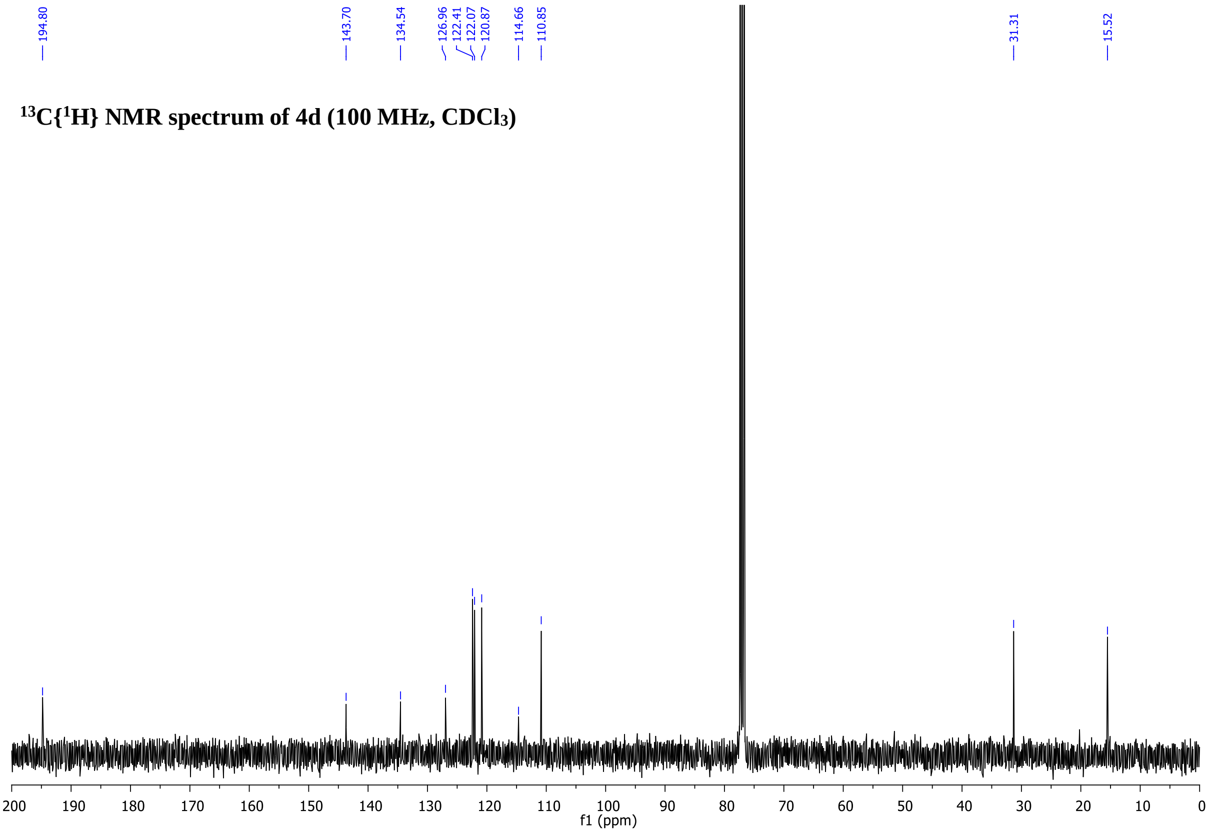

1-(4-Phenyl-1*H*-indol-2-yl)ethan-1-one (5aa)

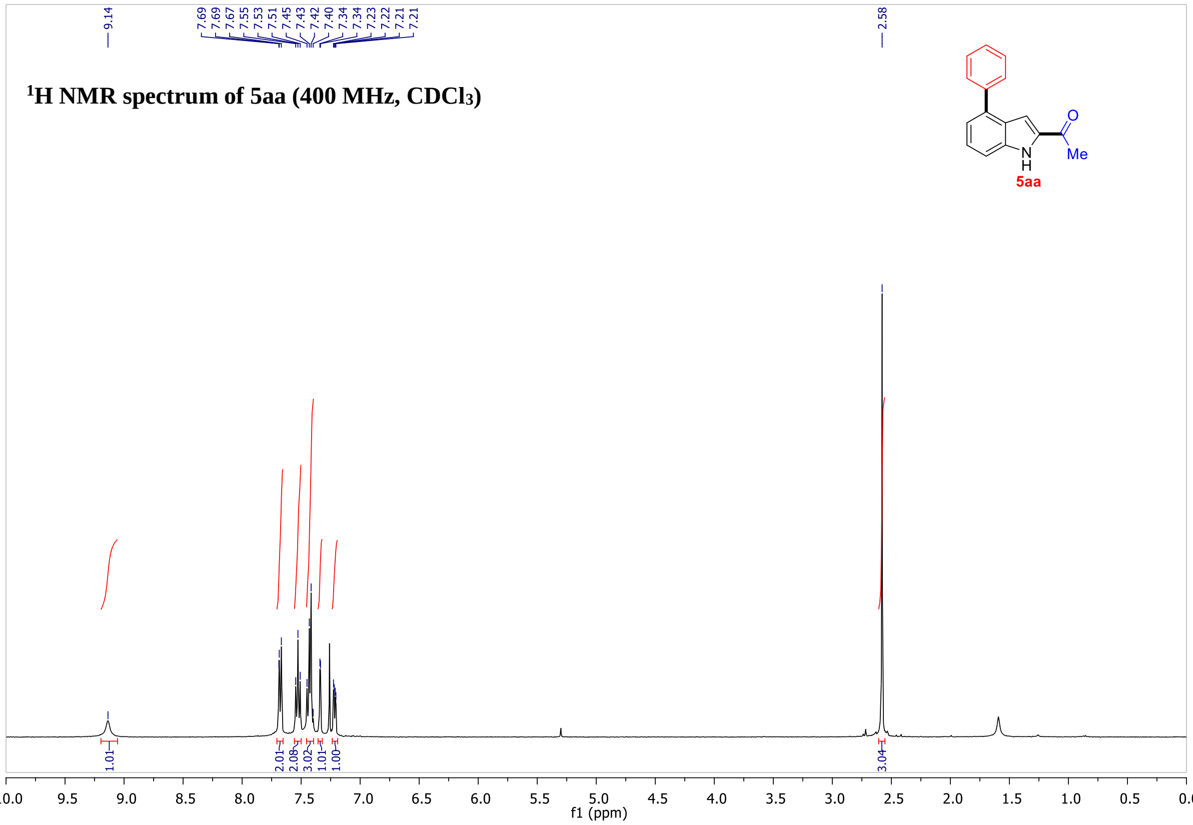

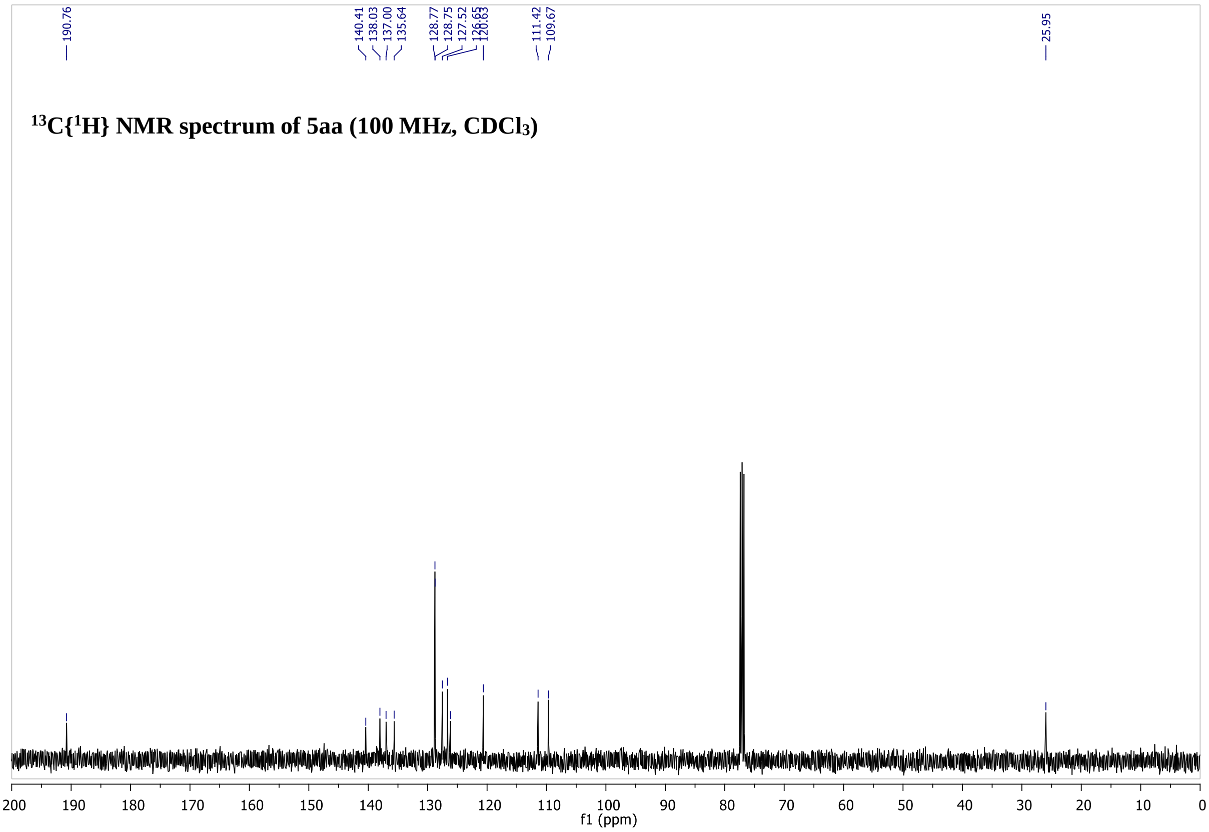

1-(4-(*p*-Tolyl)-1*H*-indol-2-yl)ethan-1-one (5ab)

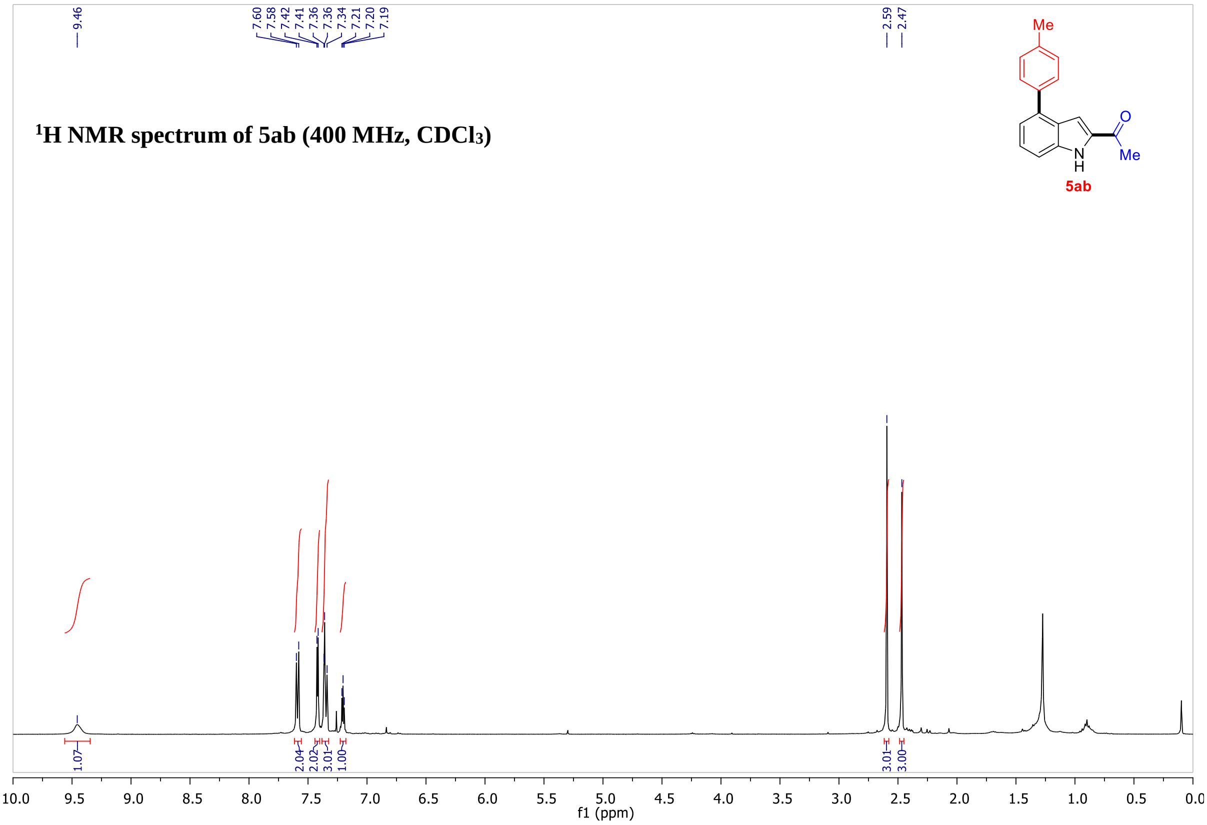

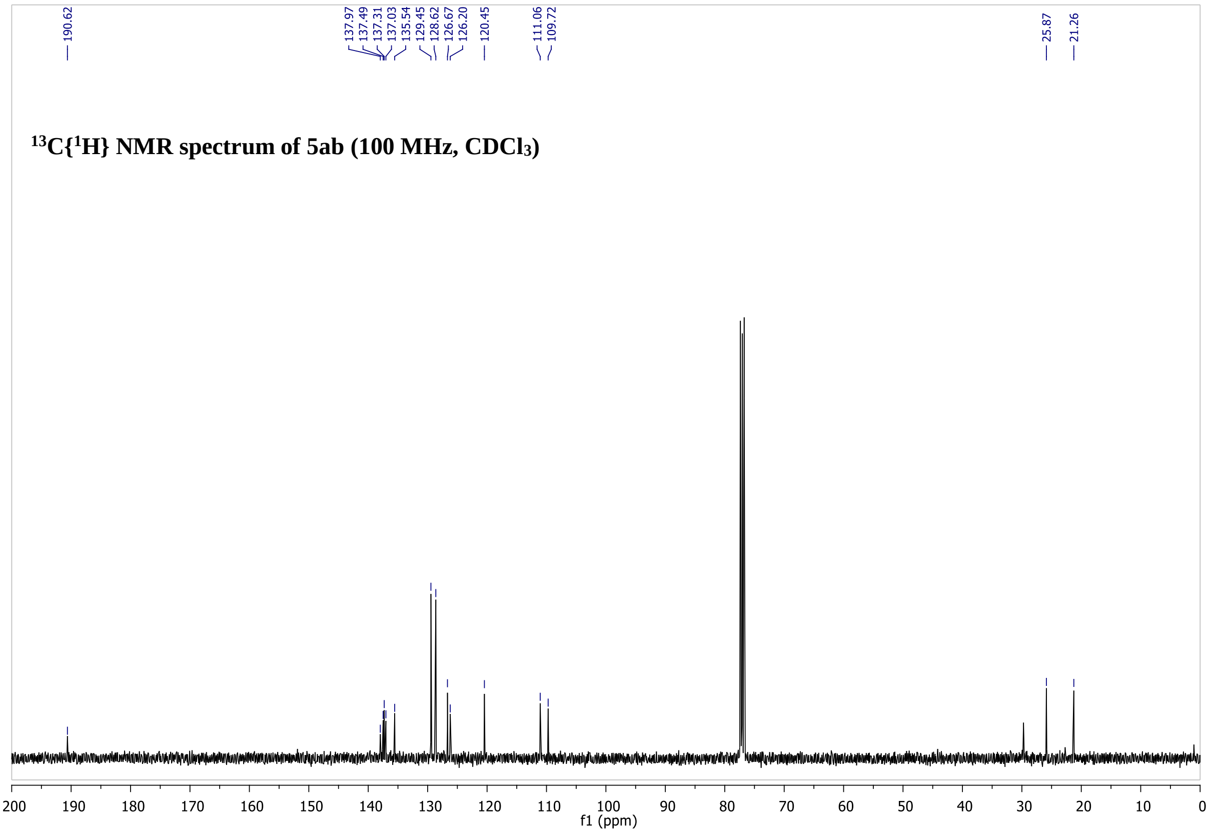

1-(4-(*m*-Tolyl)-1*H*-indol-2-yl)ethan-1-one (5ac)

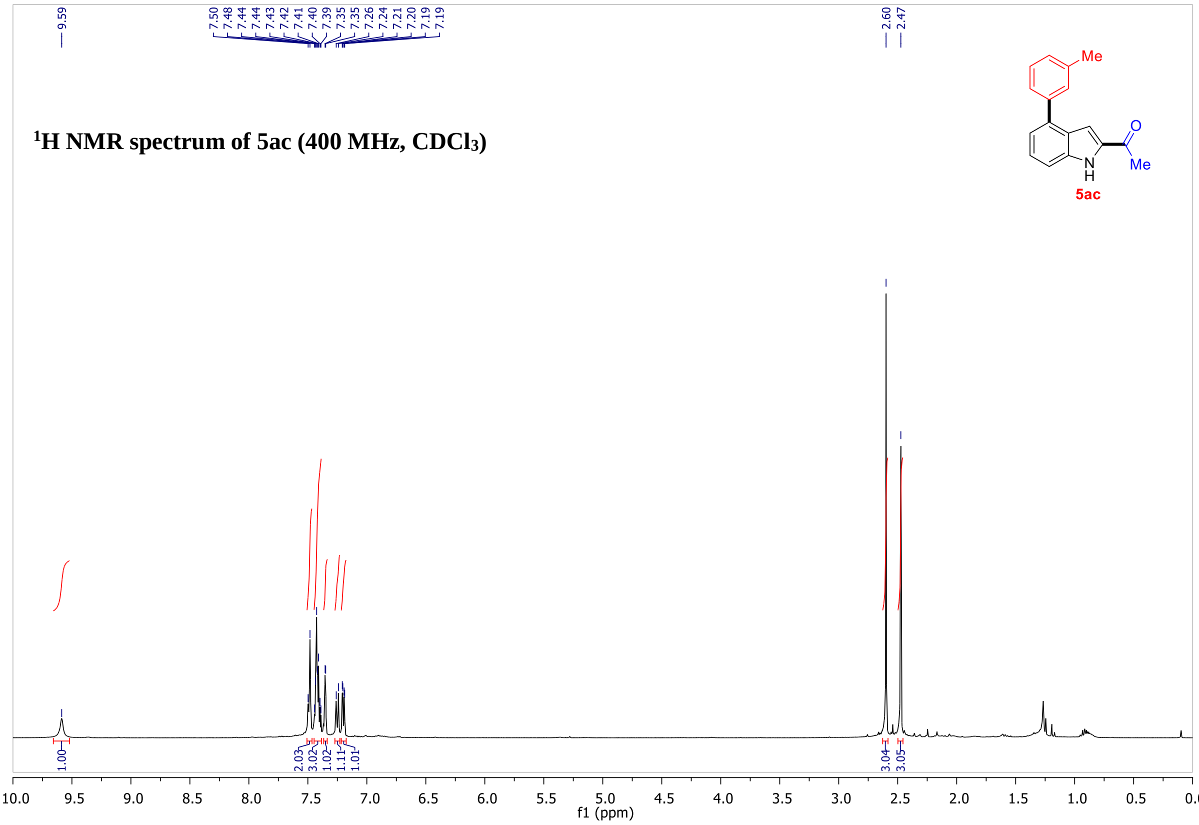

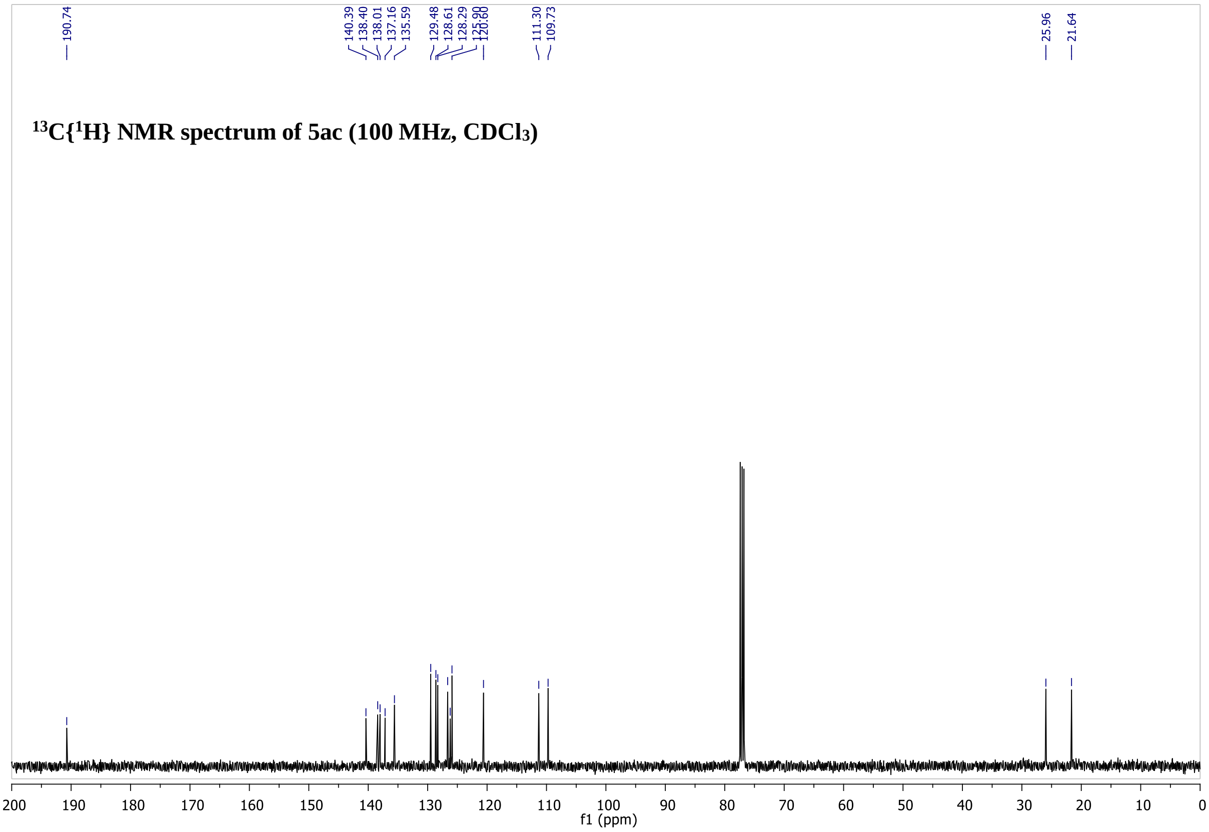

1-(4-(4-(*tert*-Butyl)phenyl)-1*H*-indol-2-yl)ethan-1-one (5ad)

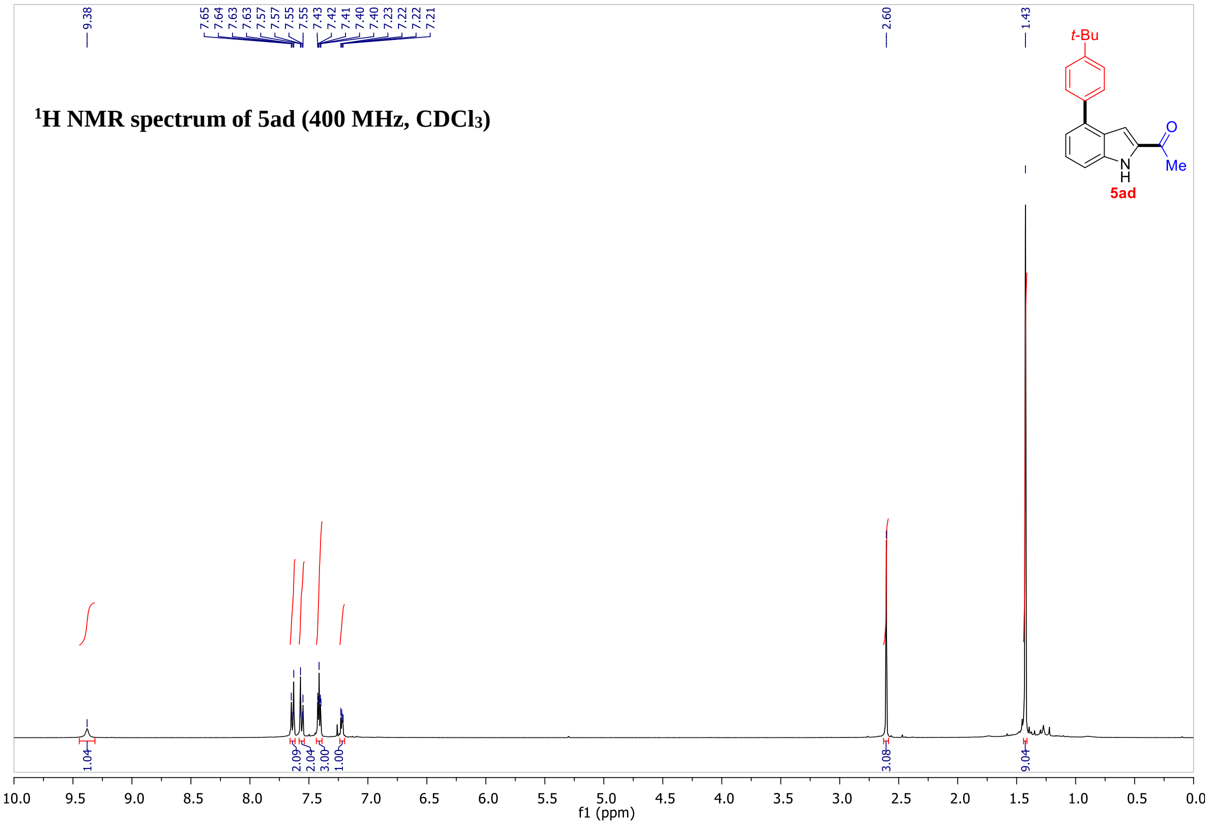

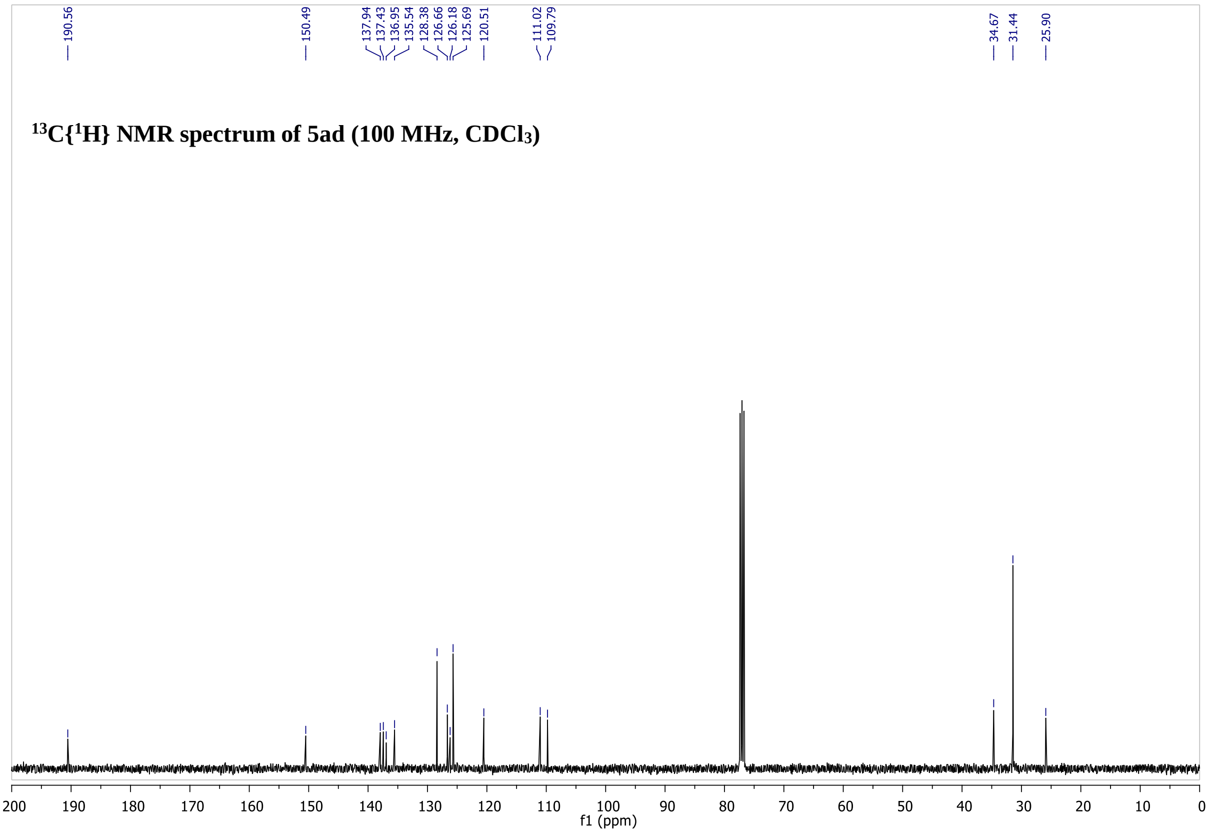

1-(4-(4-Bromophenyl)-1*H*-indol-2-yl)ethan-1-one (5ae)

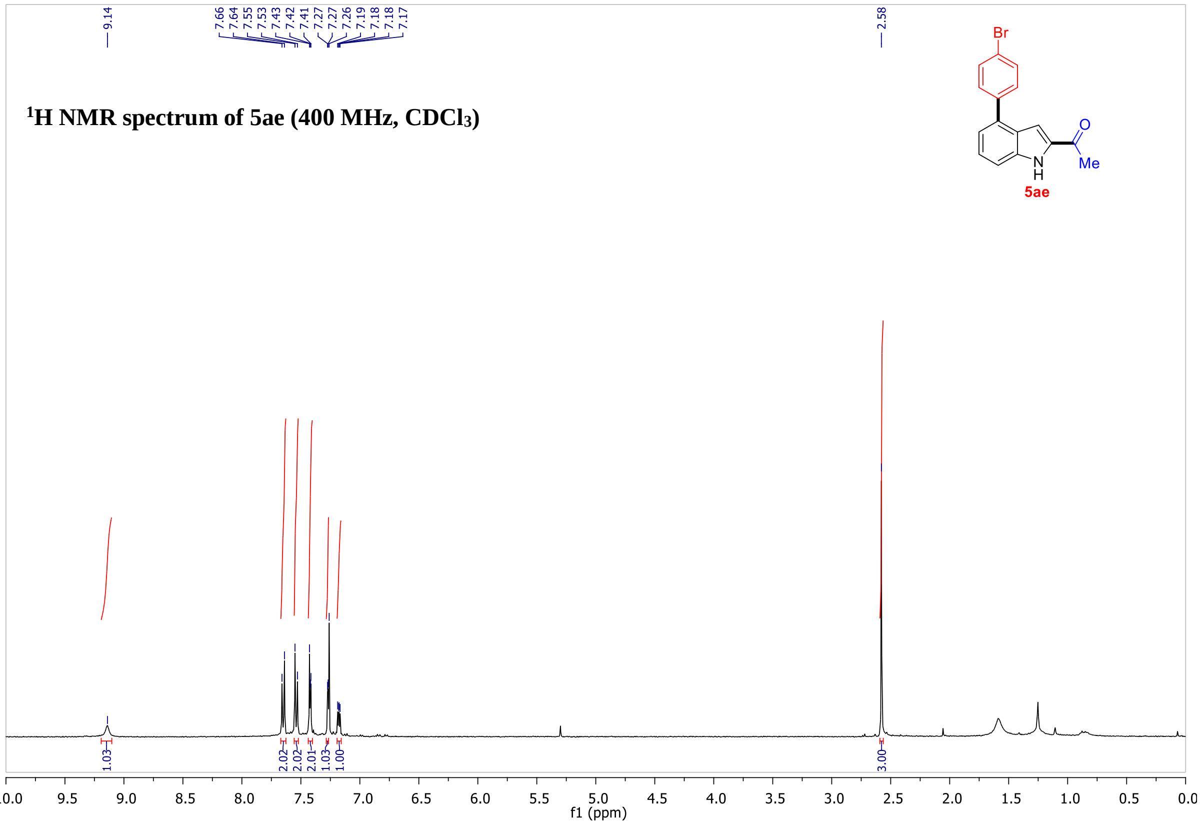

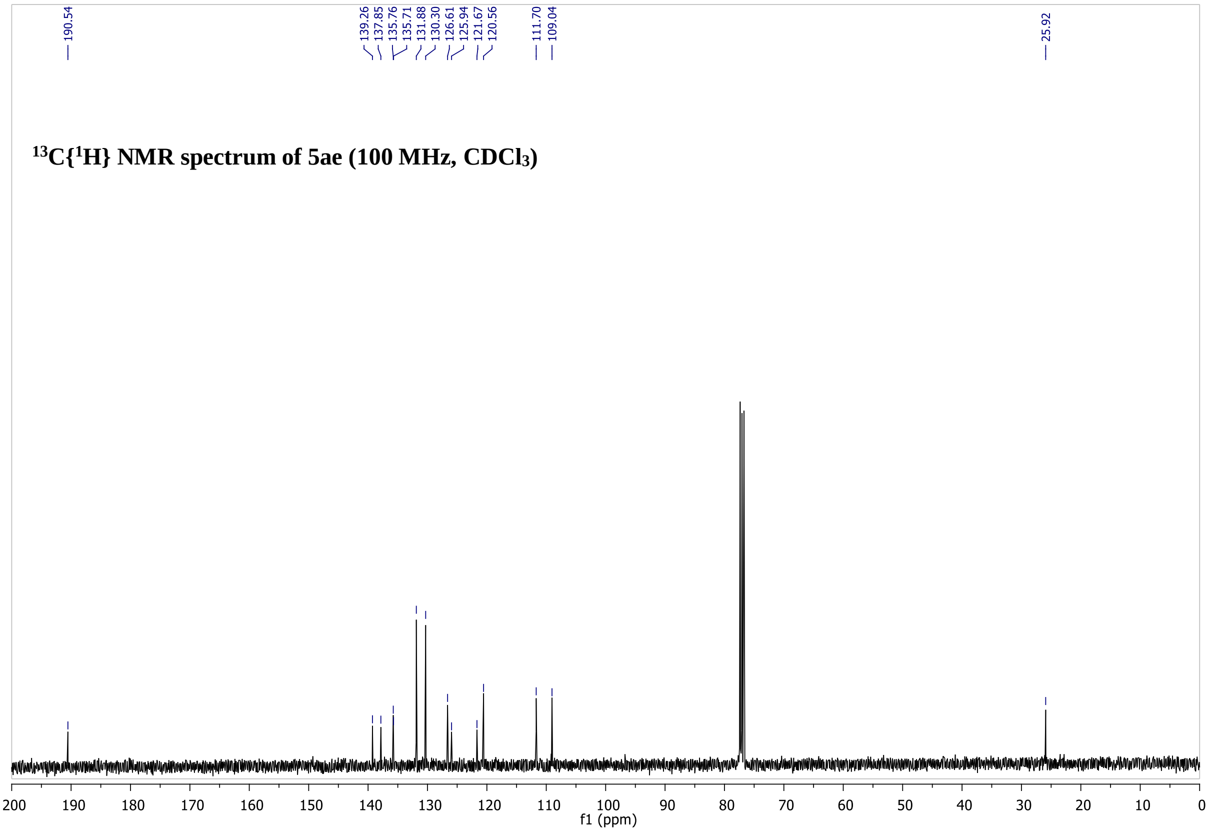

1-(4-(4-Methoxyphenyl)-1*H*-indol-2-yl)ethan-1-one (5af)

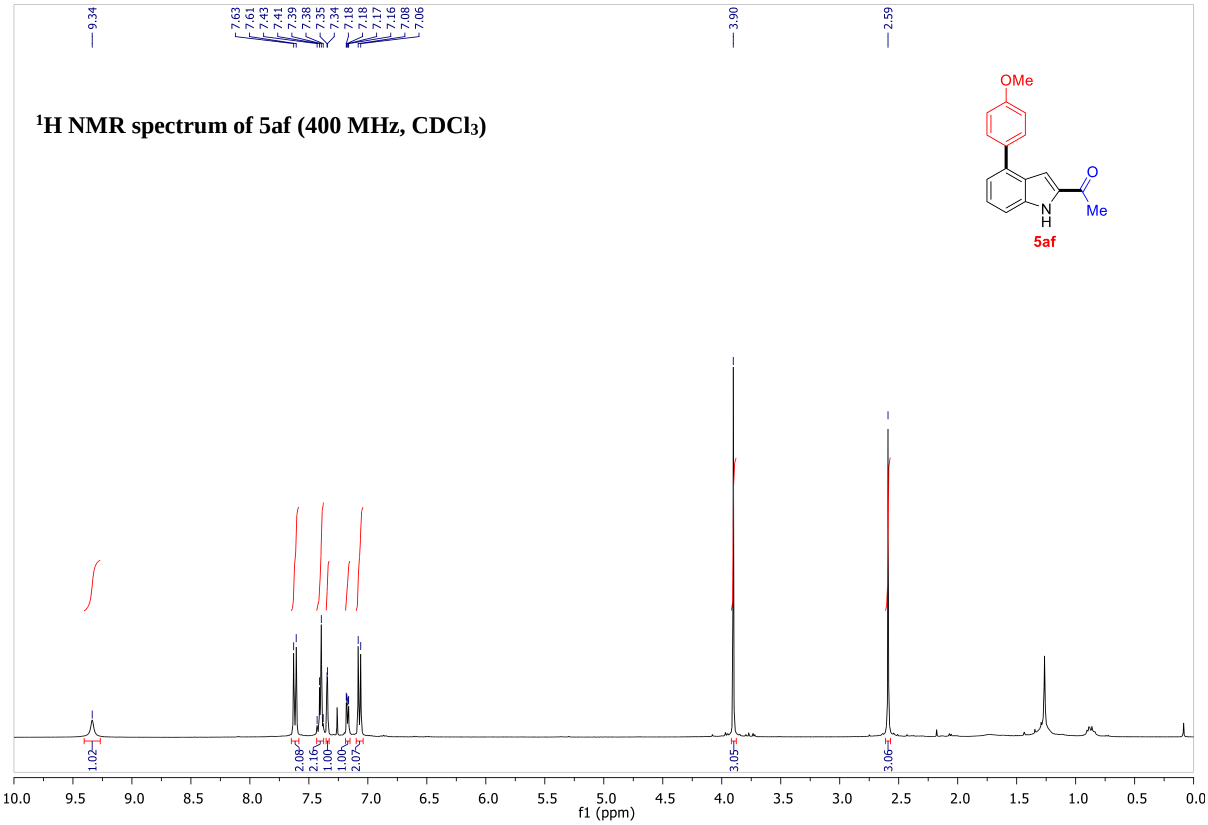

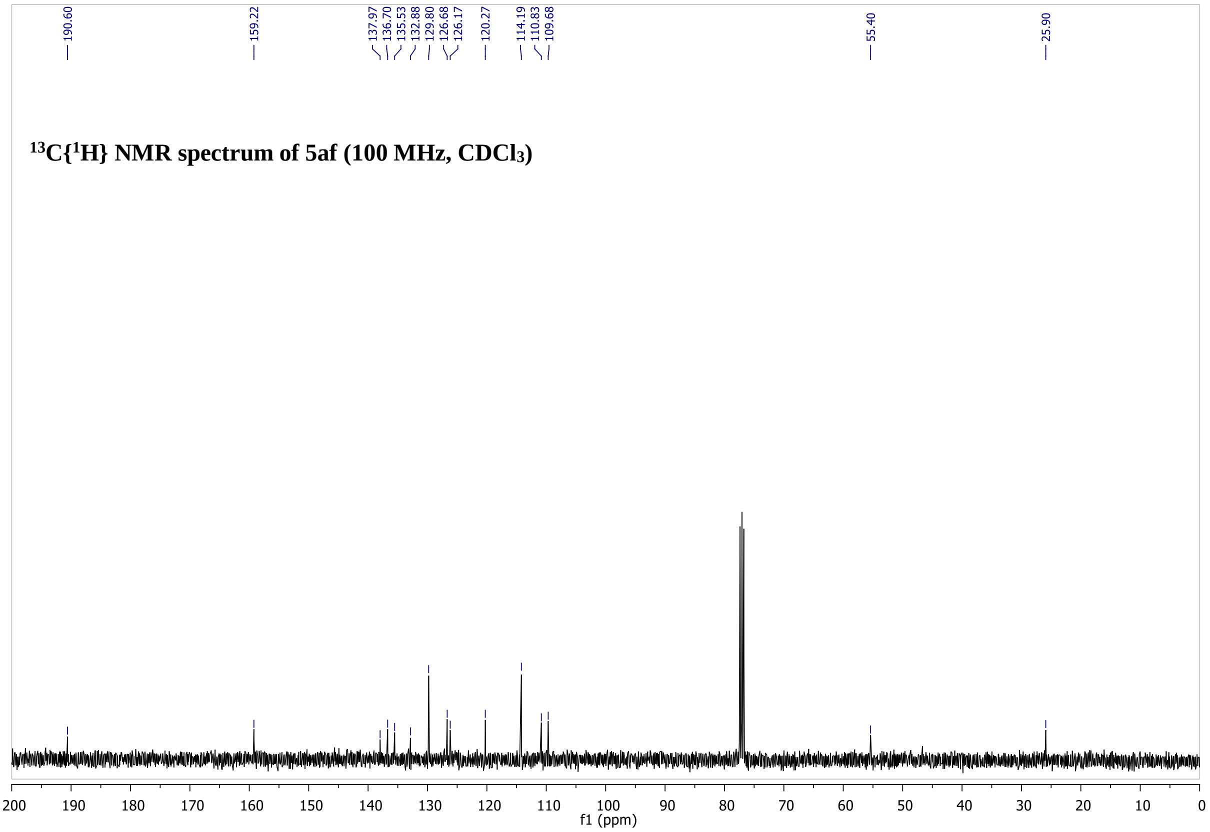

Methyl 4-(2-acetyl-1*H*-indol-4-yl)benzoate (5ag)

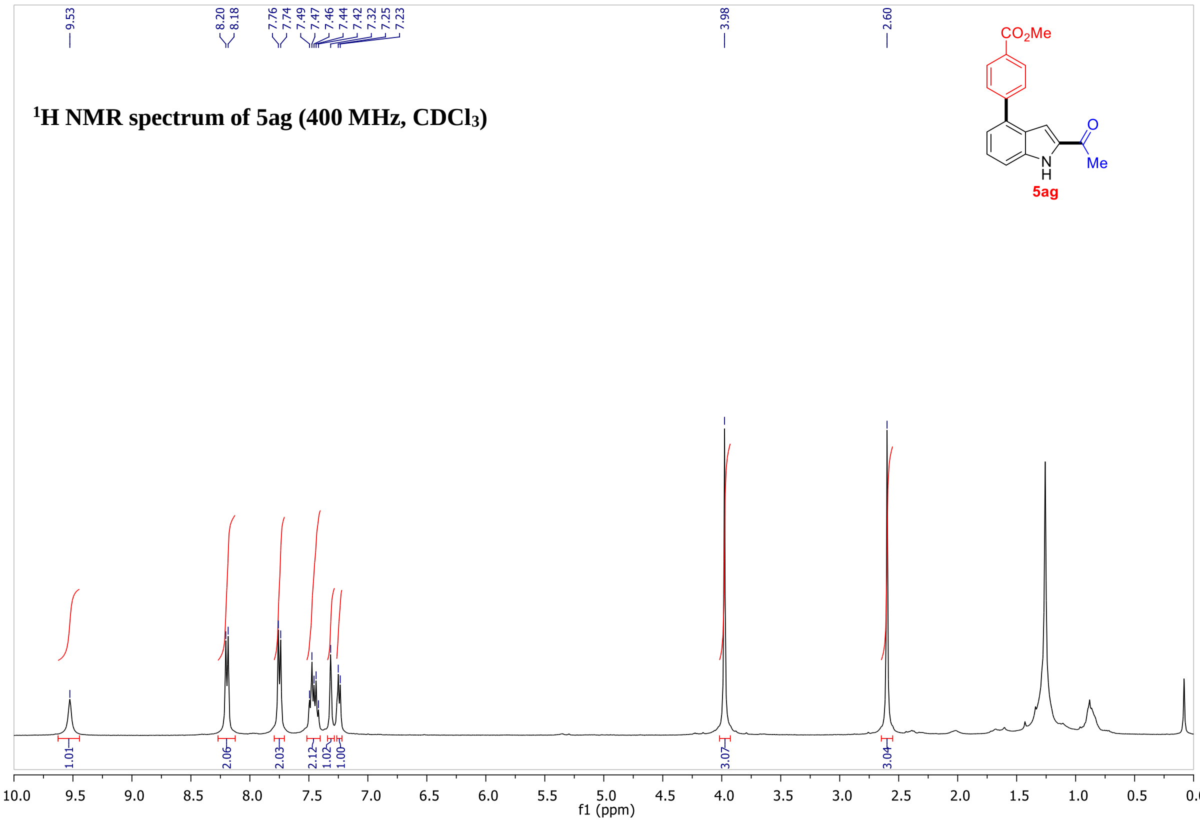

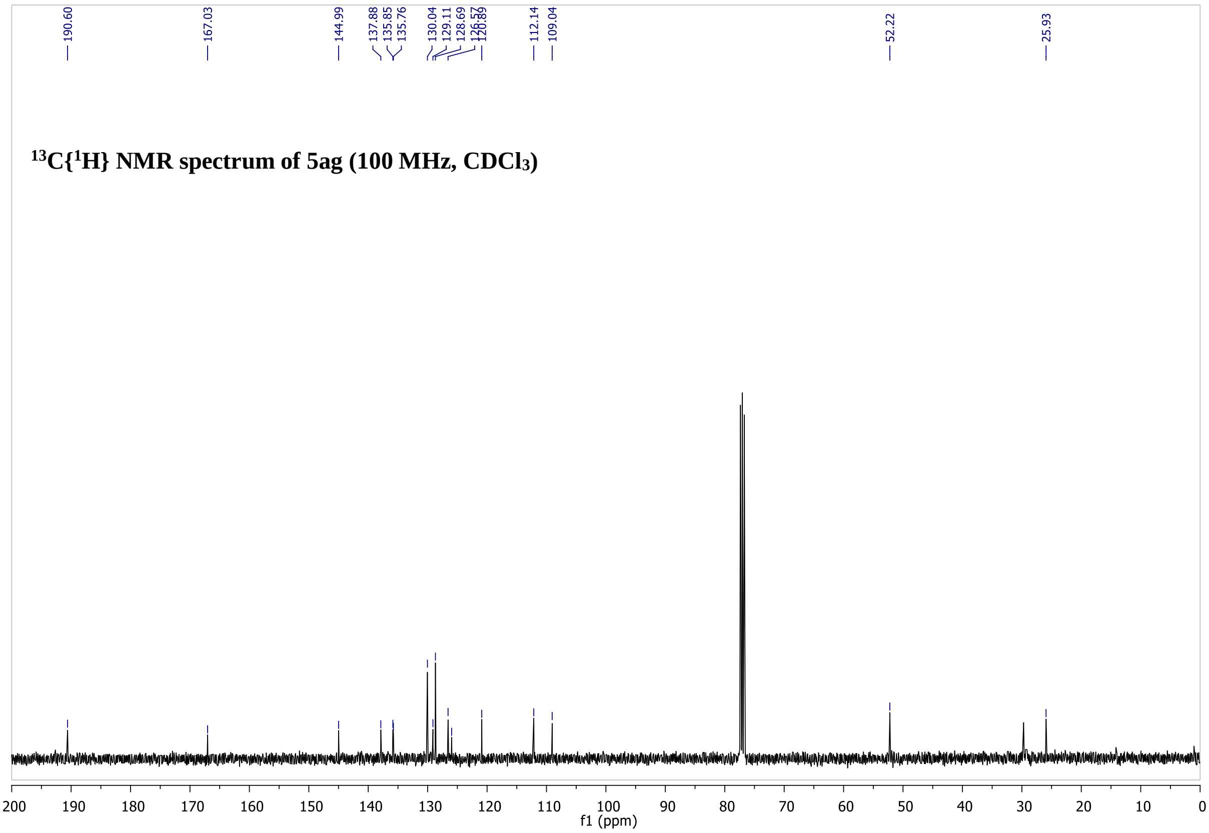

1-(4-(2-Acetyl-1*H*-indol-4-yl)phenyl)ethan-1-one (5ah)

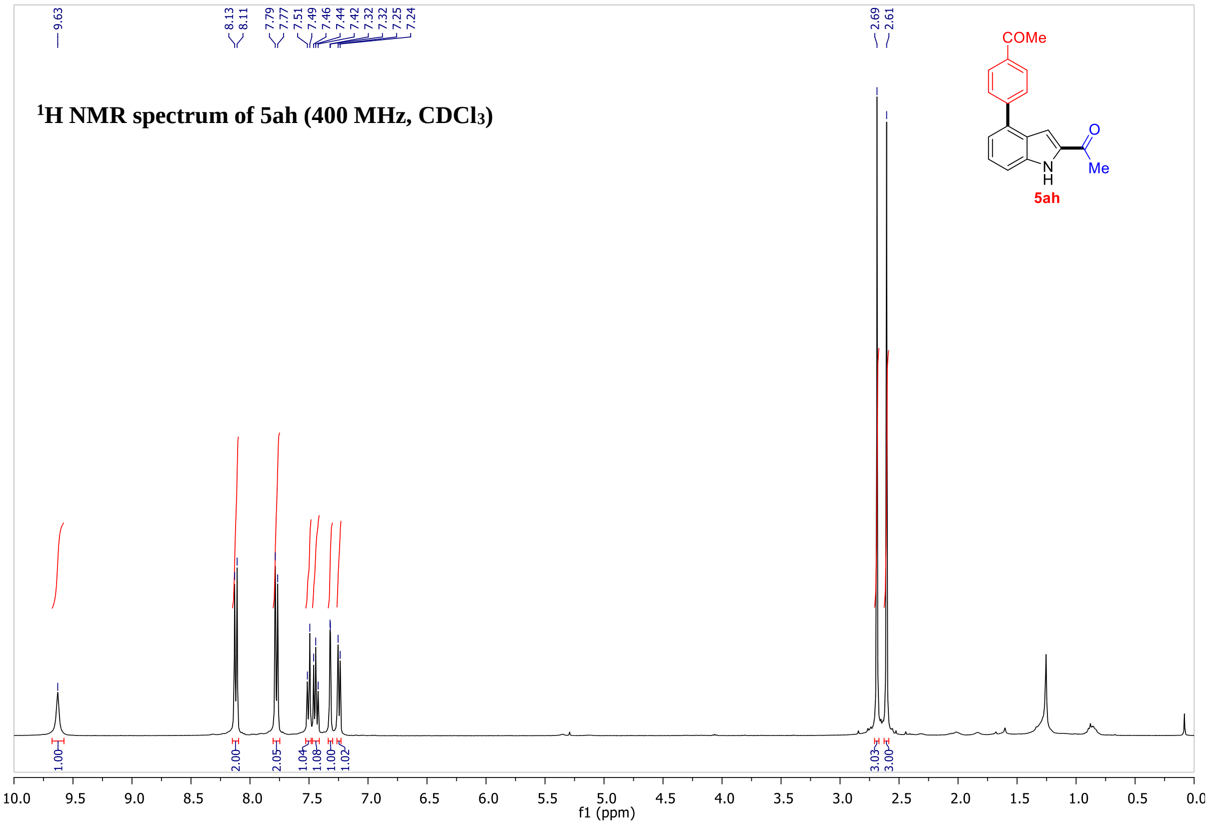

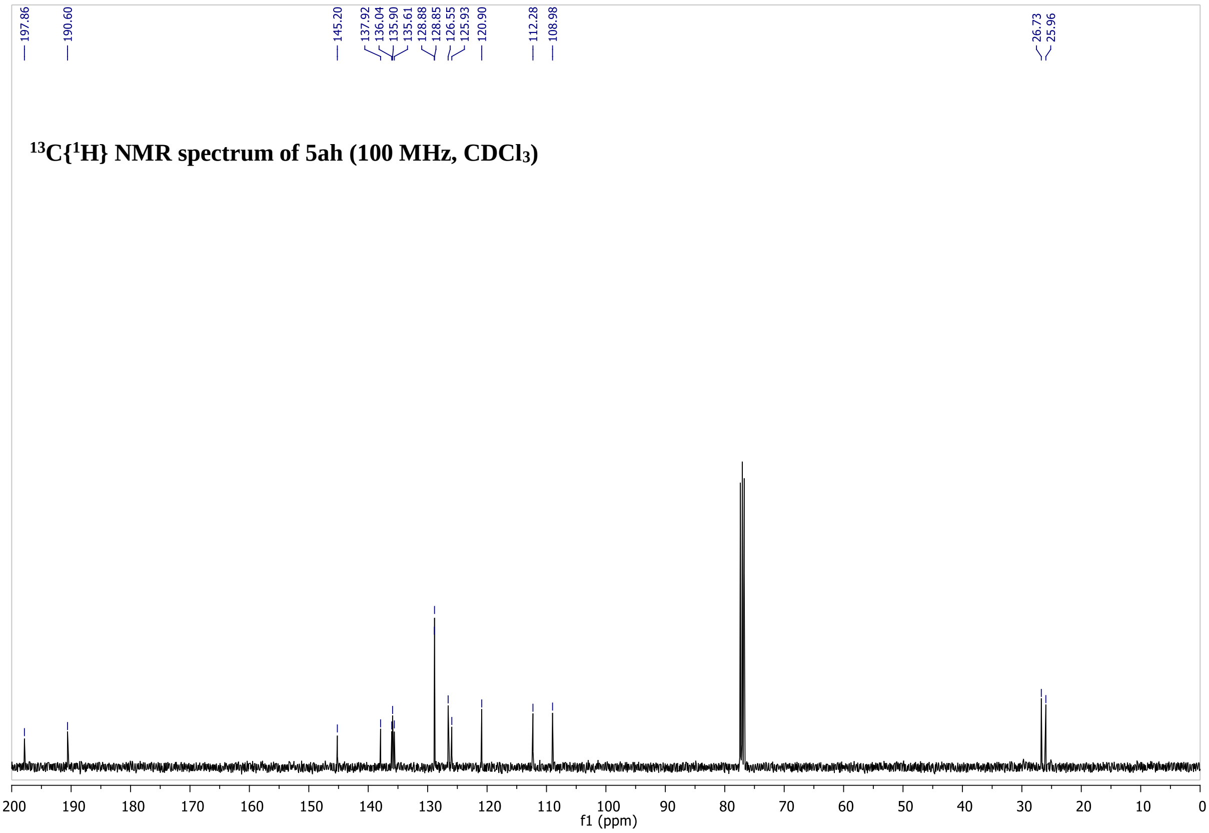

1-(4-(4-(Trifluoromethyl)phenyl)-1*H*-indol-2-yl)ethan-1-one (5ai)

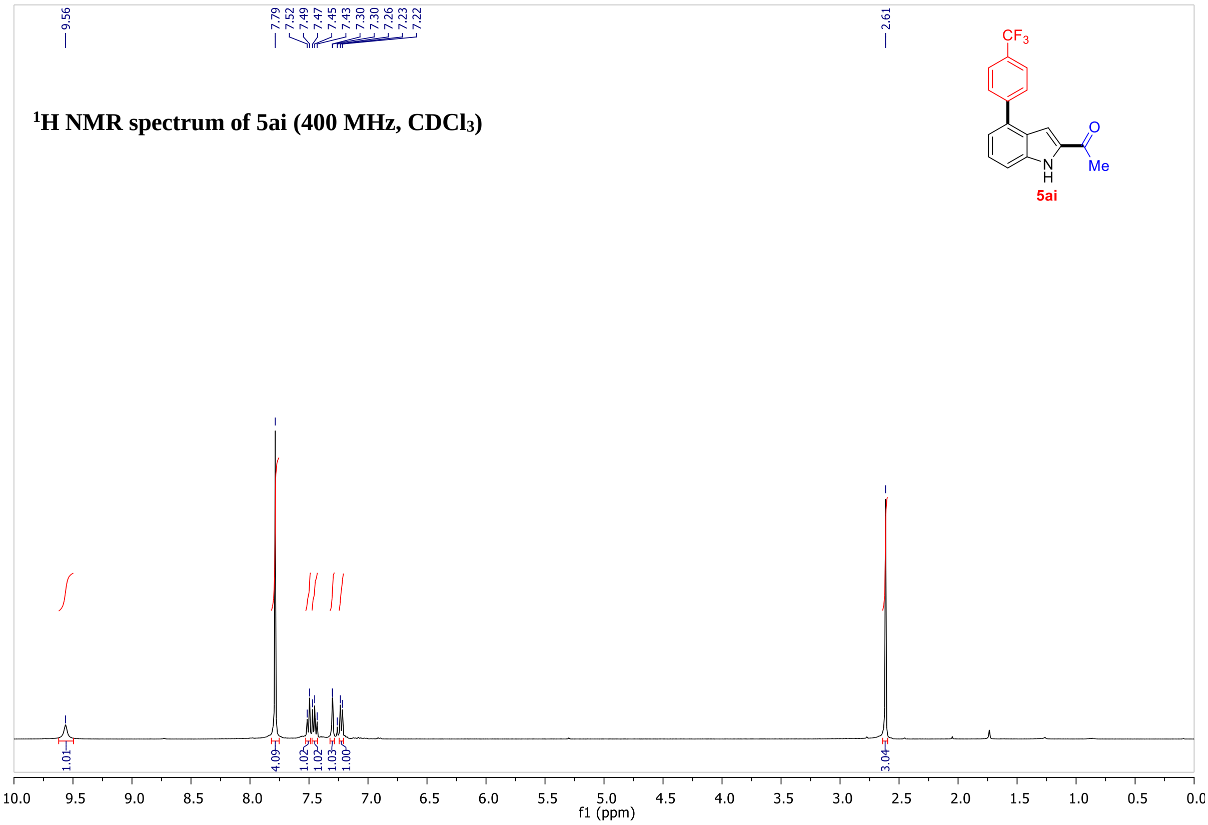

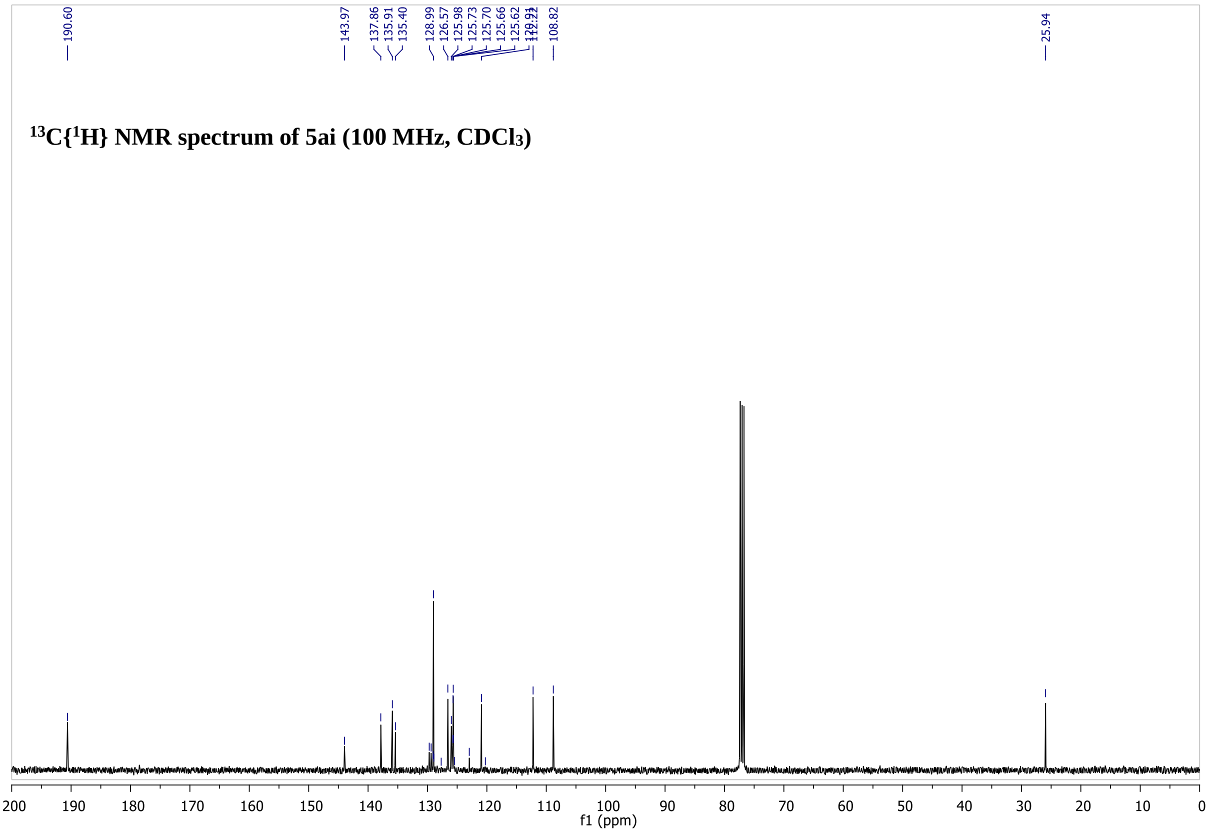

1-(4-(3,4-Dimethylphenyl)-1*H*-indol-2-yl)ethan-1-one (5ak)

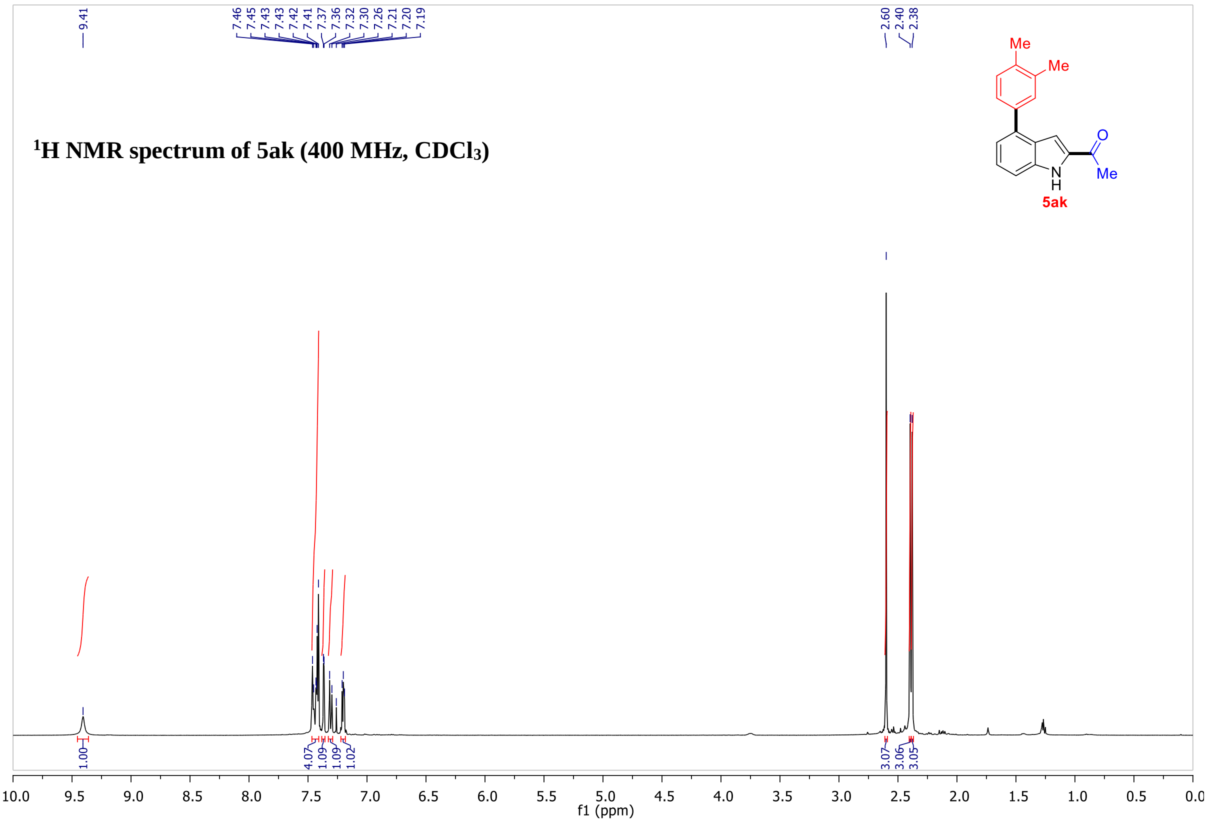

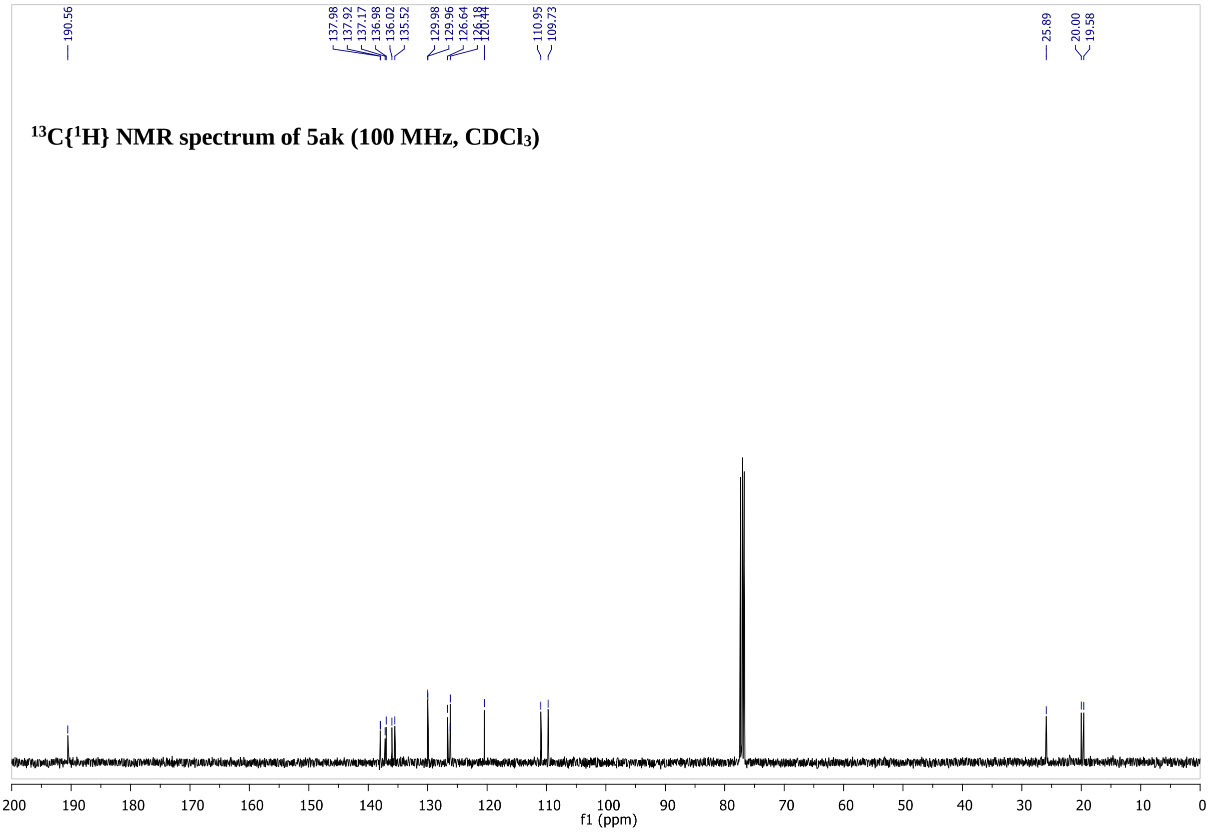

1-(4-(3-Nitrophenyl)-1*H*-indol-2-yl)ethan-1-one (5al)

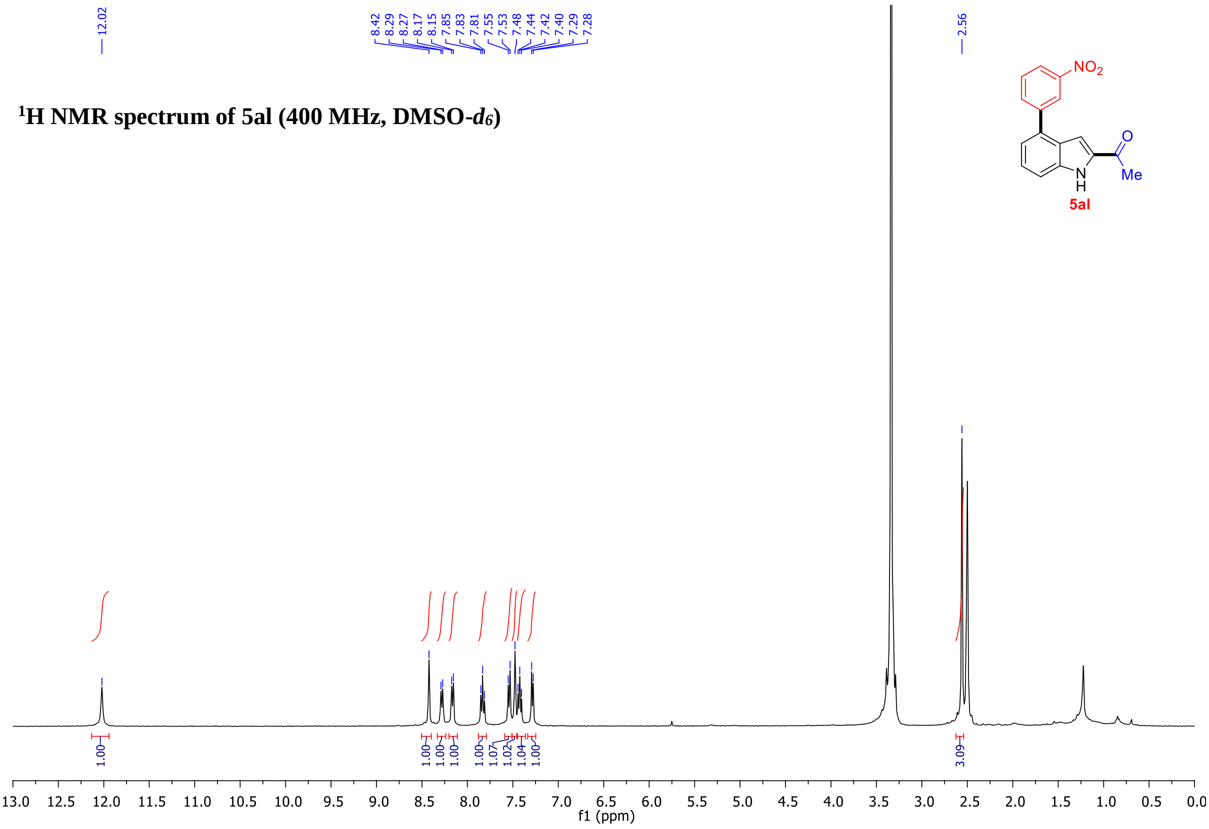

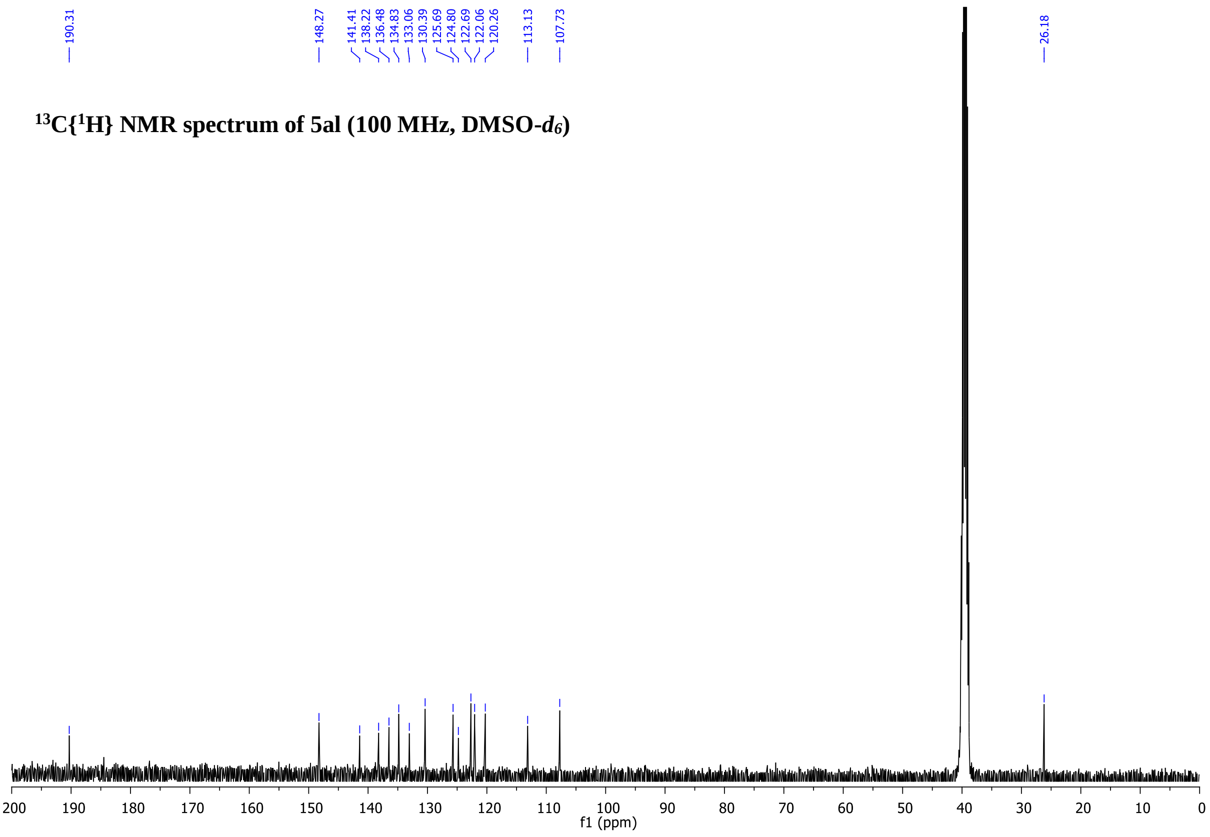

1-(7-Fluoro-4-phenyl-1*H*-indol-2-yl)ethan-1-one (5ba)

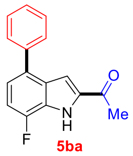

5ba

^{1}H NMR spectrum of 5ba (400 MHz, $CDCl_3$)

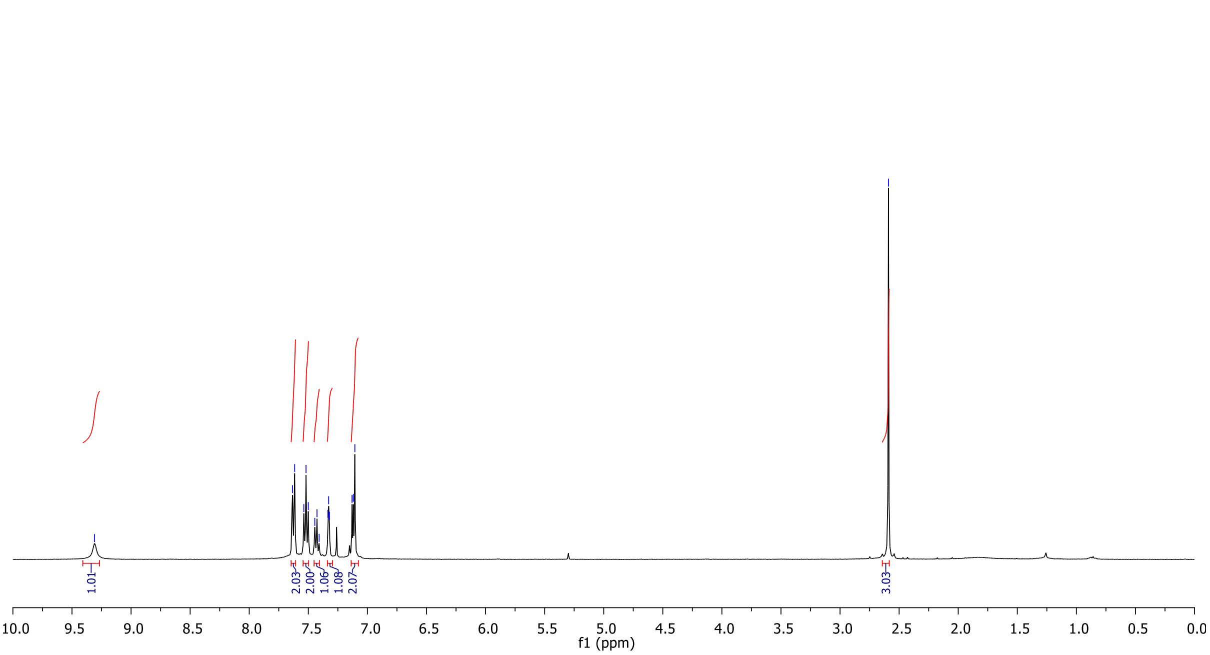

^{13}C{^{1}H} NMR spectrum of 5ba (100 MHz, $CDCl_3$)

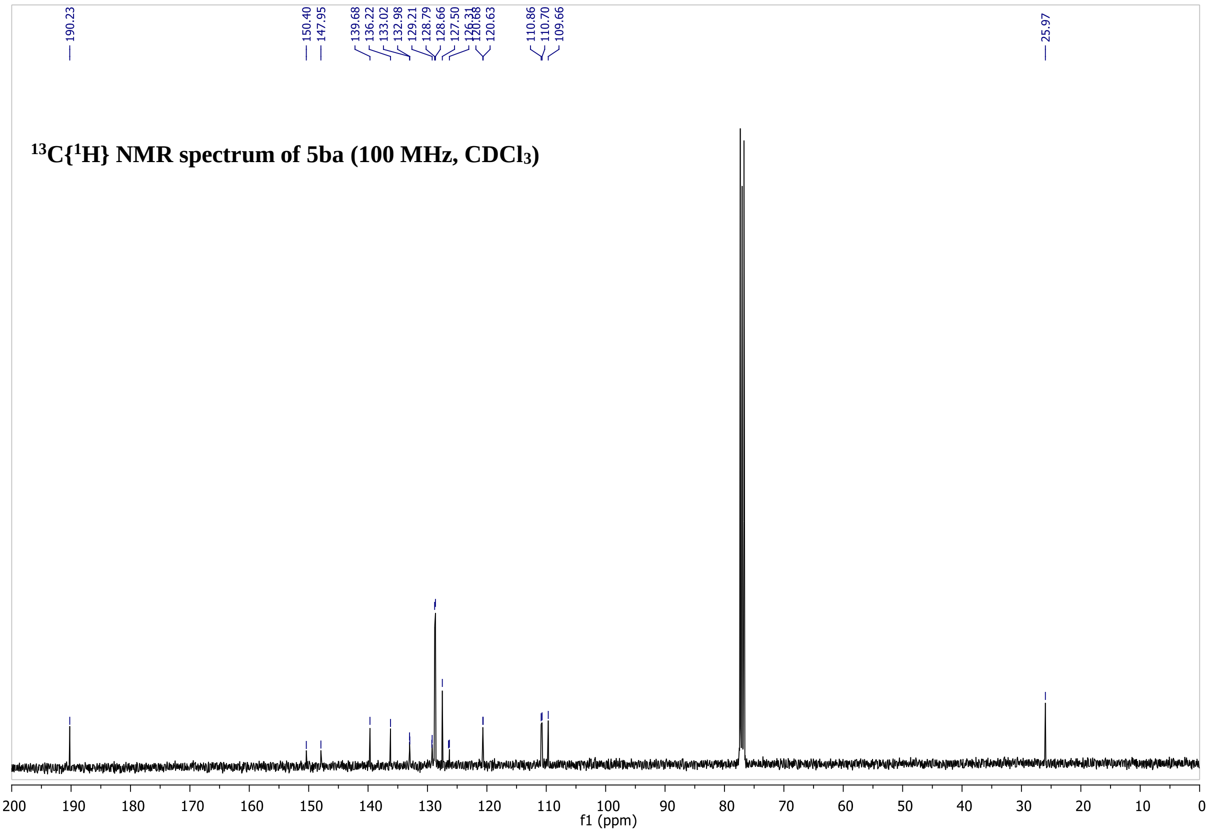

1-(7-Bromo-4-phenyl-1*H*-indol-2-yl)ethan-1-one (5ca)

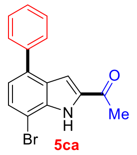

5ca

^{1}H NMR spectrum of 5ca (400 MHz, $CDCl_3$)

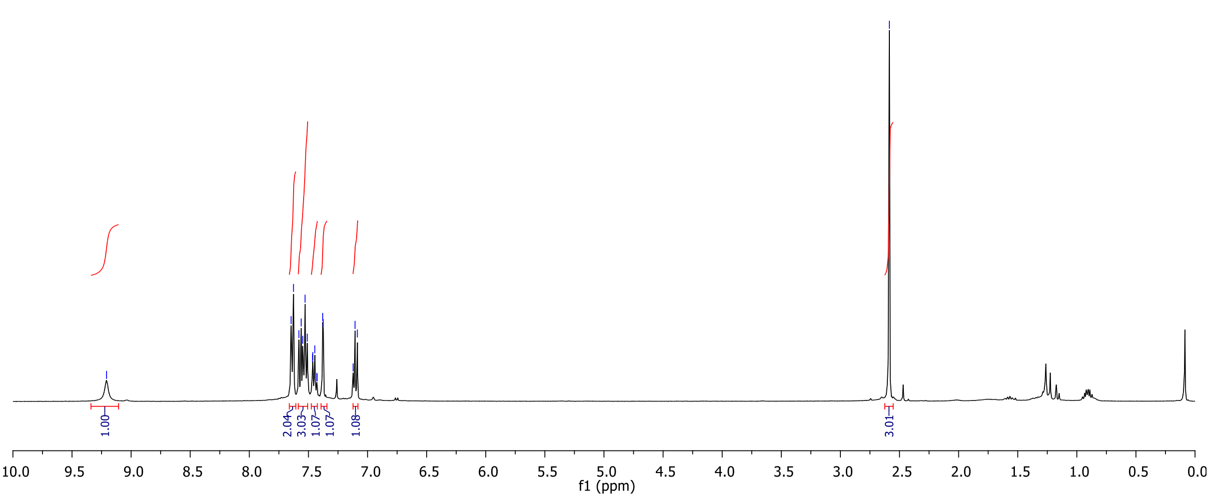

^{13}C{^{1}H} NMR spectrum of 5ca (100 MHz, $CDCl_3$)

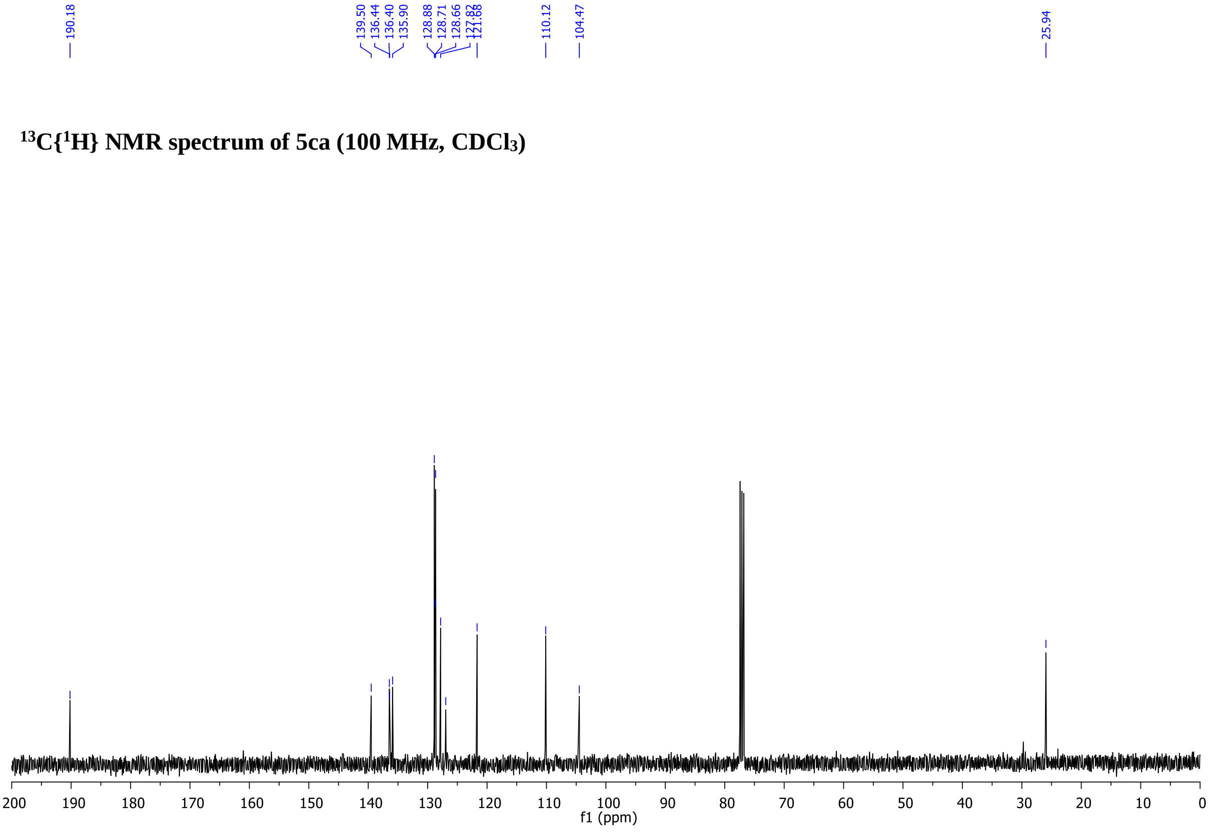

1-(7-Bromo-4-phenyl-1*H*-indol-3-yl)ethan-1-one (6ca)

^{1}H NMR spectrum of 6ca (400 MHz, $CDCl_3$)

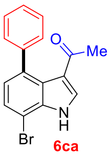

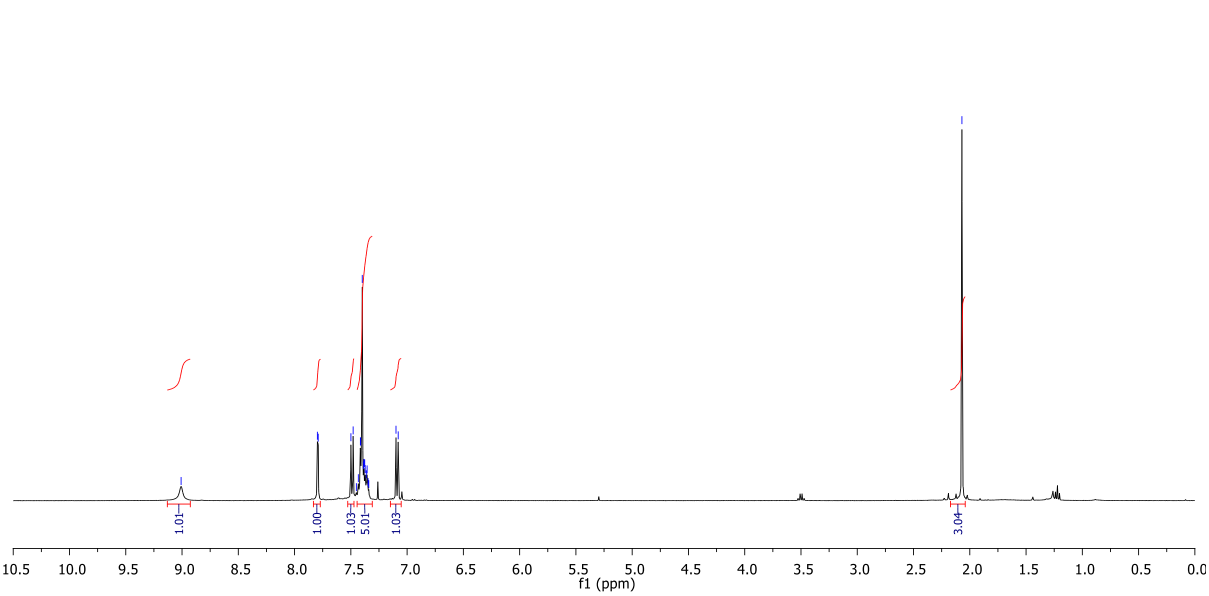

^{13}C{^{1}H} NMR spectrum of 6ca (100 MHz, $CDCl_3$)

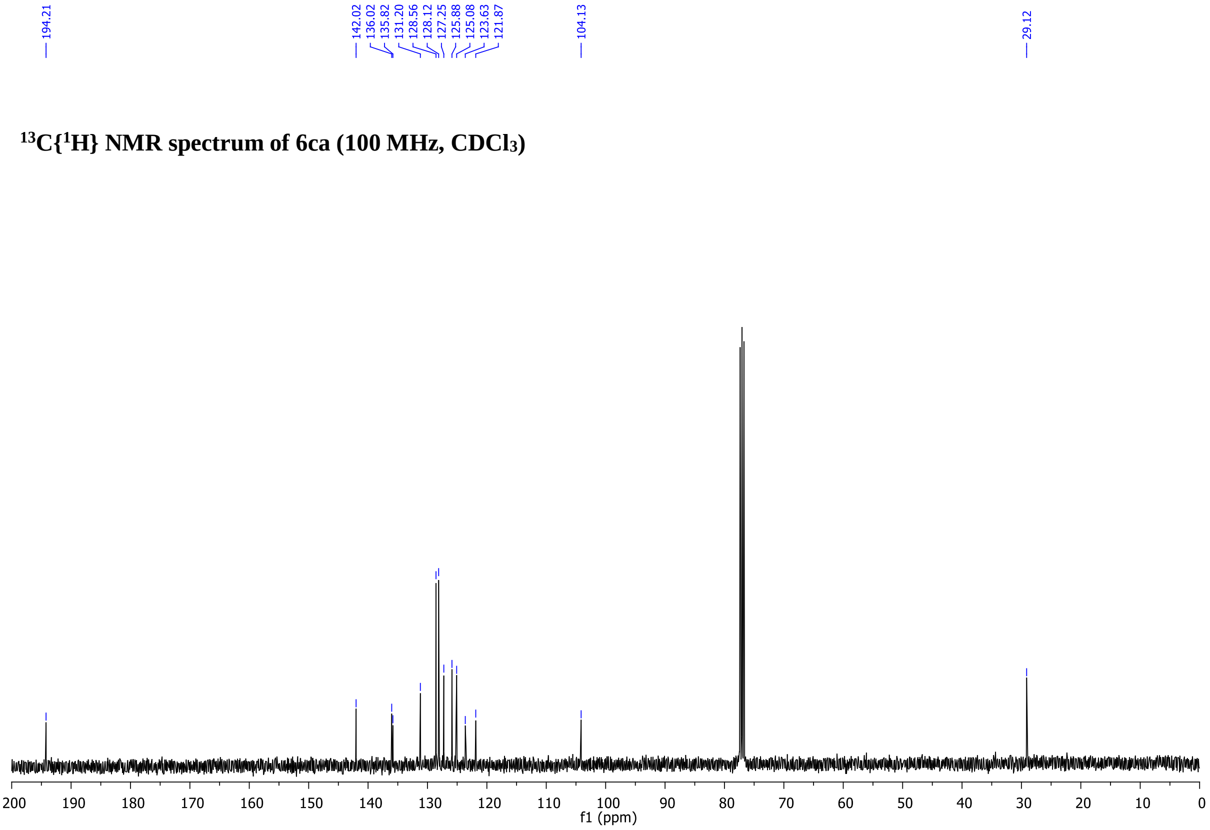

2-Methyl-4-phenyl-1*H*-indole (7da)

^{1}H NMR spectrum of 7da (400 MHz, $CDCl_3$)

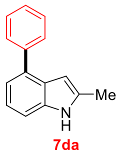

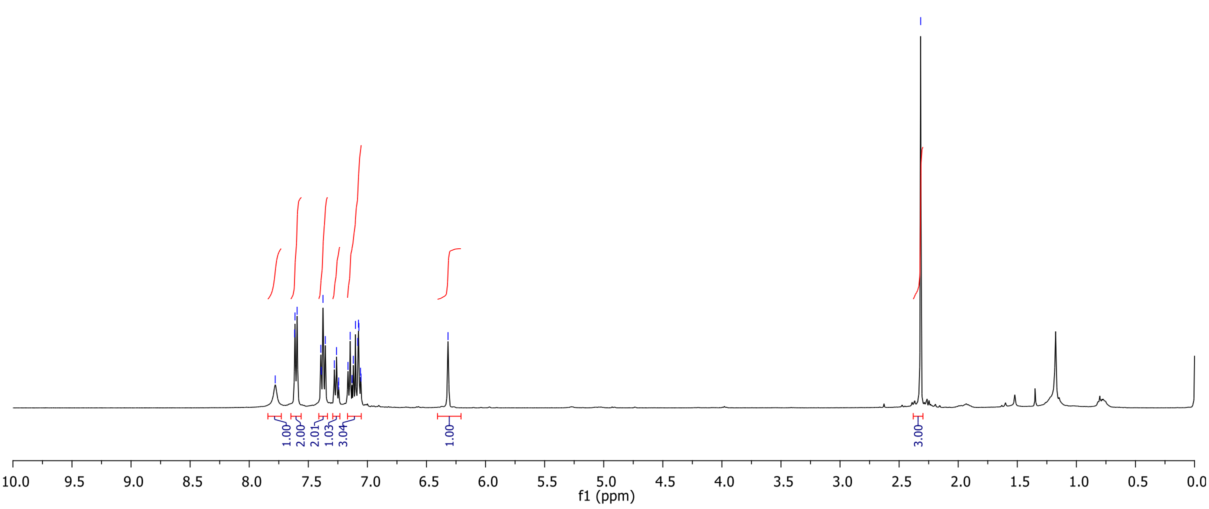

^{13}C{^{1}H} NMR spectrum of 7da (100 MHz, $CDCl_3$)

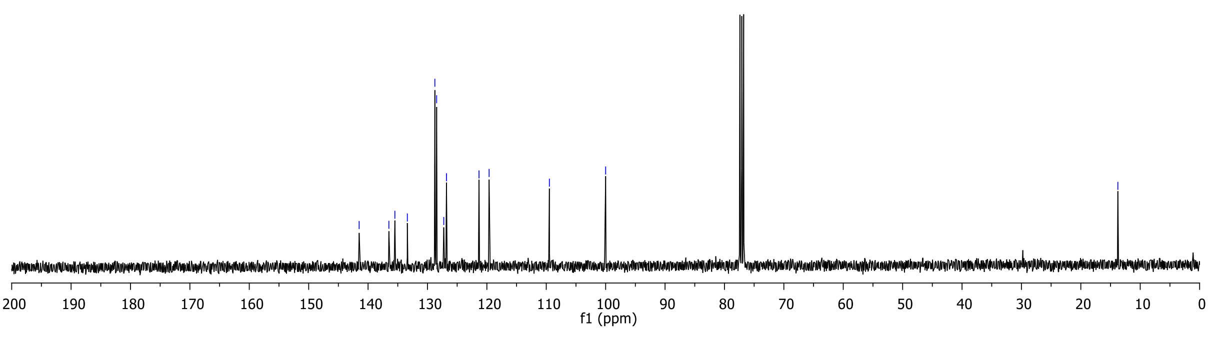

1-(1-Benzyl-1*H*-indol-3-yl)ethan-1-one (8a)

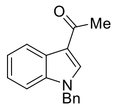

^{1}H NMR spectrum of 8a (400 MHz, $CDCl_3$)

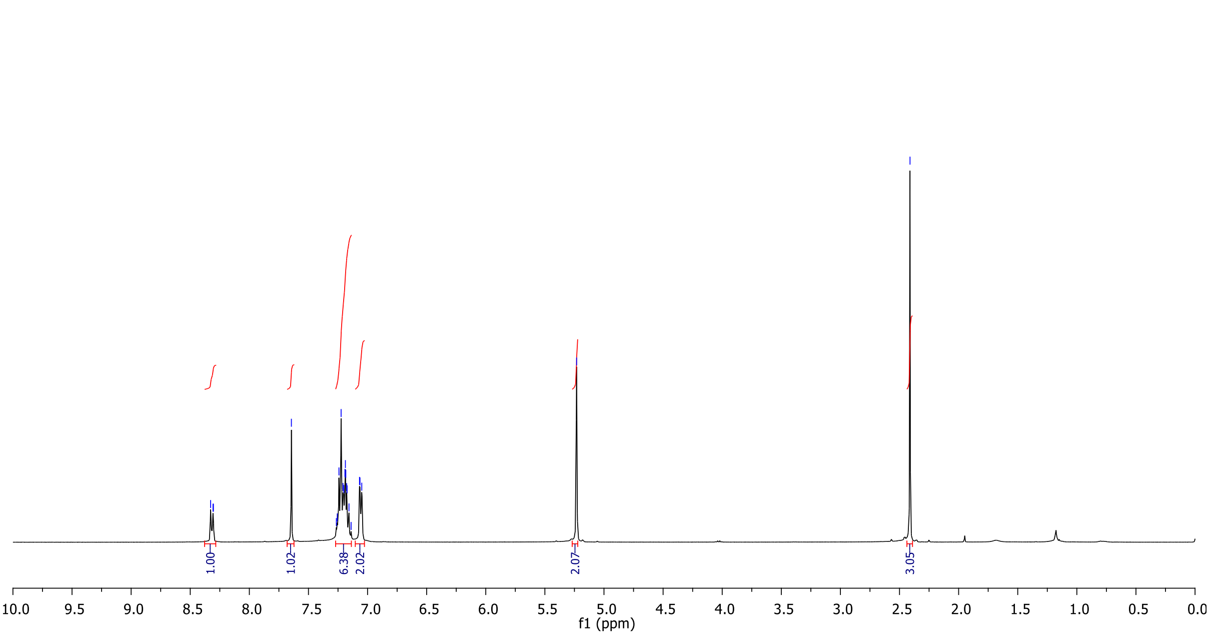

^{13}C{^{1}H} NMR spectrum of 8a (100 MHz, $CDCl_3$)

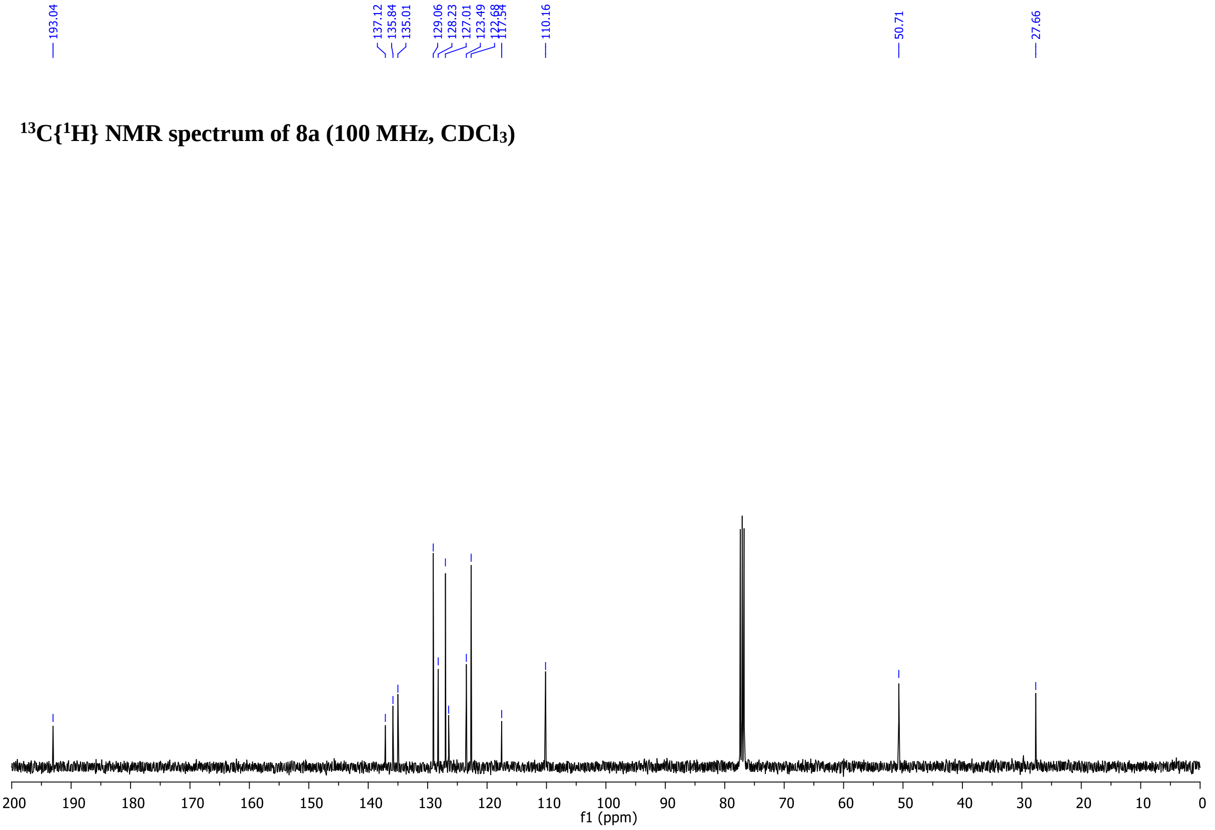

1-(1-Benzyl-7-fluoro-1*H*-indol-3-yl)ethan-1-one (8b)

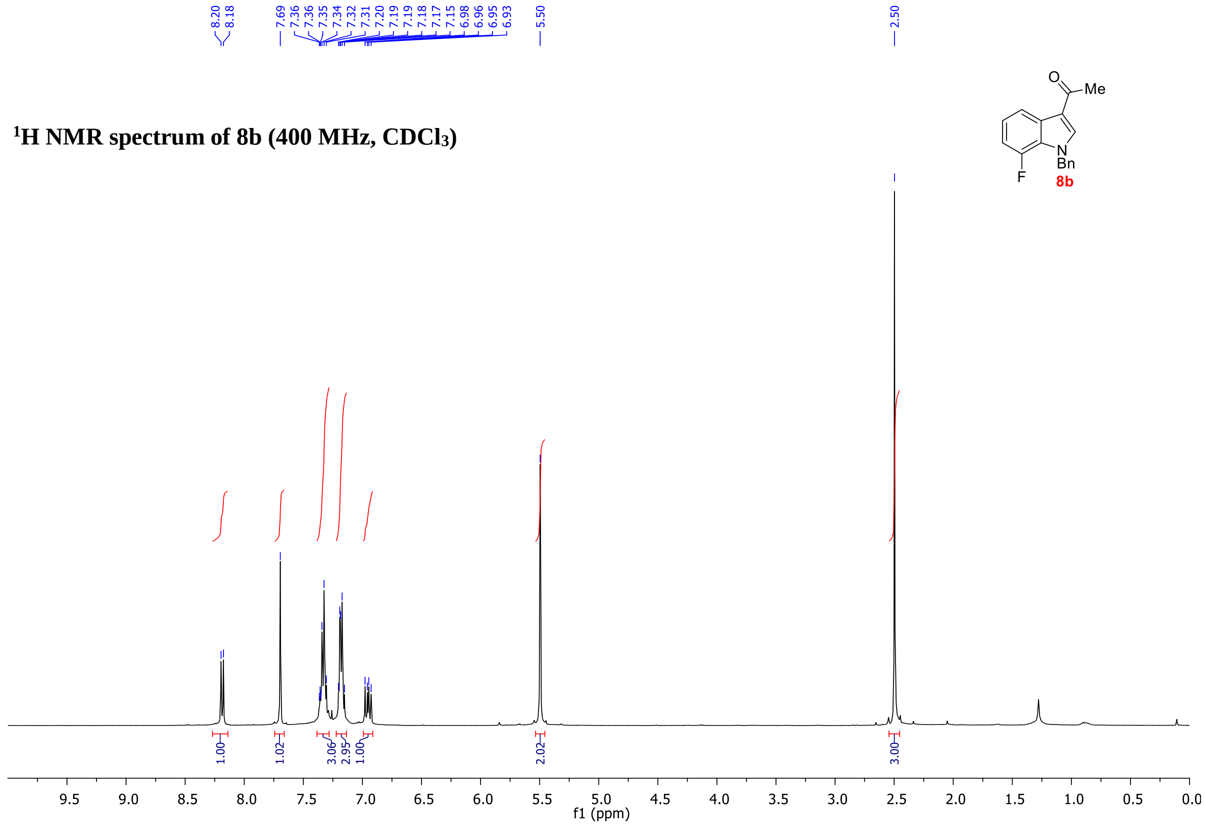

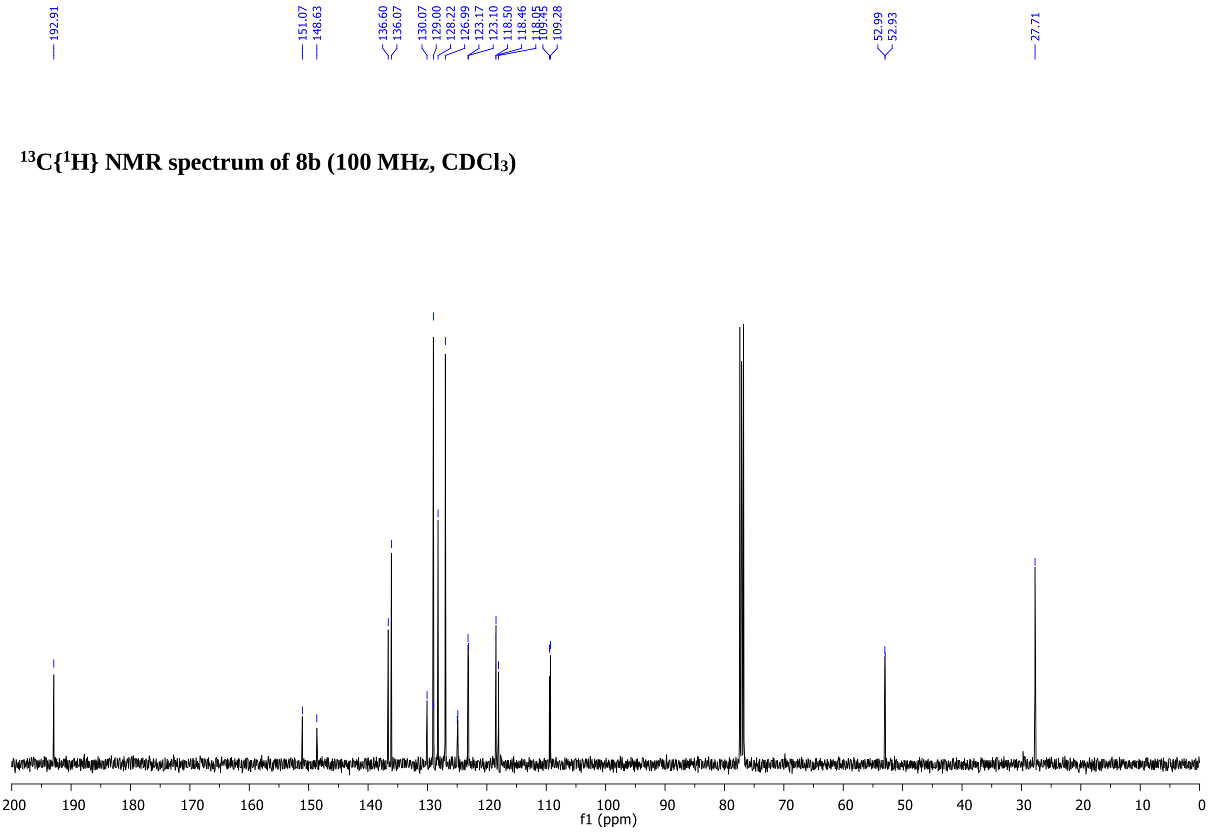

1-(1-Benzyl-7-bromo-1*H*-indol-3-yl)ethan-1-one (8c)

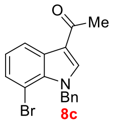

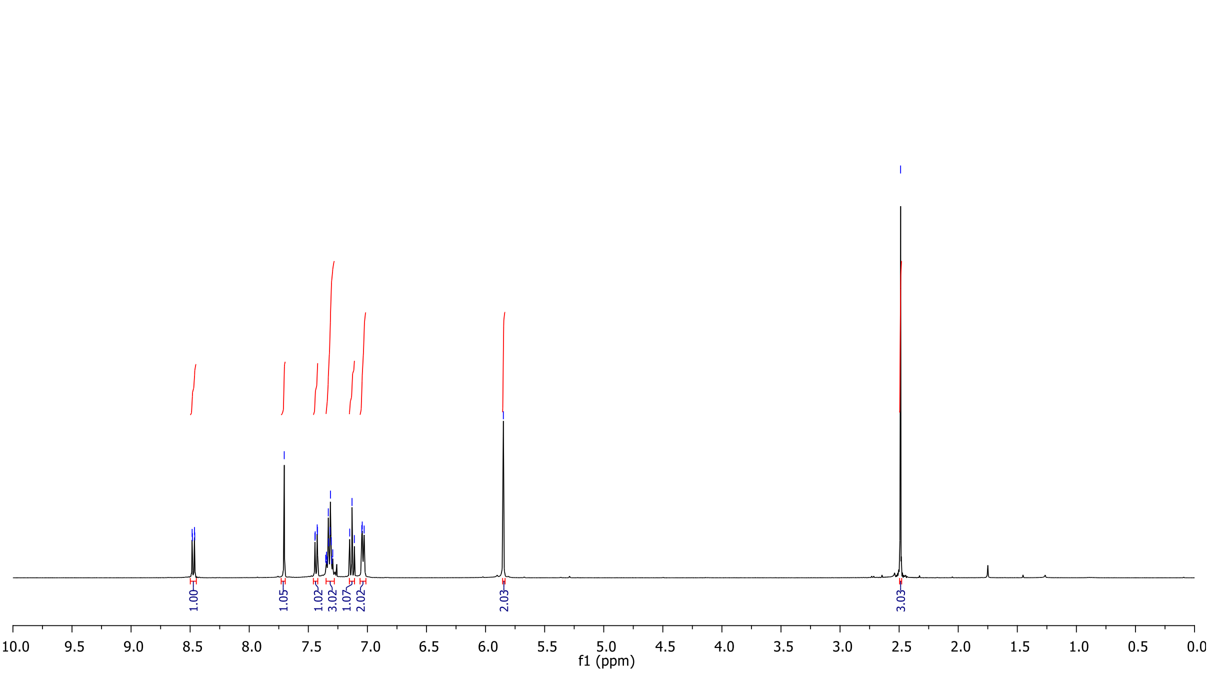

^{1}H NMR spectrum of 8c (400 MHz, $CDCl_3$)

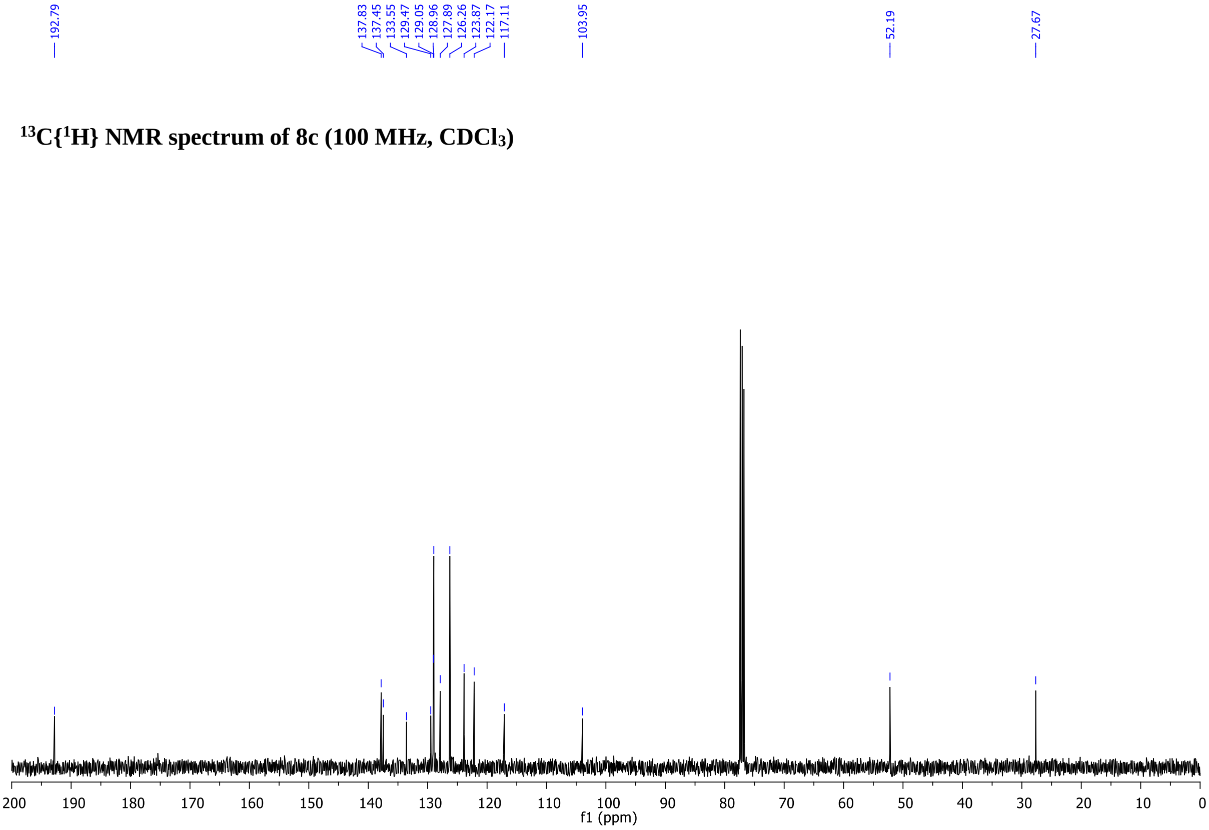

$^{13}C\{^1H\}$ NMR spectrum of 8c (100 MHz, $CDCl_3$)

1-(1-Benzyl-2-methyl-1*H*-indol-3-yl)ethan-1-one (8d)

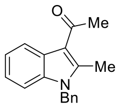

8d

^{1}H NMR spectrum of 8d (400 MHz, $CDCl_3$)

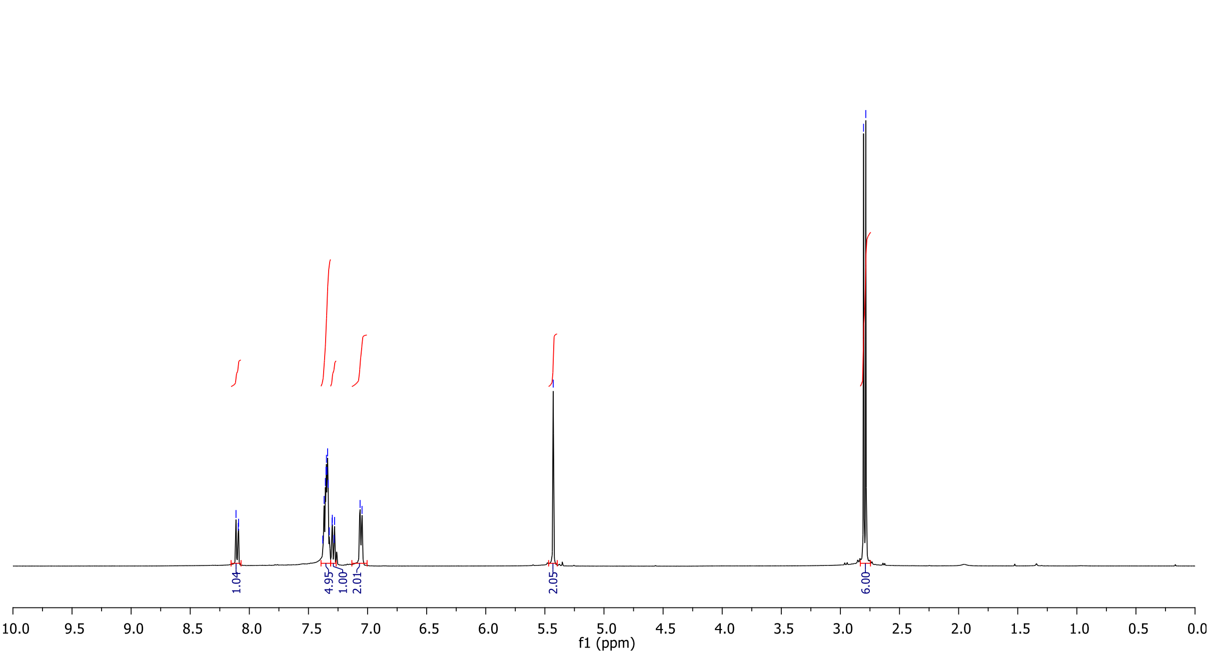

^{13}C{^{1}H} NMR spectrum of 8d (100 MHz, $CDCl_3$)

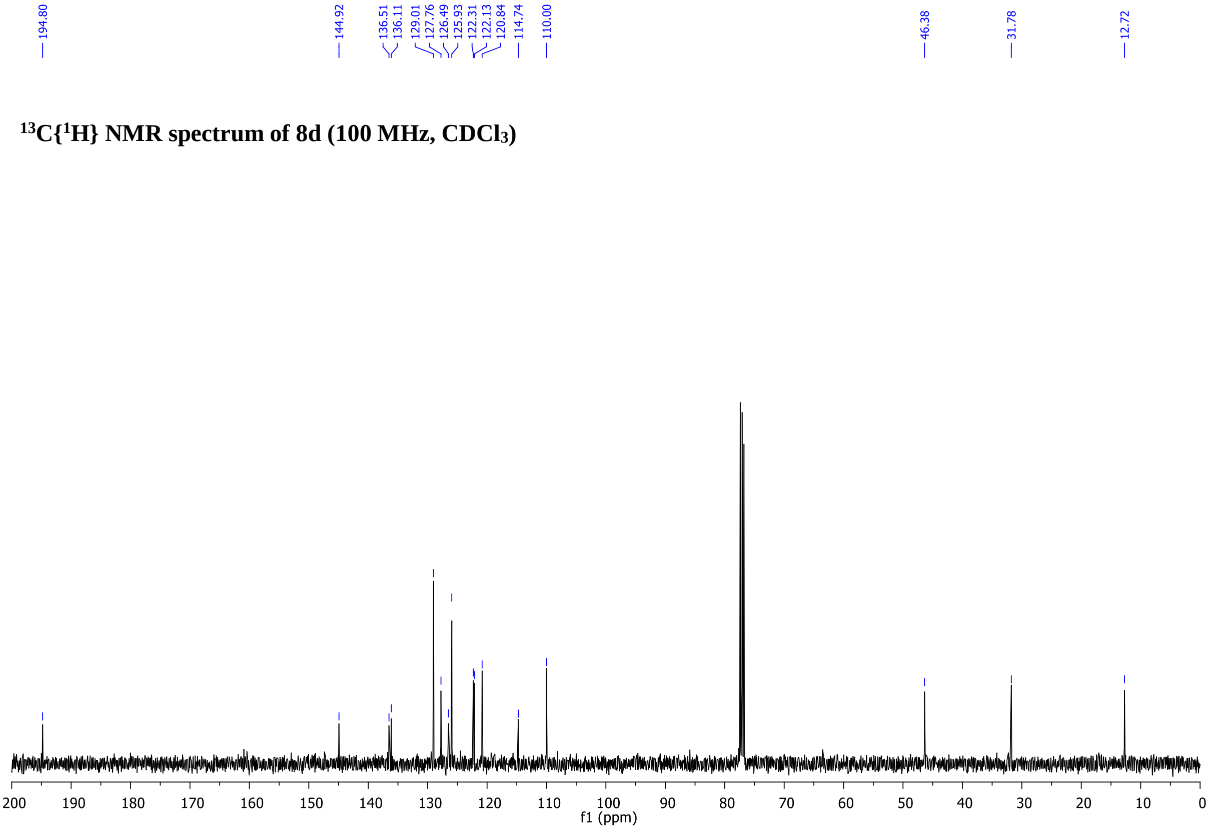

1-(1-Methyl-1*H*-indol-3-yl)ethan-1-one (8e)

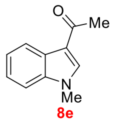

8e

^{1}H NMR spectrum of 8e (400 MHz, $CDCl_3$)

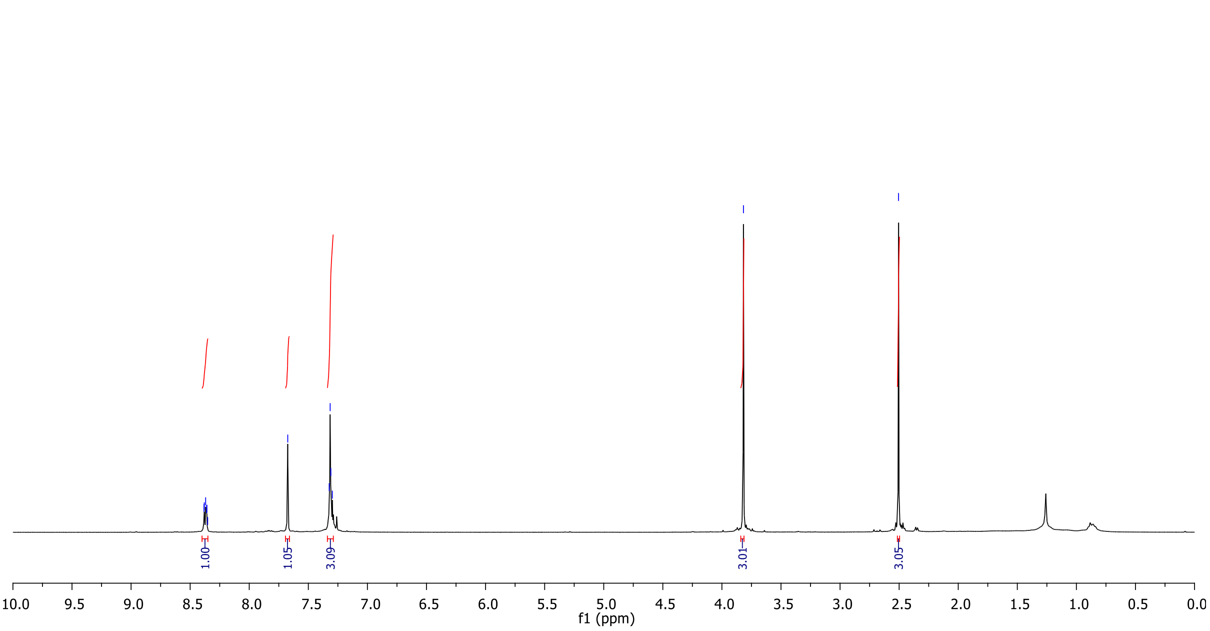

^{13}C{^{1}H} NMR spectrum of 8e (100 MHz, $CDCl_3$)

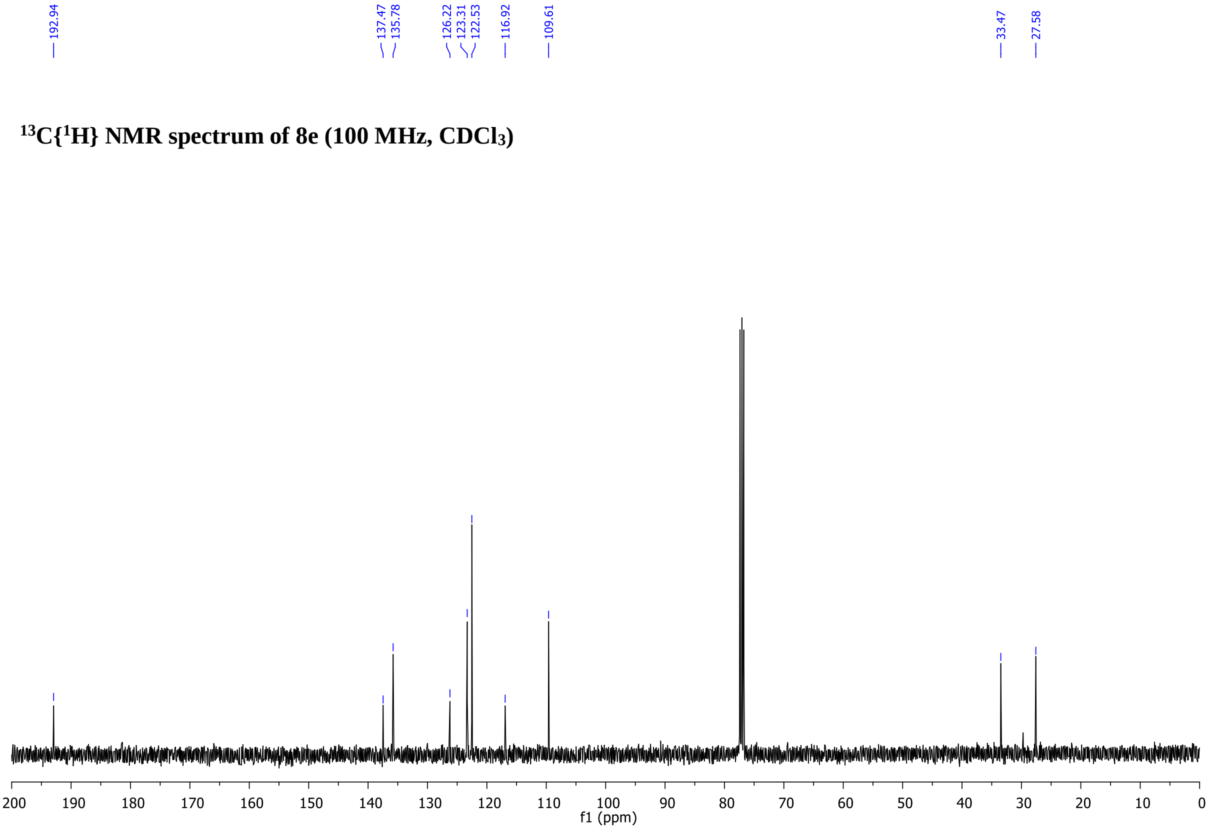

1-(1-Benzyl-4-phenyl-1*H*-indol-3-yl)ethan-1-one (9aa)

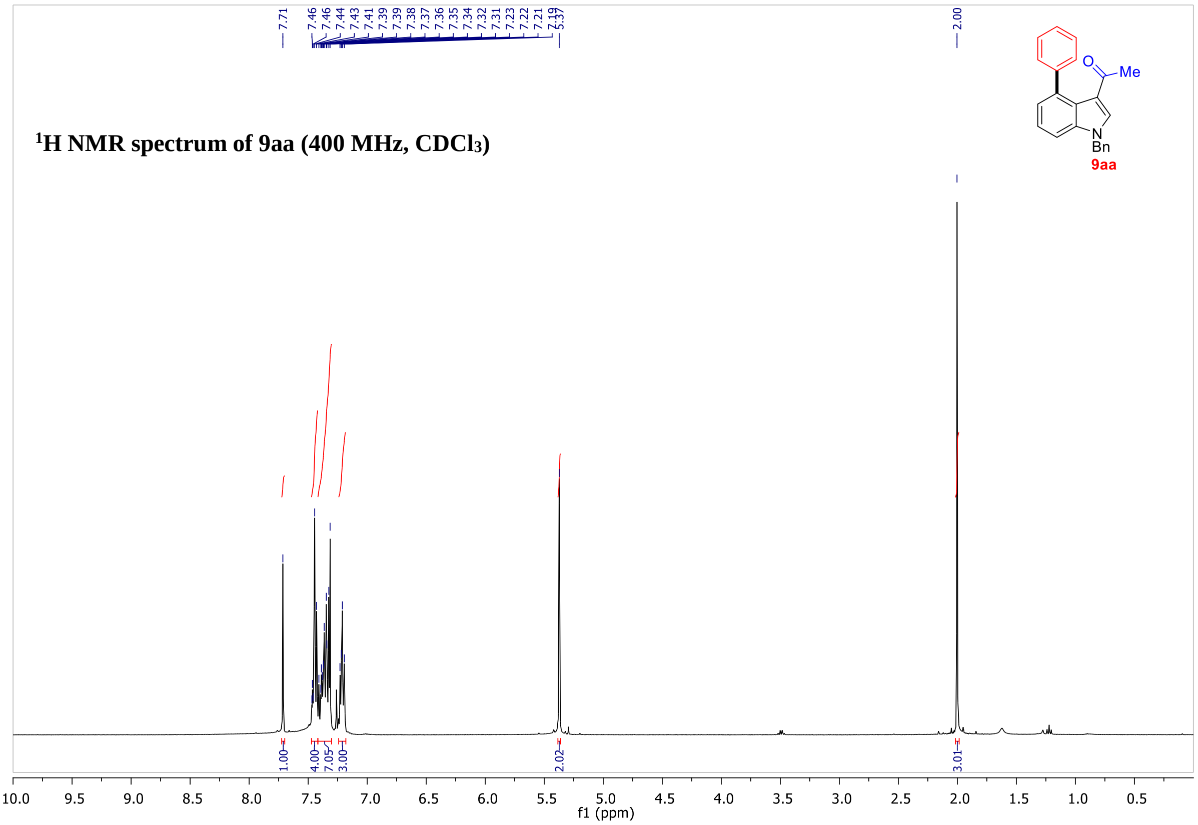

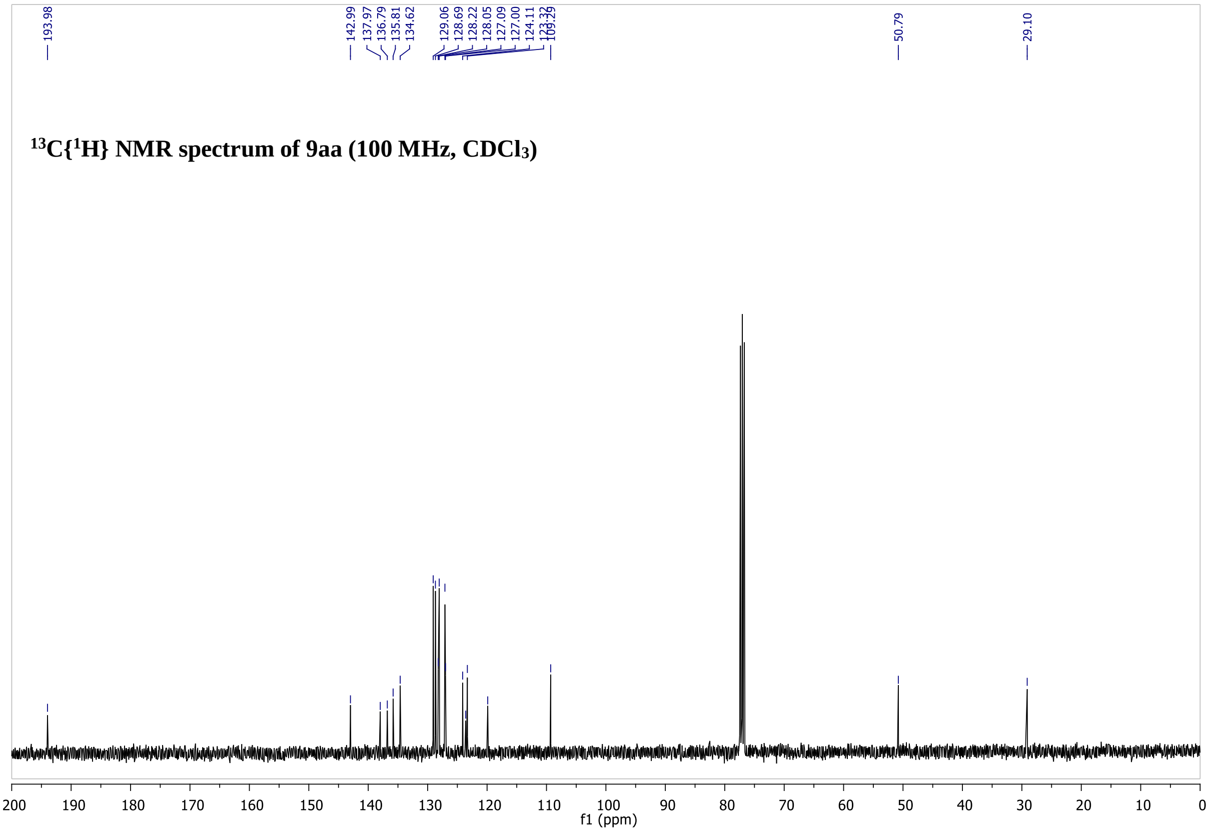

1-(1-Benzyl-4-(*p*-tolyl)-1*H*-indol-3-yl)ethan-1-one (9ab)

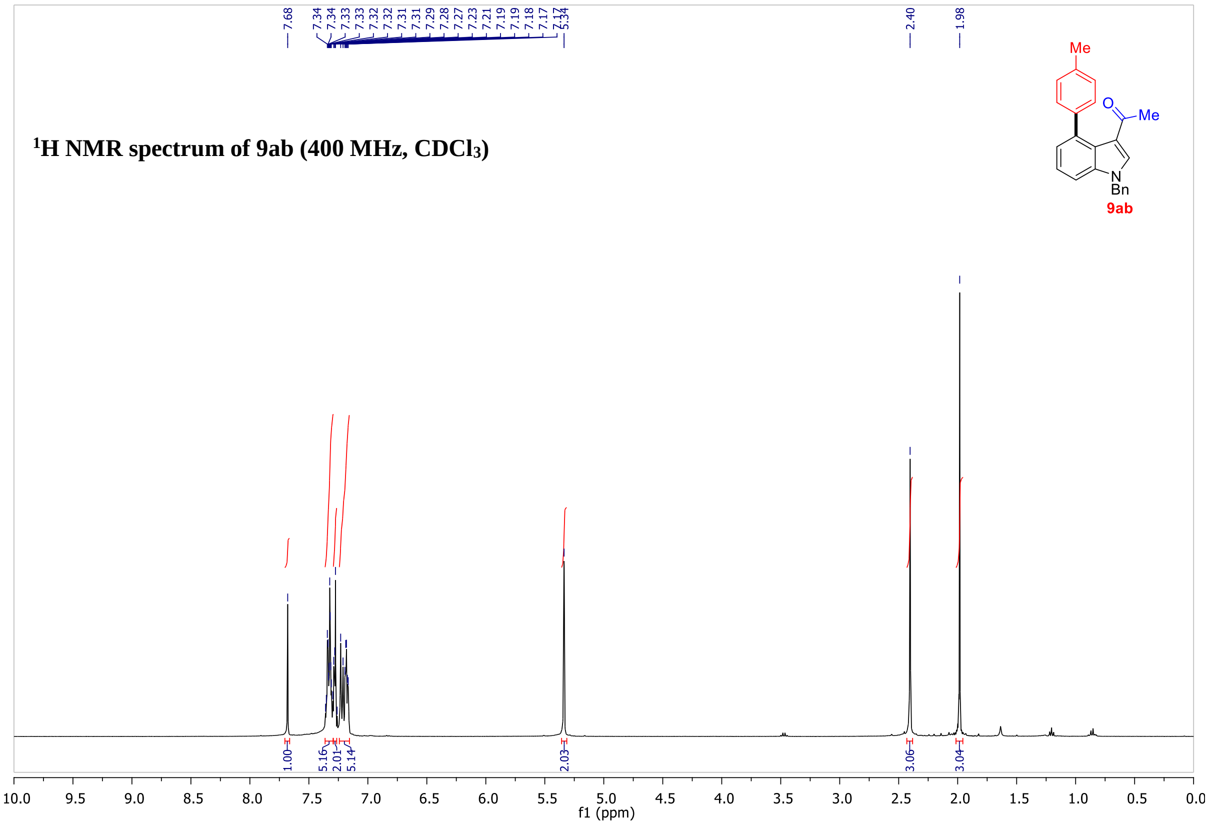

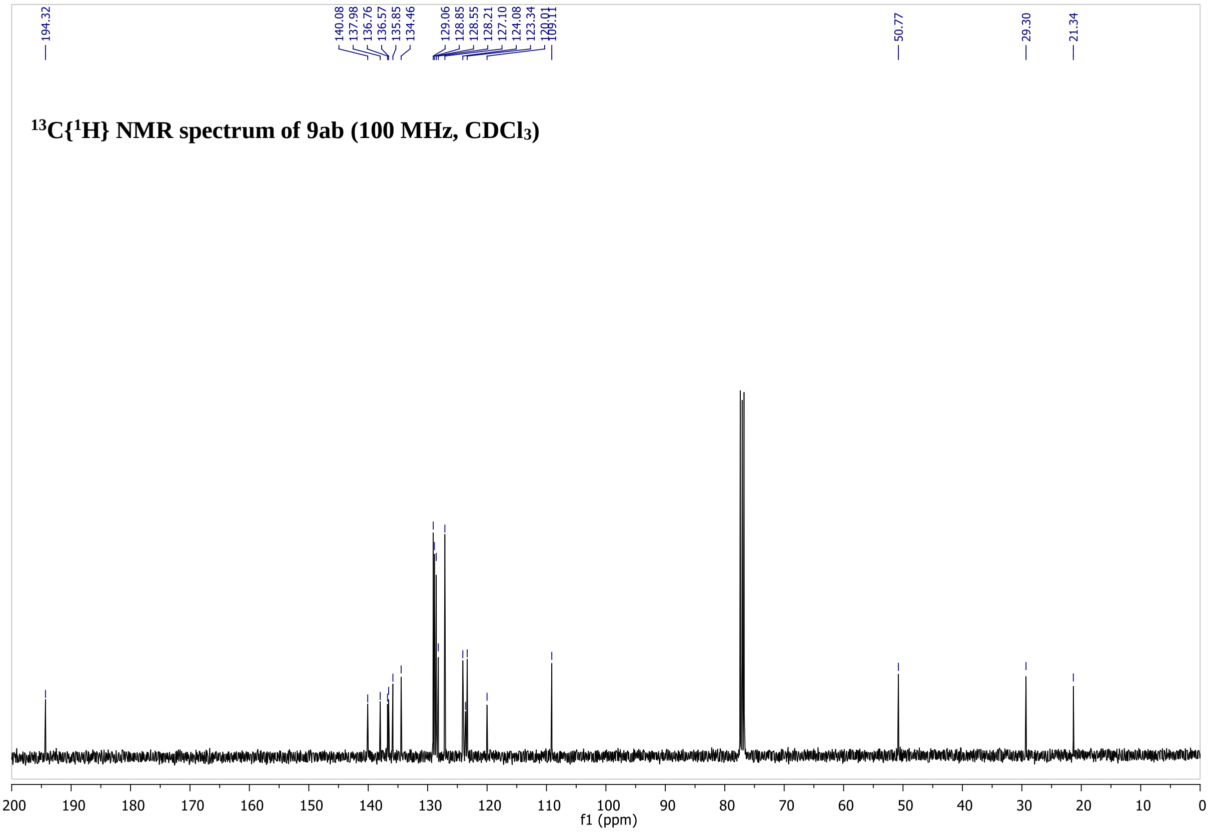

1-(1-Benzyl-4-(*m*-Tolyl)-1*H*-indol-3-yl)ethan-1-one (9ac)

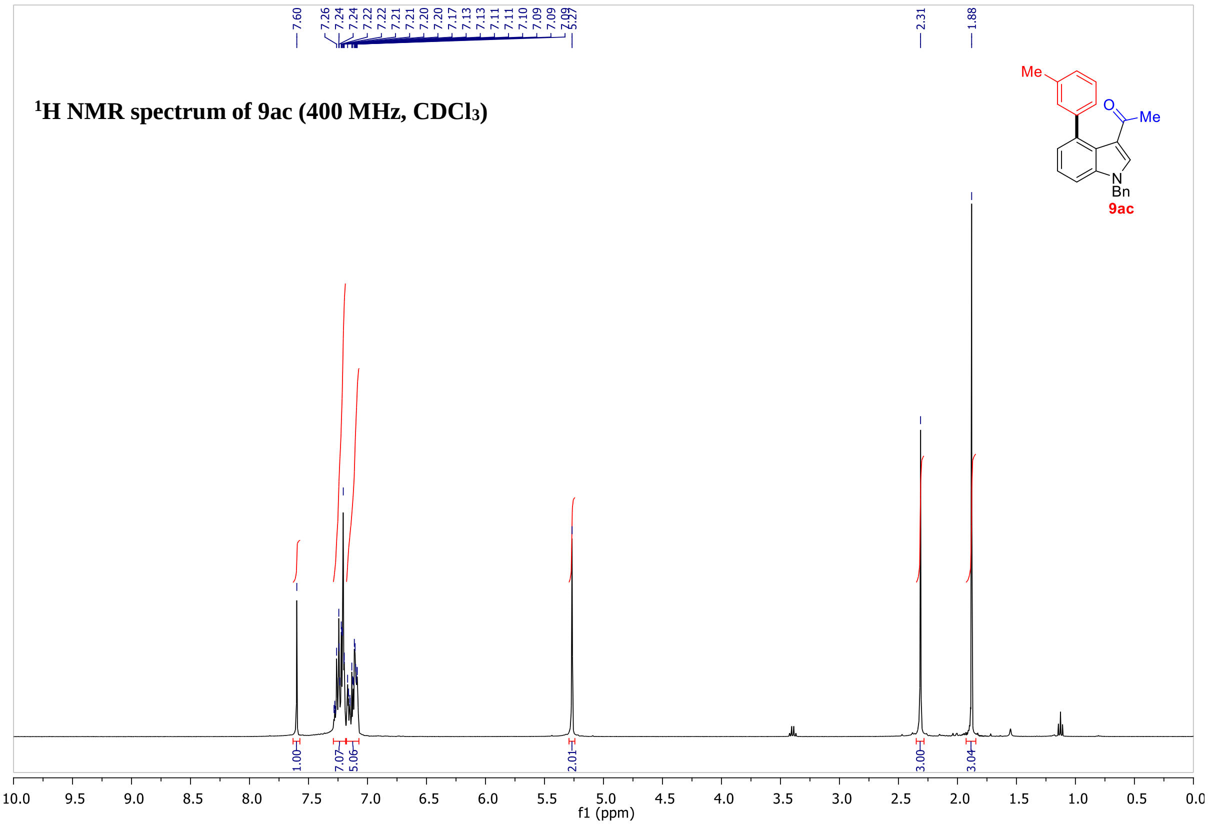

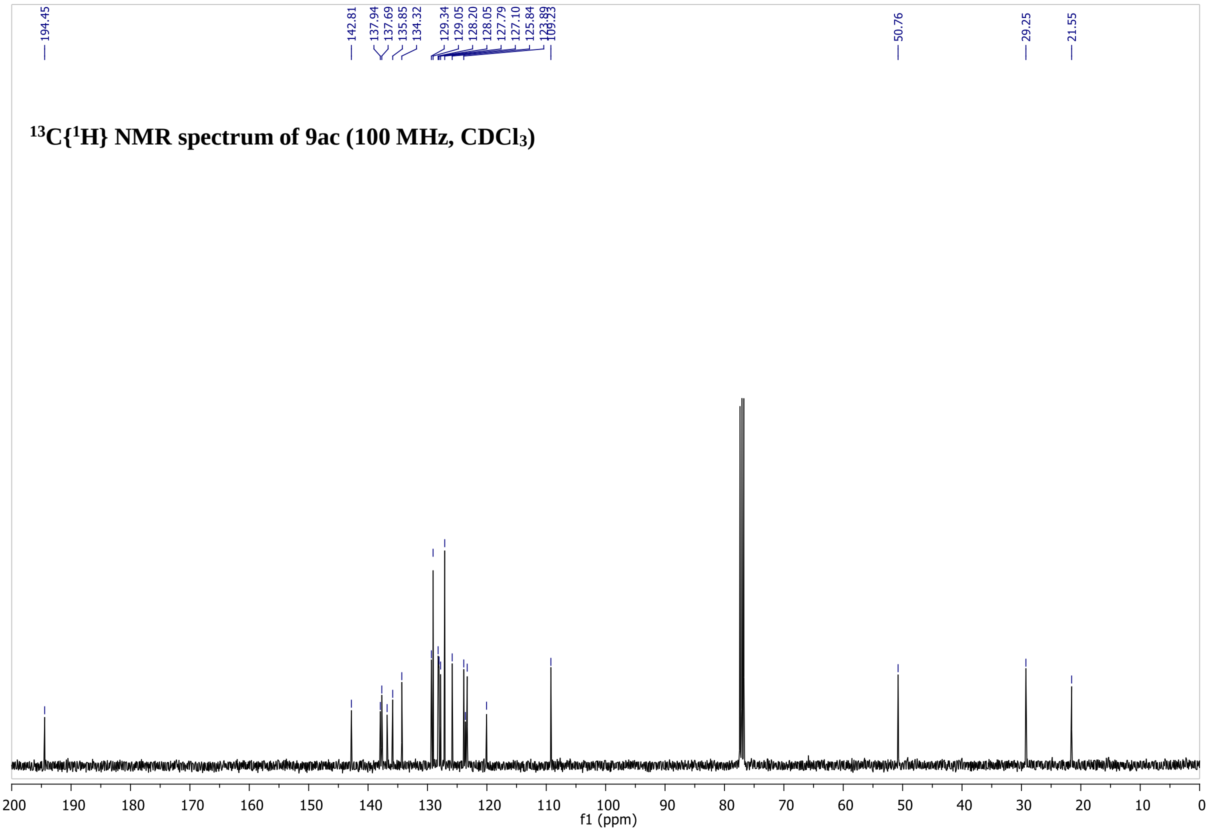

1-(1-Benzyl-4-(4-(*tert*-butyl)phenyl)-1*H*-indol-3-yl)ethan-1-one (9ad)

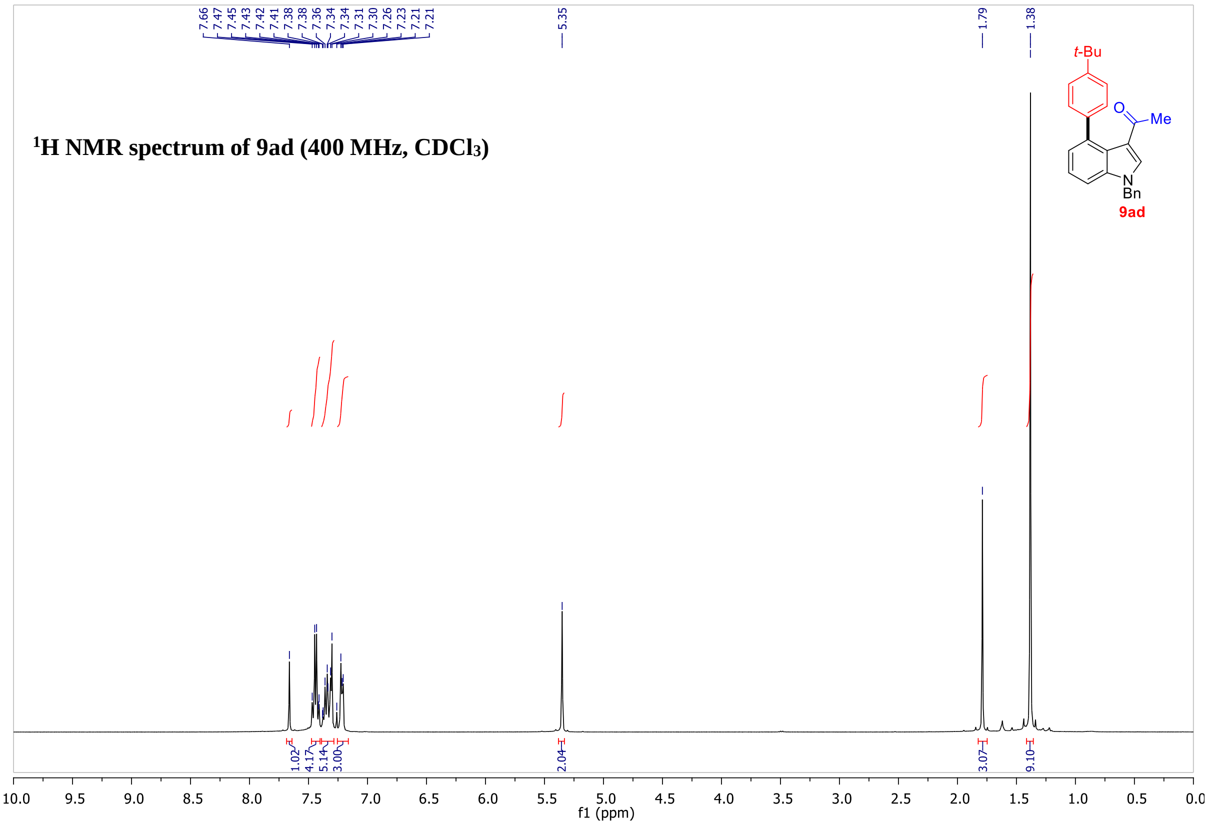

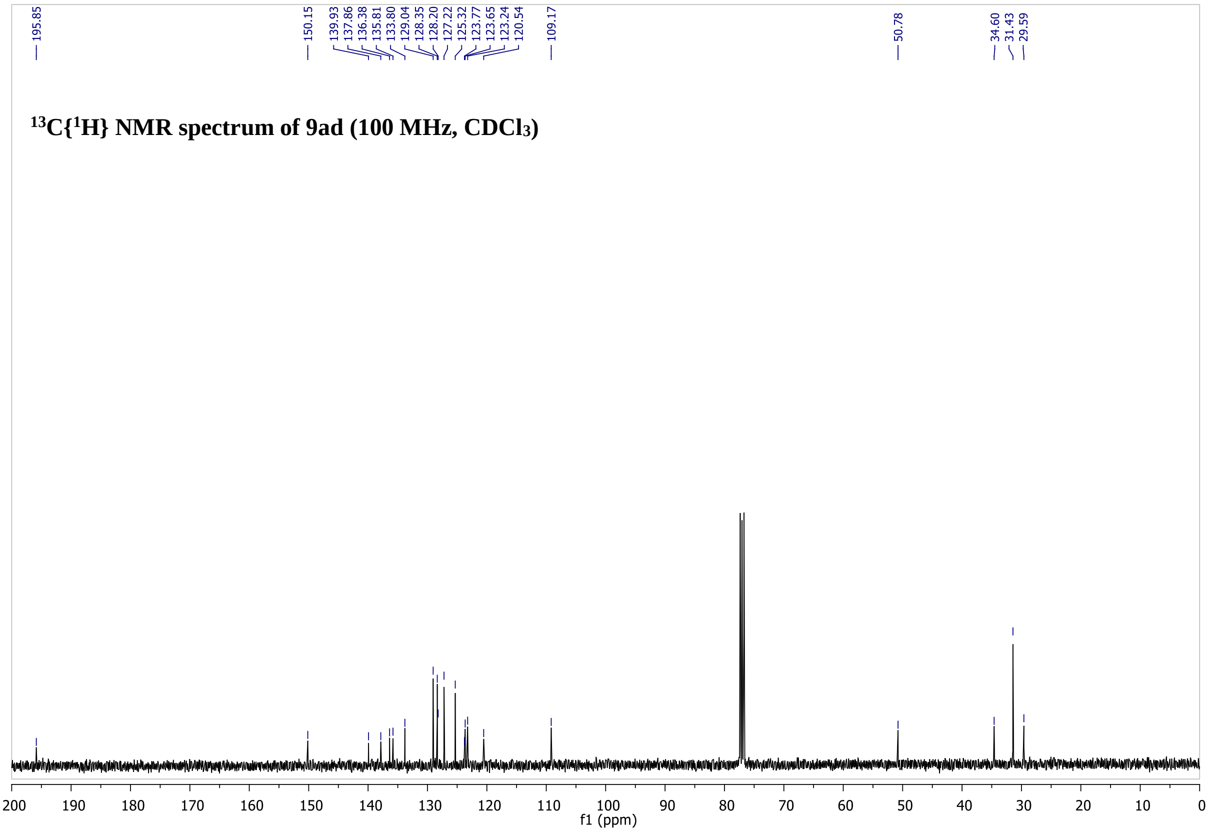

1-(1-Benzyl-4-(4-bromophenyl)-1*H*-indol-3-yl)ethan-1-one (9ae)

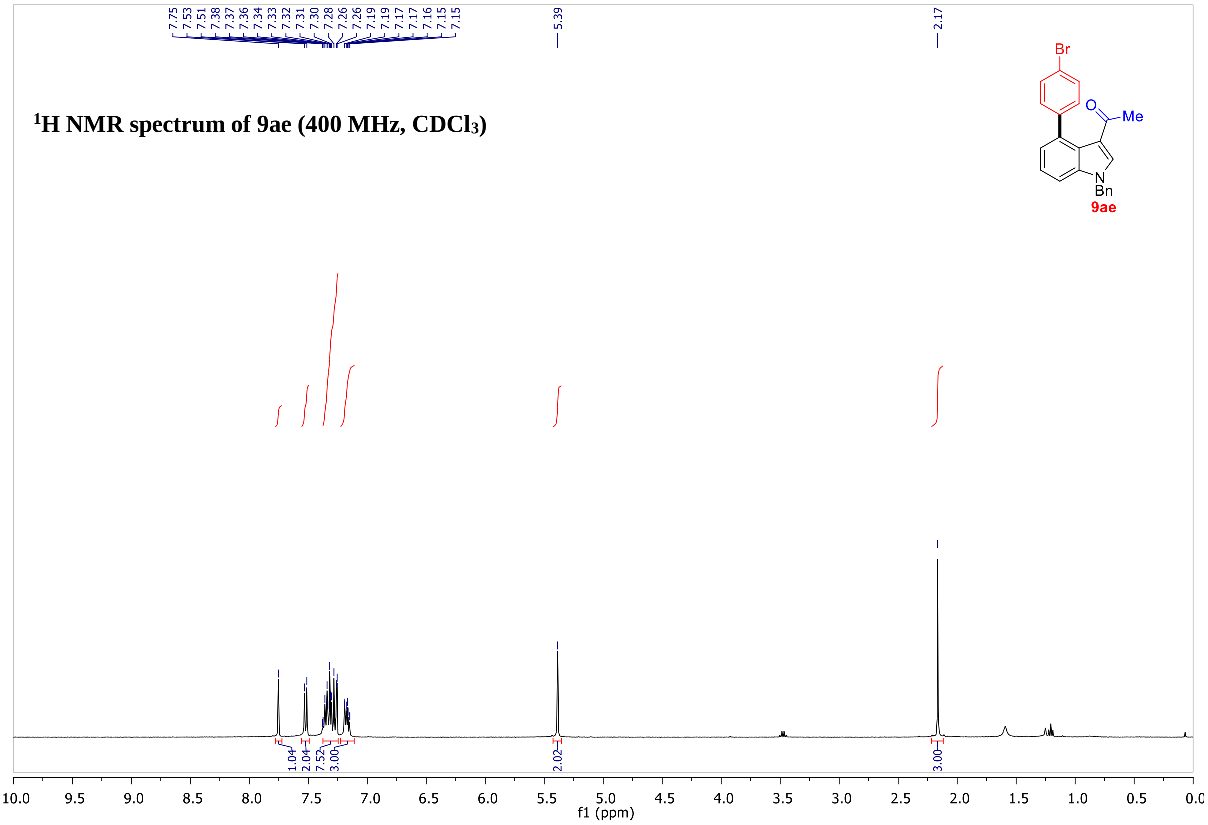

^{1}H NMR spectrum of 9ae (400 MHz, $CDCl_3$)

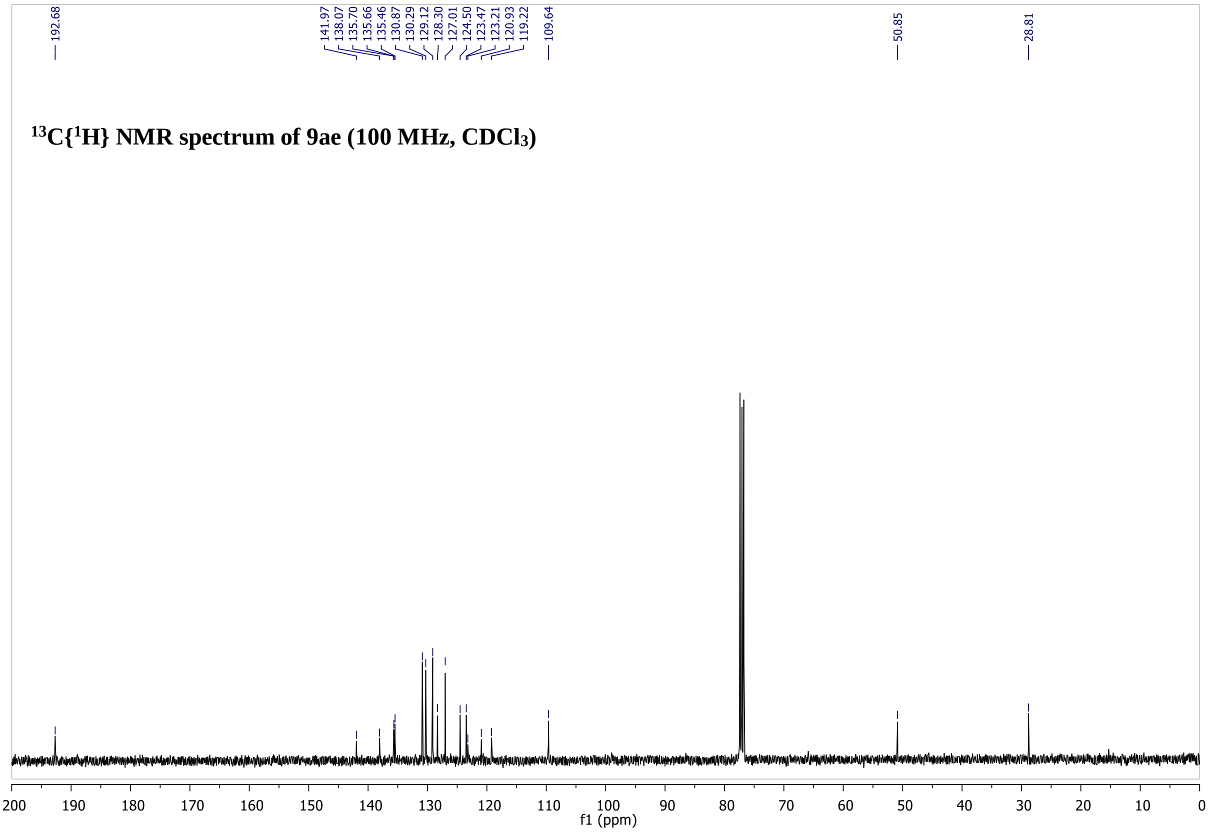

$^{13}C\{^1H\}$ NMR spectrum of 9ae (100 MHz, $CDCl_3$)

1-(1-Benzyl-4-(4-methoxyphenyl)-1*H*-indol-3-yl)ethan-1-one (9af)

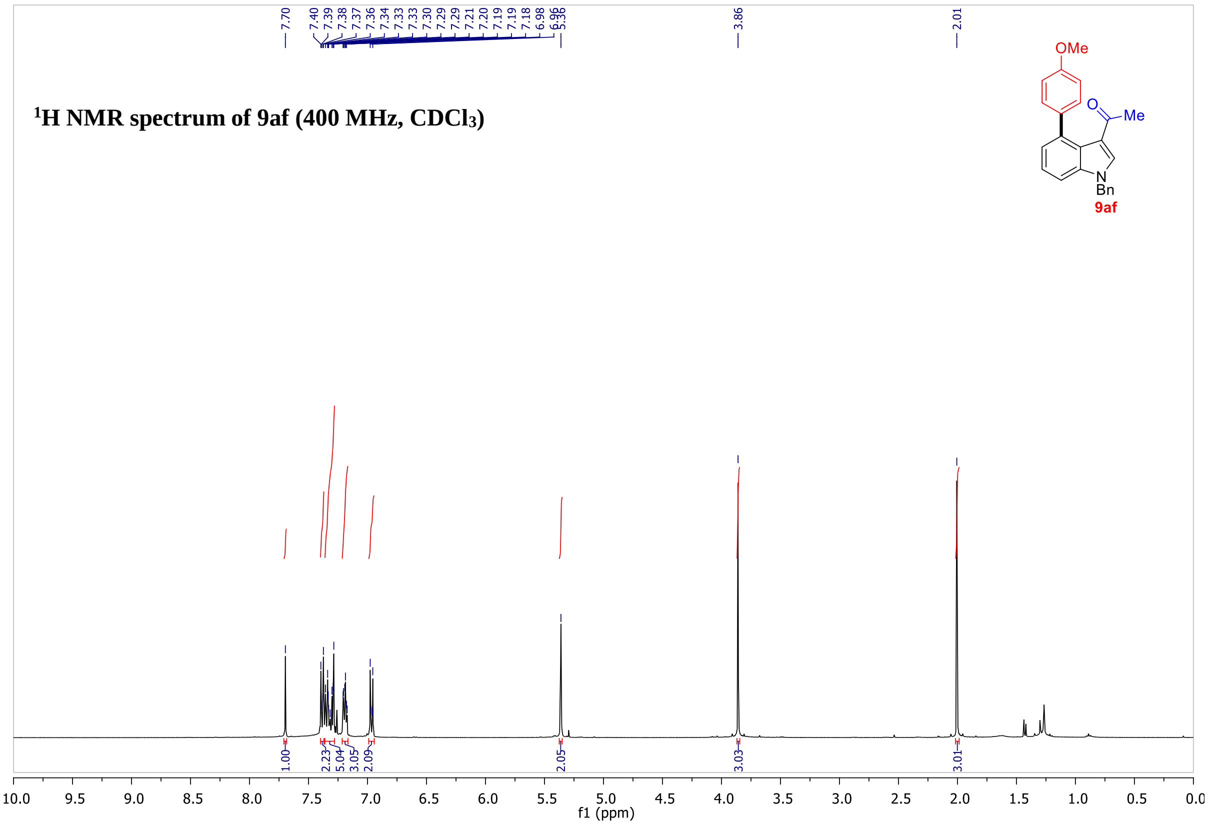

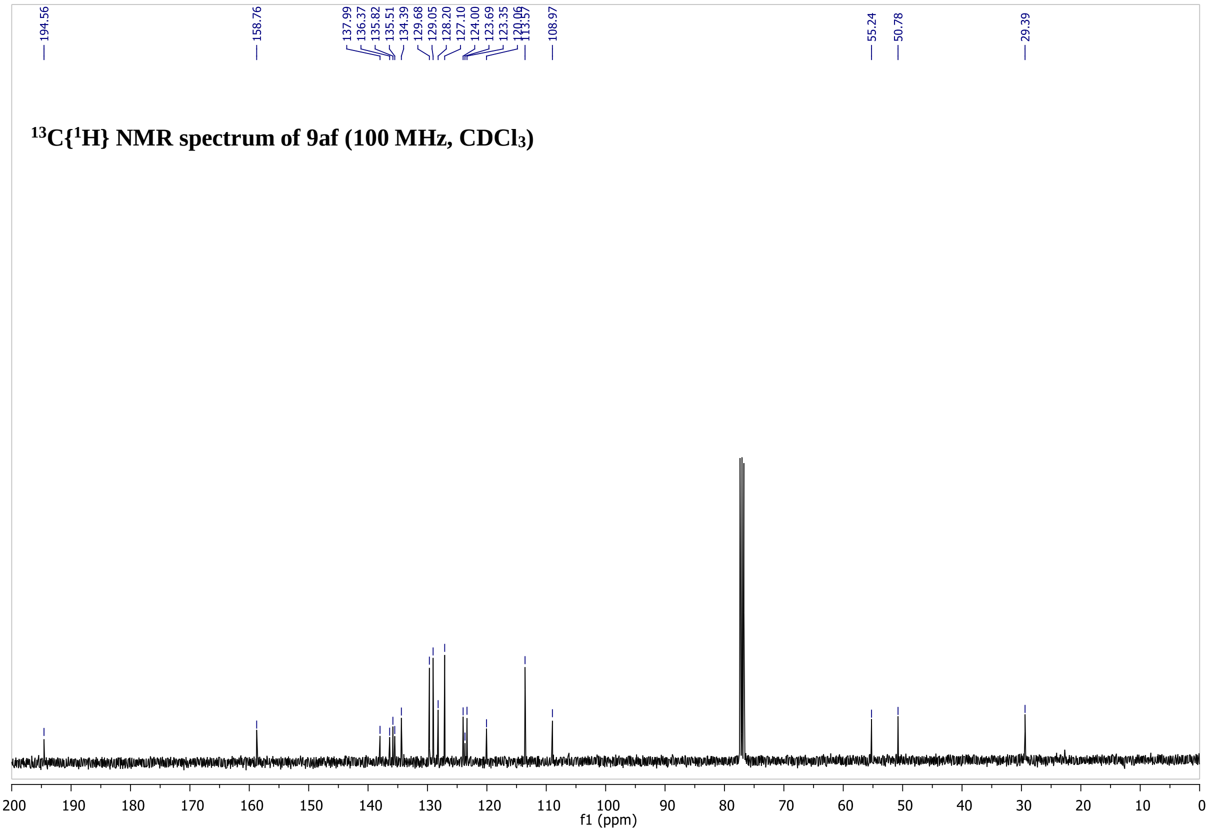

Methyl 4-(3-acetyl-1-benzyl-1*H*-indol-4-yl)benzoate (9ag)

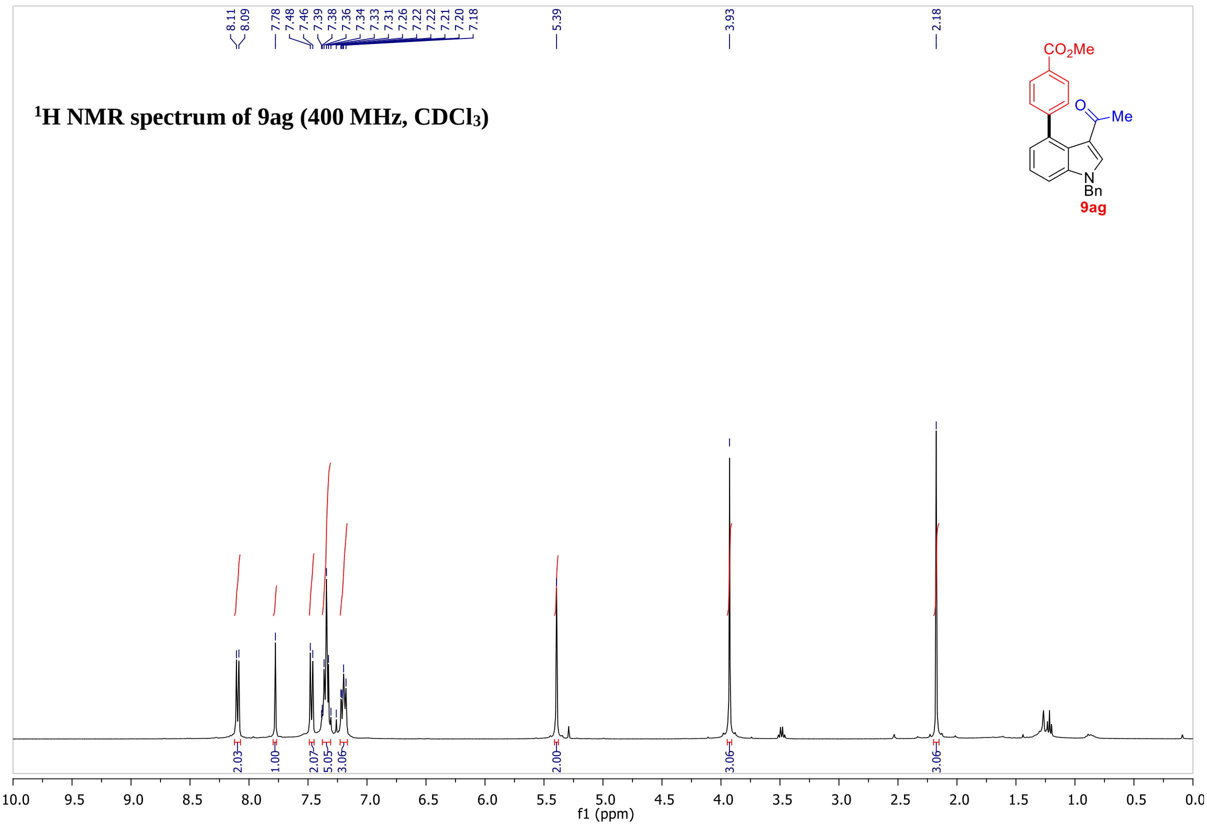

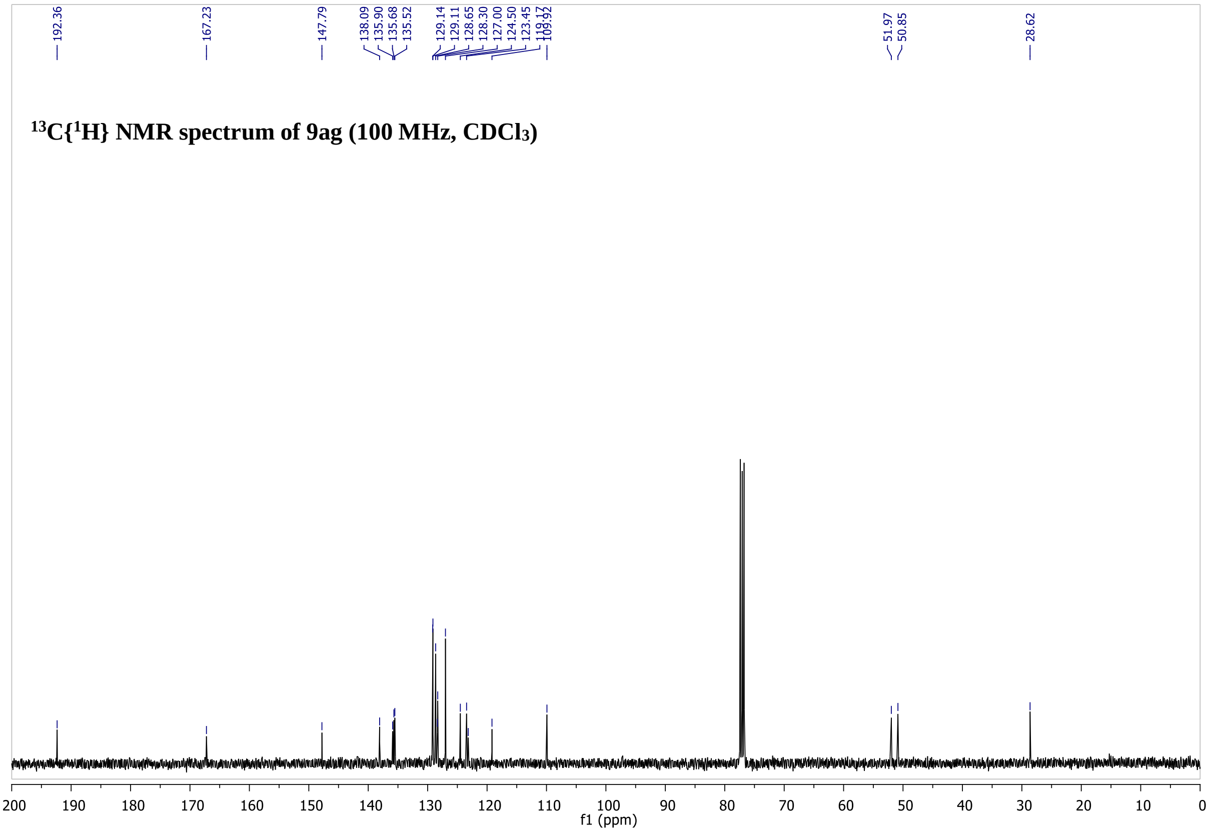

1-(4-(3-Acetyl-1-benzyl-1*H*-indol-4-yl)phenyl)ethan-1-one (9ah)

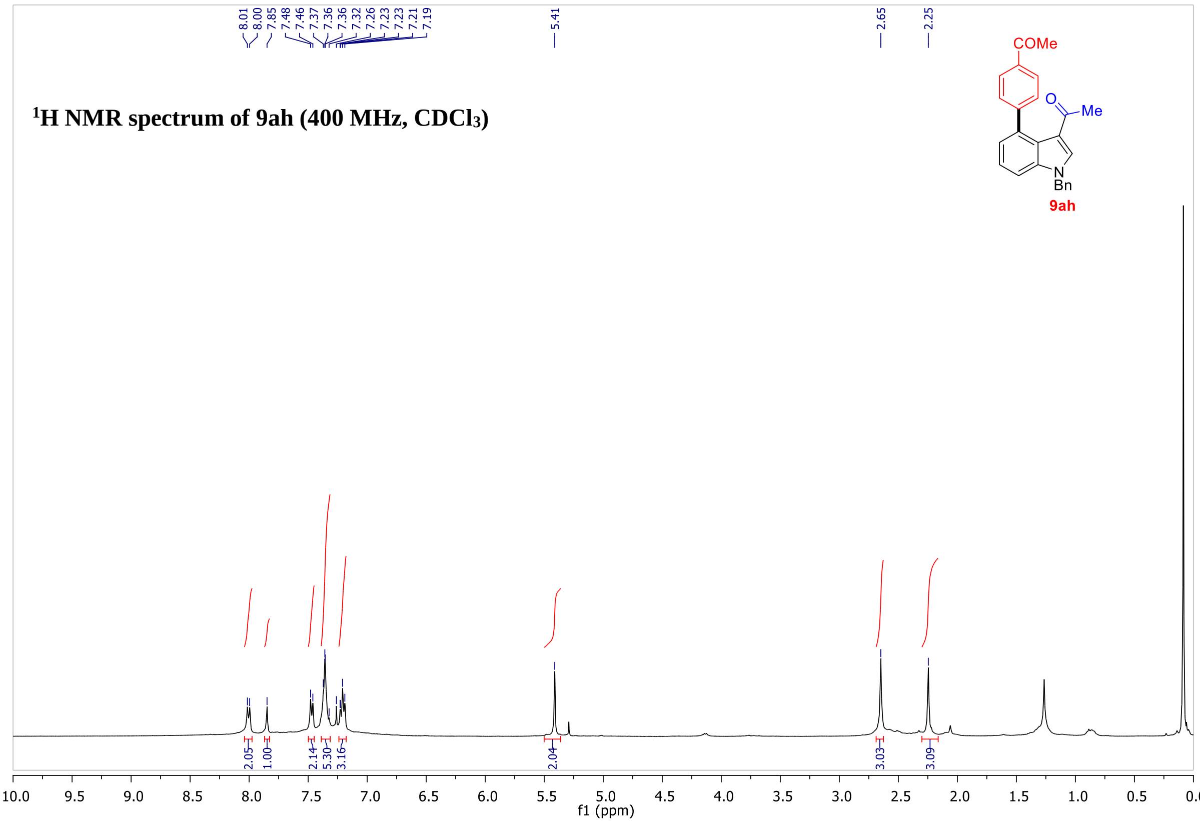

^{1}H NMR spectrum of 9ah (400 MHz, $CDCl_3$)

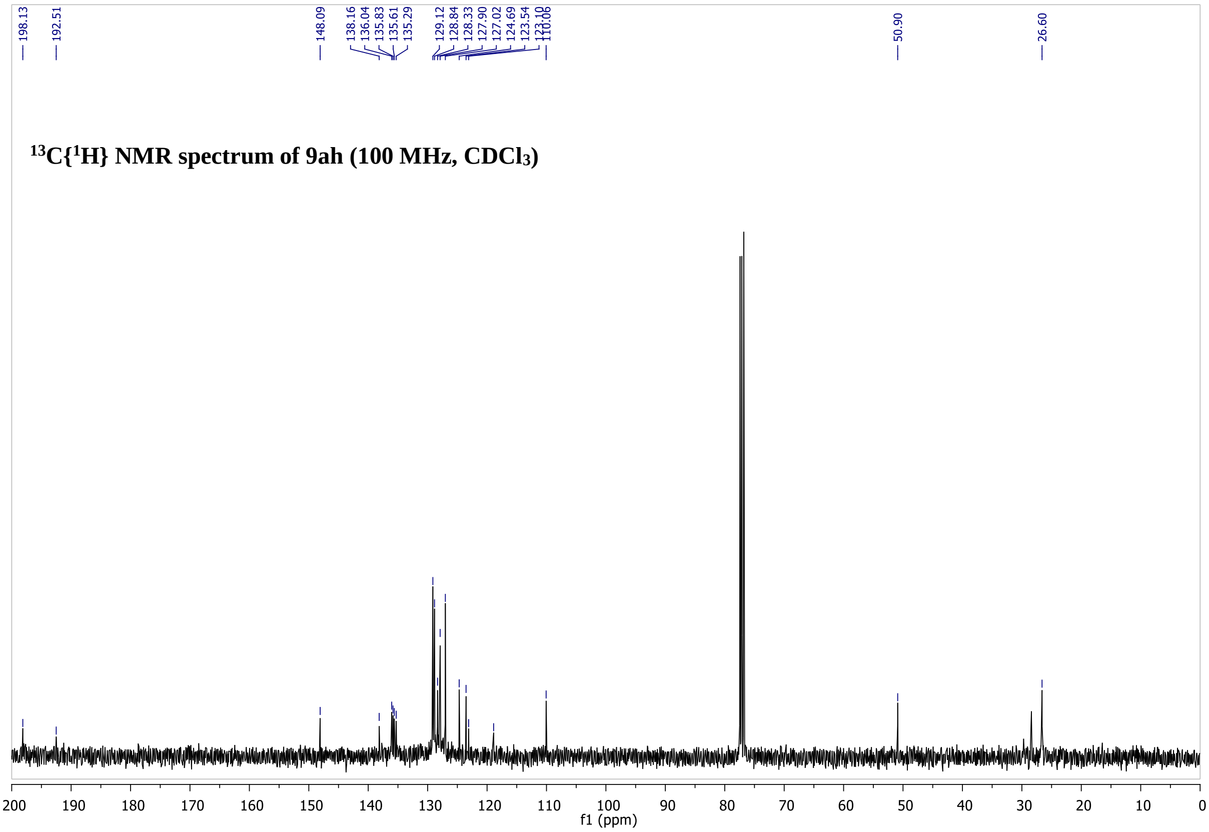

^{13}C{^{1}H} NMR spectrum of 9ah (100 MHz, $CDCl_3$)

1-(1-Benzyl-4-(4-(trifluoromethyl)phenyl)-1*H*-indol-3-yl)ethan-1-one (9ai)

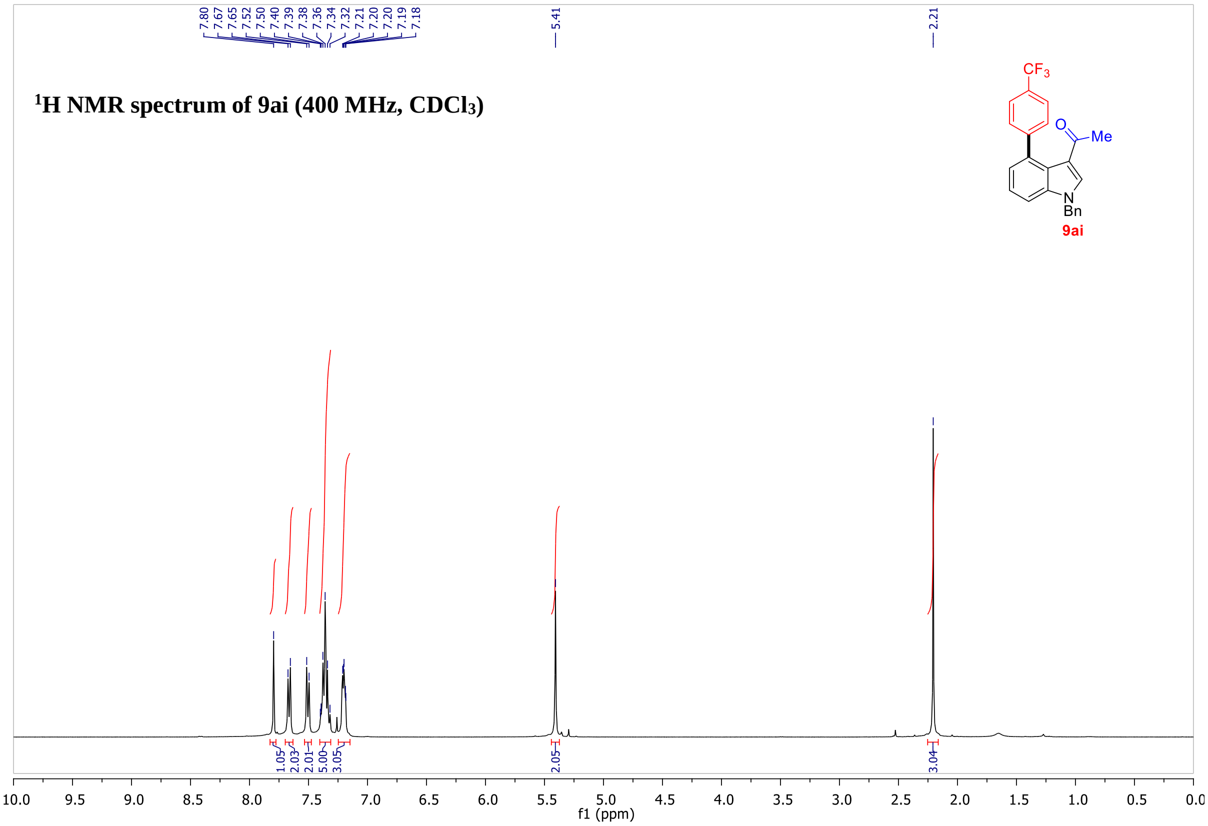

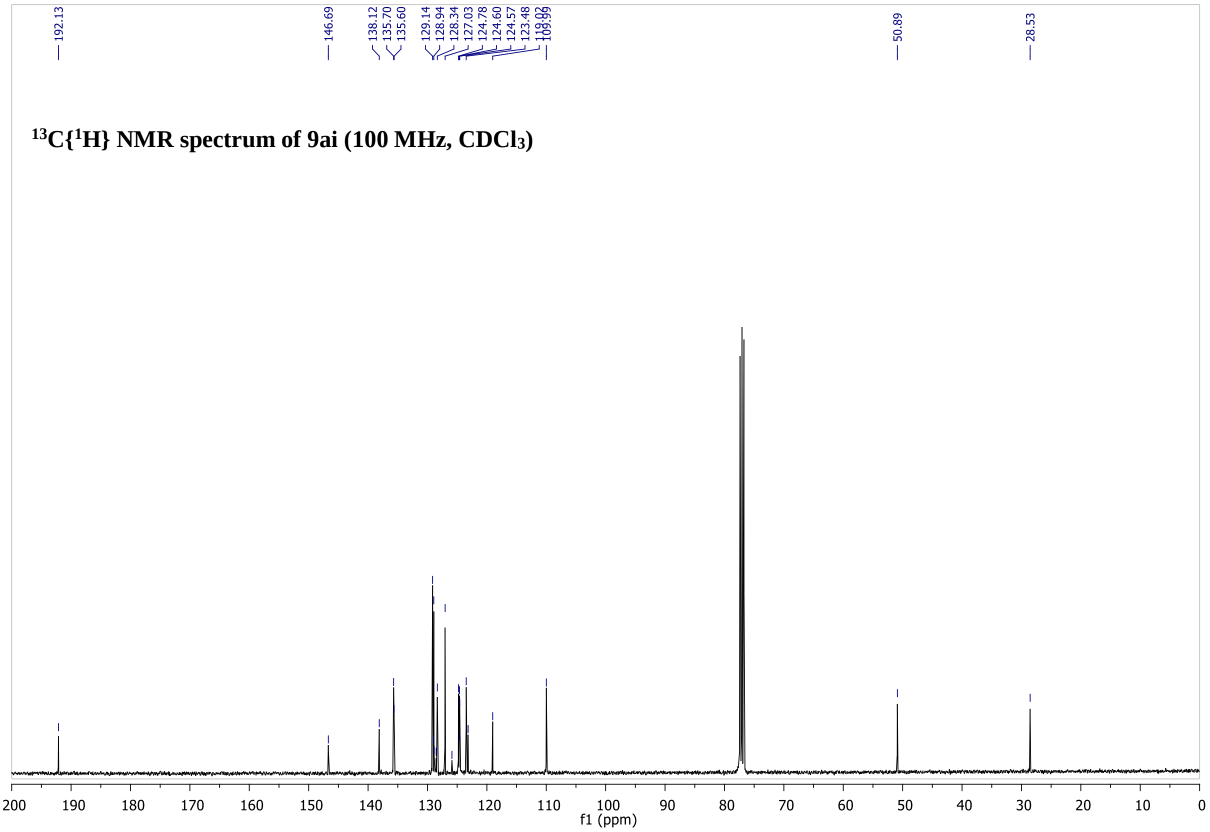

1-(1-Benzyl-4-(3,4-dimethylphenyl)-1*H*-indol-3-yl)ethan-1-one (9ak)

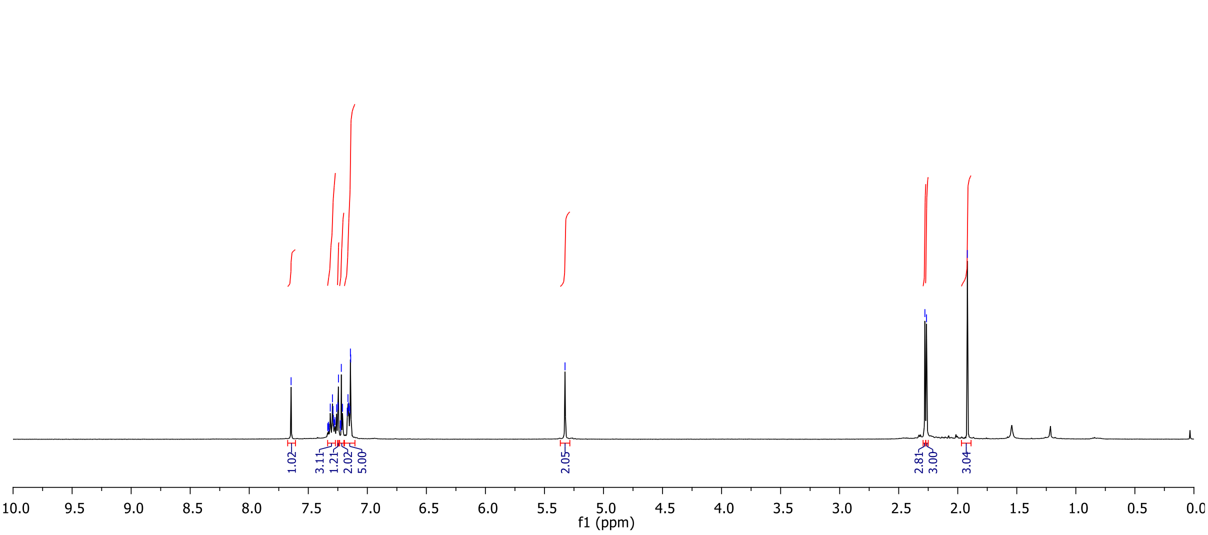

^{1}H NMR spectrum of 9ak (400 MHz, $CDCl_3$)

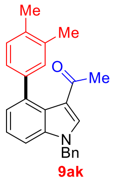

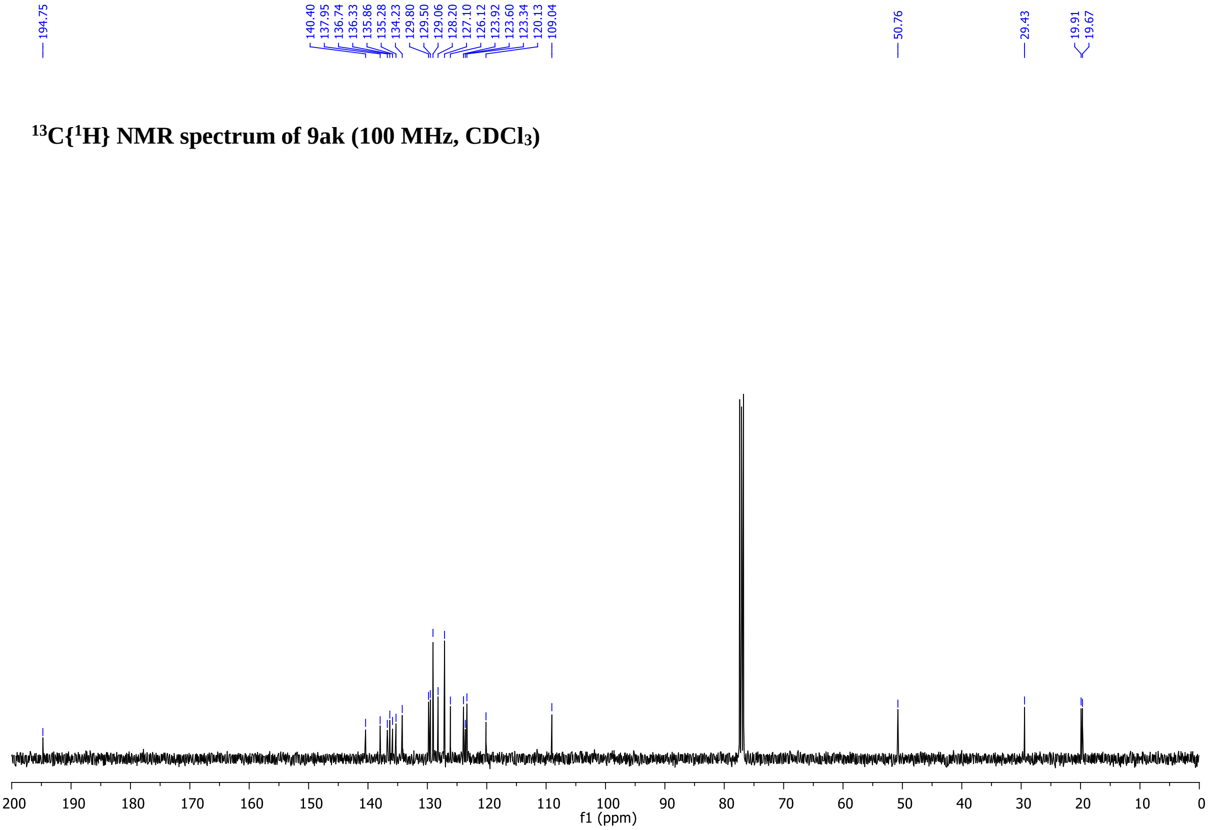

$^{13}C\{^1H\}$ NMR spectrum of 9ak (100 MHz, $CDCl_3$)

1-(1-Benzyl-4-(3-nitrophenyl)-1*H*-indol-3-yl)ethan-1-one (9al)

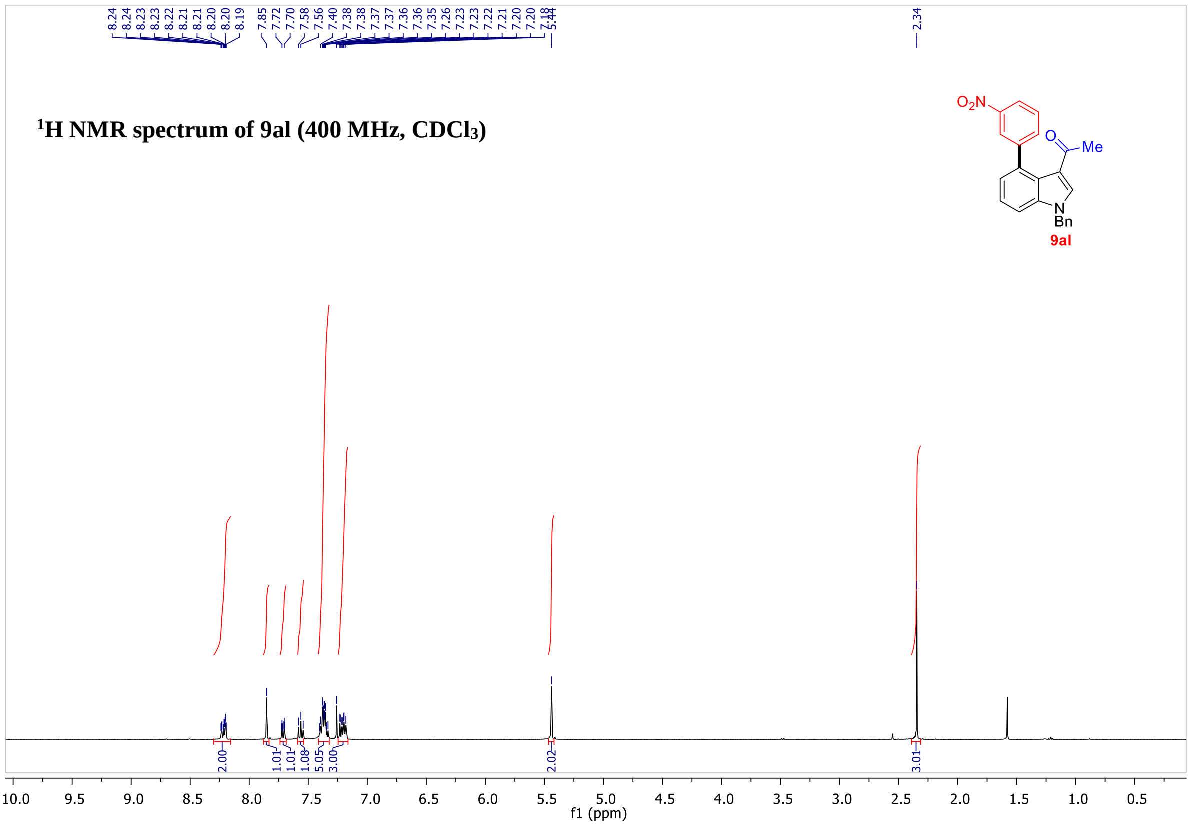

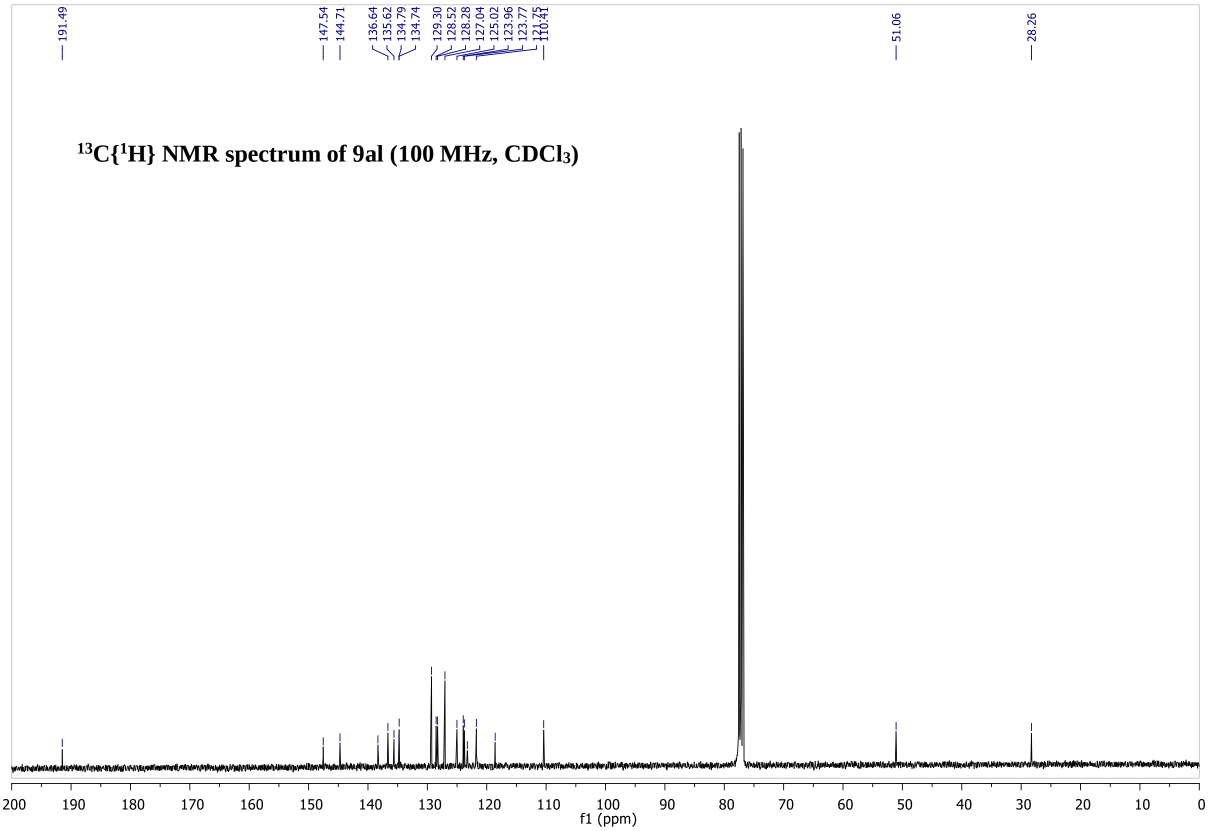

1-(1-Benzyl-7-fluoro-4-phenyl-1*H*-indol-3-yl)ethan-1-one (9ba)

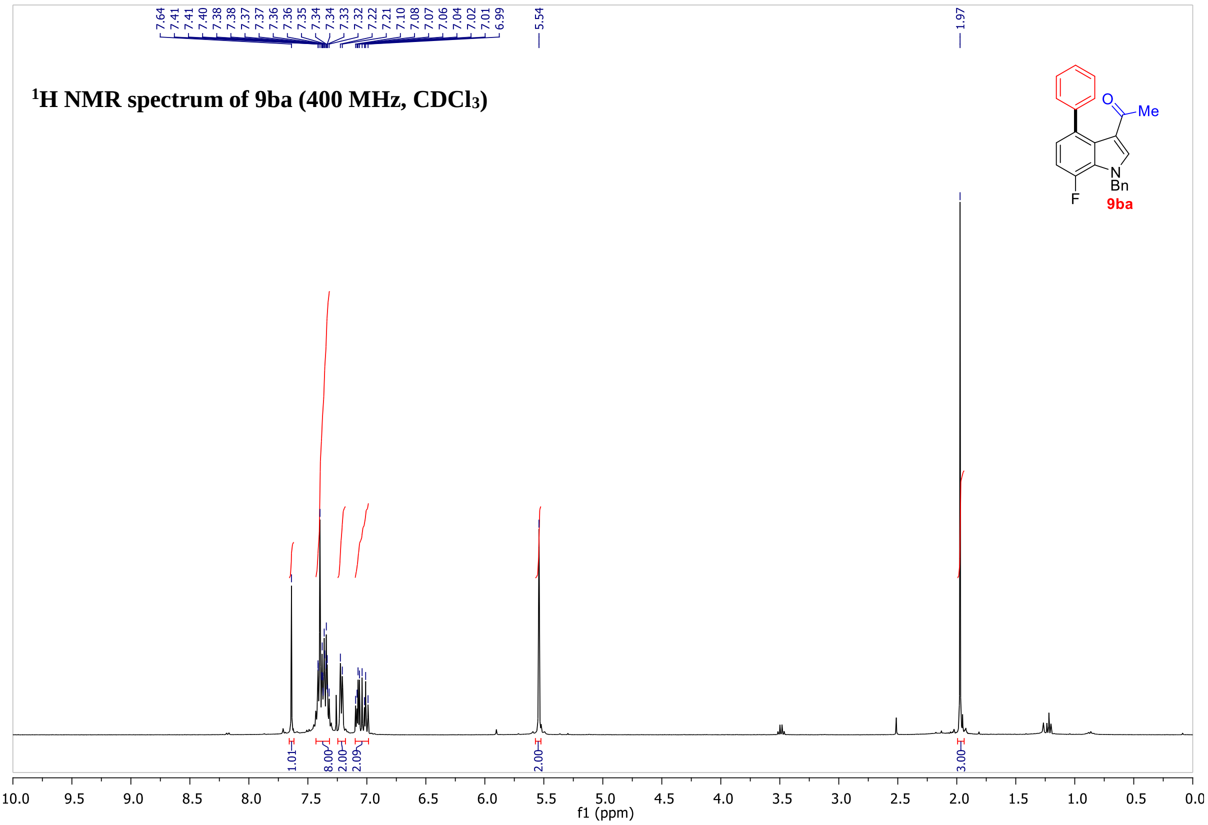

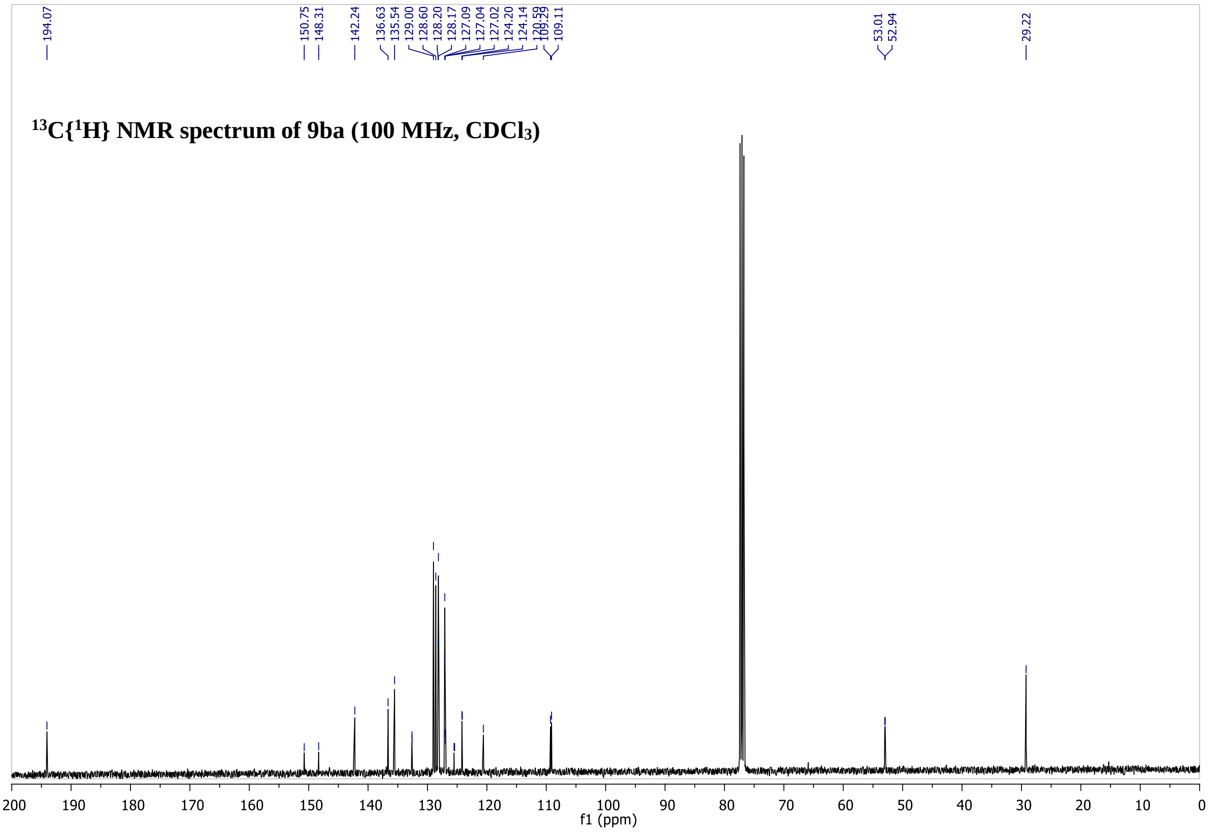

1-(1-Benzyl-7-bromo-4-phenyl-1*H*-indol-3-yl)ethan-1-one (9ca)

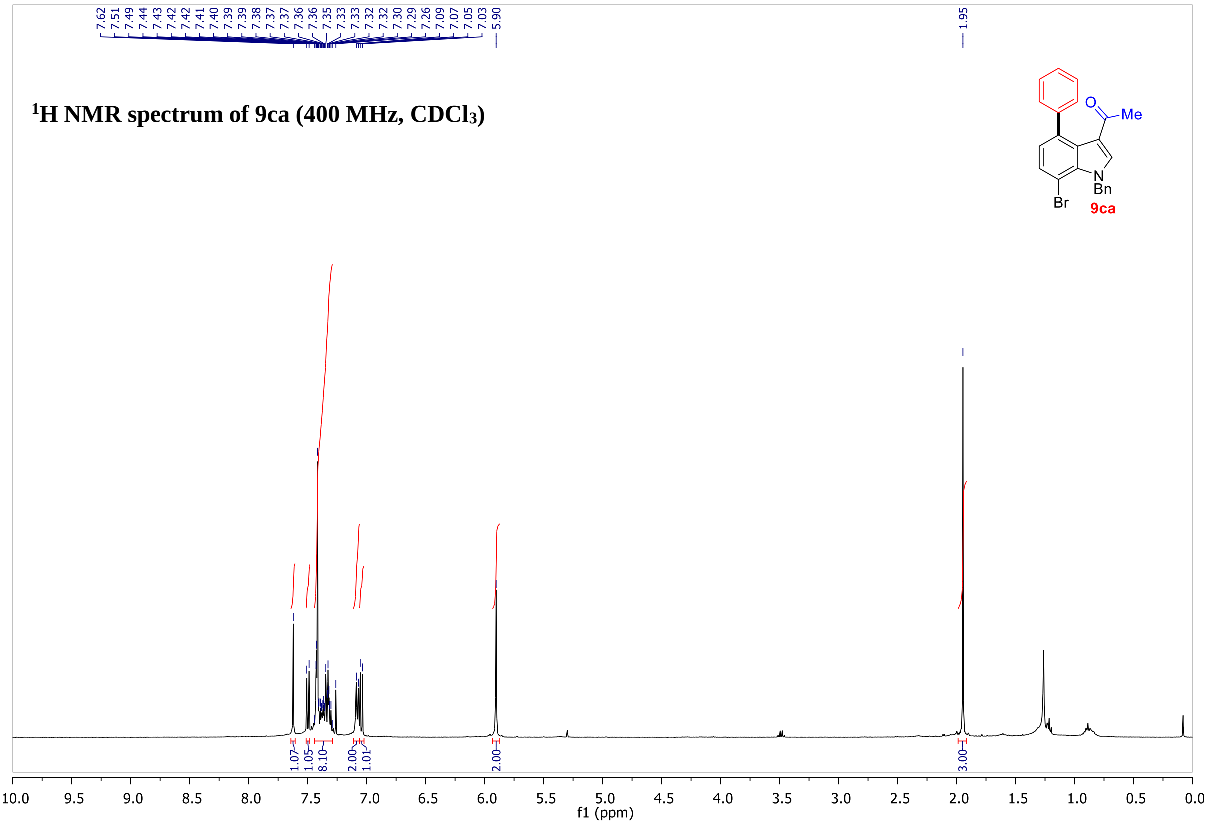

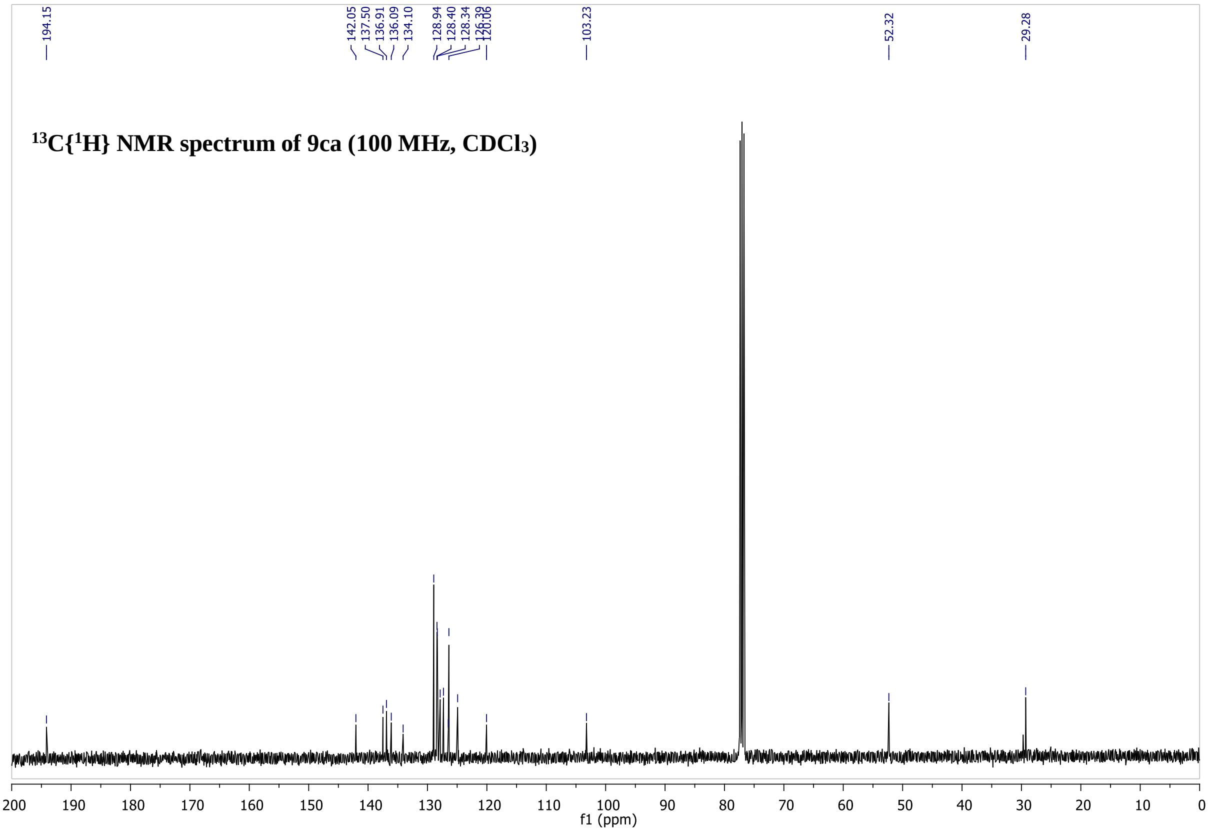

1-(1-Methyl-4-phenyl-1*H*-indol-3-yl)ethan-1-one (9ea)

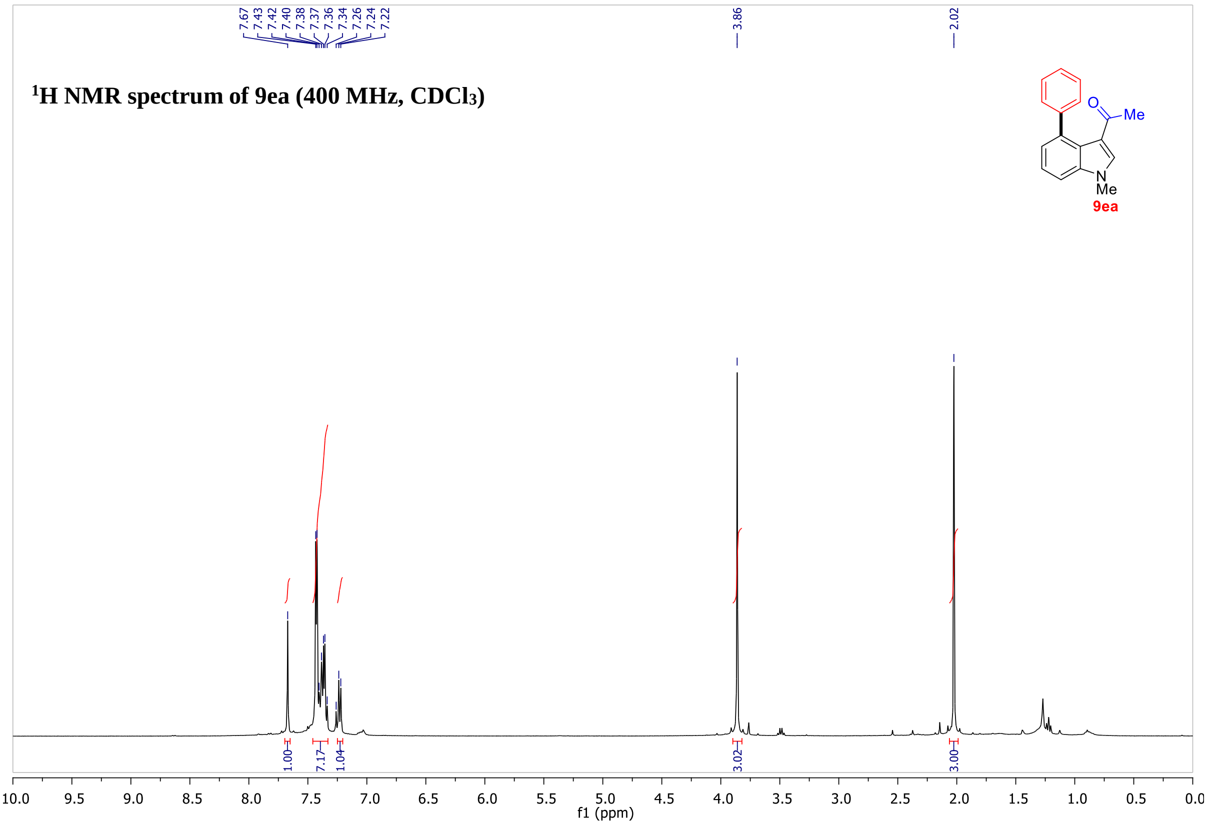

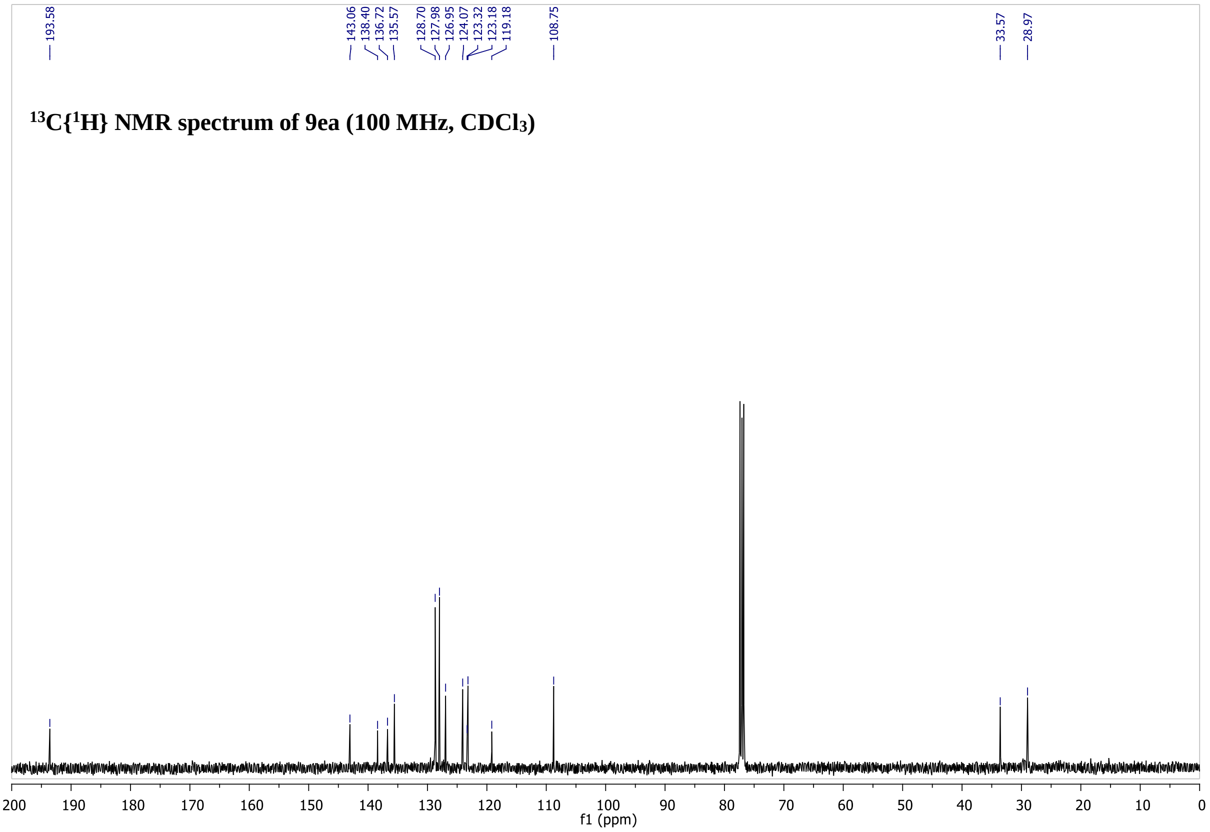

Methyl 4-(3-acetyl-1-methyl-1*H*-indol-4-yl)benzoate (9eg)

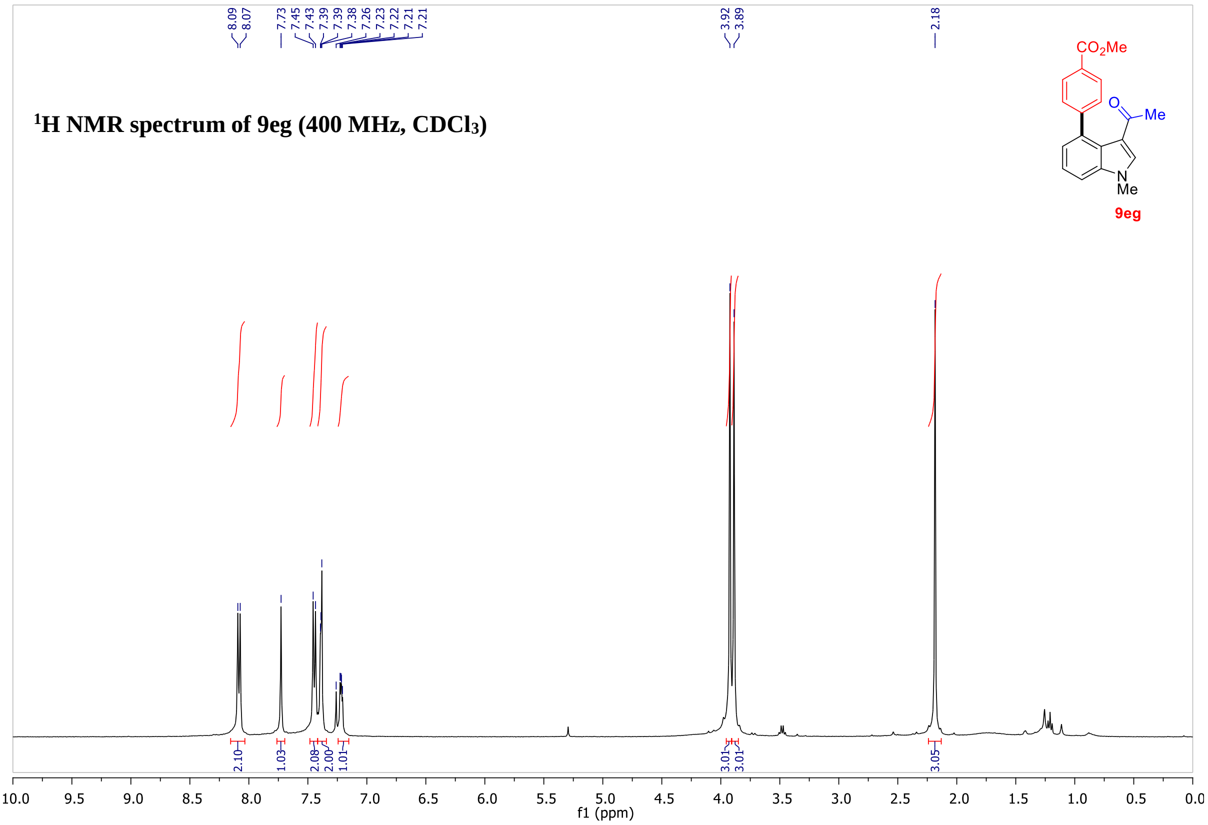

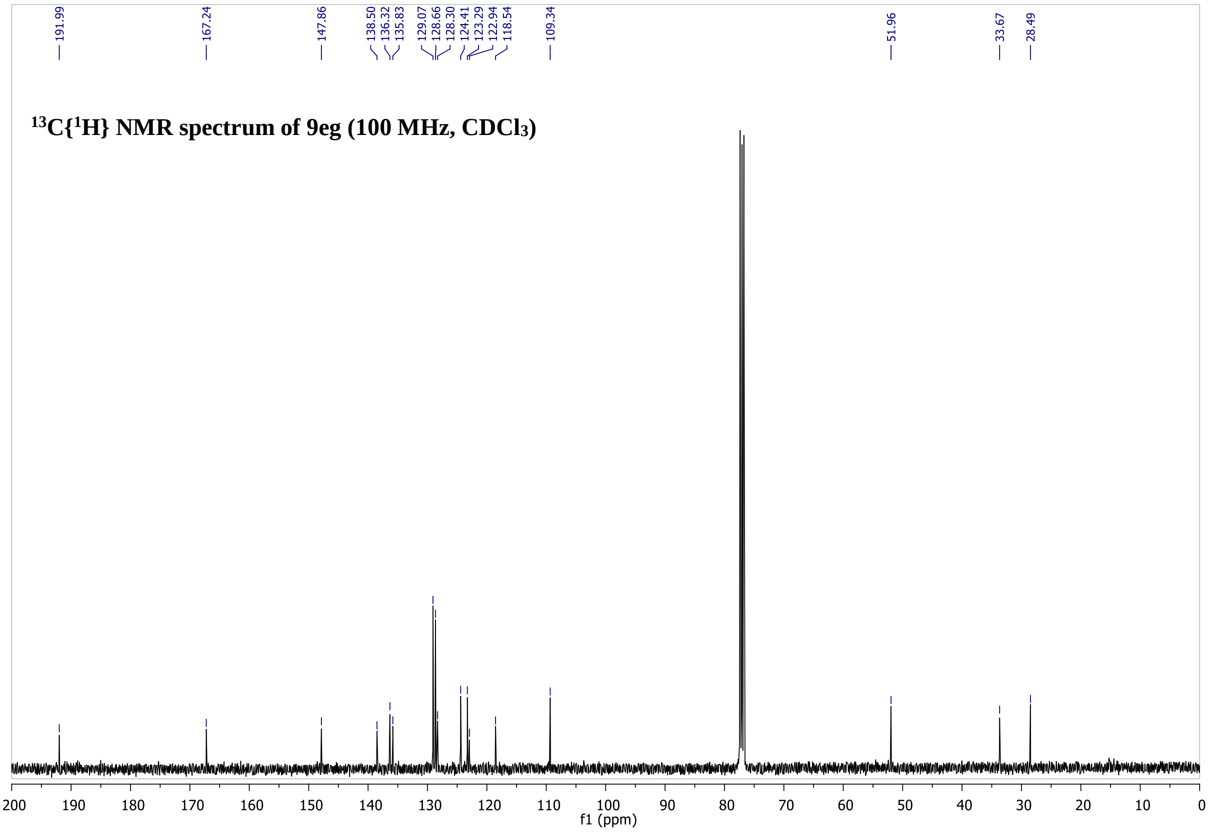

2-Methyl-1*H*-indole-3-carboxylic acid (10c)

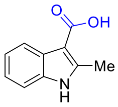

10c

^{1}H NMR spectrum of 10c (400 MHz, DMSO-d_6)

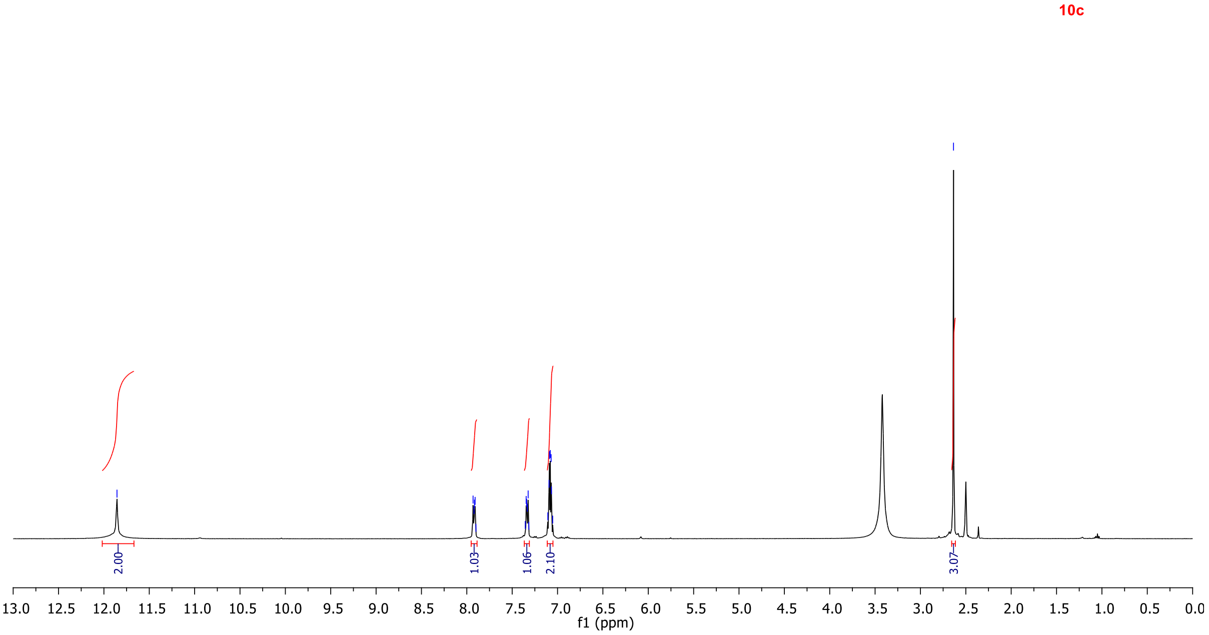

^{13}C{^{1}H} NMR spectrum of 10c (100 MHz, DMSO-d_6)

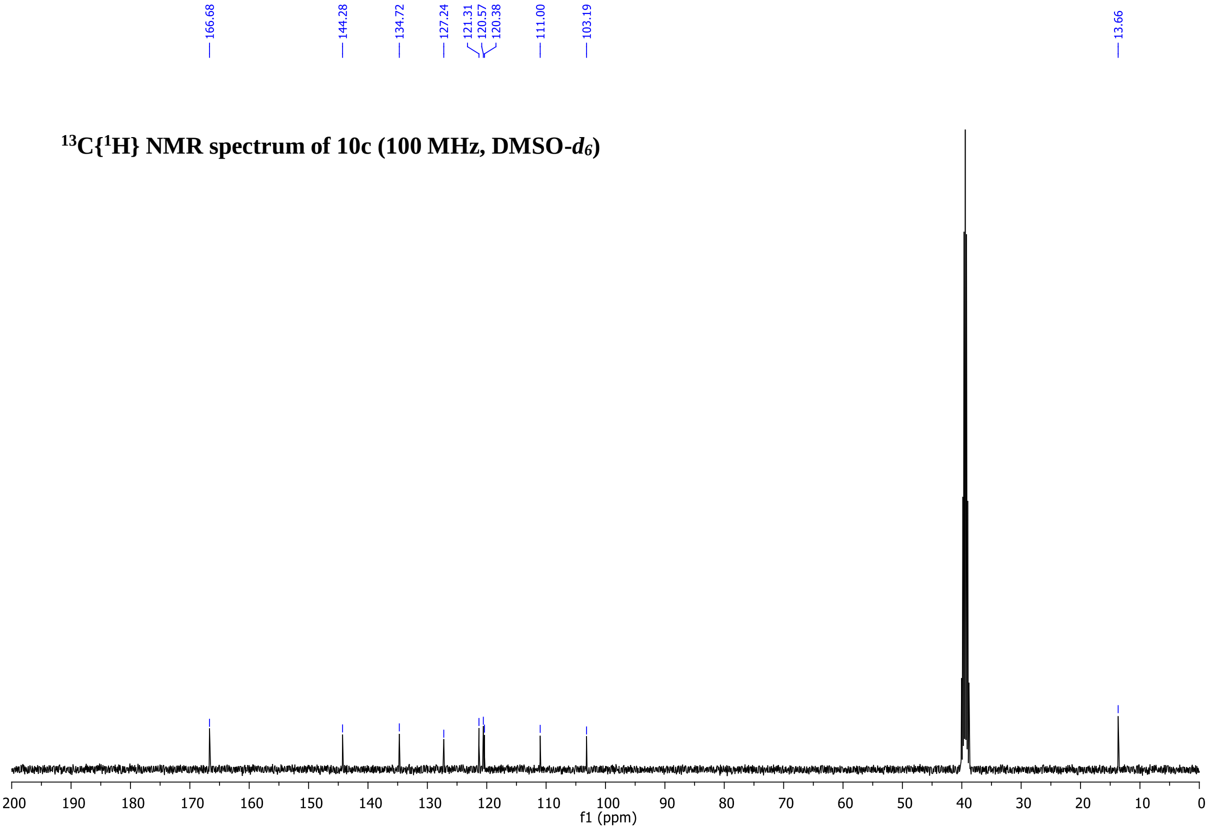

2-Phenyl-1*H*-indole (11a)

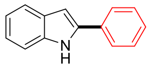

11a

^{1}H NMR spectrum of 11a (400 MHz, $CDCl_3$)

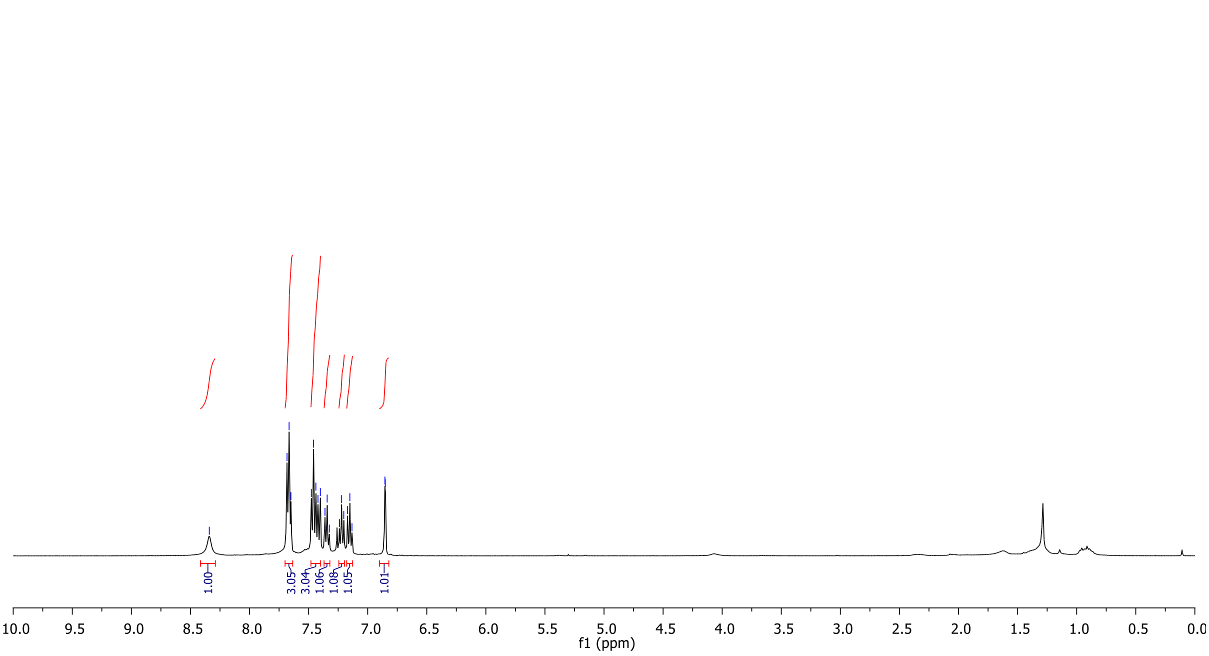

^{13}C{^{1}H} NMR spectrum of 11a (100 MHz, $CDCl_3$)

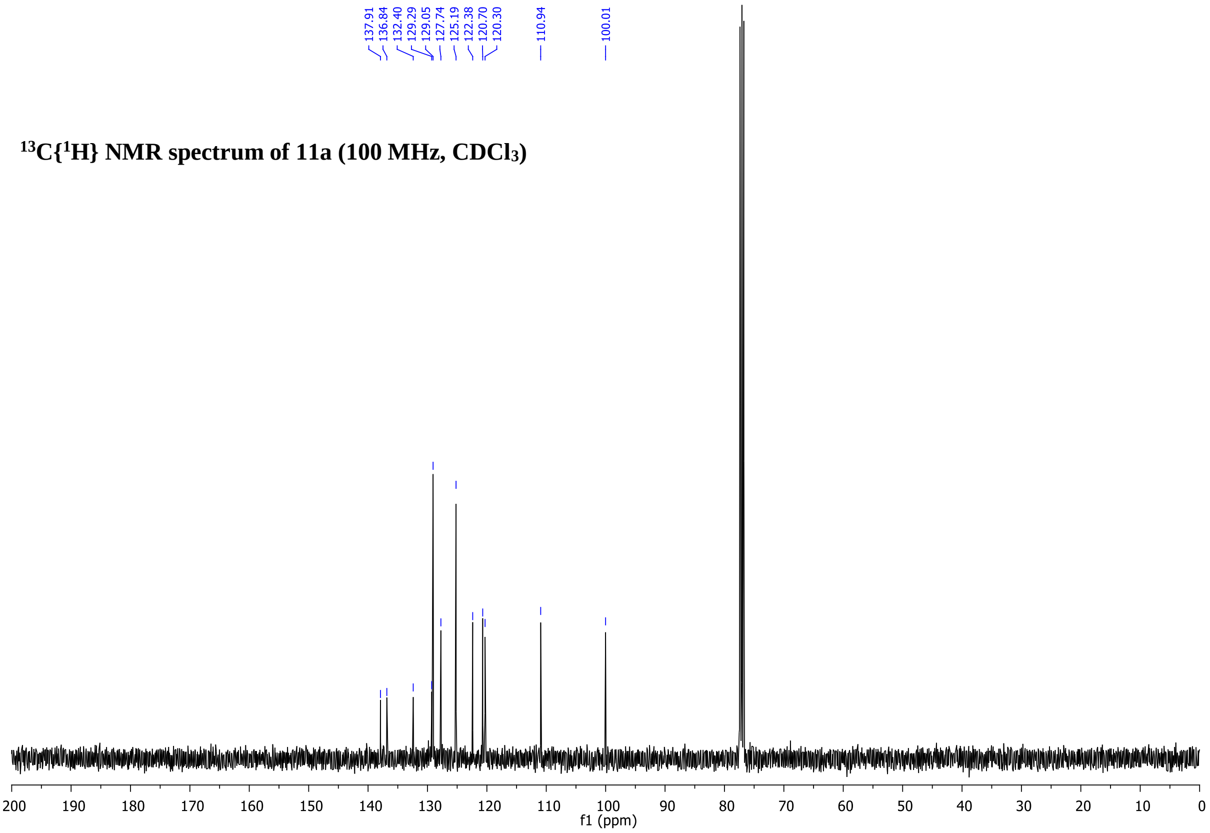

2-(*p*-Tolyl)-1*H*-indole (11b)

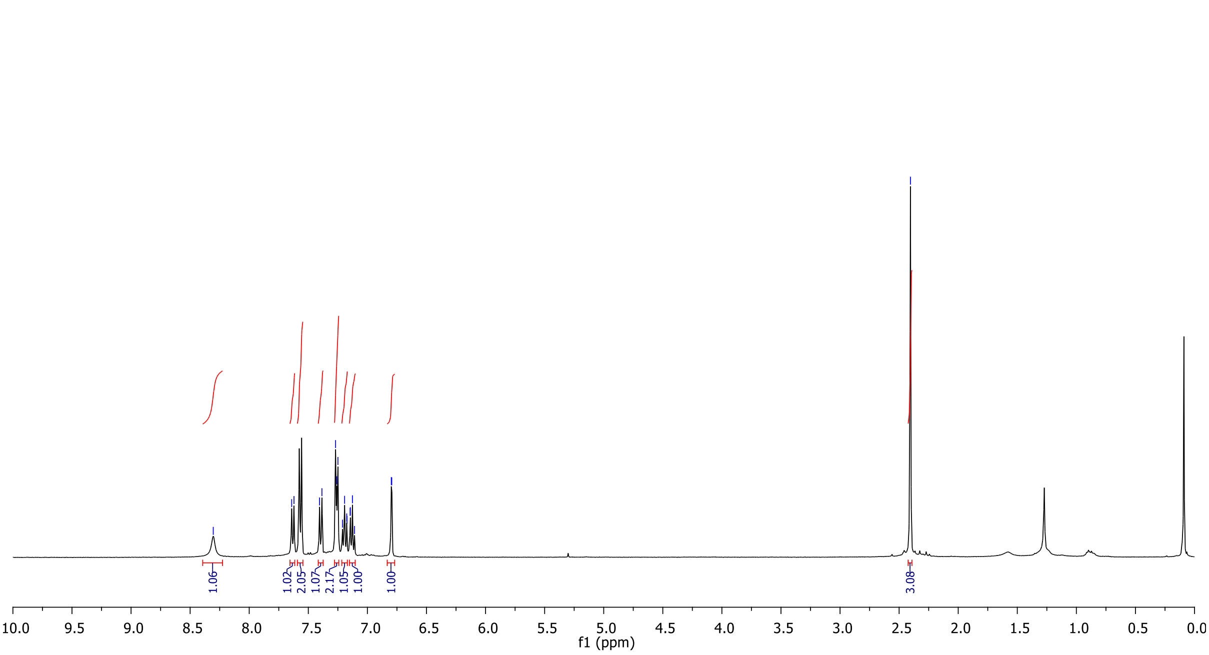

^{1}H NMR spectrum of 11b (400 MHz, $CDCl_3$)

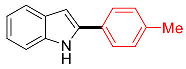

11b

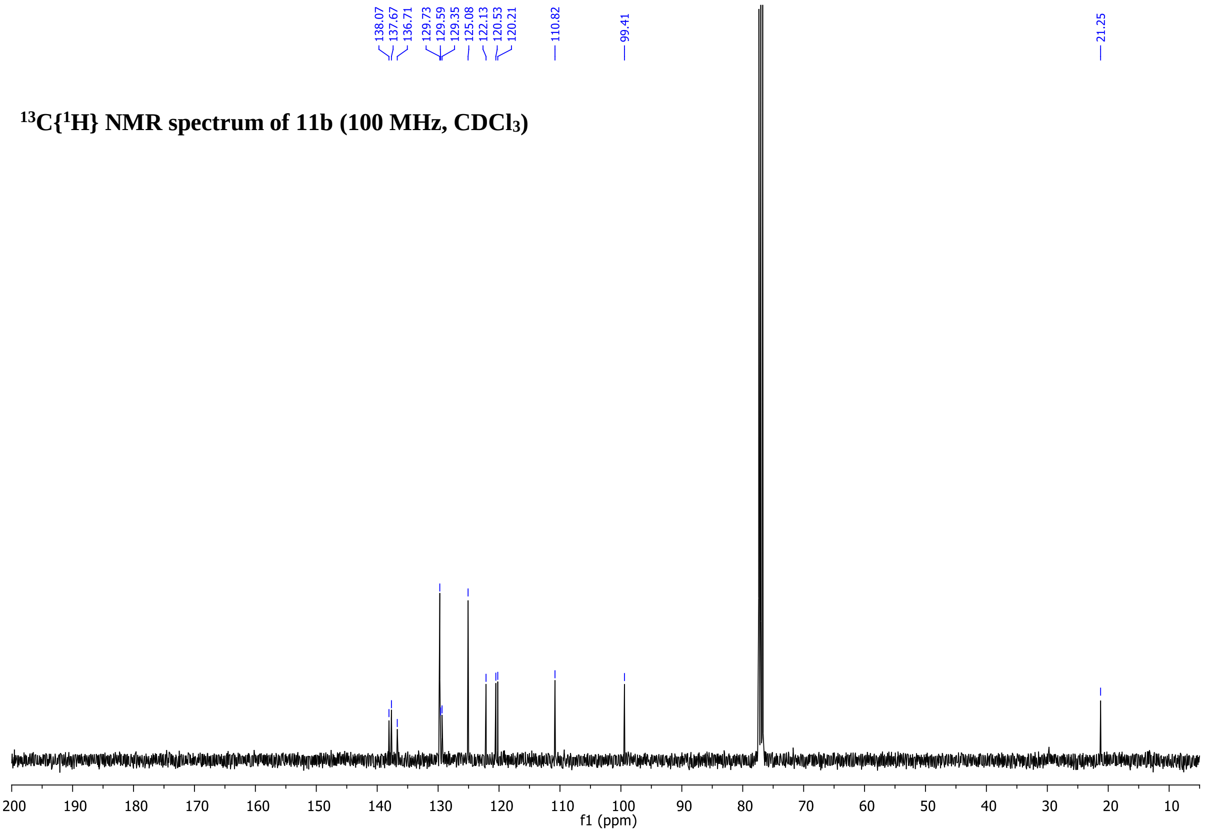

^{13}C{^{1}H} NMR spectrum of 11b (100 MHz, $CDCl_3$)

2-(*m*-Tolyl)-1*H*-indole (11c)

^{1}H NMR spectrum of 11c (400 MHz, $CDCl_3$)

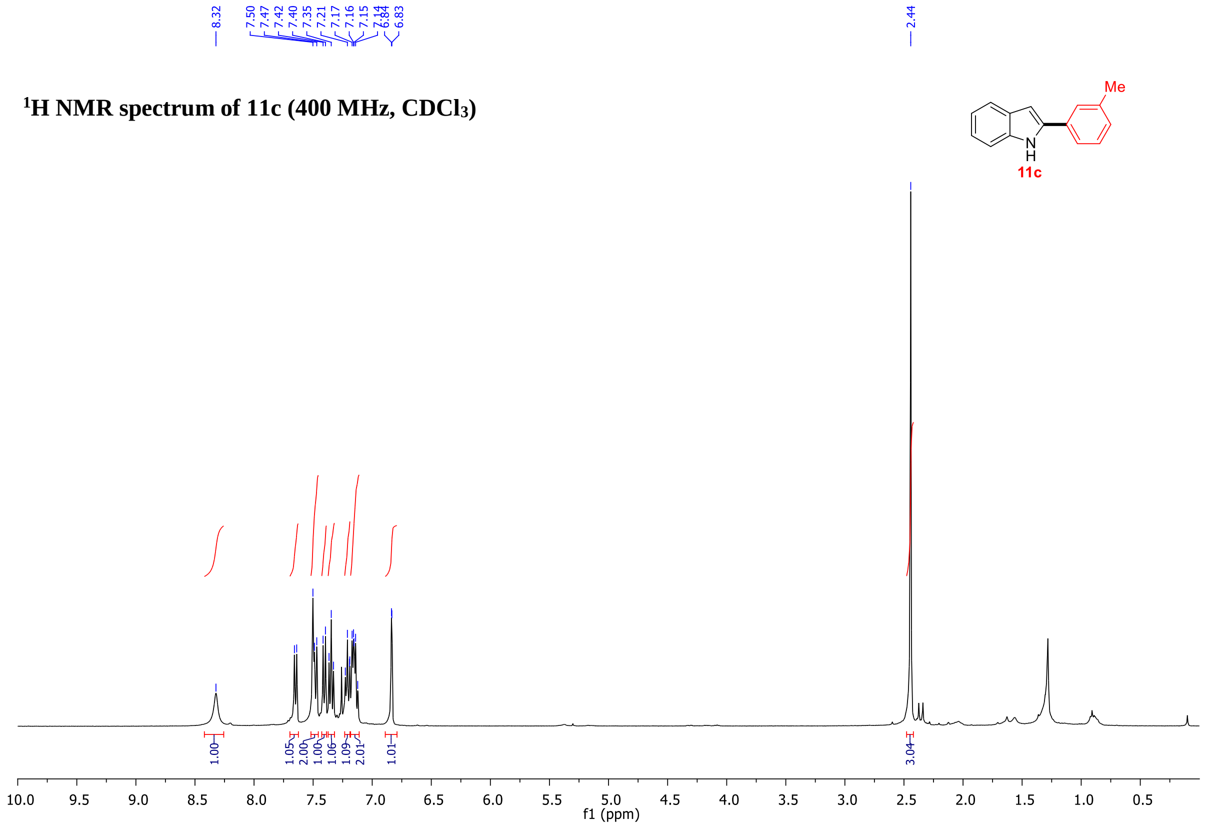

^{13}C{^{1}H} NMR spectrum of 11c (100 MHz, $CDCl_3$)

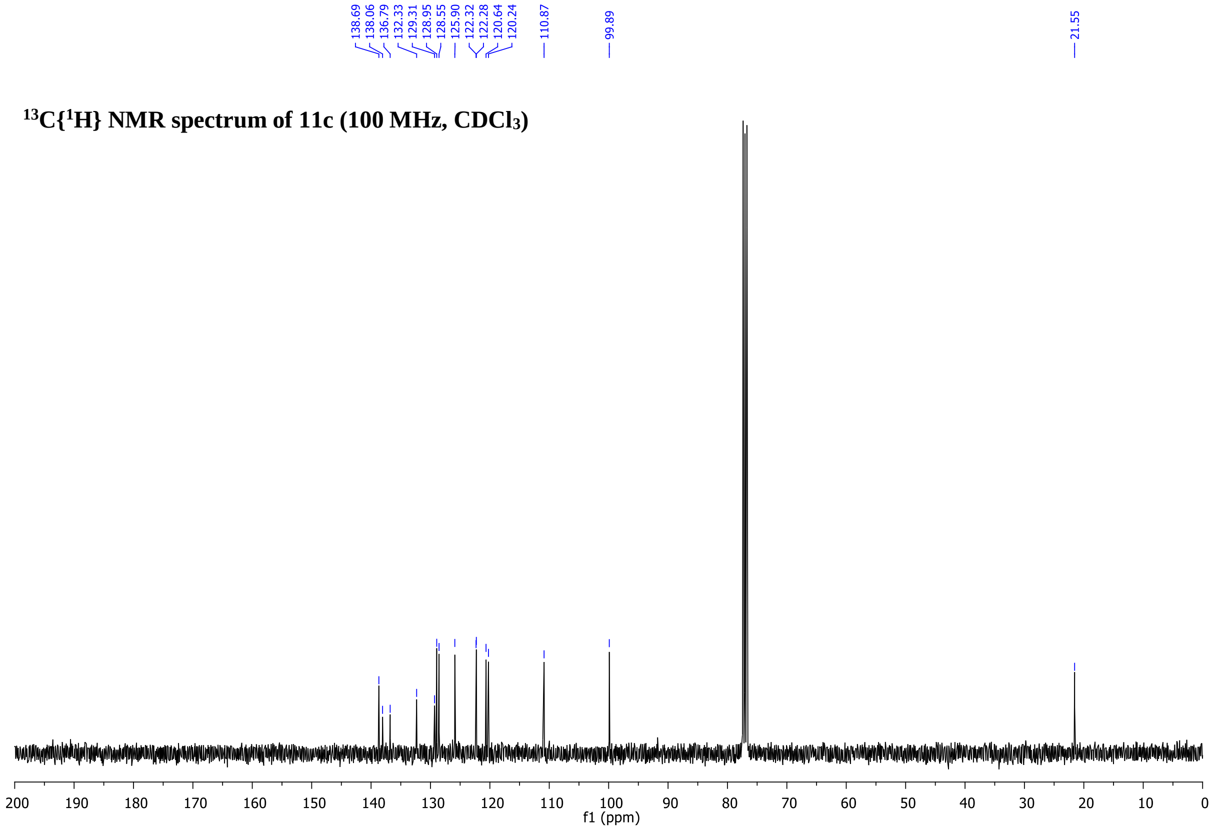

2-(4-(*tert*-Butyl)phenyl)-1*H*-indole (11d)

^{1}H NMR spectrum of 11d (400 MHz, $CDCl_3$)

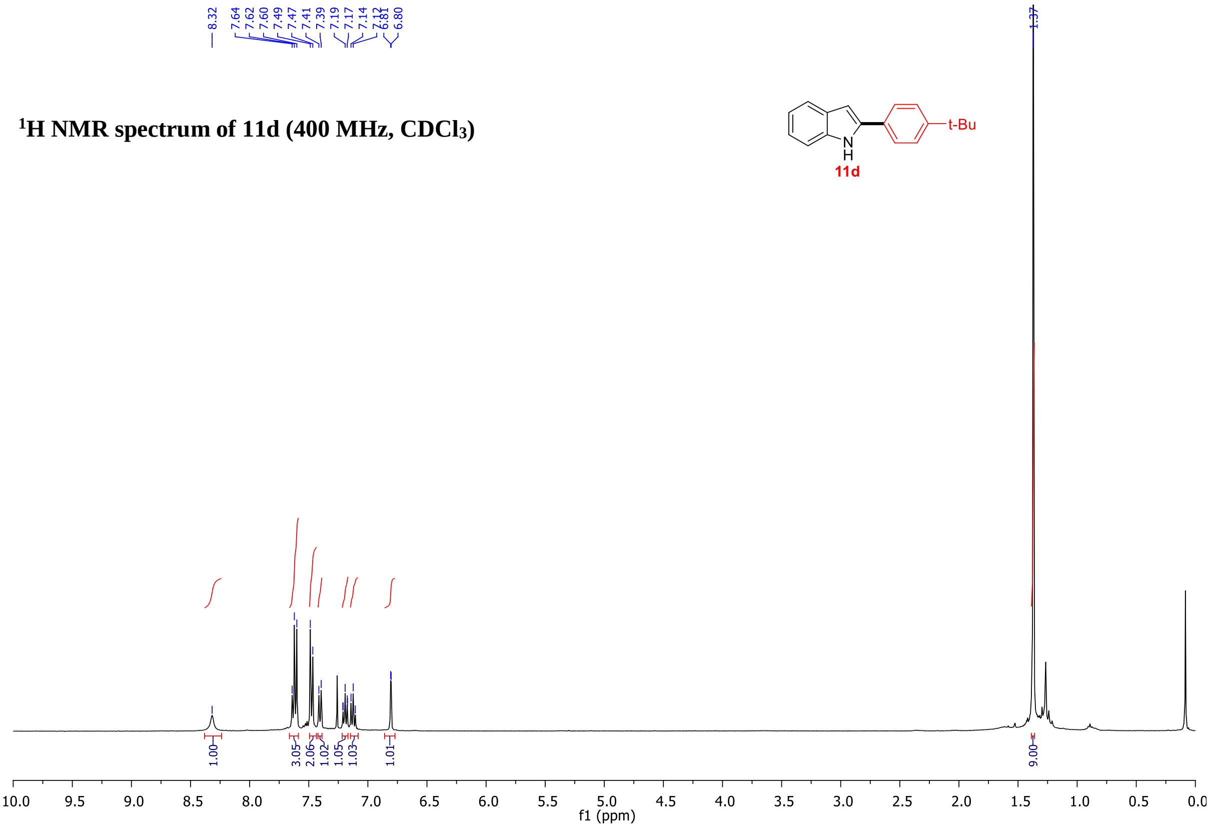

^{13}C{^{1}H} NMR spectrum of 11d (100 MHz, $CDCl_3$)

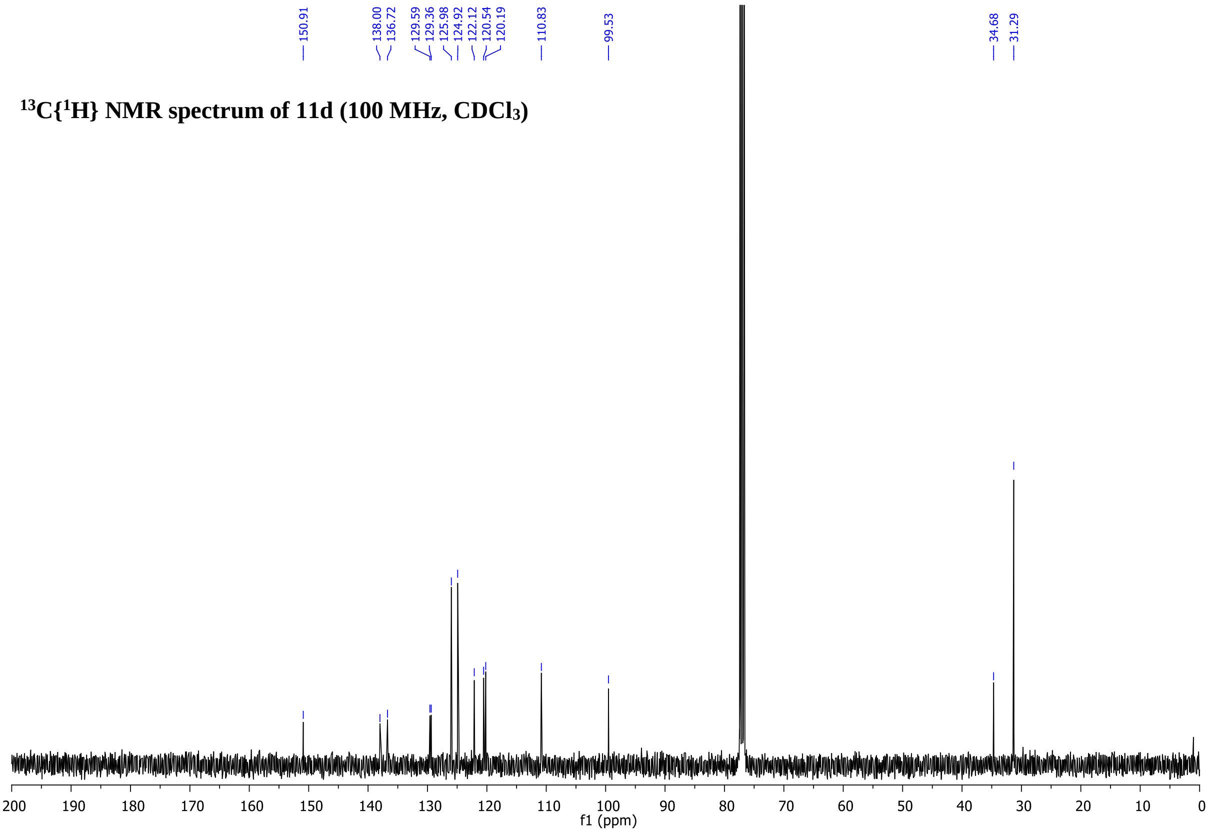

2-(4-Bromophenyl)-1*H*-indole (11e)

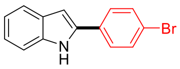

^{1}H NMR spectrum of 11e (400 MHz, $CDCl_3$)

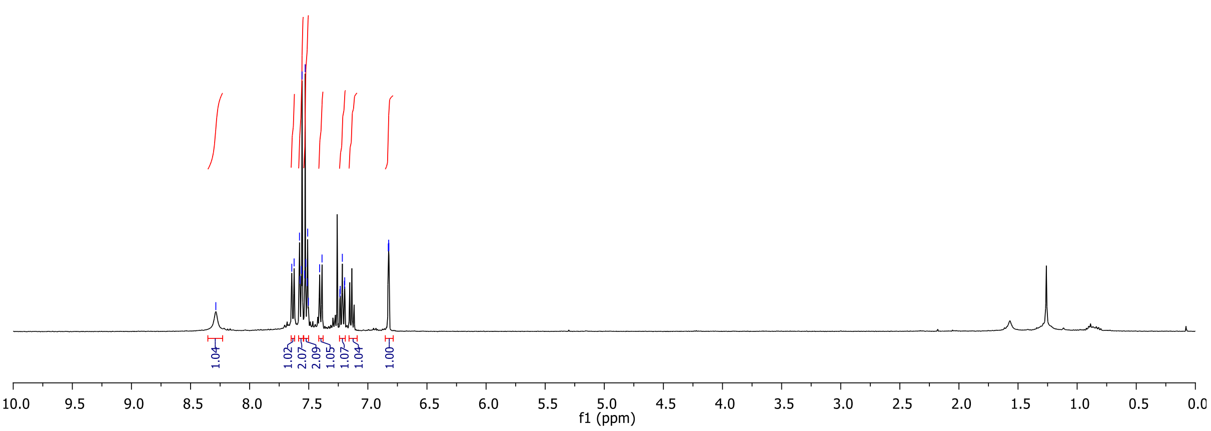

$^{13}C\{^{1}H\}$ NMR spectrum of 11e (100 MHz, $CDCl_3$)

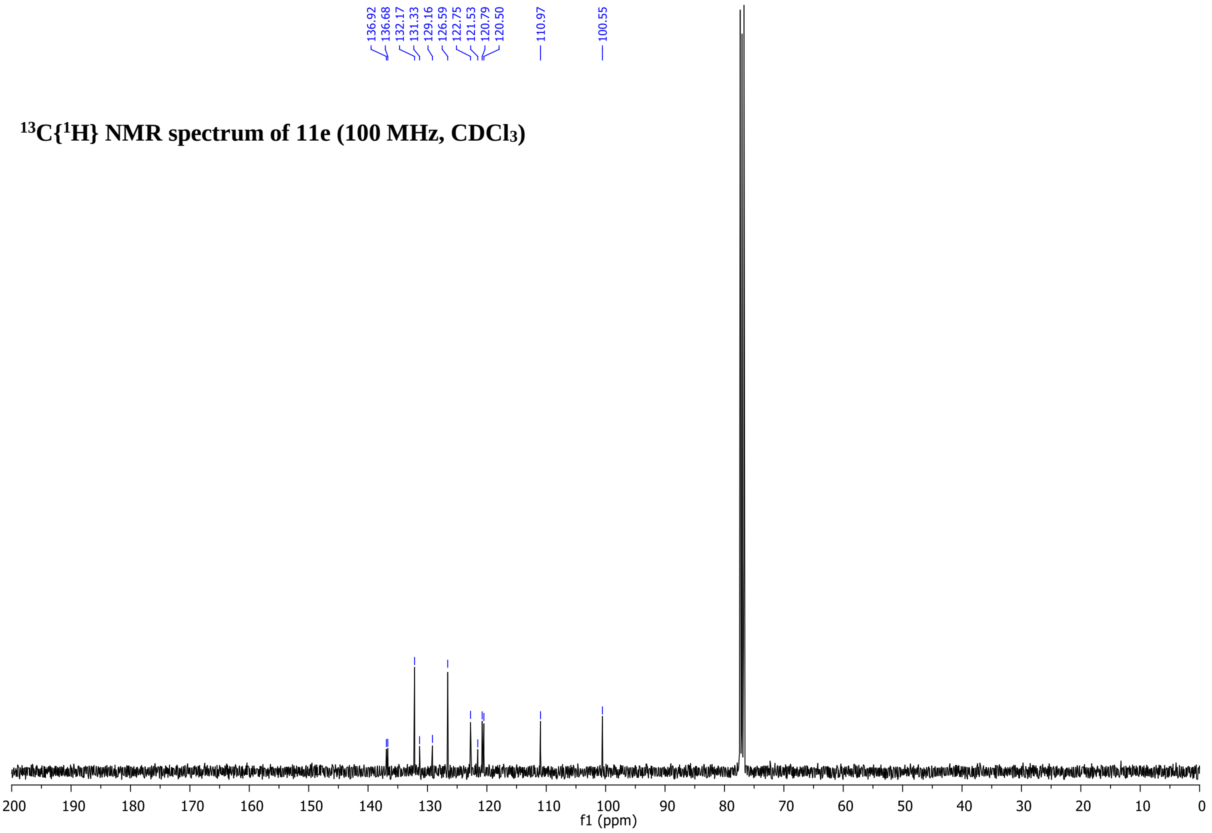

2-(3,4-Dimethylphenyl)-1*H*-indole (11k)

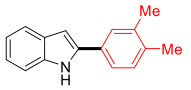

^{1}H NMR spectrum of 11k (400 MHz, $CDCl_3$)

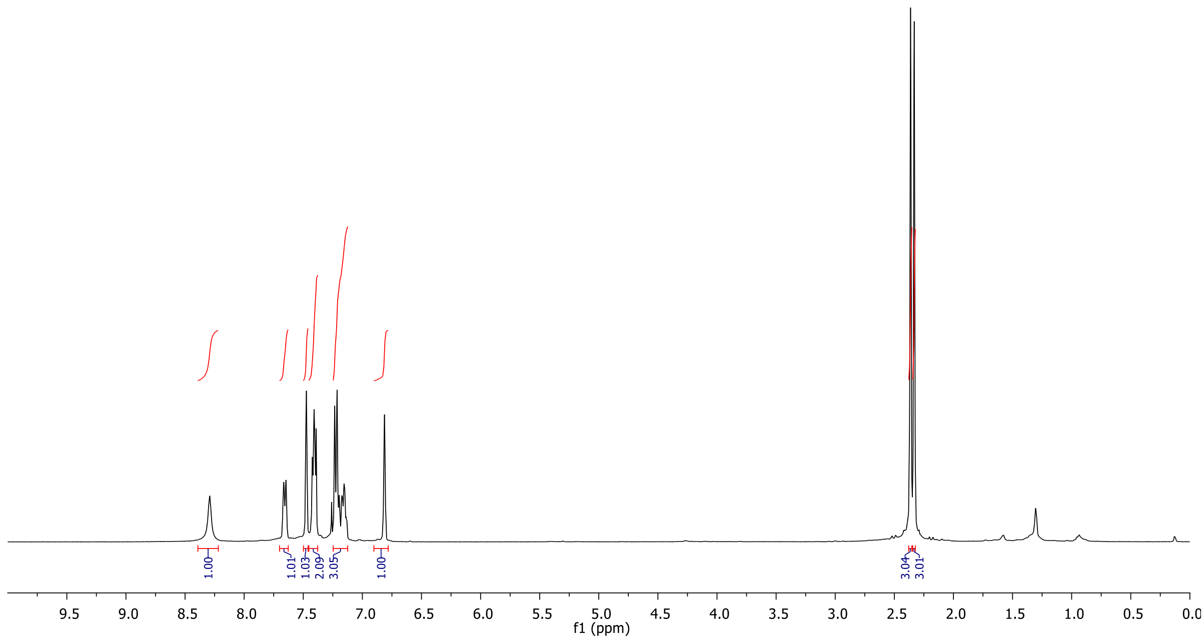

^{13}C{^{1}H} NMR spectrum of 11k (100 MHz, $CDCl_3$)

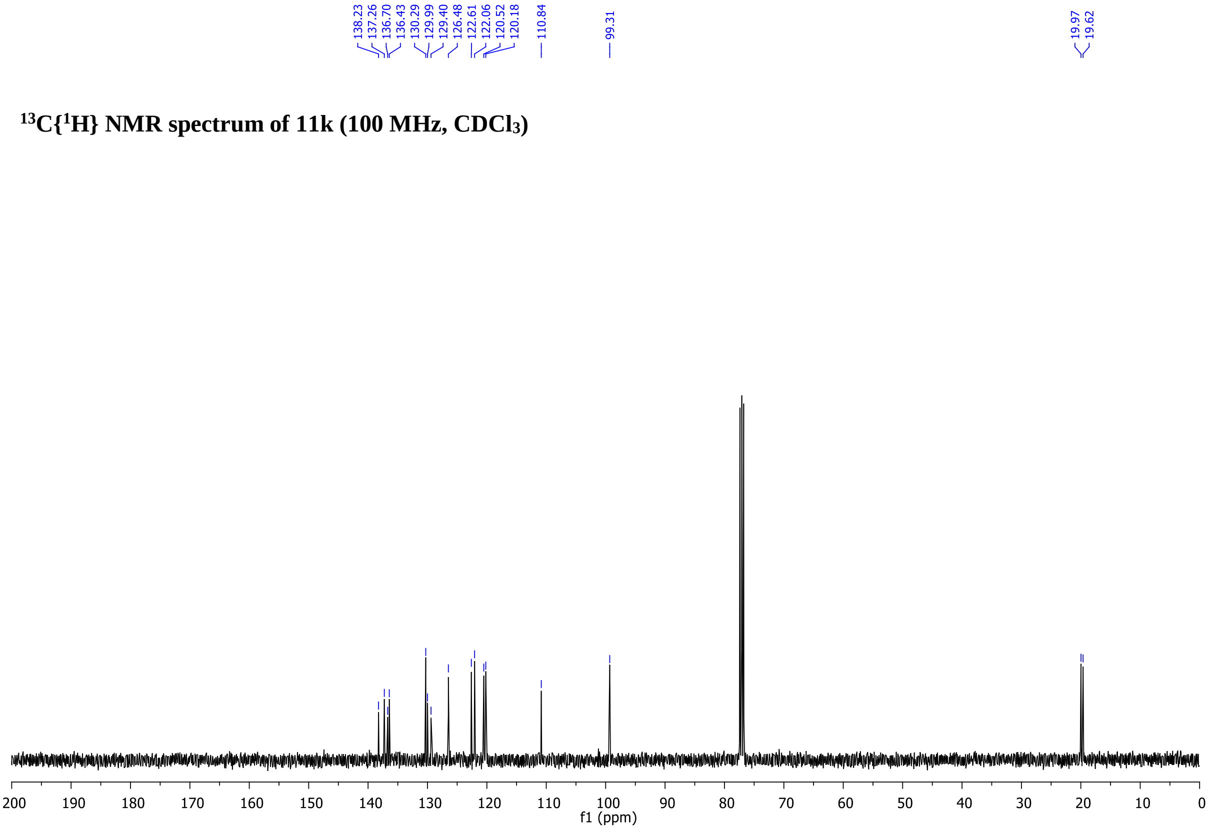

1-(1-Benzyl-4-phenyl-1*H*-indol-2-yl)ethan-1-one (12)

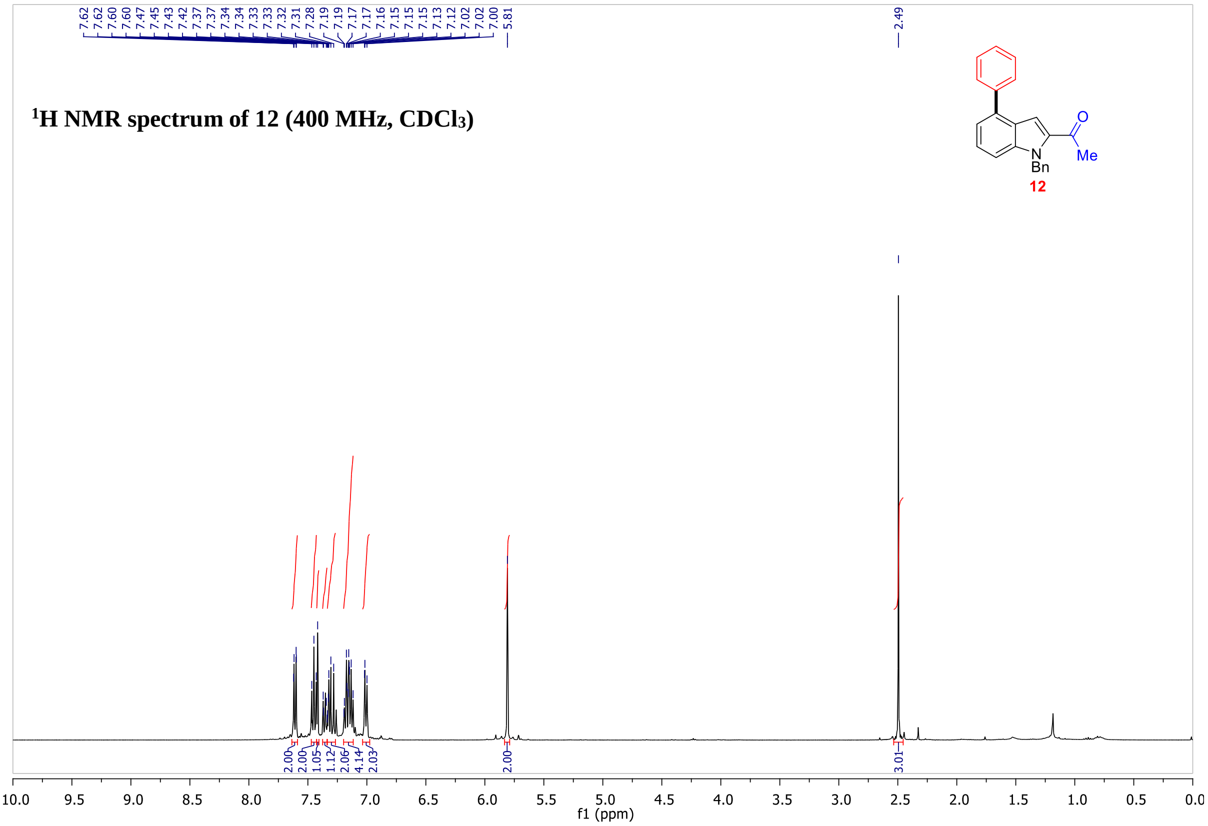

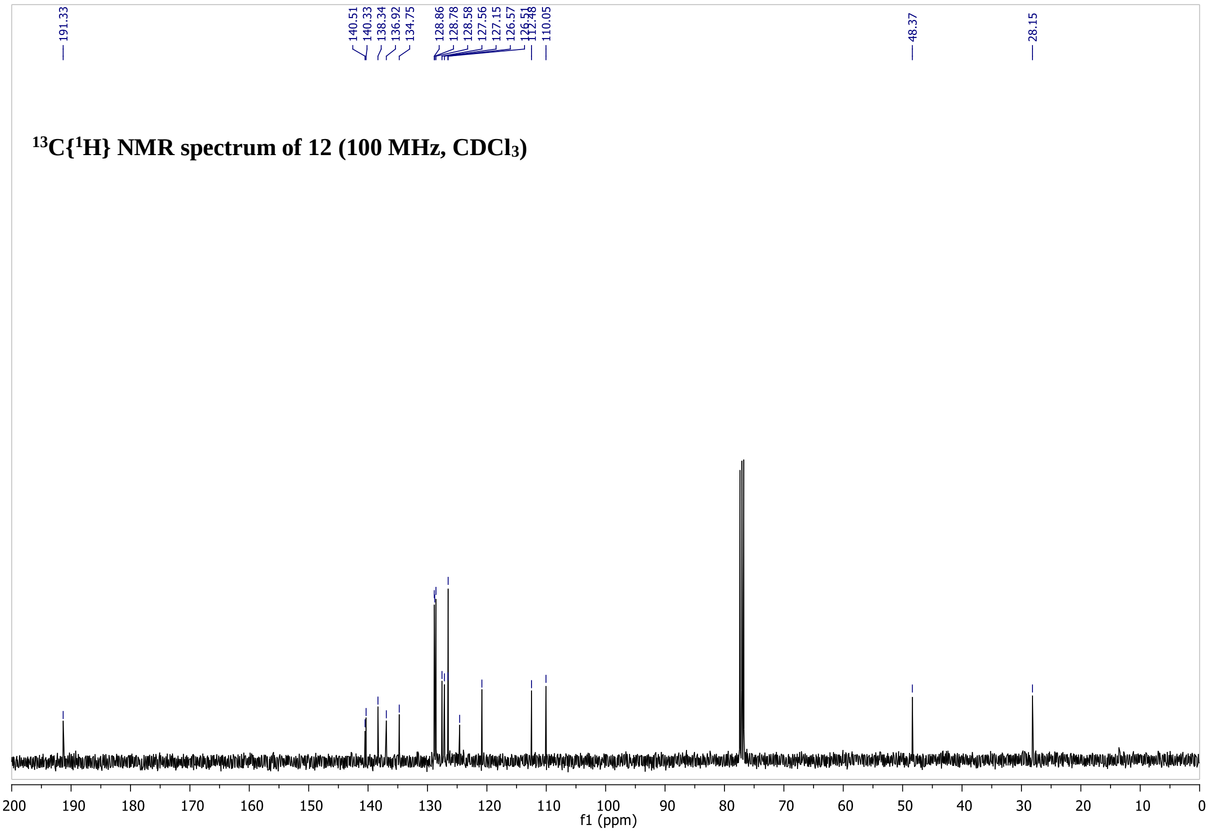

1-(4-Phenyl-7-(thiophen-2-yl)-1*H*-indol-2-yl)ethan-1-one (13)

^{1}H NMR spectrum of 13 (400 MHz, $CDCl_3$)

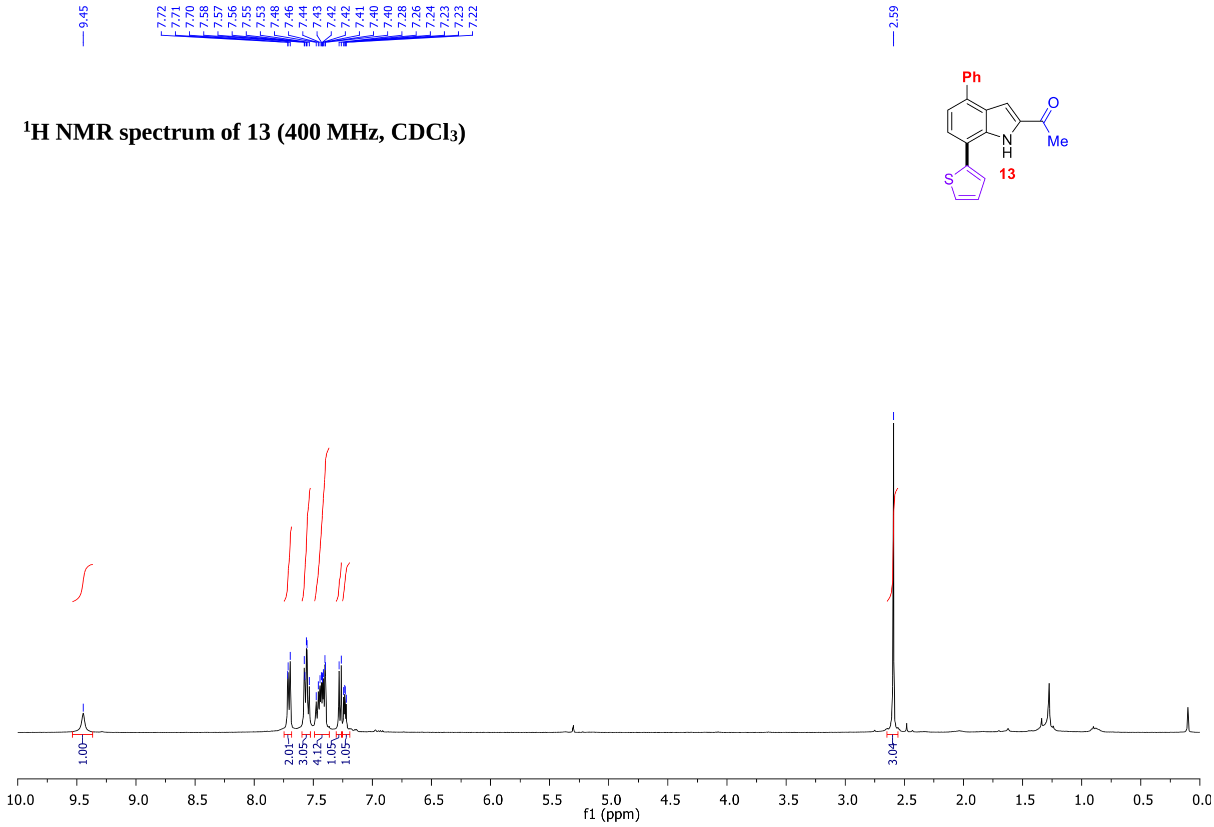

^{13}C{^{1}H} NMR spectrum of 13 (100 MHz, $CDCl_3$)

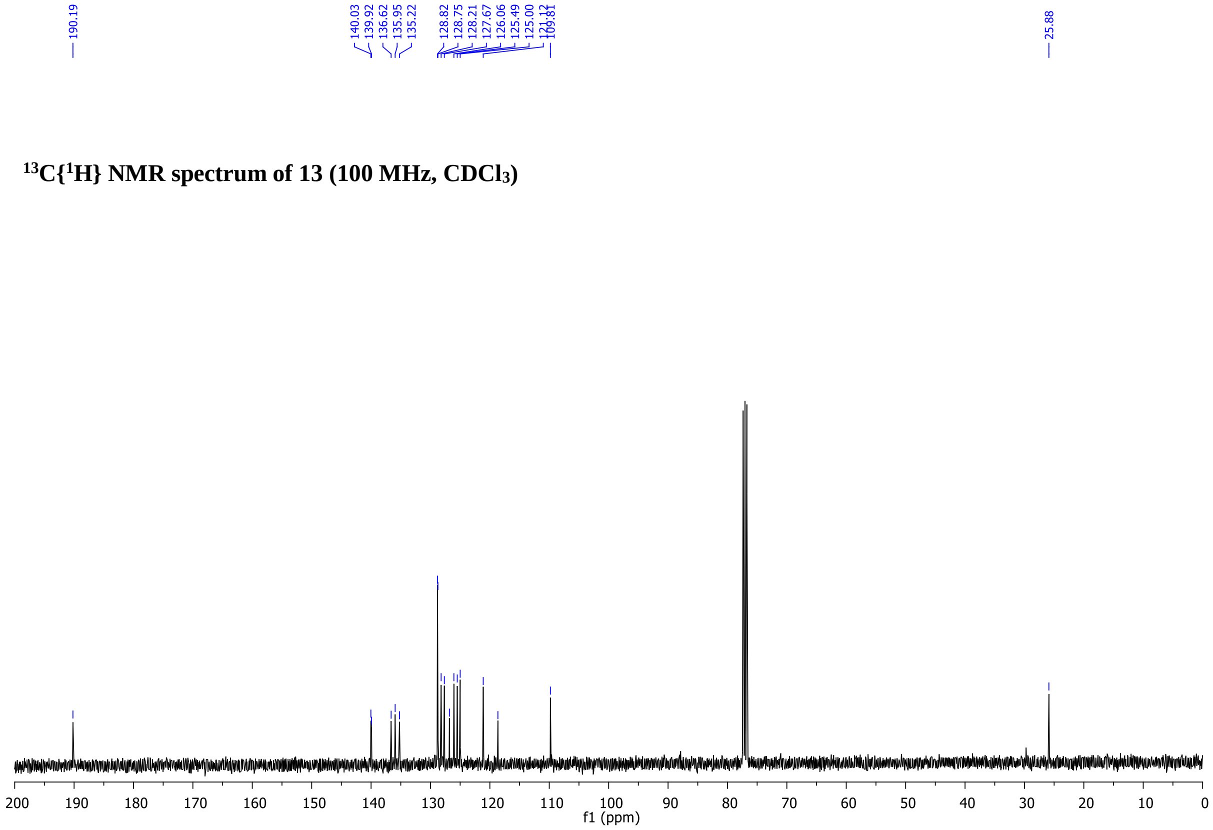

(1-Benzyl-4-(4-(*tert*-butyl)phenyl)-1*H*-indol-3-yl)(6-methoxy-9*H*-pyrido[3,4-b]indol-1-yl)methanone (16)

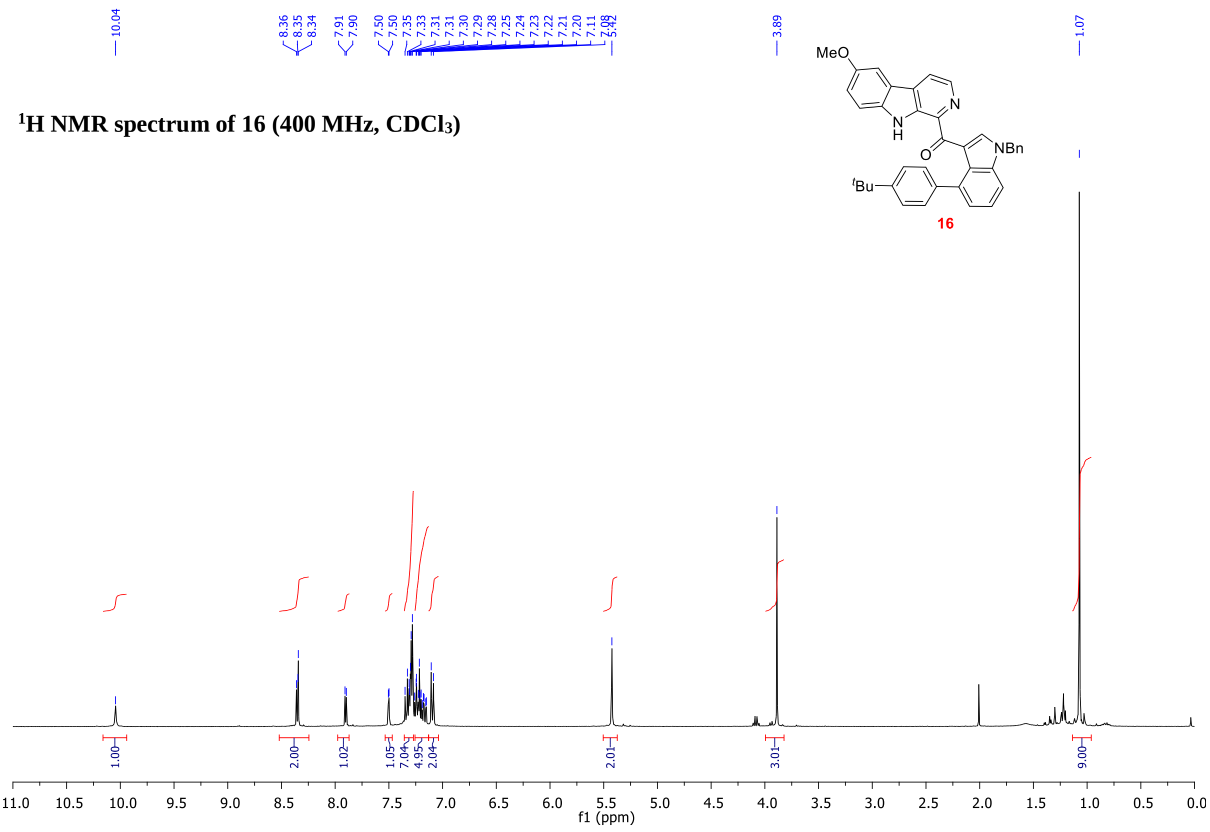

^{1}H NMR spectrum of 16 (400 MHz, $CDCl_3$)

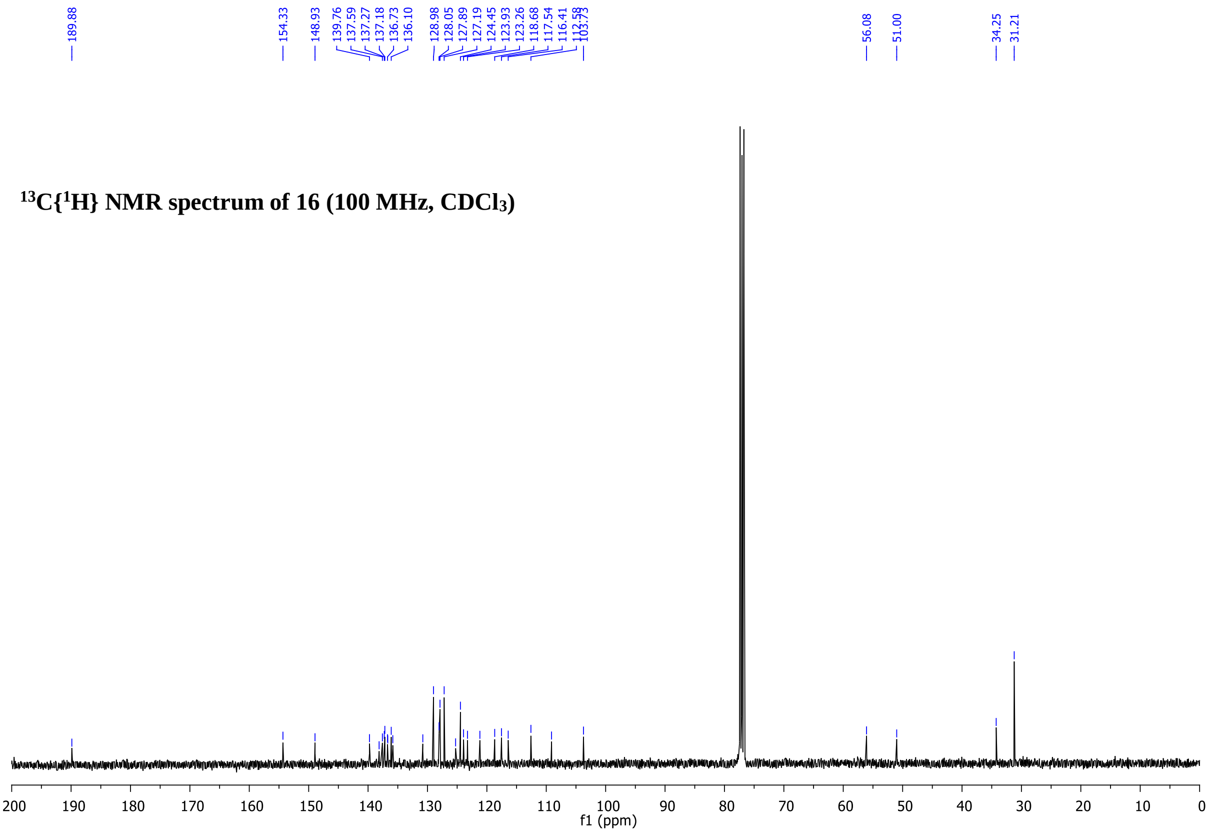

^{13}C{^{1}H} NMR spectrum of 16 (100 MHz, $CDCl_3$)

(6-Methoxy-9H-pyrido[3,4-b]indol-1-yl)(4-phenyl-1H-indol-2-yl)methanone (17)

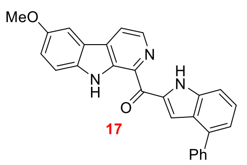

17

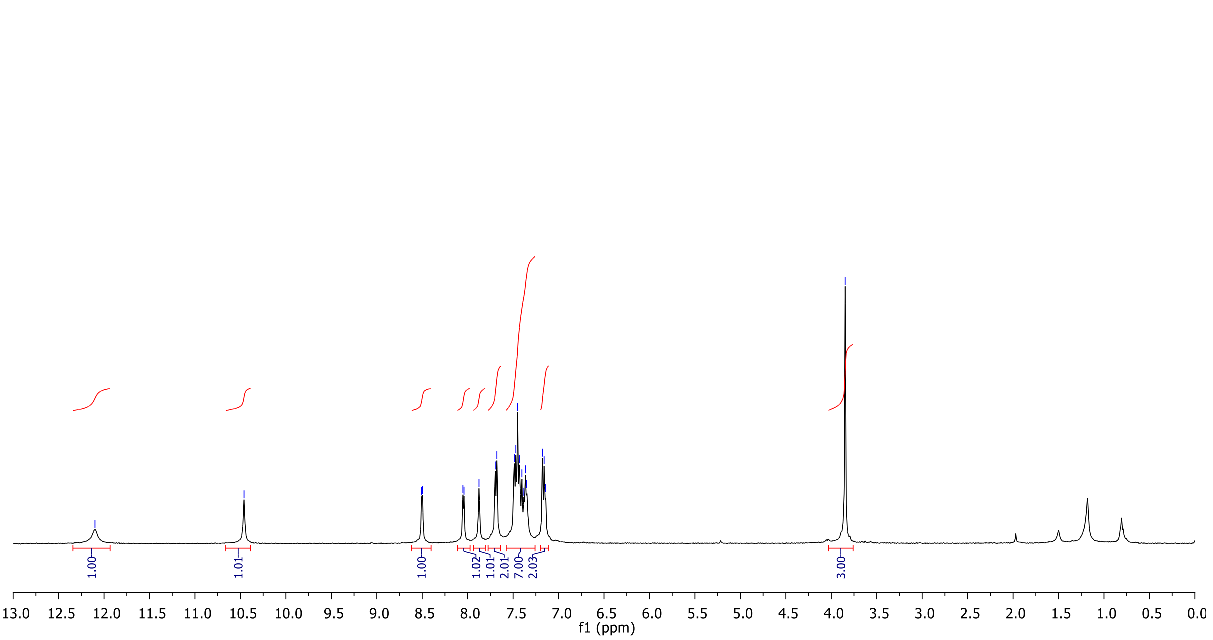

^{1}H NMR spectrum of 17 (400 MHz, $CDCl_3$)

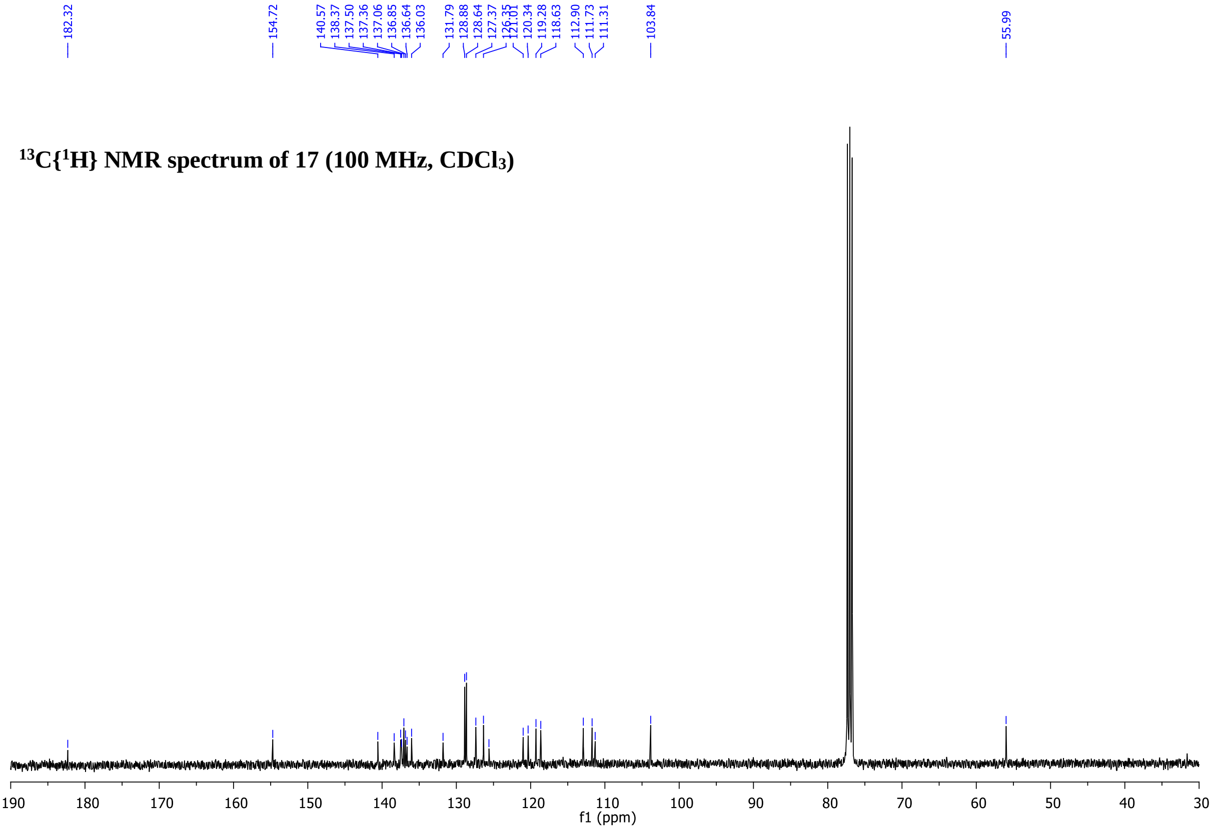

$^{13}C\{^{1}H\}$ NMR spectrum of 17 (100 MHz, $CDCl_3$)

2,2,2-Trifluoro-1-(1*H*-indol-3-yl)ethan-1-one (18)

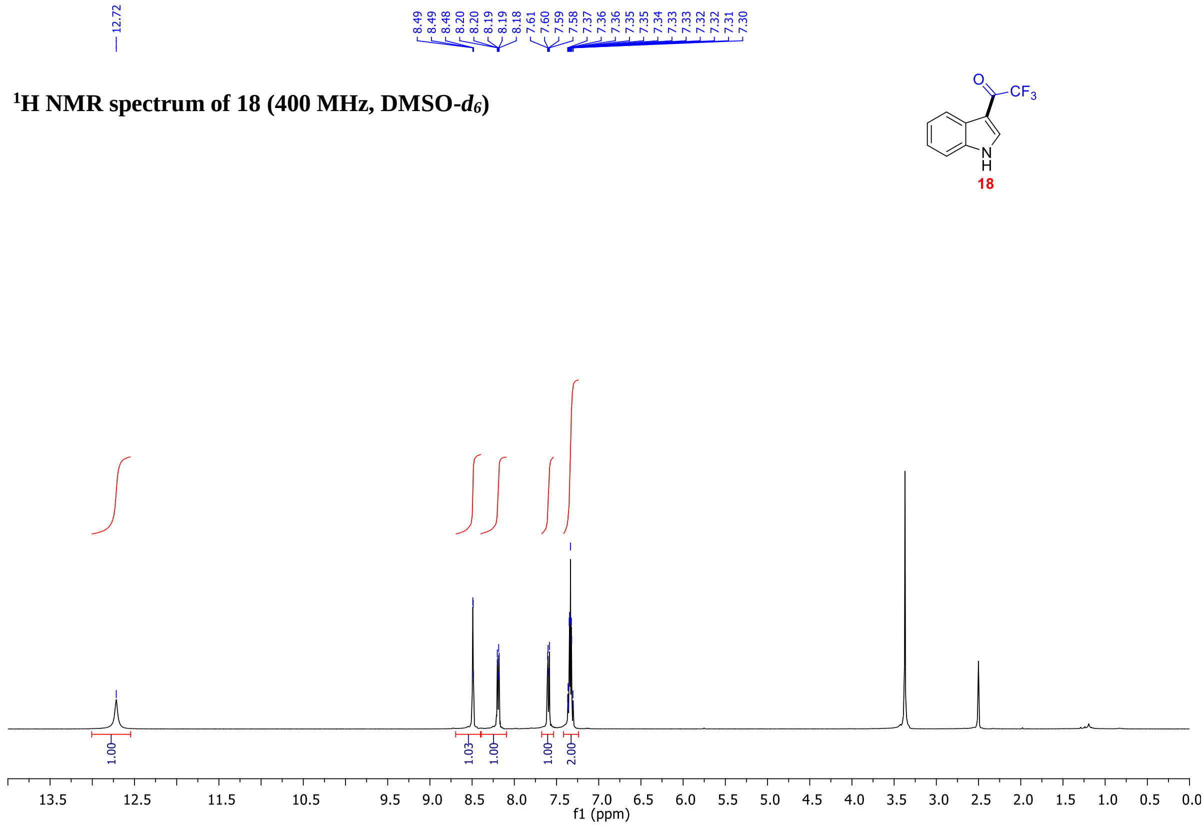

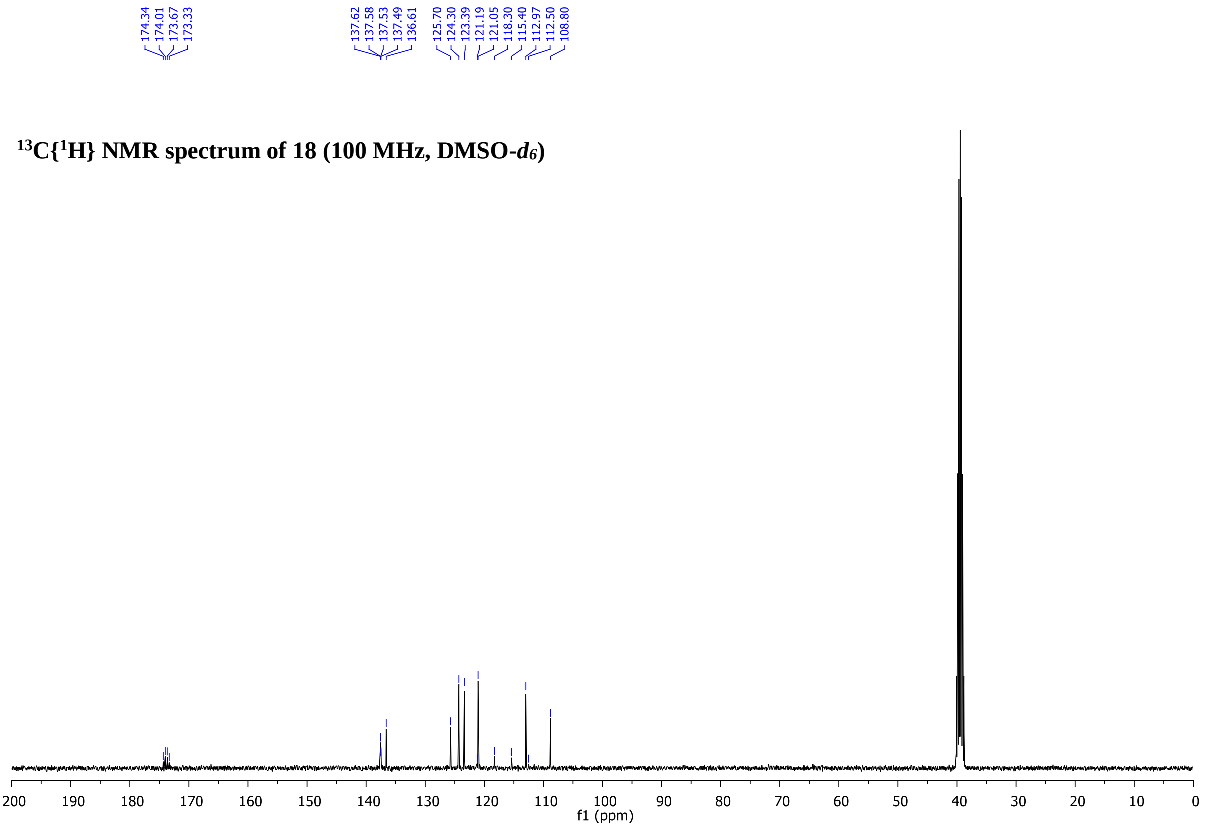

2-Methyl-1*H*-indole (19b)

^{1}H NMR spectrum of 19b (400 MHz, $CDCl_3$)

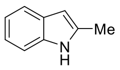

19c

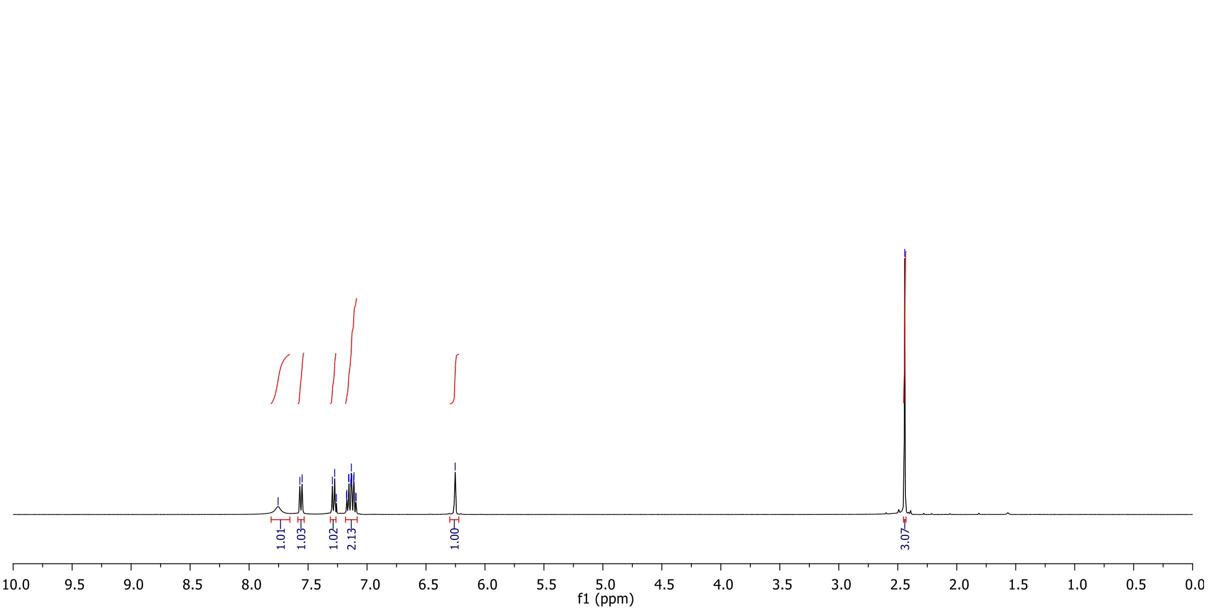

^{13}C{^{1}H} NMR spectrum of 19b (100 MHz, $CDCl_3$)

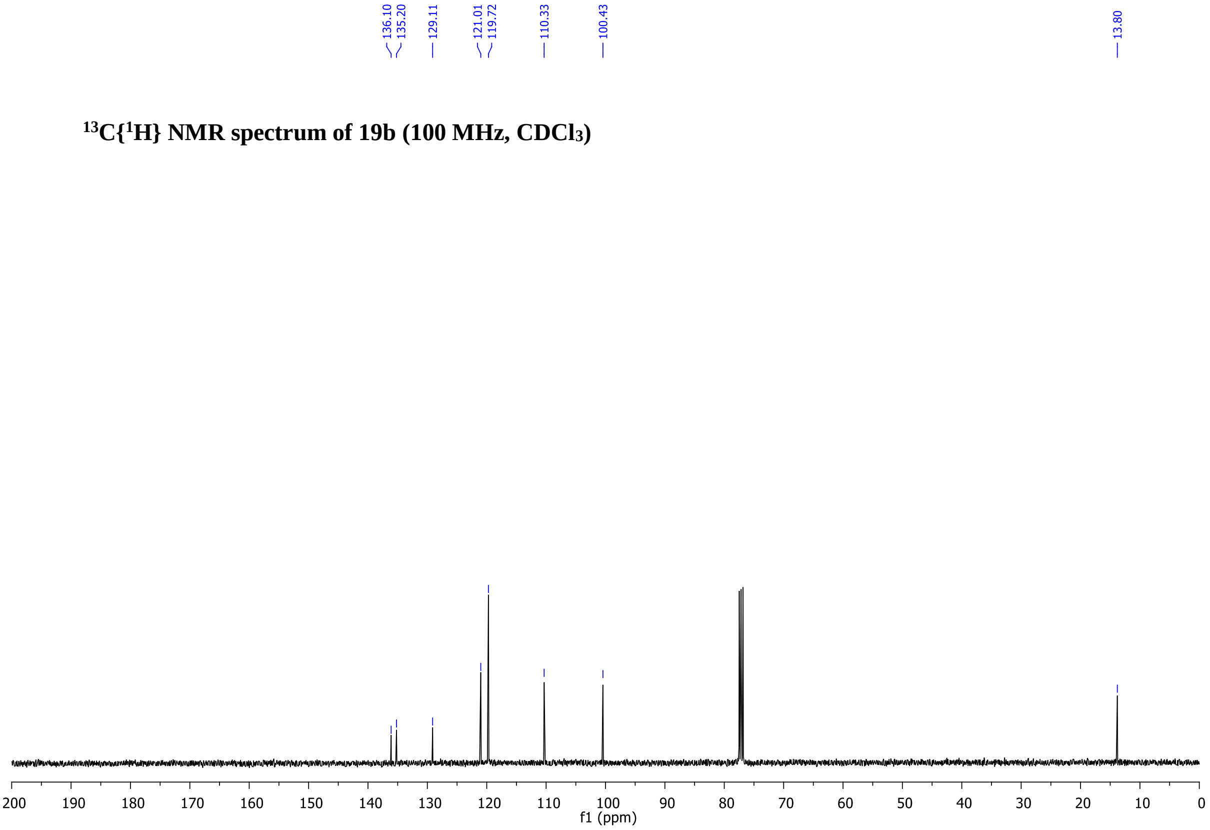

5. HRMS spectra of compounds

HRMS spectrum of **3aa**

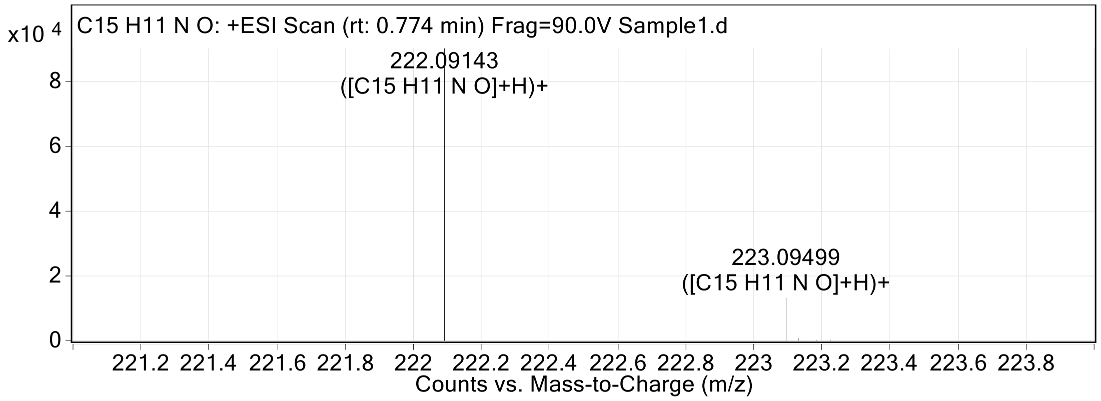

HRMS spectrum of **3ab**

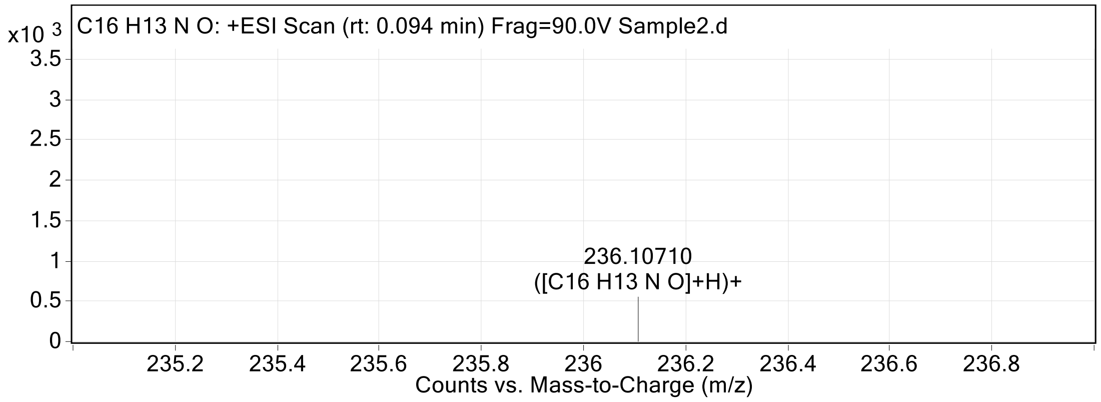

HRMS spectrum of **3ac**

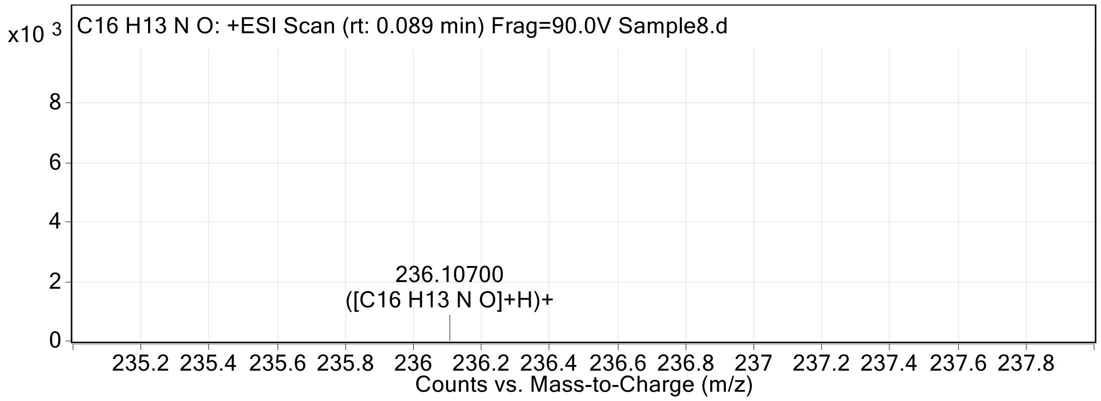

HRMS spectrum of **3ad**

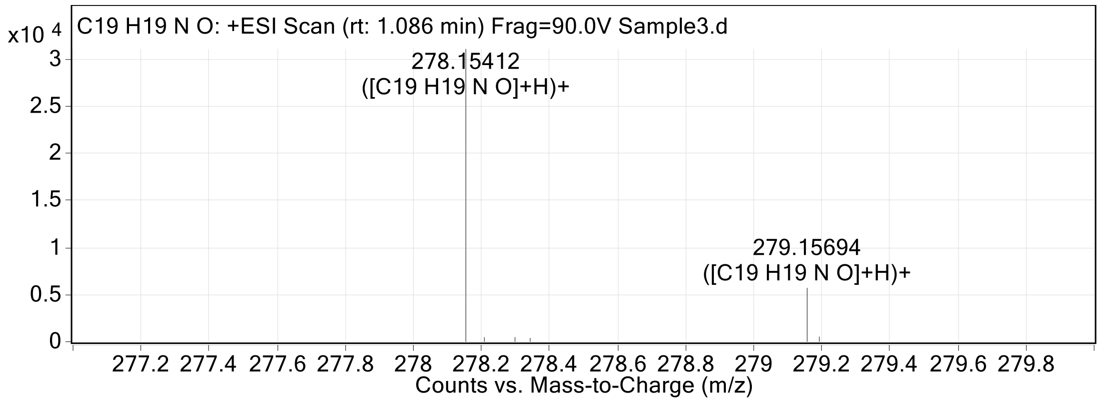

HRMS spectrum of **3ae**

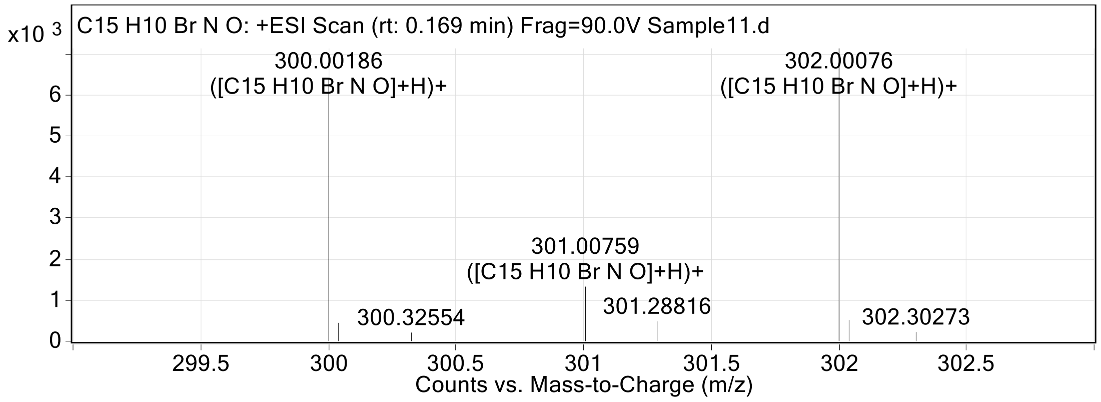

HRMS spectrum of **3af**

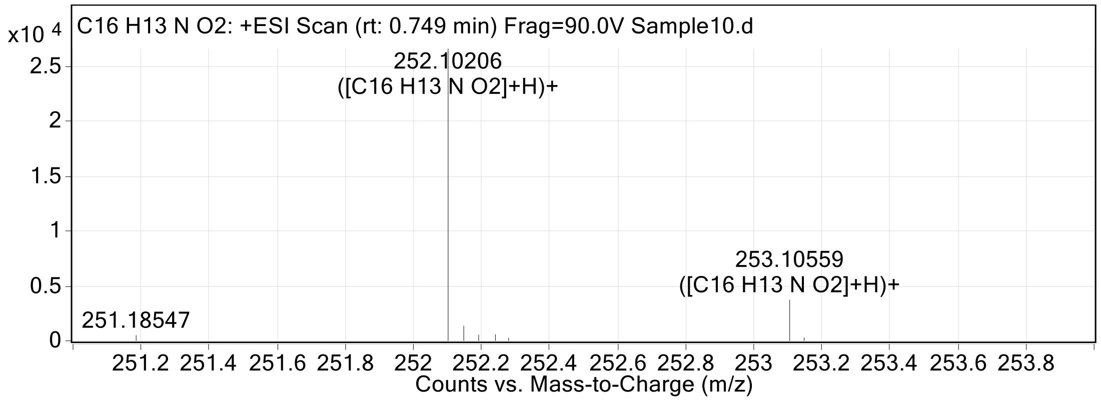

HRMS spectrum of **3ag**

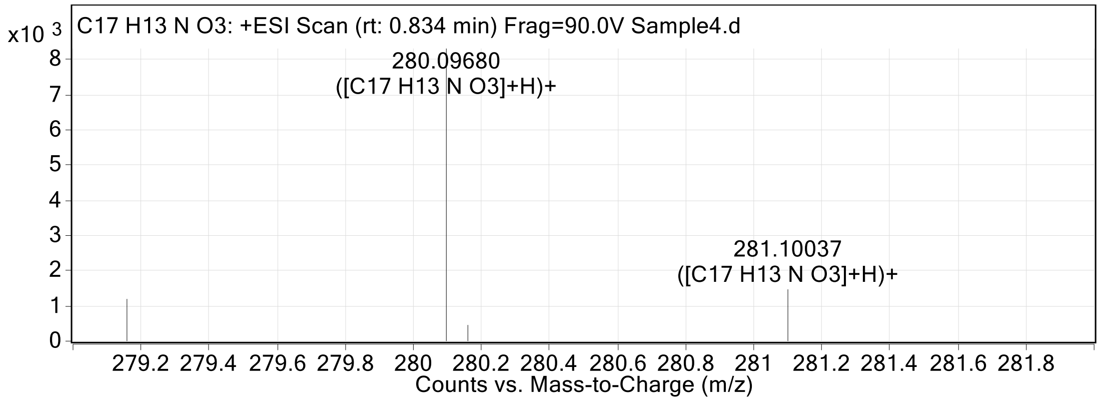

HRMS spectrum of **3ah**

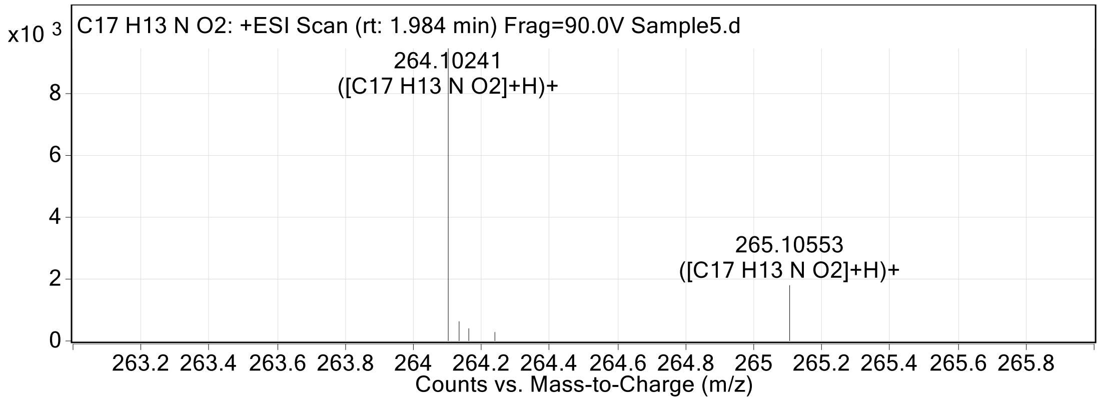

HRMS spectrum of **3ai**

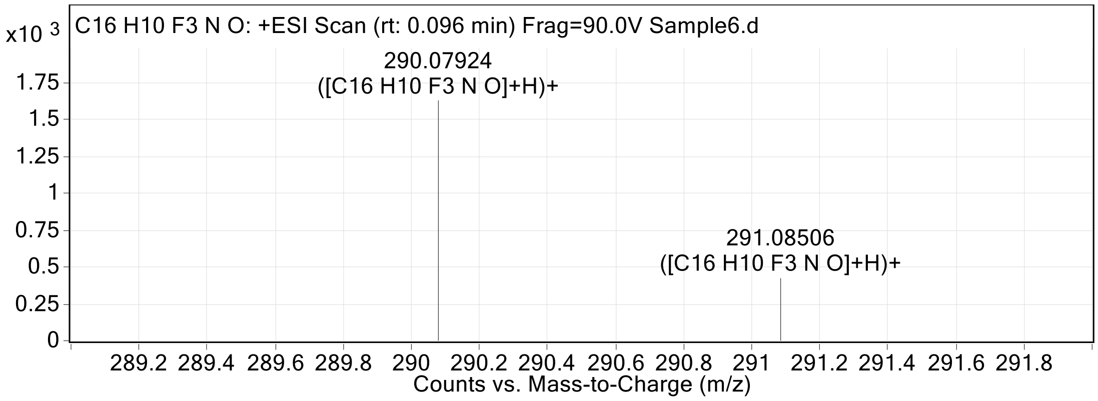

HRMS spectrum of **3al**

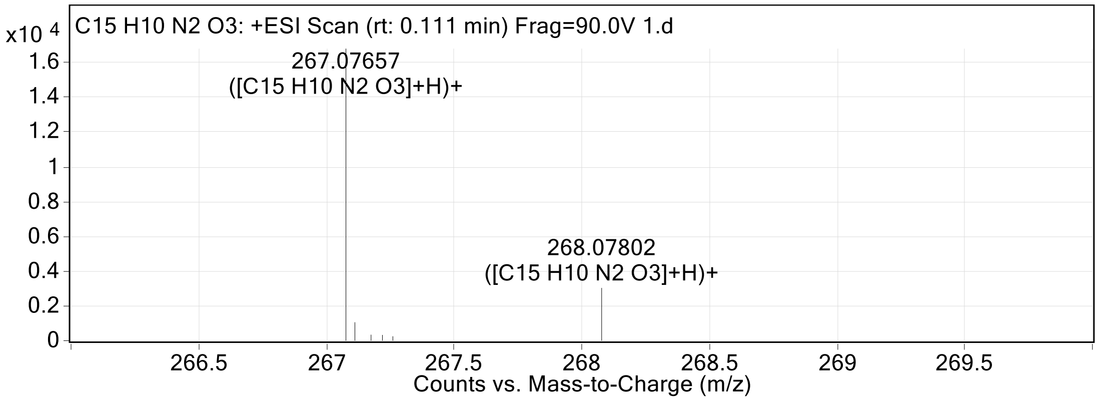

HRMS spectrum of **3ba**

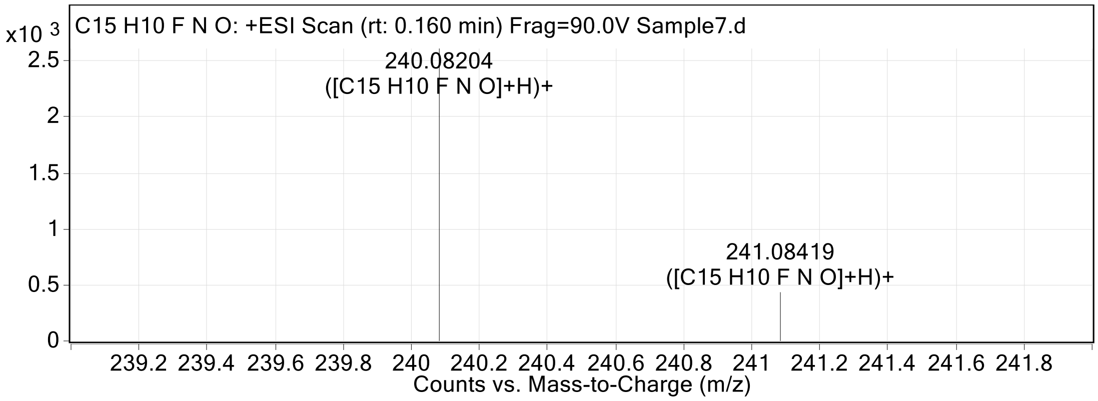

HRMS spectrum of **3ca**

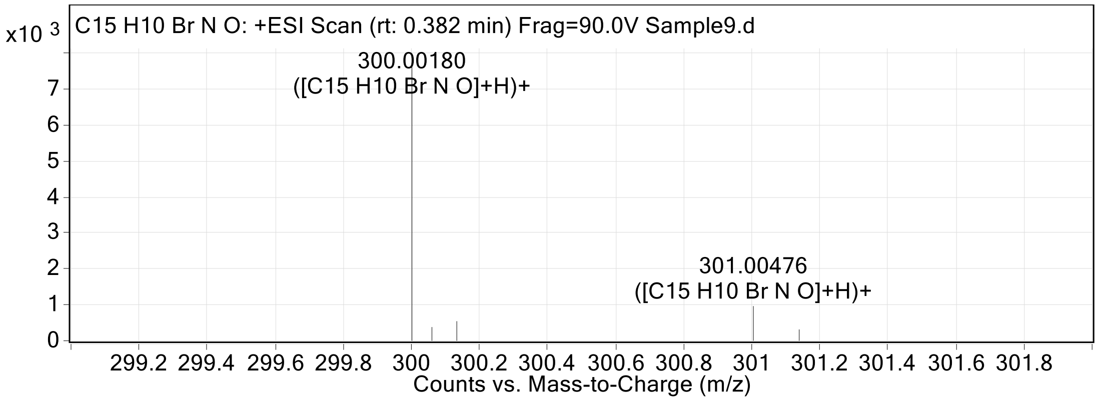

HRMS spectrum of **3da**

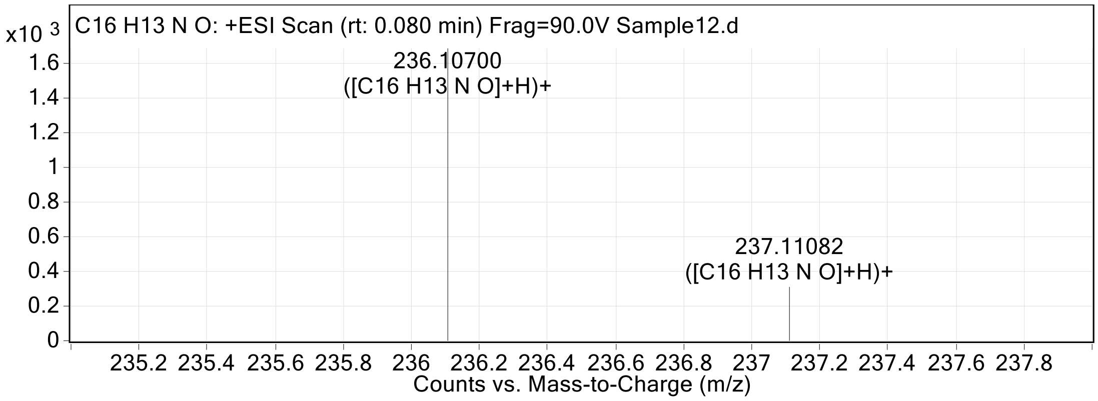

HRMS spectrum of **3ha**

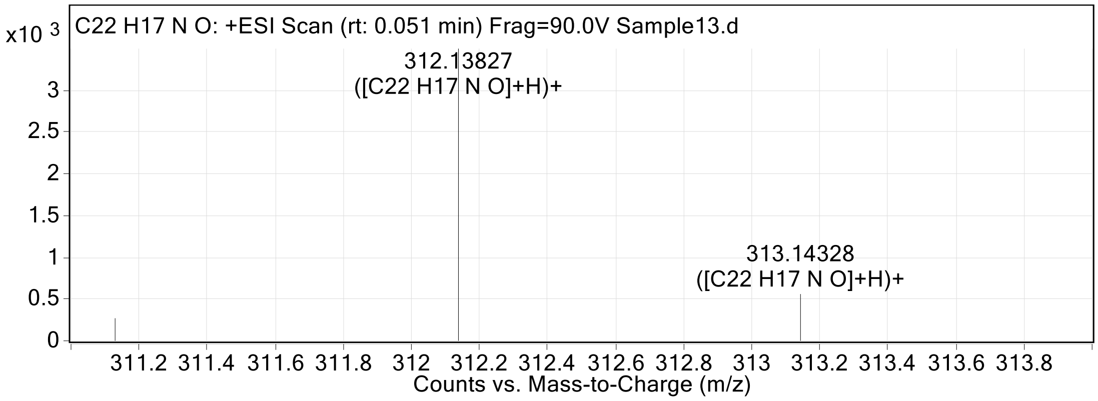

HRMS spectrum of **3ia**

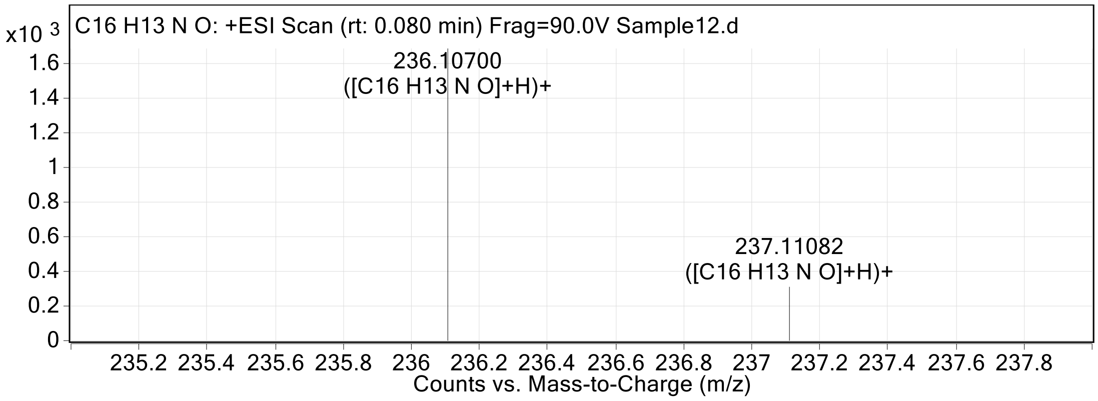

HRMS spectrum of **4b**

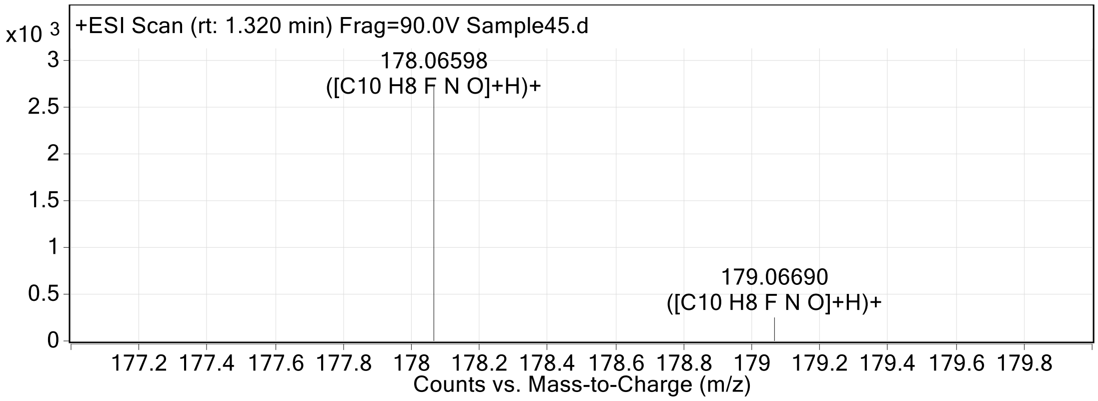

HRMS spectrum of **5aa**

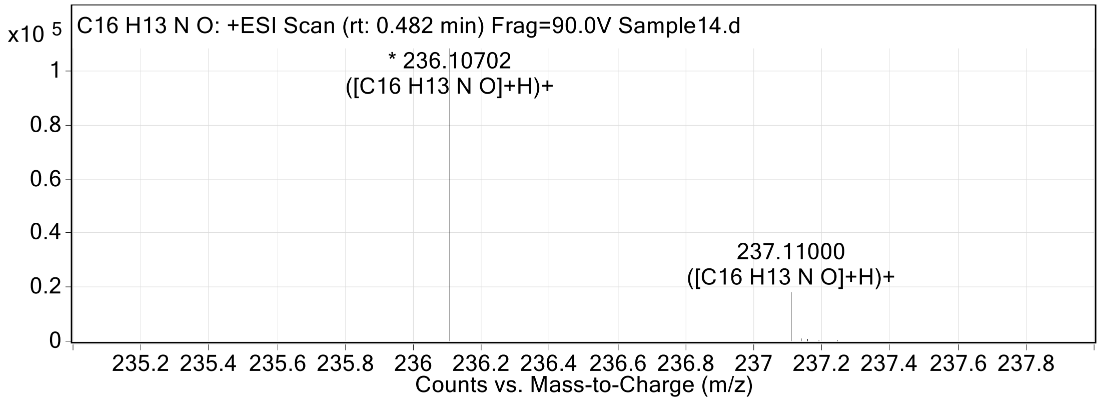

HRMS spectrum of **5ab**

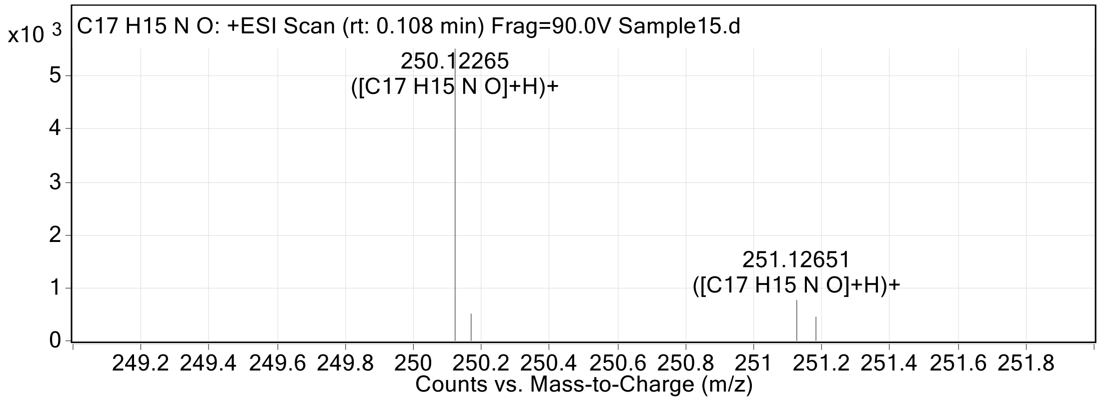

HRMS spectrum of **5ac**

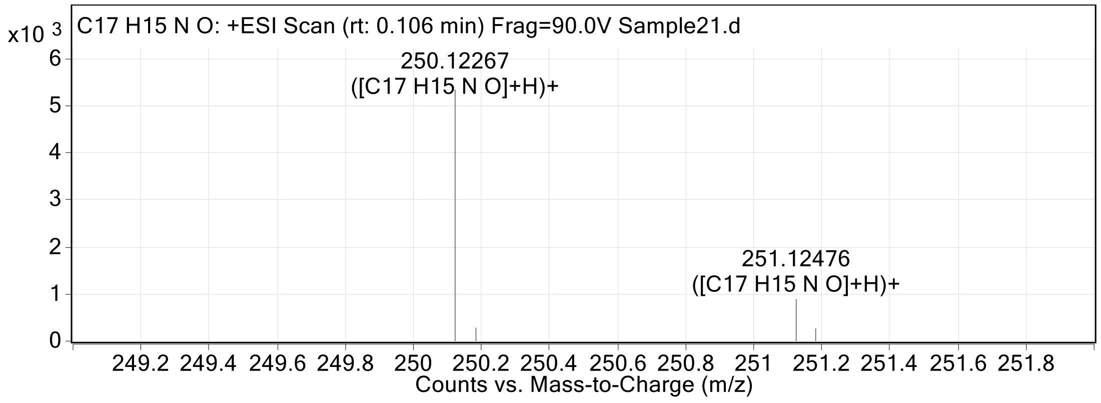

HRMS spectrum of **5ad**

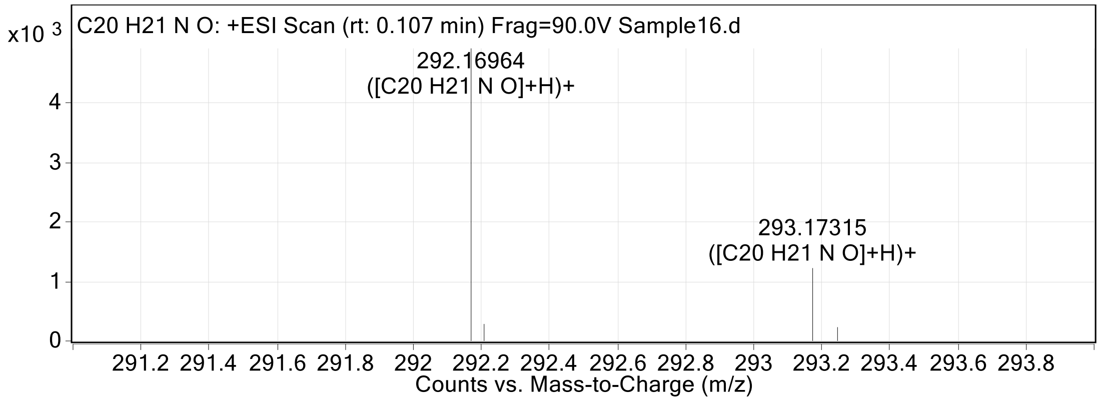

HRMS spectrum of **5ae**

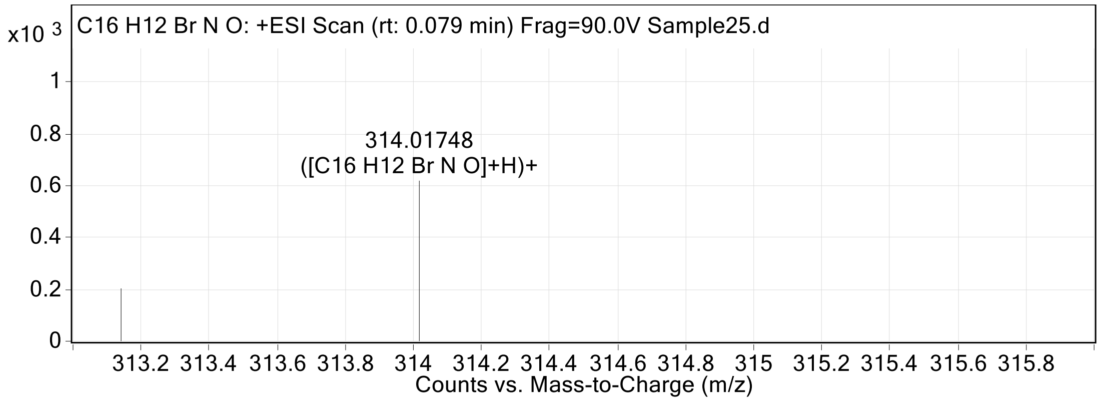

HRMS spectrum of **5af**

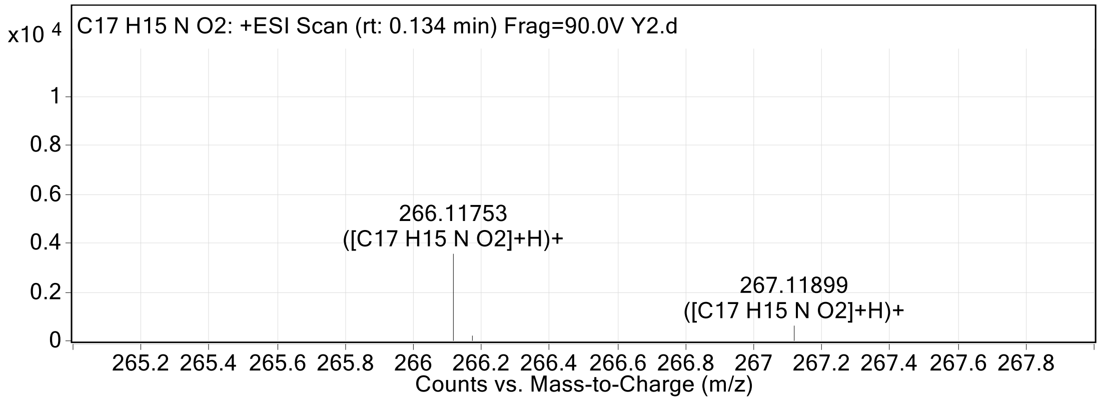

HRMS spectrum of **5ag**

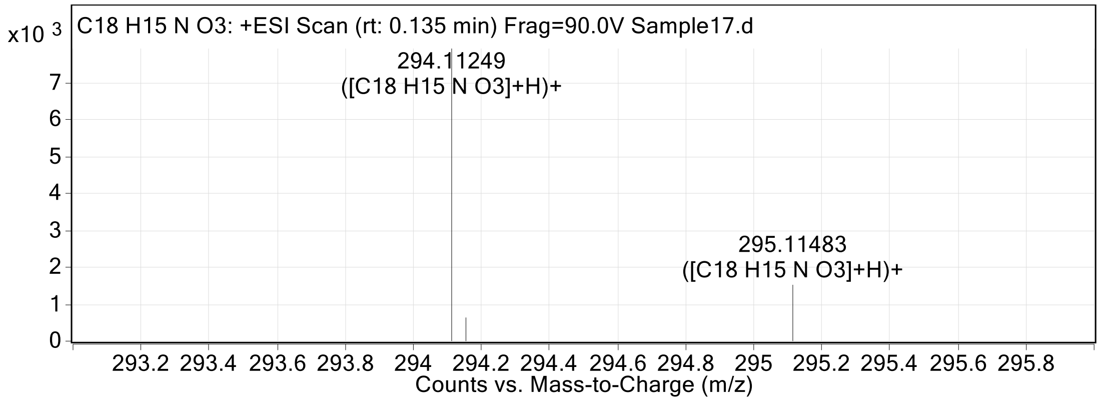

HRMS spectrum of **5ah**

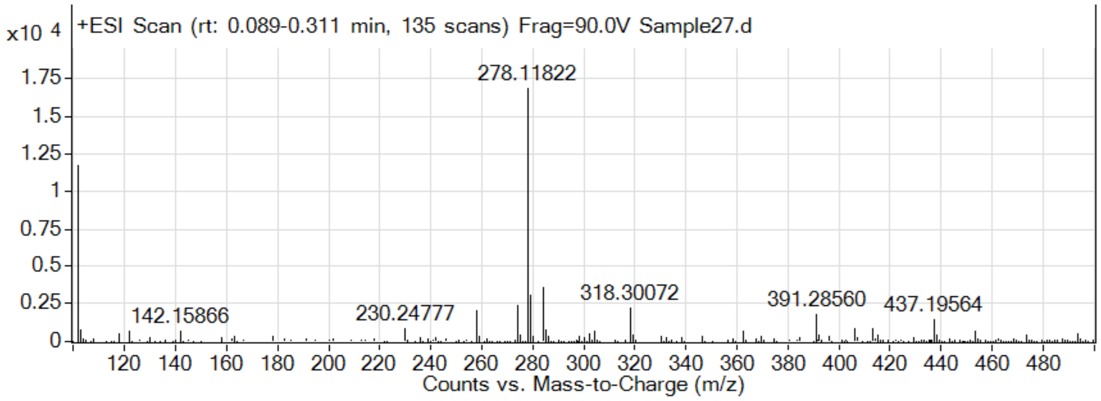

HRMS spectrum of **5ai**

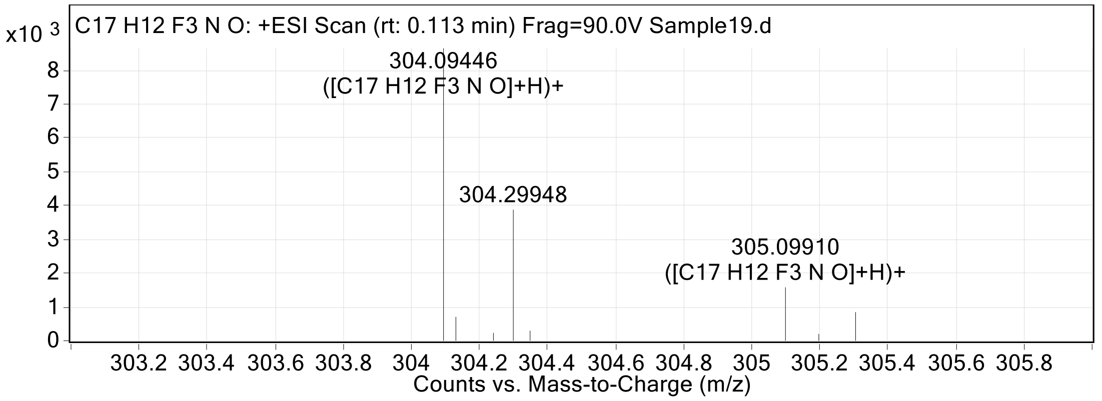

HRMS spectrum of **5ak**

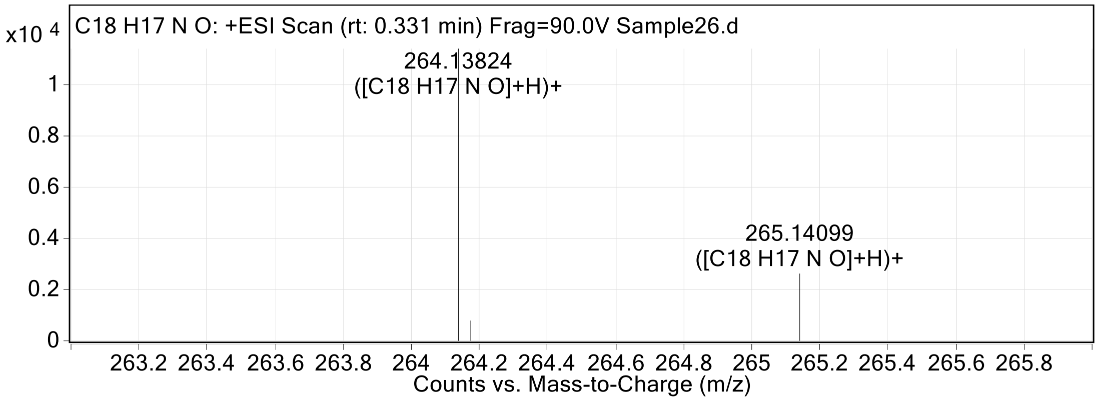

HRMS spectrum of **5al**

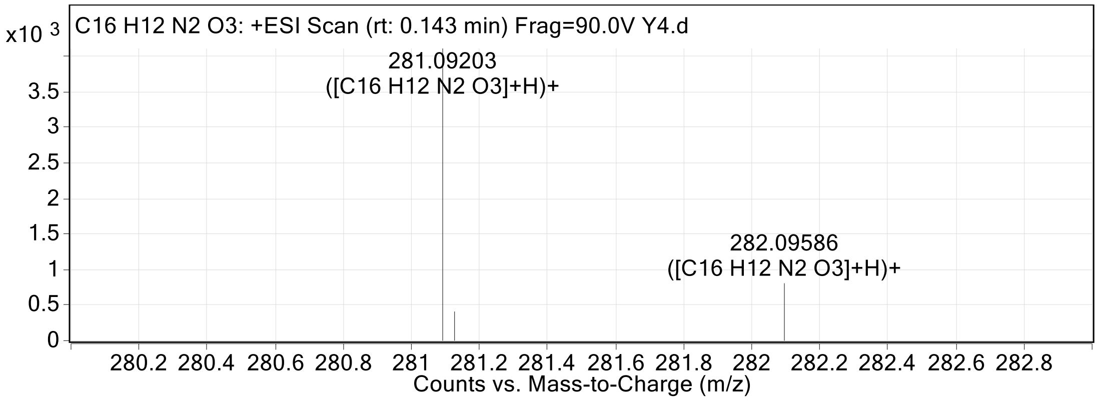

HRMS spectrum of **5ba**

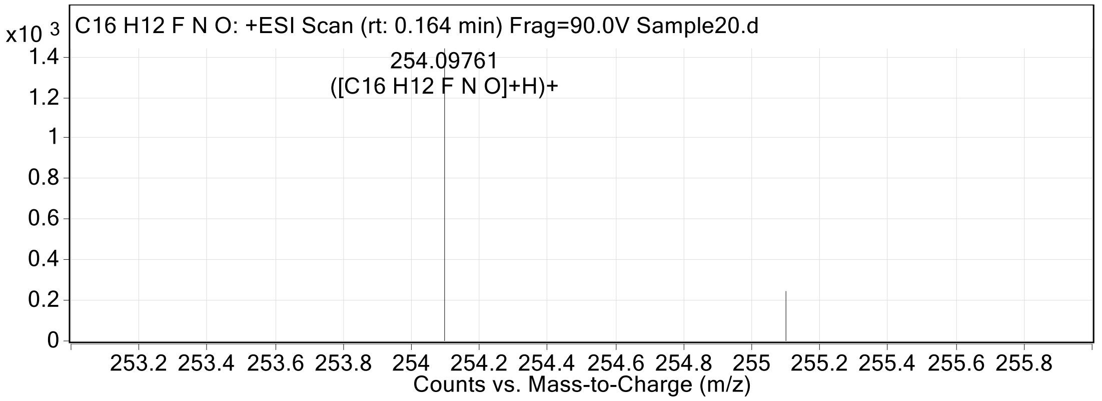

HRMS spectrum of **5ca**

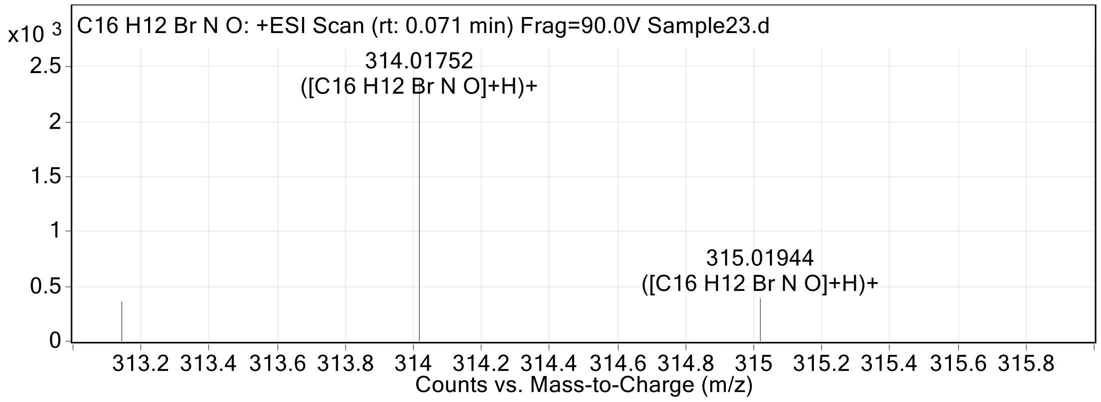

HRMS spectrum of **6ca**

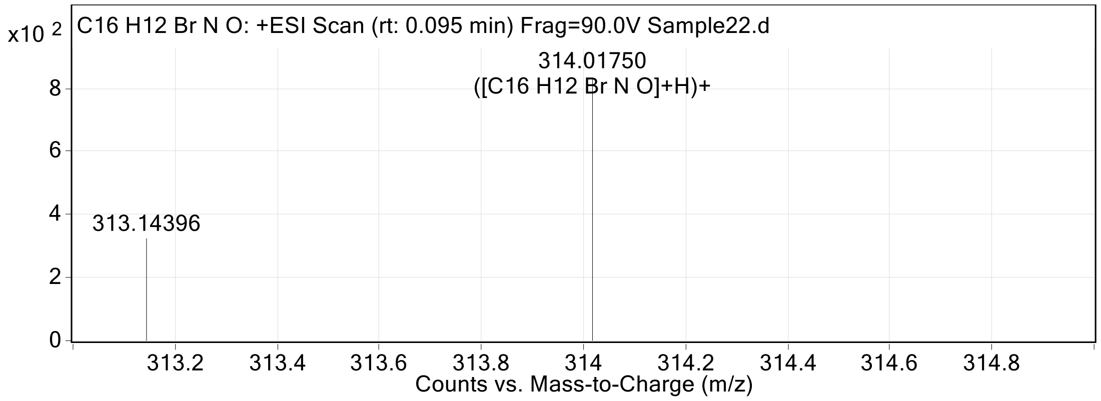

HRMS spectrum of **7da**

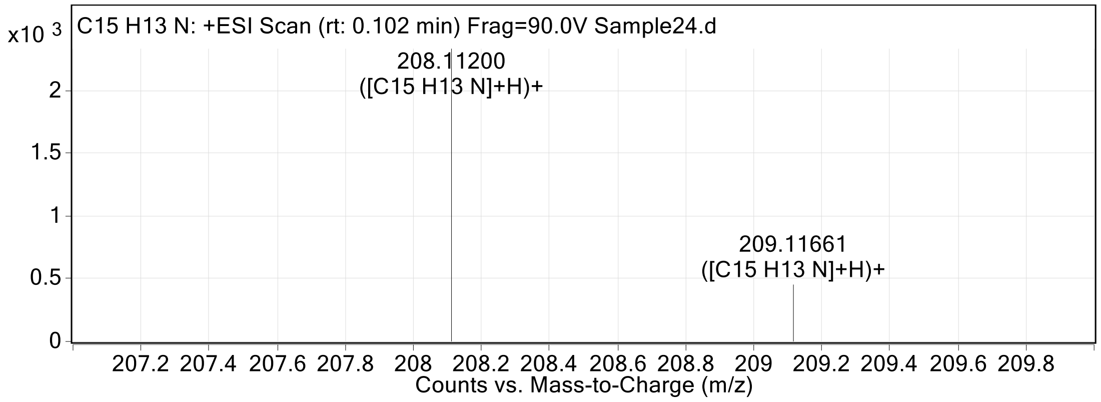

HRMS spectrum of **8b**

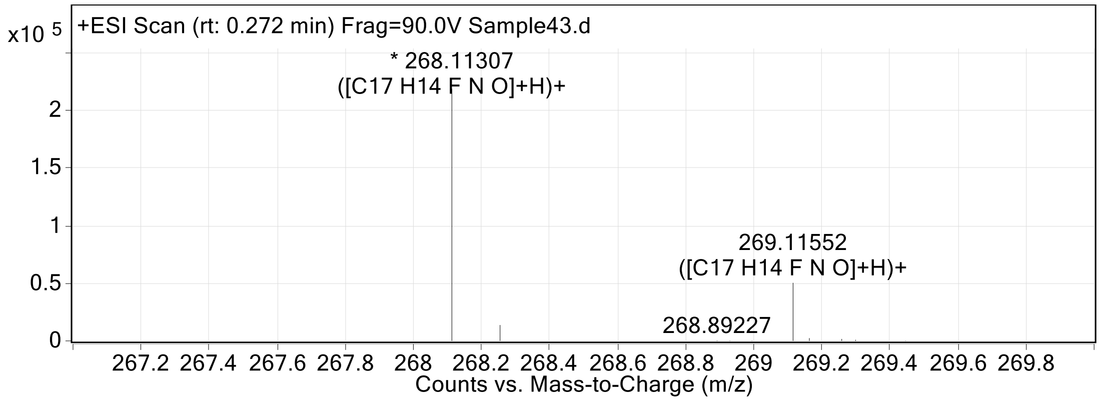

HRMS spectrum of **8c**

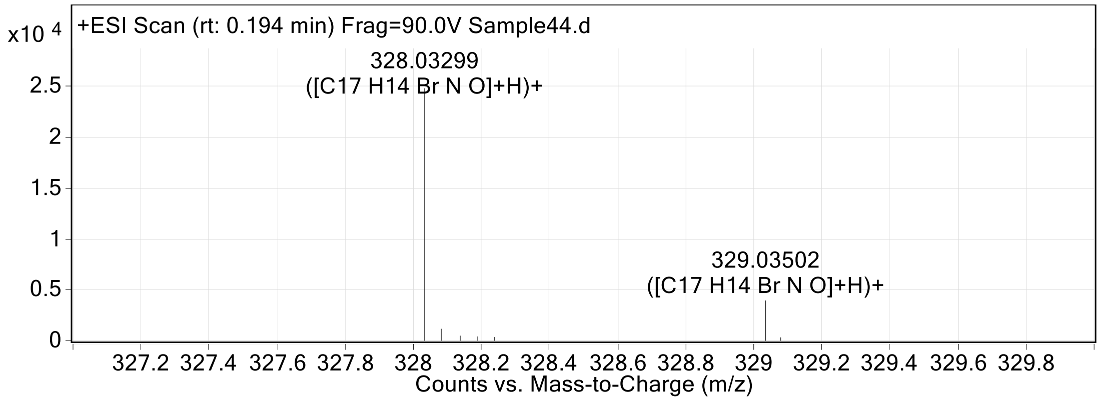

HRMS spectrum of **9aa**

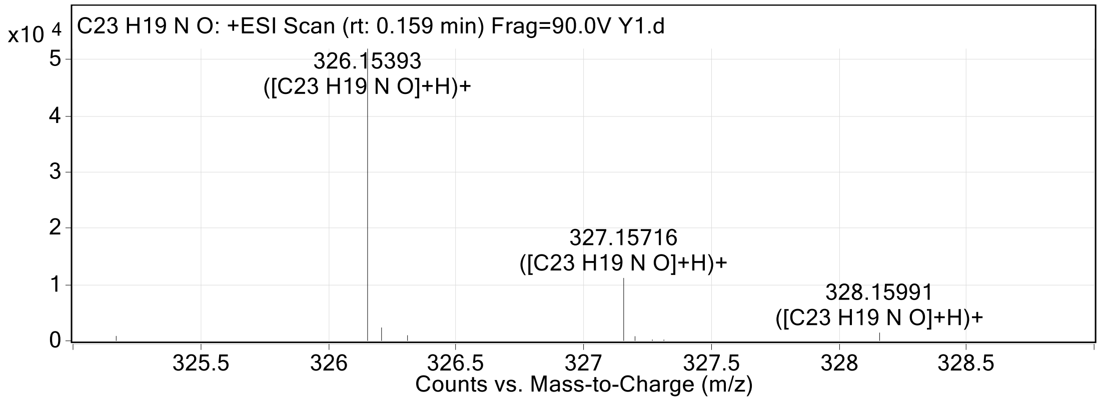

HRMS spectrum of **9ab**

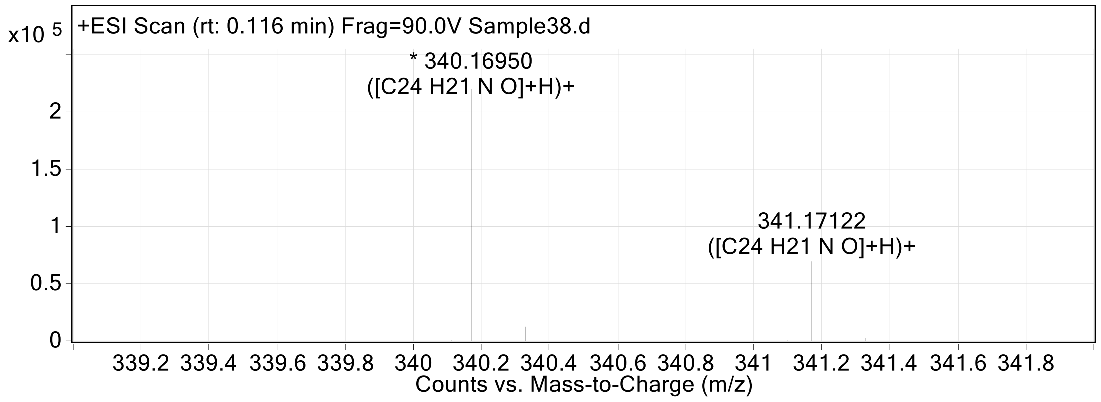

HRMS spectrum of **9ac**

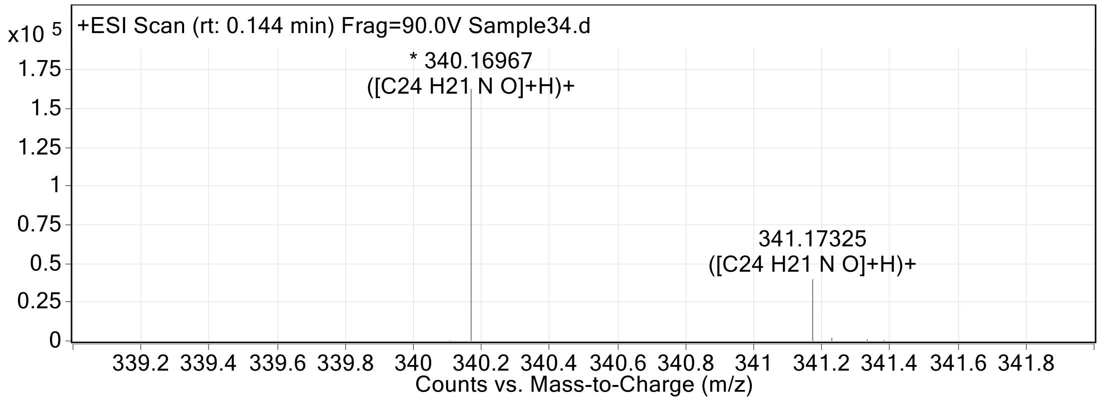

HRMS spectrum of **9ad**

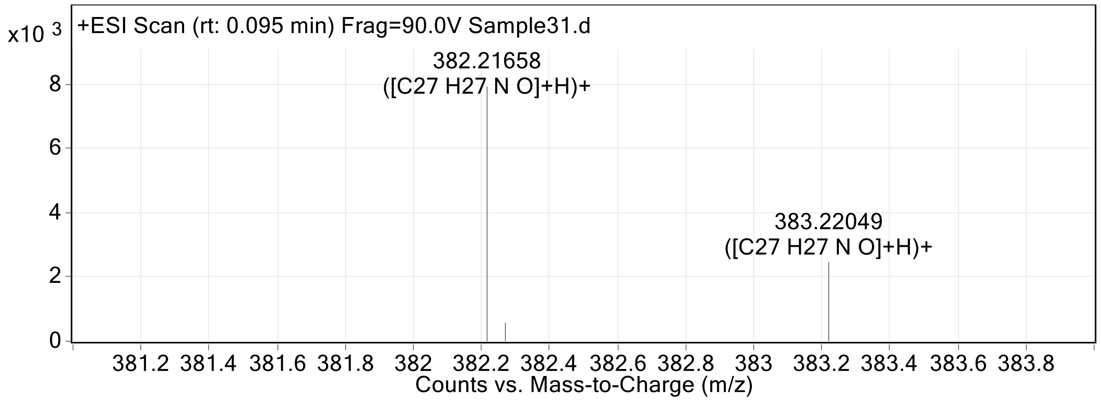

HRMS spectrum of **9ae**

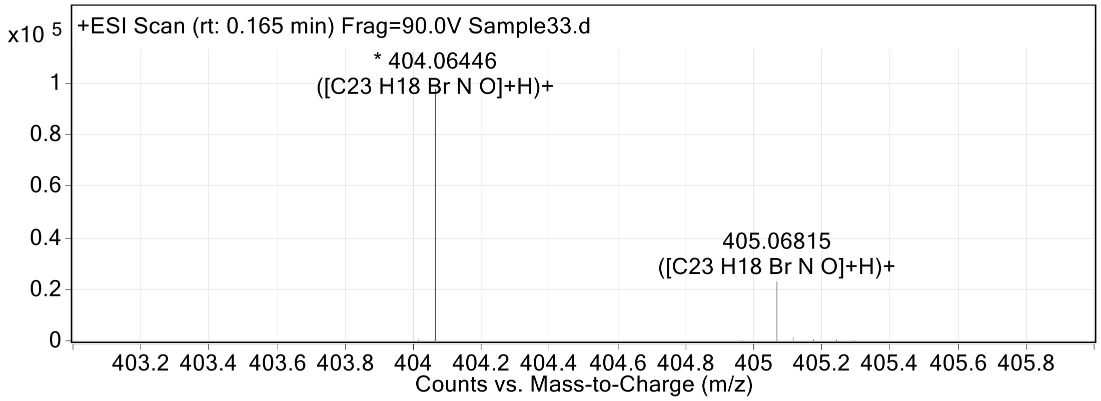

HRMS spectrum of **9af**

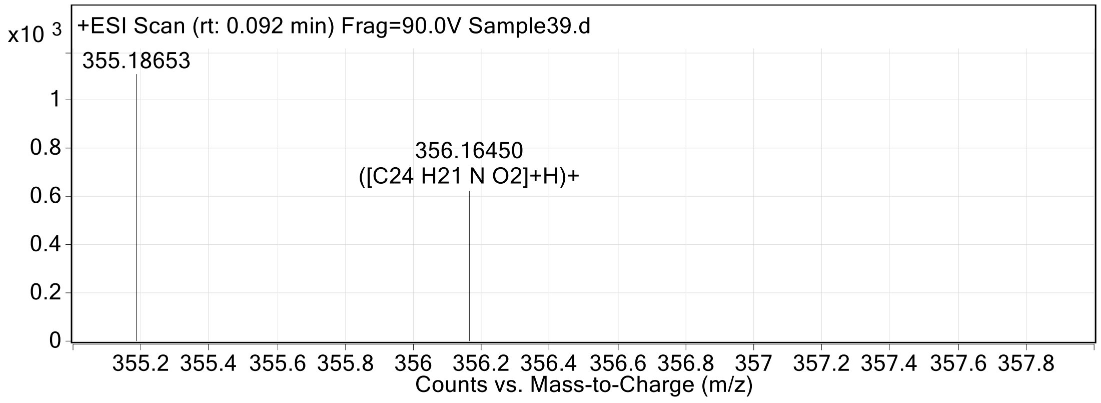

HRMS spectrum of **9ag**

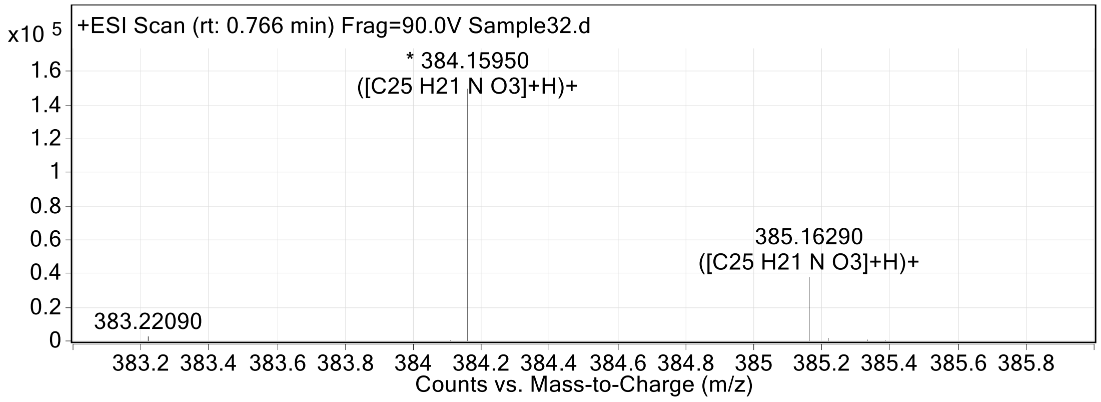

HRMS spectrum of **9ah**

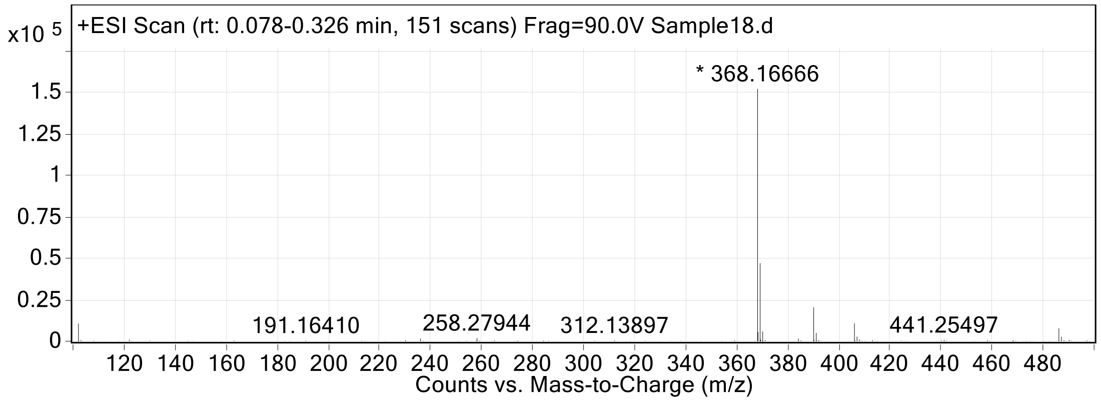

HRMS spectrum of **9ai**

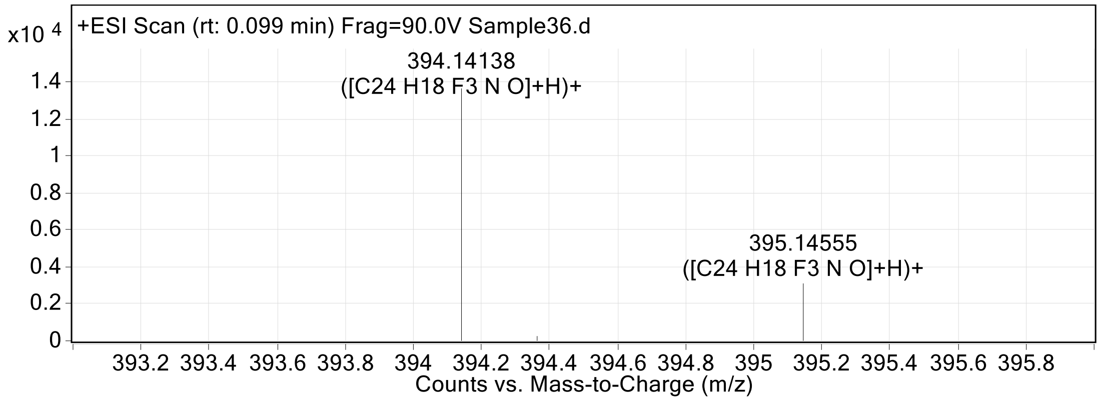

HRMS spectrum of **9ak**

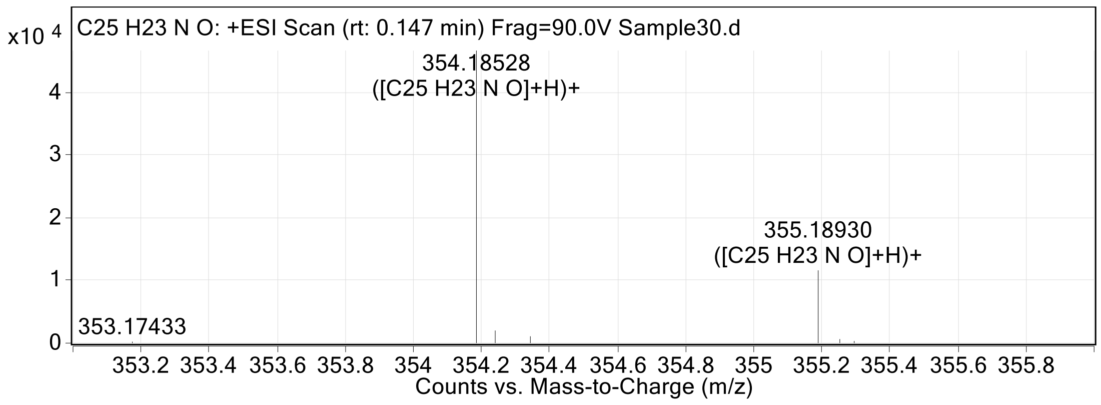

HRMS spectrum of **9al**

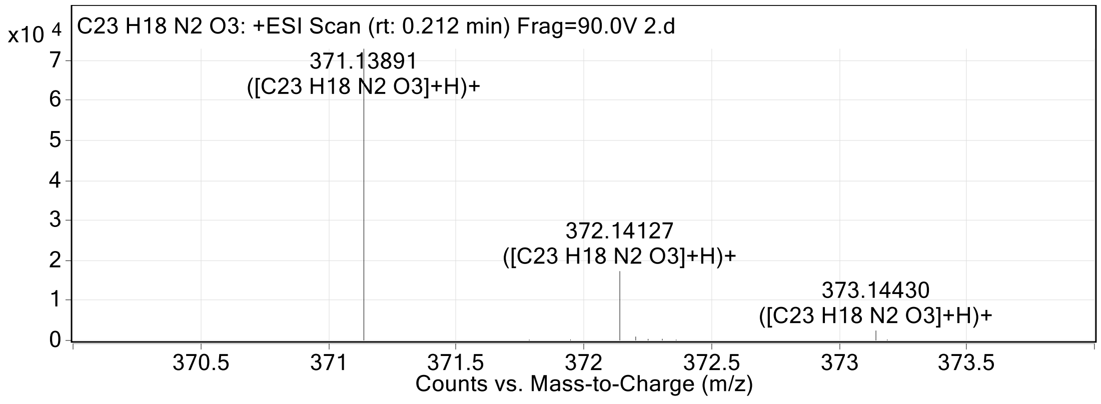

HRMS spectrum of **9ba**

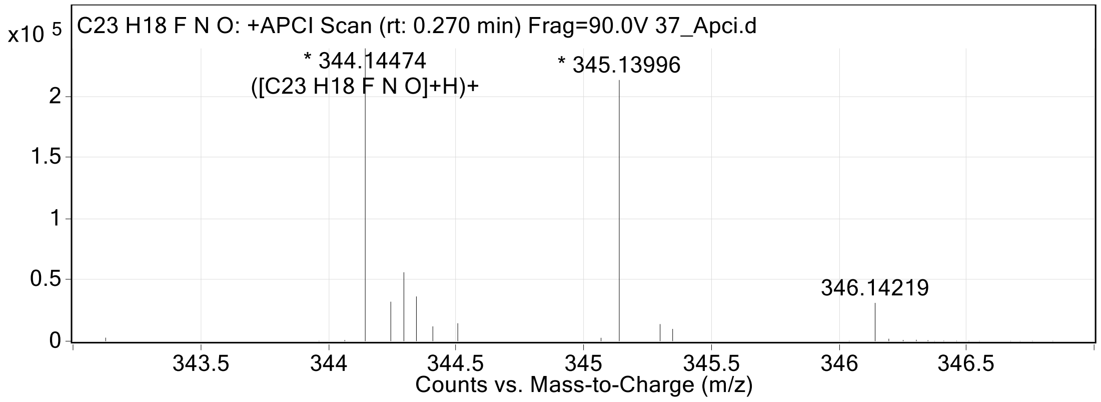

HRMS spectrum of **9ca**

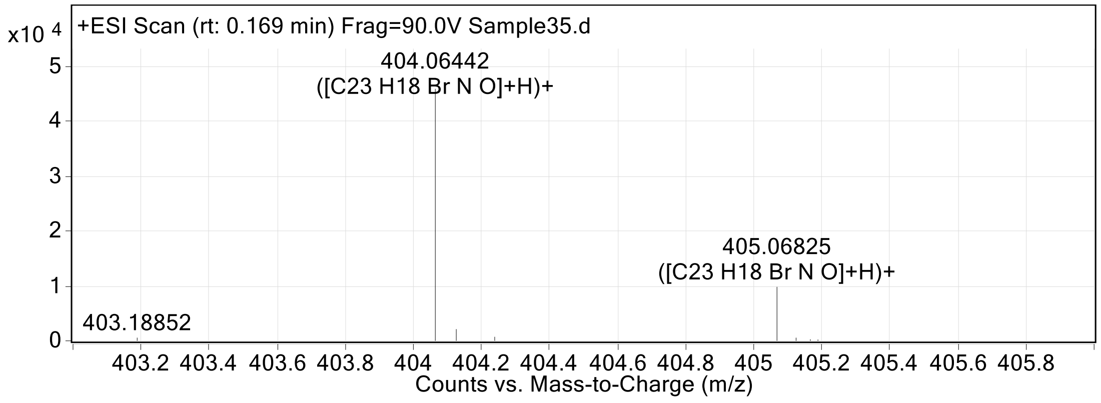

HRMS spectrum of **9ea**

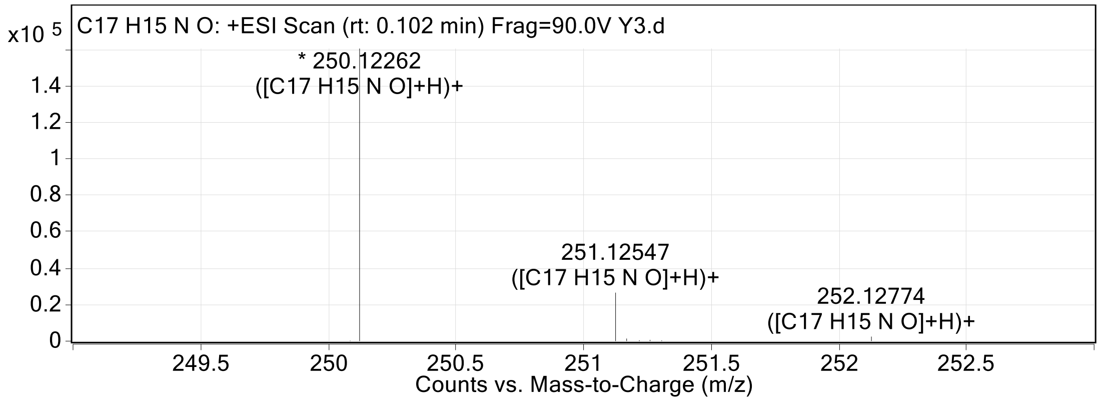

HRMS spectrum of **9eg**

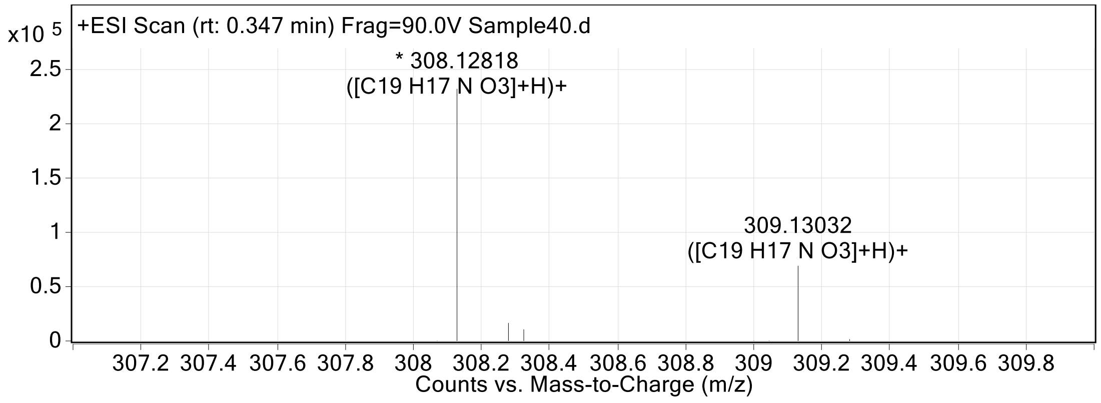

HRMS spectrum of **12**

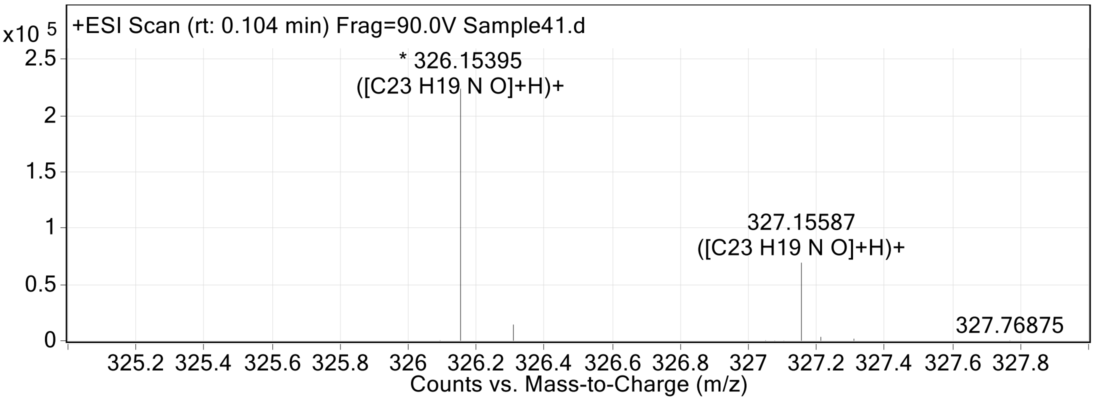

HRMS spectrum of **13**

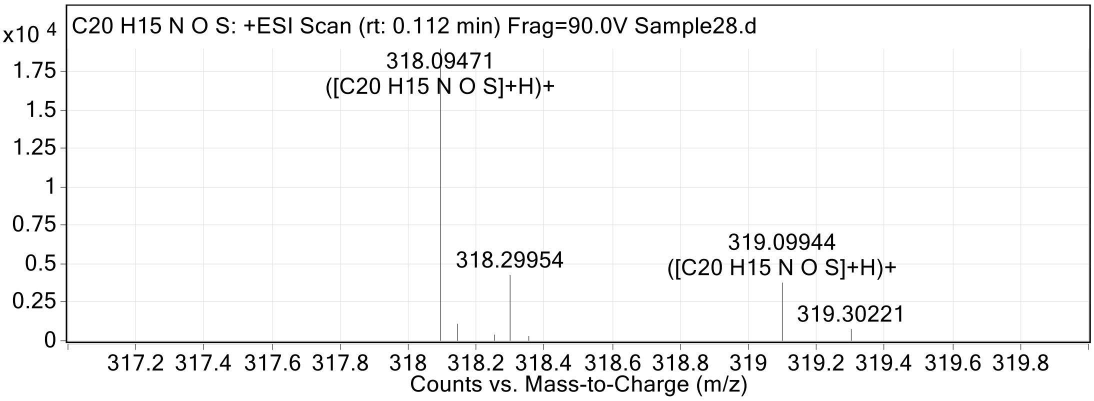

HRMS spectrum of **16**

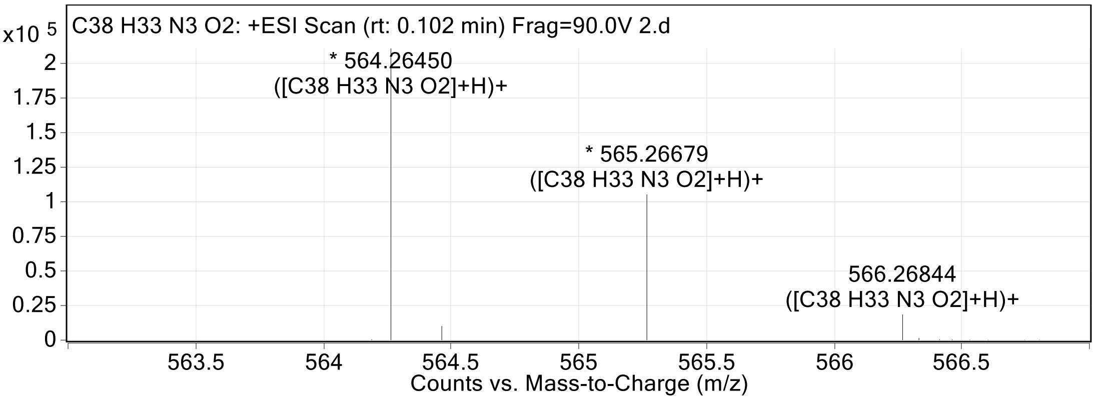

HRMS spectrum of **17**

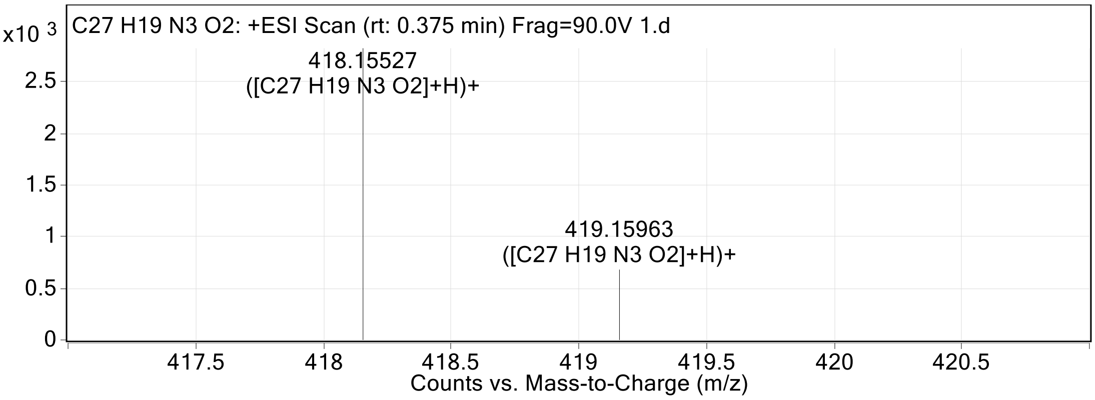

Supplement: Supplementary file 1 — jo2c00716_si_001.pdf [file jo2c00716_si_001.pdf]
